# Pangenomic and genomic plasticity analyses of the genus *Rickettsia*

**DOI:** 10.1007/s42770-026-02030-7

**Published:** 2026-07-24

**Authors:** Paula Cristina de Magalhães, Andrei Giacchetto Felice, Siomar de Castro Soares

**Affiliations:** UFTM: Universidade Federal do Triangulo Mineiro, Uberaba, Brazil

**Keywords:** Bioinformatics, Genomics, *Rickettsia*, Genes

## Abstract

The *Rickettsia* genus comprises obligate intracellular bacteria transmitted by arthropods and responsible for clinically relevant zoonoses, rickettsioses, such as spotted fever and typhus. The difficulty of cultivating these bacteria in vitro reinforces the importance of in silico approaches, such as pangenomic and genomic plasticity analyses. This study analyzed 165 genomes from 31 *Rickettsia* species available in the REFSEQ (NCBI) database. Tools such as Orthofinder, ANIclustermap, Gegenees, and Mauve were used to classify genes into core, shared, and singletons, assess genomic similarity, and identify structural rearrangements. The results indicate that the genus has an open pangenome (α = 0,842), suggesting high genetic variability and adaptive and expansion potential. Species such as *R. typhi* exhibited a nearly closed pangenome (α = 0,999), with high genomic conservation, whereas *R. rhipicephali* showed an open pangenome (α = 0,876), reflecting greater plasticity and intraspecies diversity. Functional categorization of genes revealed that the core genome is associated with vital functions, while singletons include genes related to genetic mobility, indicating possible acquisition through horizontal transfer. Synteny analysis demonstrated high gene conservation in *R. typhi* and extensive structural reorganization in *R. rhipicephali*. Statistical correlation reinforced the stability of the core genome regardless of pangenome expansion and revealed an inverse relationship between the number of singletons and the value of α. The findings demonstrate the existence of contrasting evolutionary trajectories within the *Rickettsia* genus, with conserved, specialized species coexisting alongside genetically dynamic species that are more adaptable to different niches. Thus, this study expands the understanding of clonality, genomic plasticity, and functional diversity within the genus, providing support for future investigations into virulence factors, vaccine targets, and bacterial evolution.

**Supplementary Information:**

The online version contains supplementary material available at 10.1007/s42770-026-02030-7.

## Introduction

The *Rickettsia* genus was first described in 1909, following the initial isolation of a species by Ricketts in 1906. Since then, it has been characterized as an obligate intracellular organism, which limits its in vitro cultivation and requires specific laboratory technology for experimental studies [1]. Bacteria of this genus are Gram-negative, small (0.3–0.5 μm in diameter and 0.8–2.0 μm in length), rod-shaped or coccoid, belonging to the family Rickettsiaceae, and transmitted by arthropod vectors such as ticks, fleas, lice, or mites from domestic or wild vertebrate hosts. These bacteria live and replicate intracellularly within host cells in vivo in mammals, mainly in endothelial cells [2].

The genus is classified into four groups, with the main disease-causing agents that affect human populations belonging to two of them: the Spotted Fever Group (SFG), which includes *Rickettsia conorii*, *Rickettsia massiliae*, *Rickettsia rickettsii*, *Rickettsia africae*, and *Rickettsia parkeri*, *Rickettsia fournieri*,* Rickettsia helvetica*,* Rickettsia honei*,* Rickettsia hoogstraalii*,* Rickettsia tamurae*, among 32 other species; and the Typhus Group (TG), comprising *Rickettsia prowazekii* and *Rickettsia typhi*. In addition, there are two other groups: the Ancestral Group, composed of the ancestral bacteria *Rickettsia bellii* and *Rickettsia canadensis*, and the Transitional Group, which includes *Rickettsia akari*, *Rickettsia felis*, and *Rickettsia australis* [3–11].

The Typhus and Spotted Fever groups include species responsible for diseases collectively known as rickettsioses, zoonotic infections that affect both humans and animals. The main pathogenic species are *R. typhi*, *R. prowazekii*, *R. rickettsii*, and *R. conorii*, which cause murine typhus, epidemic typhus, Rocky Mountain spotted fever, and Mediterranean spotted fever, respectively [12, 13].

Rickettsial infections are an important cause of undifferentiated febrile illness, particularly in endemic regions of developing and underdeveloped countries with poor sanitation and hygiene conditions. They are especially prevalent in tropical and subtropical climates, where socioeconomically vulnerable environments favor the proliferation of vectors responsible for *Rickettsia spp*. transmission [14, 15]. Organisms of this genus are present on all continents except Antarctica, as shown in SUPPLEMENTARY MATERIAL A by the study of Abdad and collaborators [16], which supports the information that the ecological profile of vectors, including climate, ecosystem, and geographic expansion, directly influences the distribution of *Rickettsia* species [16].

In the laboratory, rickettsiae can be cultivated only within viable eukaryotic host cells, such as in cell cultures, embryonated eggs, or susceptible animals. Consequently, the difficulty in studying these organisms highlights the need for bioinformatics tools to analyze such bacteria. Therefore, it is important to emphasize the role of pangenomic analyses, which are characterized as studies of the complete gene repertoire of a given group, aiming to identify genomic similarities and differences across all analyzed genomes [17].

After identifying these genes, in silico tools are used to classify them into three pangenome subgroups. The core genome comprises conserved genes present in all analyzed genomes and generally responsible for essential cellular functions; the shared genome includes genes found in two or more lineages or species, though not universal among all of them; and the singletons correspond to genes specific to a single species or lineage, depending on the level of analysis, whether at the genus or species level [18].

In this context, it is noteworthy that rickettsioses are difficult to diagnose due to their symptomatology being similar to that of other tropical diseases. Nevertheless, studies have shown that a significant number of diseases worldwide are caused by rickettsiae. Thus, this study is essential to broaden our understanding of the *Rickettsia* species responsible for rickettsioses, neglected diseases that remain of great relevance today [14, 15]. Therefore, the present study aimed to perform a genus-level pangenomic analysis using well-established tools described in the literature, with the objective of identifying the clonality and genomic plasticity of the *Rickettsia* genus. Specifically, the study sought to classify clusters of orthologous genes into the pangenome subsets: core genome, shared genome and singletons, and to characterize them, conduct phylogenomic and gene synteny analyses, and fit the development curves of the pangenome, core genome and singletons in order to assess the patterns of clonality and variability among the species belonging to the genus.

## Materials and methods

### Samples

Ethical approval was not required for this study, as it did not involve the use of primary human data or experimentation with vertebrate animals that would necessitate ethical evaluation, being conducted entirely in silico.

For the pangenomic analyses, 165 complete genomes from 31 *Rickettsia* species (Table 1) were used (Accessed in September, 2024). These genomes are publicly available in the Reference Sequence Database (RefSeq) of the National Center for Biotechnology Information (NCBI – https://www.ncbi.nlm.nih.gov). The extended table containing all information related to each species of the genus can be found in SUPPLEMENTARY MATERIAL A, Table 3.

**Table 1:** Comparative summary of species of the *Rickettsia* genus

Species	No. of Genomes	Group	Genome Size Range (MB)	GC Content Range (%)	Protein Count Range
*Rickettsia africae*	1	Spotted Fever Group	1,30	32,5	1343
*Rickettsia akari*	1	Transitional Group	1,20	32,5	1124
*Rickettsia amblyommatis*	5	Spotted Fever Group	1,43 − 1,50	32,5–33,0	1321–1431
*Rickettsia argasii*	1	-	1,43	32,5	1374
*Rickettsia asembonensis*	2	-	1,37 − 1,42	32,0	1359–1504
*Rickettsia asiatica*	1	-	1,41	32,5	1436
*Rickettsia australis*	2	Transitional Group	1,32	32,5	1278–1282
*Rickettsia bellii*	5	Ancestral Group	1,52 − 1,61	31,5	1439–1491
*Rickettsia canadensis*	2	Ancestral Group	1,15 − 1,16	31,0	916–944
*Rickettsia conorii*	17	Spotted Fever Group	1,25 − 1,48	32,5	1237–1505
*Rickettsia felis*	3	Transitional Group	1,49 − 1,58	32,5	1352–1395
*Rickettsia fournieri*	1	Spotted Fever Group	1,44	32,5	1391
*Rickettsia gravesii*	1	-	1,35	32,0	1372
*Rickettsia helvetica*	3	Spotted Fever Group	1,41 − 1,42	32,0	1386–1442
*Rickettsia honei*	1	Spotted Fever Group	1,27	32,5	1318
*Rickettsia hoogstraalii*	2	Spotted Fever Group	1,48 − 1,57	32,5	1479–1544
*Rickettsia japonica*	35	Spotted Fever Group	1,27 − 1,28	32,5	1294–1329
*Rickettsia massiliae*	2	Spotted Fever Group	1,28 − 1,38	32,5	1259–1370
*Rickettsia monacensis*	2	Spotted Fever Group	1,28 − 1,35	32,5	1298
*Rickettsia parkeri*	5	Spotted Fever Group	1,30 − 1,35	32,5	1340–1394
*Rickettsia peacockii*	1	Spotted Fever Group	1,31	32,5	1313
*Rickettsia philipii*	1	Spotted Fever Group	1,29	32,5	1328
*Rickettsia prowazekii*	12	Typhus Group	1,11	29,0	795–836
*Rickettsia rhipicephali*	4	Spotted Fever Group	1,27 − 1,45	32,5	1263–1405
*Rickettsia rickettsii*	26	Spotted Fever Group	1,26 − 1,29	32,5	1300–1357
*Rickettsia sibirica*	11	-	1,25 − 1,28	32,5	1265–1360
*Rickettsia slovaca*	2	Spotted Fever Group	1,27	32,5	1359–1361
*Rickettsia sp.*	8	-	1,17 − 1,38	30,5–33,0	915–1405
*Rickettsia tamurae*	3	Spotted Fever Group	1,45 − 1,78	32,5	1499–1683
*Rickettsia tillamookensis*	1	Transitional Group	1,44	32,5	1286
*Rickettsia typhi*	4	Typhus Group	1,11	29,0	806–811

Source: By author, 2025. Note: Comparative summary of *Rickettsia* species, including the number of analyzed genomes, the phylogenetic or clinical group to which they belong, and the observed variations in key genomic parameters: genome size (in megabases), GC content variation (percentage of guanine and cytosine bases in a DNA or RNA sequence), and the variation in the number of encoded proteins. The “-” sign represents species that have not been classified in the literature yet [19].

### Pangenome development

The Orthofinder v.2.5.4 software [20], using DIAMOND v.2.0.12 [21], was employed for the analysis and identification of orthologous genes. This software was used in standard mode. *In-house* scripts were used for the classification of genes into core (conserved genes that are present in all analyzed genomes and play essential roles in cellular processes), shared (genes found in two or more lineages or species, but not universally present in all of them), and singletons (species- or lineage-specific genes), as well as for the calculation of pangenome development.

Species with four or more available genomes were filtered to enable pangenome analysis, since the formulas applied to calculate the core genome and singletons depend on three variables, requiring at least three points on the curve, in addition to excluding the first genome to avoid bias in the results. Thus, it was not possible to perform pangenome analysis for the 31 initially selected species, but only for ten subsequently chosen ones, as many of them had fewer than four genomes available in the NCBI database. The selected species were *R. amblyommatis*,* R. bellii*,* R. conorii*,* R. japonica*,* R. parkeri*,* R. prowazekii*,* R. rhipicephali*,* R. rickettsii*,* R. sibirica*, and *R. typhi*. It is worth noting that *Rickettsia sp*. genomes were not included, as these isolates have not yet been taxonomically classified at the species level.

The pangenome development was calculated by applying Heap’s Law and the least-squares correction of exponential decay regression, following the standard pattern established by the *in-house* scripts used in this study, which performed 20 permutations with each genome occupying every possible position during the analysis. Furthermore, the *in-house* scripts employed in this work were developed by our research group solely as a tool to automate the application of methodologies already well established in the literature.

Heap’s Law is represented by the equation n = k*N^(−α)^, while the least-squares correction of exponential decay regression follows the model n = k*exp(-x/τ) + tg(θ). In these equations, *n* represents the number of genes observed for a given number of genomes; *N* is the total number of genomes considered in the pangenome extrapolation; *x* represents the number of genomes analyzed for core genome and singleton calculations; *exp* refers to Euler’s number; and *k*, *α*, *τ*, and *tg(θ)* are constants adjusted to best fit the behavior of each curve.

According to Heap’s Law, a value of α < 1 indicates an open pangenome, meaning that the total number of genes continues to increase as new genomes are added. Conversely, α > 1 characterizes a closed pangenome, where the inclusion of additional genomes does not significantly alter the total number of genes [22].

In the least-squares exponential decay regression, the term tg(θ) provides a more predictive insight: in core genome analysis, it represents the estimated number of genes that remain constant even after multiple genomes are added, while in singleton analysis, it corresponds to the approximate average number of new genes expected to be found per additional sequenced genome [23].

Finally, the genes identified were functionally classified using the COGclassifier program [24], after adapting the files generated by Orthofinder with the aid of *in-house* scripts.

### Comparative genomics

The Gegenees v.2.2.1 tool was used to calculate similarity through all-against-all genome comparison [25] for the ten previously filtered species, and the ANIclustermap v.2.0.1 software (https://github.com/moshi4/ANIclustermap) was employed to confirm genome similarity using ANI (Average Nucleotide Identity) indices for all 165 genomes, allowing assessment of the genetic relatedness among samples [26]. The program Gegenees applies a methodology based on genomic fragment alignment, using a fragment size of 200 and a step size of 100, enabling the simultaneous comparison of multiple genomes. These software was used in standard mode.

To improve the delimitation of genomic clusters and increase the reliability of species differentiation, an outgroup was included using the genome of *Orientia tsutsugamushi* (strain UT76, NCBI Taxonomy ID 682184) to verify the consistency of clustering and reinforce the genomic distinction observed between groups with higher and lower alpha values. Thus, *O. tsutsugamushi* was selected as the outgroup in the comparative analysis due to its close phylogenetic relationship to the genus *Rickettsia*, while still possessing a sufficiently distinct genome, as highlighted in the study by Gillespie and Salje [27].

The Mauve v.2.4.0 software [28] was used to perform gene synteny analyses, applying multiple genome alignments to investigate potential genomic rearrangements and identify possible events of genomic plasticity that may be associated with species evolution [29]. This software was used in standard mode.

### Philogenetic analysis

Phylogenetic inference was conducted using all genomes considered in the study. Orthologous groups were assigned across the proteomes using OrthoFinder v.2.5.4 [20], with sequence similarity searches performed through the DIAMOND v.2.0.12 algorithm [21]. The resulting orthogroups were subsequently aligned using MAFFT [30], applying the -M msa option, inside OrthoFinder pipeline. The phylogenetic tree was then reconstructed under the maximum likelihood criterion using IQ-TREE 2 [31], with 1.000 bootstrap replicates and all remaining parameters set to their default values. Tree visualization was performed using iTOL v7 [32] (https://itol.embl.de/).

### Statistical analysis

Statistical analyses were performed to evaluate the correlation of the α values and to generate the pangenome, core and singleton development curves using Microsoft Excel^®^ for Windows and GraphPad Prism^®^, version 8.0 (GraphPad, Inc., La Jolla, CA, USA). This software was used in standard mode. Correlations between variables were evaluated using Pearson’s correlation test. A significance level of 5% (*p* < 0.05) was adopted for all analyses.

### Identification of virulence and resistance genes

The ABRicate software (https://github.com/tseemann/abricate) was used to identify genetic determinants associated with antimicrobial resistance, and virulence factors. Thus the screening for antimicrobial resistance and virulence-associated genes was performed against the databases NCBI [33], CARD [34], ARG-ANNOT [35], ResFinder [36], EcOH [37], MEGARes [38], and VFDB [39] databases using Abricate. Genome sequences in FASTA (.fna) format were used as input, and only hits exhibiting sequence identity values greater than 95% were retained for downstream analyses [40].

### Identification of mobile elements

For the specific detection of possible plasmid sequences, the analyses were performed using the PlasmidFinder database [41] on ABRicate software (https://github.com/tseemann/abricate). Whole-genome nucleotide sequences in FASTA (.fna) format were used as input, and only matches showing sequence identity above 95% were considered for subsequent analyses [40].

For the identification of mobile genetic elements (MGEs), analyses were performed using MEFinder software v1.1.2 (https://pypi.org/project/MobileElementFinder/). Whole-genome nucleotide sequences in FASTA (.fna) format were used as input, and the prediction of MGEs was conducted based on sequence similarity searches against the integrated mobile genetic element database with 0.9 of coverage. Default parameters were applied throughout the analyses. For each MGE region identified, the corresponding protein products were retrieved from the NCBI Nucleotide database (https://www.ncbi.nlm.nih.gov/nuccore) based on their genomic coordinates and compiled into a spreadsheet, allowing the functional annotation of the genes associated with each mobile element.

## Results

### *Rickettsia *genus

#### Pangenome development

OrthoFinder classified the genomes into three distinct categories: core genome, shared genome, and singletons to identify orthologous genes.

The results obtained from OrthoFinder were filtered according to the species that had more than four representative genomes. With the ten selected *Rickettsia* species (Table 2), it was possible to compile a table showing the core genome values for each species as a percentage of the mean number of genes they contained.

**Table 2:** Results of orthologous identification and analysis

Species	α	Core	Singletons	Gene Average	Core (%)	Singletons (%)
*R. typhi*	0,999	788	0	808,50	97%	0%
*R. prowazekii*	0,994	722	1	824,83	88%	0,12%
*R. japonica*	0,986	1231	4	2608,46	47%	0,15%
*R. rickettsii*	0,977	1154	36	1327,46	87%	2,71%
*R. sibirica*	0,969	1142	2	1324,45	86%	0,15%
*R. parkeri*	0,96	1209	80	1353,40	89%	5,91%
*R. amblyommatis*	0,956	1036	10	1369,00	76%	0,73%
*R. bellii*	0,929	1198	39	1437,40	83%	2,71%
*R. conorii*	0,904	785	132	1312,94	60%	10,05%
*R. rhipicephali*	0,876	989	102	1330,75	74%	7,66%

Source: By author, 2025. Note: The table presents the selected species and the results obtained after orthologous gene identification, showing α values, core genes, singletons, average gene counts, and the corresponding percentages.

The findings revealed core genome percentages ranging from 47% to 97% and α values ranging from 0.876 to 0.999. Additionally, the number of genes in the core genome varied from 722 to 1231, and singletons ranged from 0 to 132, as shown below.

**Fig. 1:**
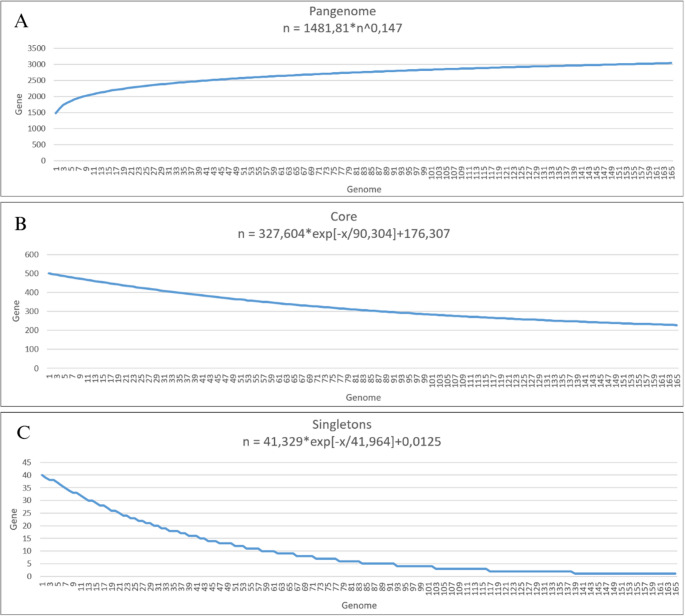
*Rickettsia* genus. Source: By author, 2025. Note: Graphs illustrating the dynamics of the pangenome (**A**), core genome (**B**), and singletons (**C**) among the analyzed *Rickettsia* genomes. The adjustment equations shown in each graph represent the regression models used to describe the trends

Based on these results, pangenome, core, and singleton development curves were generated in Microsoft Excel^®^. Figure 1 presents the developmental pangenome, core genome, and singleton curves for the *Rickettsia* genus.

In Fig. 1A, the pangenome curve shows an upward trend for the genus, based on the 165 analyzed strains, ranging from 1482 to 3040 genes. The α value is obtained using the formula α = 1-γ, where the γ value for the genus is derived from the equation *n* = 1481,81*n^0,147^. With γ = 0,147, the corresponding α value is 0,853, indicating an open pangenome.

In Fig. 1B, the core genome curve follows the model *n* = 327,604*exp[-x/90,304] + 176,307, with a decreasing gene range from 500 to 228. In Fig. 1C, the decreasing singleton curve ranges from 40 to 1 gene, modeled by *n* = 41,329*exp[-x/41,964] + 0,0125.

#### Characterization of orthologous genes

**Fig. 2:**
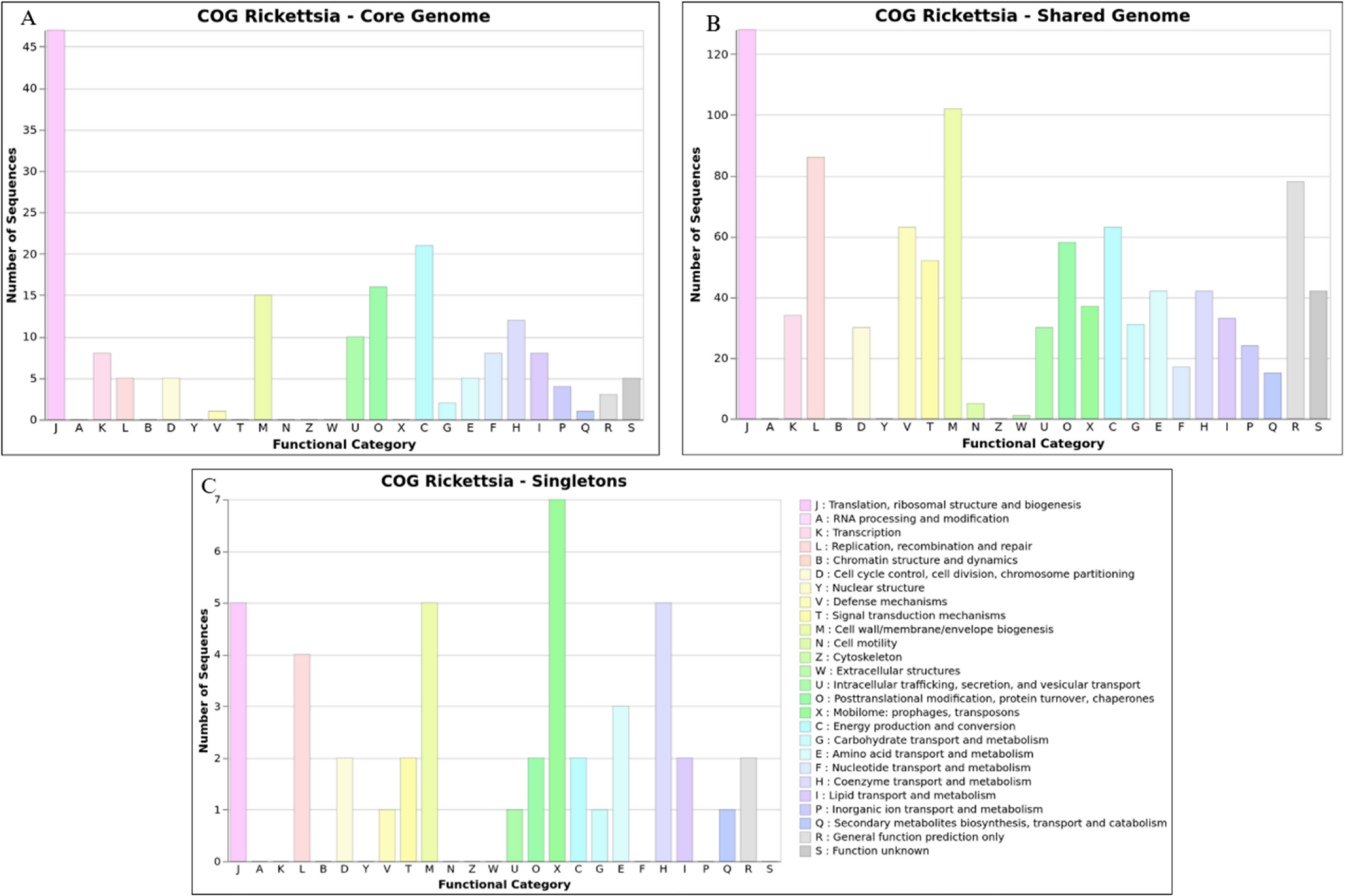
Characterization of orthologous genes in the *Rickettsia* genus. Source: By author, 2025. Note: Functional distribution of orthologous genes according to COG categories in the core genome (**A**), shared genome (**B**), and exclusive genes (**C**). The bars represent the number of sequences assigned to each COG functional category, according to the legend shown next to each graph

Figure 2 presents the results of the orthologous gene analysis for the *Rickettsia* genus. The analyses were performed using all species, and COG functional classification was assigned to the core genome, the shared genome, and the singletons, respectively. Differences among the functional categories of these gene sets are evident. In the core genome (2 A), category COG J predominates, with 47 genes associated with translation, ribosomal components, and biogenesis. In the shared genome (2B), COG J also predominates, with 128 genes, followed by COG M with 102 genes related to cell wall and membrane structures, and COG L with 86 genes involved in DNA replication and repair. For the singletons (2 C), there is a predominance of genes in COG X, with 7 genes associated with gene transposition and others not yet categorized, displaying a dispersed and varied distribution across categories.

#### Taxonomic analysis

**Fig. 3:**
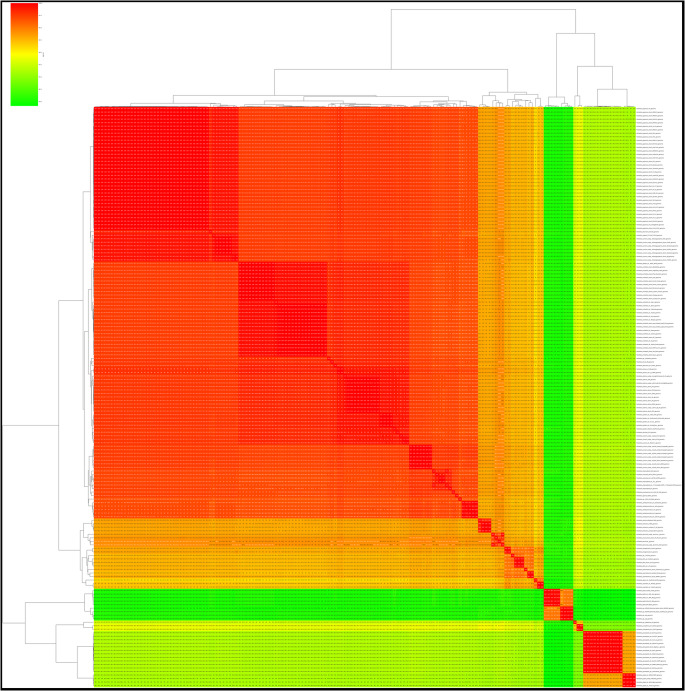
ANIclustermap for the *Rickettsia* genus. Source: By author, 2025. Note: Heatmap and dendrograms generated by ANIclustermap, showing average nucleotide identity values ranging from red (high similarity) to green (low similarity). The dendrogram displays the hierarchical clustering of the strains based on genomic relatedness

The taxonomic analysis of the 165 genomes in this study was performed using the ANIclustermap tool, and the results are shown in Fig. 3. The similarity confirmation analysis based on Average Nucleotide Identity (ANI) indices is displayed through a clustermap, heatmap, and dendrograms, with a color scale ranging from green (low identity) to red (high identity). Clear clusters of high similarity appear as intense red blocks along the main diagonal, indicating that genomes within each group share ANI values above 95%, a widely accepted threshold for species delineation. In contrast, green or yellow regions indicate low nucleotide identity between certain groups.

The resulting dendrogram reveals a clear phylogenomic structure, with well-defined clusters based on genomic proximity. 

### Comparison between *R.typhi* and *R.rhipicephali*

#### Pangenome development

**Fig. 4:**
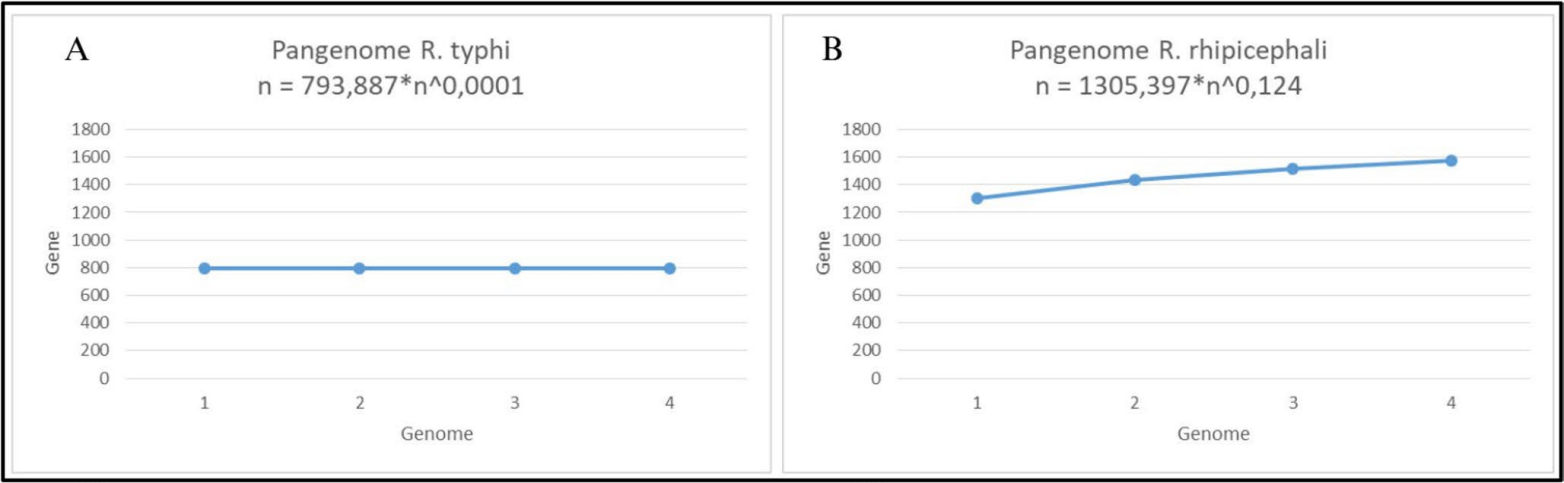
Pangenome of *R. typhi* and *R. rhipicephali. *Source: By author, 2025. Note: Graphs representing pangenome expansion in R. typhi (**A**) and R. rhipicephali (**B**), based on the cumulative number of genes identified as additional genomes are incorporated. The equations shown correspond to the regression models used to describe the observed variation

**Fig. 5:**
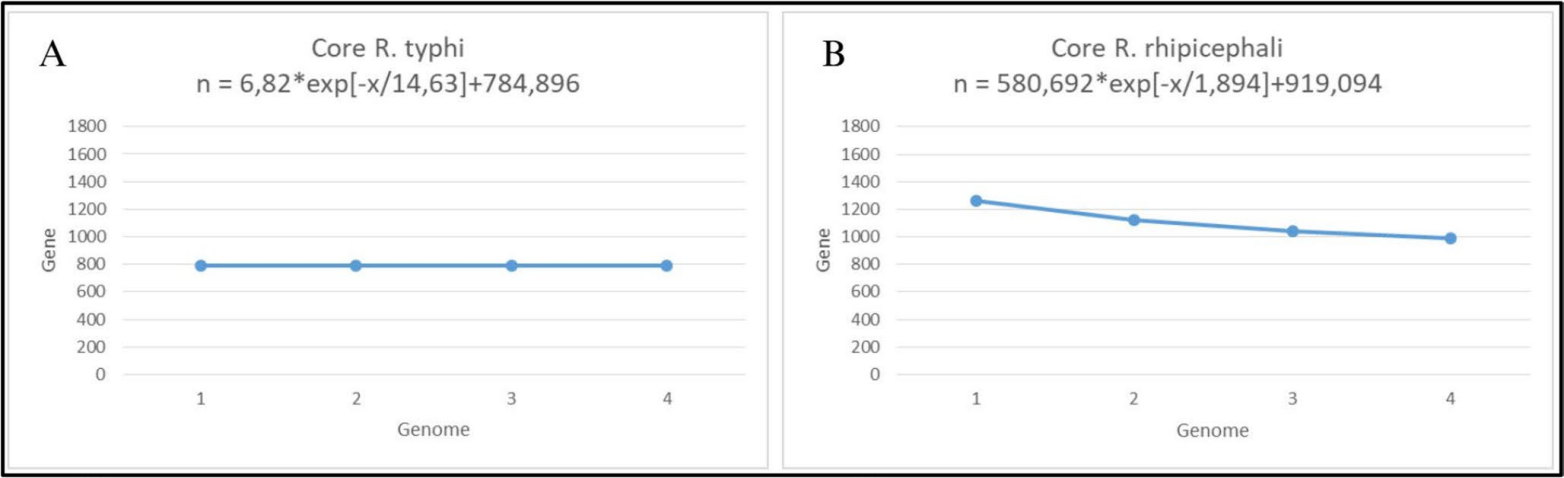
Core genome of *R. typhi* and *R. rhipicephali. *Source: By author, 2025. Note: Graphs illustrating the evolution of the core genome in R. typhi (**A**) and R. rhipicephali (**B**) as new genomes are incorporated. The equations shown correspond to the regression models applied to represent the observed variation

**Fig. 6:**
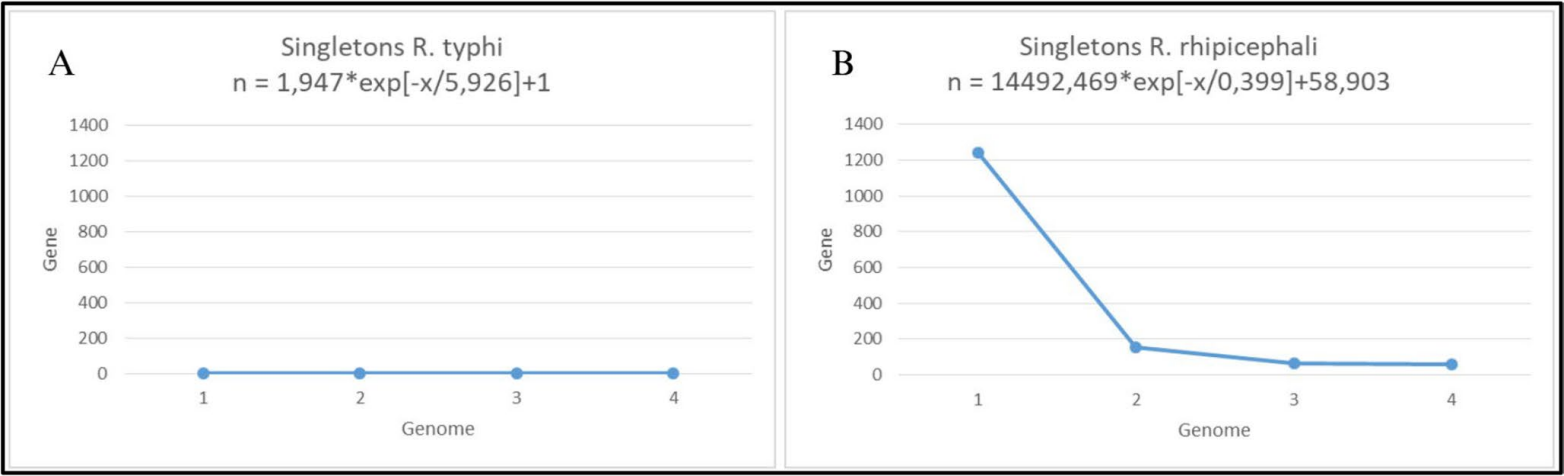
Singletons genes in the genomes of *R. typhi* and *R. rhipicephali. *Source: By author, 2025. Note: Graphs showing the variation in the number of unique genes (singletons) in R. typhi (**A**) and R. rhipicephali (**B**) as additional genomes are analyzed. Exponential regression models were used to generate the curves, with their respective equations shown

Figures 4 and 5, and 6 show, respectively, the pangenomes of the species with the highest alpha value (*R. typhi*) and the lowest alpha value (*R. rhipicephali*), along with the core genomes and singletons of each.

Figure 4A refers to the pangenome of *R. typhi*, modeled by the equation *n* = 793,887*n^0,0001^, where 0.0001 corresponds to the γ value, resulting in 794 genes across all lineages. Figure 4B shows the pangenome of *R. rhipicephali*, modeled by *n* = 1305,397*n^0,124^, with γ = 0.124 and gene counts ranging from 1305 to 1576. Accordingly, the α values obtained were 0.999 for *R. typhi* and 0.876 for *R. rhipicephali*, indicating an almost closed pangenome (α ≈ 1) for the former and a more open pangenome (α < 1) for the latter.

Figure 5A presents the behavior of the core genome of *R. typhi*, modeled by the equation *n* = 6,82*exp[-x/14,63] + 784,896 with variation between 791 and 789 genes. Figure 5B shows the core genome of *R. rhipicephali*, modeled by *n* = 580,692*exp⁡[-x/1,894] + 919,094, with a marked variation in gene numbers, from approximately 1262 down to 989 as more genomes are included.

Figure 6A shows the behavior of singleton genes in *R. typhi*, modeled by the exponential regression *n* = 1,947*e[-x/5,926] + 1. The resulting curve trends toward stabilization at approximately one single gene, regardless of the number of genomes analyzed. In contrast, Fig. 6B shows the singletons of *R. rhipicephali*, modeled by *n* = 14,492,469*e[-x/0,399] + 58,903. Here, a sharp decrease is observed as more genomes are added, starting from 1.242 singletons in the first genome and dropping to around 60 genes by the fourth genome.

The pangenome, core genome, and singleton curves for the remaining eight *Rickettsia* species are presented in SUPPLEMENTARY MATERIAL B and correspond to *Rickettsia amblyommatis* (B1), *R. bellii* (B2), *R. conorii* (B3), *R. japonica* (B4), *R. parkeri* (B5), *R. prowazekii* (B6), *R. rickettsii* (B7), and *R. sibirica* (B8).

#### Characterization of orthologous genes

**Fig. 7:**
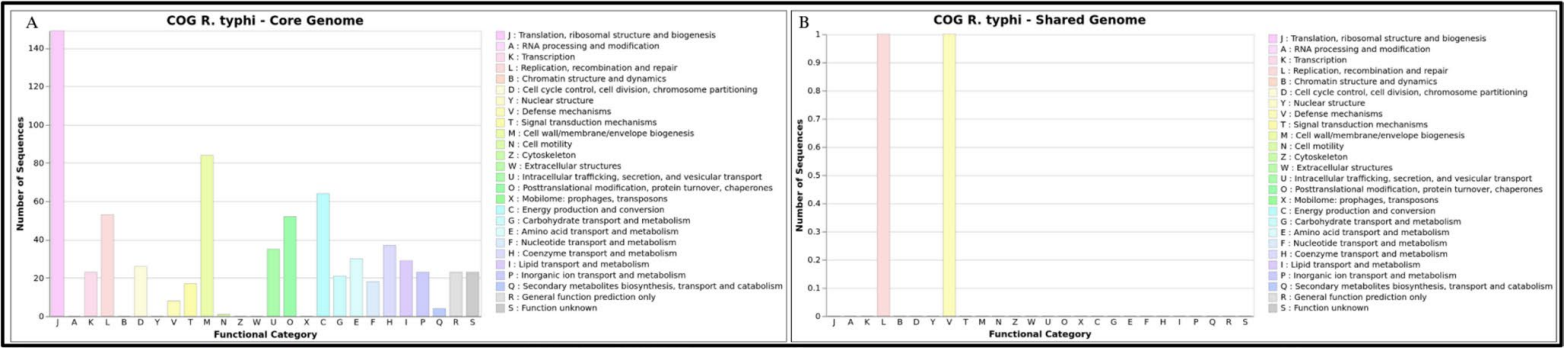
Characterization of orthologous genes of *R. typhi. *Source: By author, 2025. Note: Functional distribution of orthologous genes according to COG categories within the core genome (**A**) and shared genome (**B**). Bars represent the number of sequences assigned to each functional COG category for R. typhi, as indicated in the legend beside each graph

Figure 7 presents the data referring to the classification of the genes of *R. typhi*, the species with the highest alpha value identified in previous analyses. Gene classification was assigned to the core genome and shared genome, as the species did not present any singletons for COG classification. In the core genome (7 A), category COG J predominated, with 149 genes involved in translation, ribosomal components, and biogenesis. In the shared genome (7B), only two classifications were observed: COG L, with one gene involved in DNA replication and repair, and COG V, also represented by a single gene, associated with defense mechanisms.

**Fig. 8:**
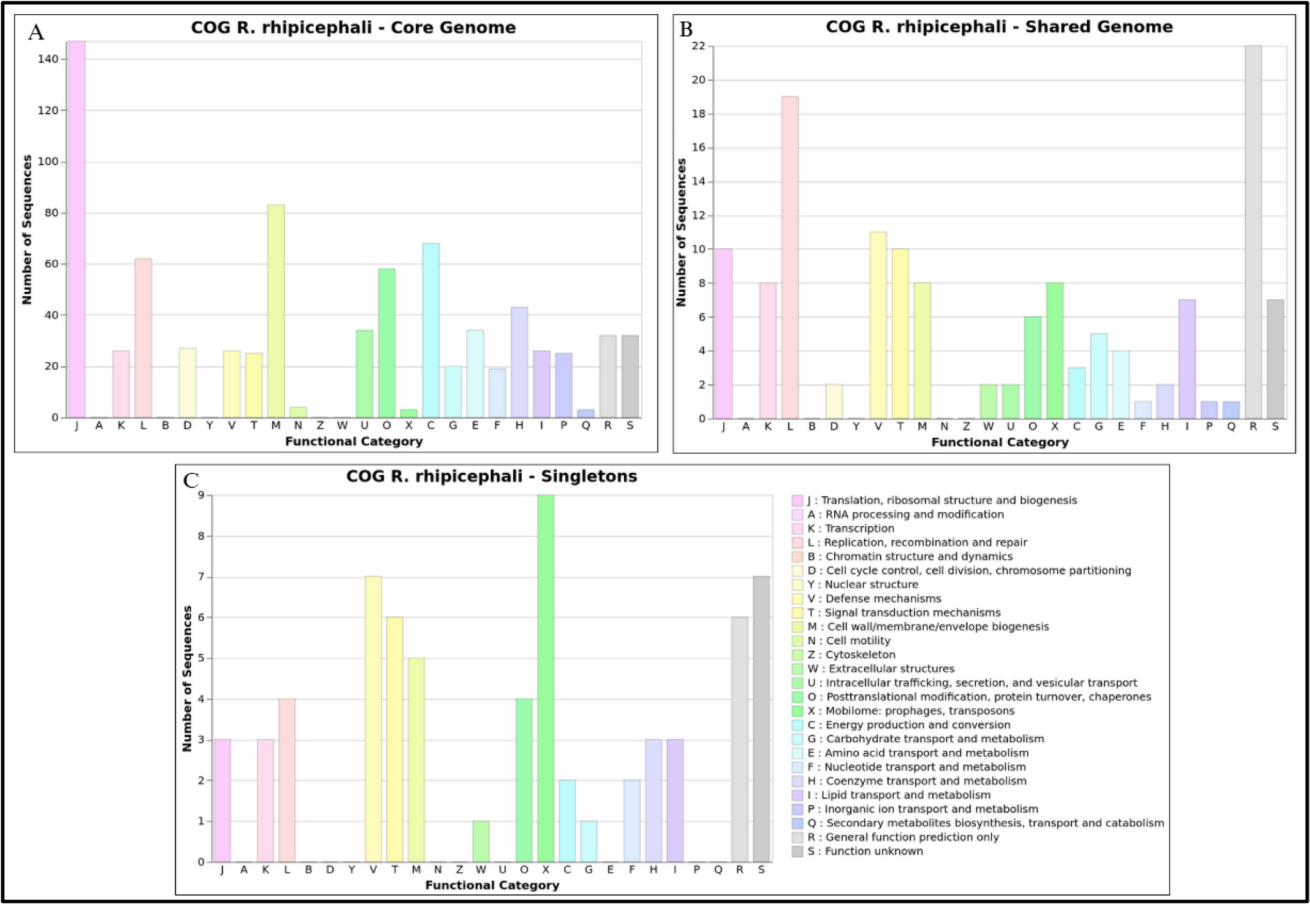
Characterization of orthologous genes of *R. rhipicephali. *Source: By author, 2025. Note: Functional distribution of orthologous genes according to COG categories within the core genome (**A**), shared genome (**B**), and exclusive genes (C). Bars represent the number of sequences assigned to each functional COG category for R. rhipicephali, as indicated in the legend beside each graph

The classification of orthologous genes for *R. rhipicephali*, the species with the lowest alpha value, is shown in Fig. 8, with COG functional categories assigned to the core genome, shared genome, and singletons, respectively. In the core genome (8 A), COG J predominates, associated with translation, ribosomal components, and biogenesis. In the shared genome (8B), COG R is the most abundant, containing 22 genes with only general functional prediction, followed by COG L, with 19 genes related to DNA replication and repair. For the singletons (8 C), there is a predominance of nine genes associated with gene transposition (COG X), seven genes not yet categorized (COG S), and another seven linked to defense mechanisms (COG V), showing a less dispersed but more varied distribution compared to the shared genome.

The figures corresponding to the characterization of orthologous genes for the other eight species of the genus *Rickettsia* are presented in SUPPLEMENTARY MATERIAL C and refer to the species *Rickettsia amblyommatis* (C1), *R. bellii* (C2), *R. conorii* (C3), *R. japonica* (C4), *R. parkeri* (C5), *R. prowazekii* (C6), *R. rickettsii* (C7), and *R. sibirica* (C8).

#### Gene synteny analyses

Gene synteny analyses were conducted using the *Rickettsia* species previously filtered to determine the degree of conservation of gene blocks. Synteny comparisons between genomes were performed using the Mauve software [29].

**Fig. 9:**
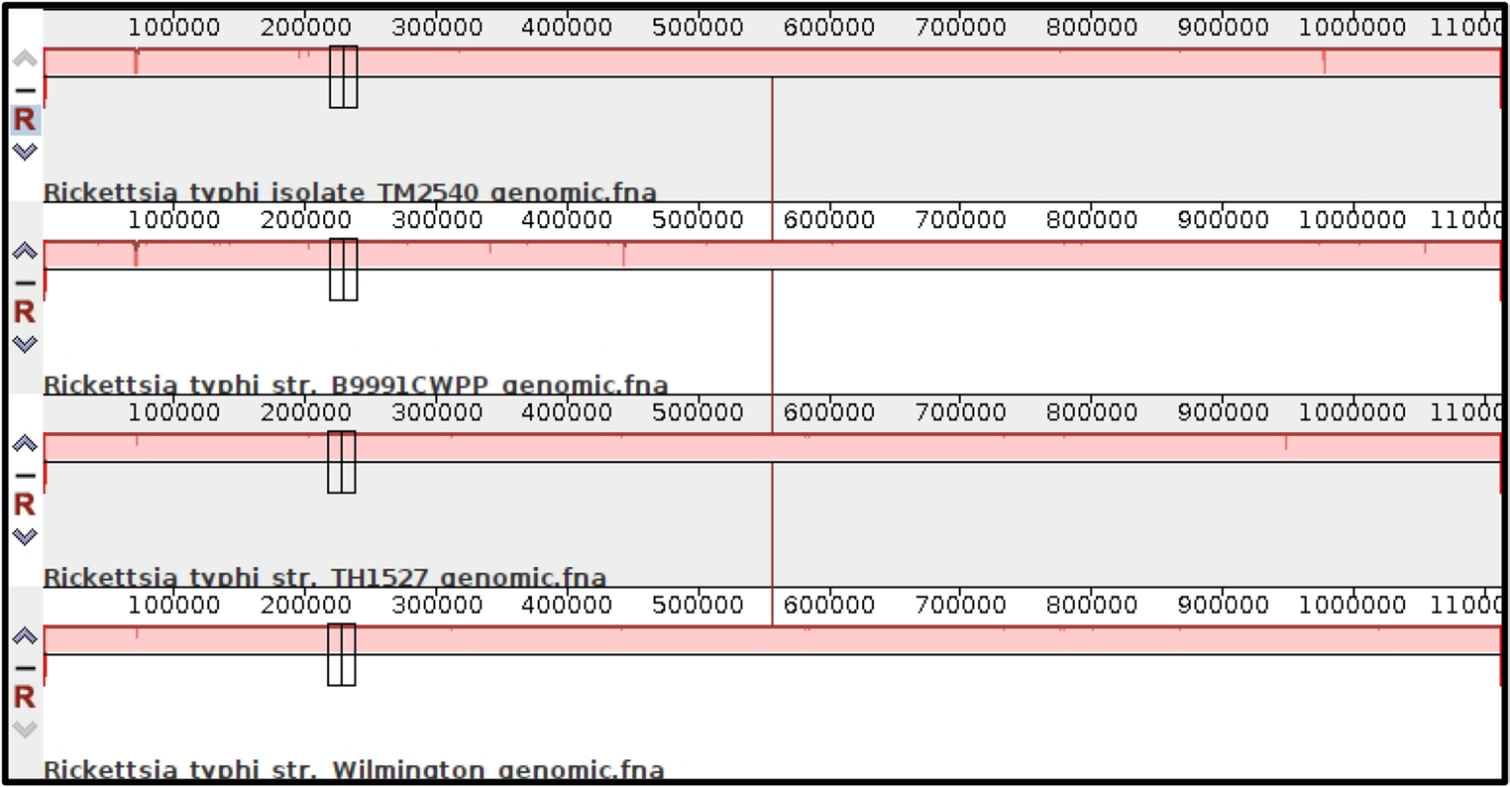
Mauve Alignment of *R. typhi. *Source: By author, 2025. Note: Genomic alignment among four genomes of R. typhi. The open bars observed in this figure represent software indicators associated with nucleotide residue positioning across samples, facilitating the visualization of potential translocated regions

With Mauve, it was possible to obtain an overview of genome conservation among species, highlighting *R. typhi* and *R. rhipicephali*. The software compared four genomes of *R. typhi*, which displayed a single aligned block in the same orientation in all genomes, with high similarity and conservation across the entire genome, as well as few visible inversions, deletions, or rearrangements, as shown in Fig. 9.

**Fig. 10:**
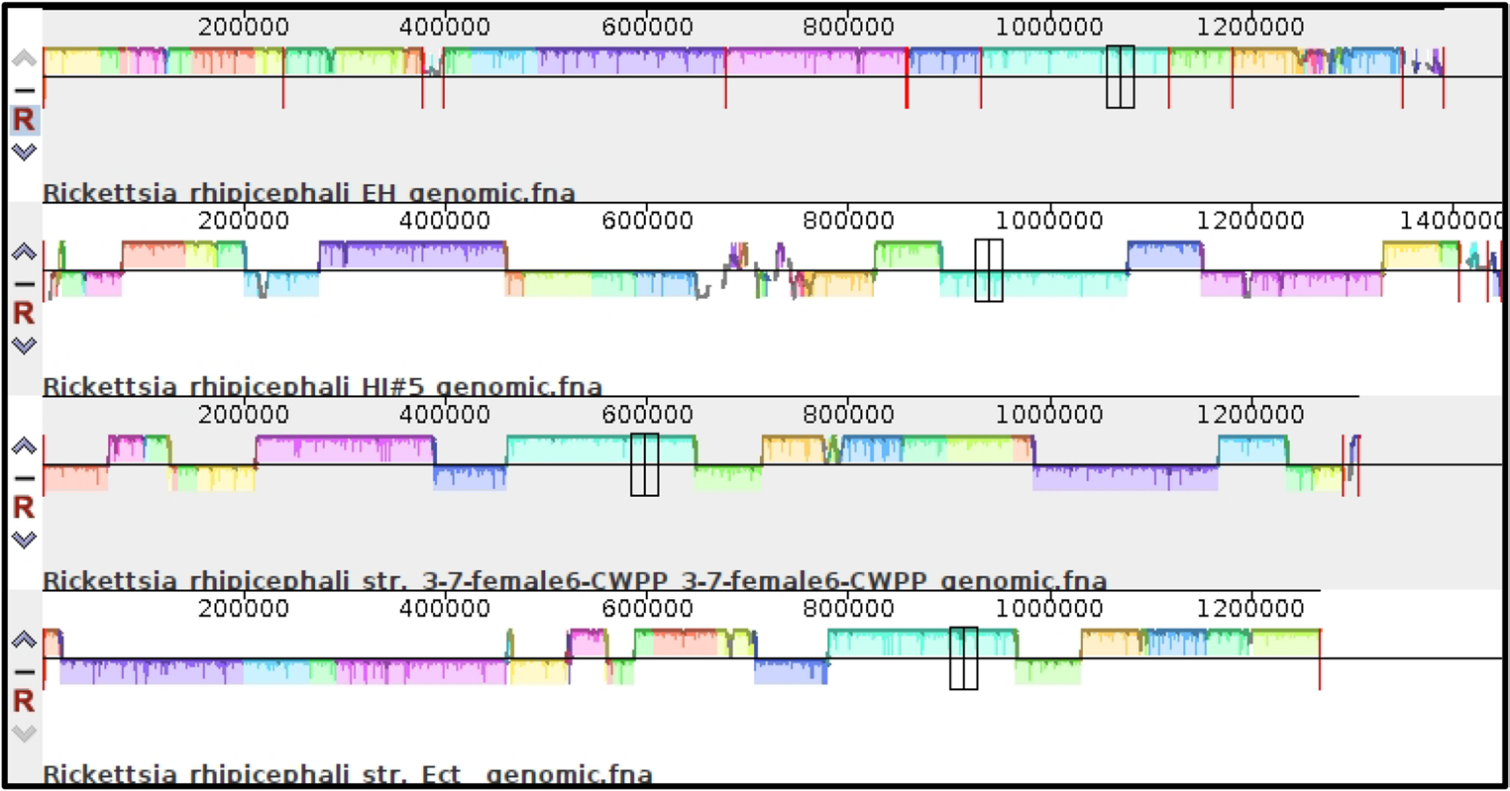
Mauve alignment of *R. rhipicephali. *Source: By author, 2025. Note: Genomic alignment among four genomes of R. rhipicephali. The open bars observed in this figure represent software indicators associated with nucleotide residue positioning across samples, facilitating the visualization of potential translocated regions

Regarding *R. rhipicephali*, the program also compared four genomes, which showed blocks of collinearity in different orientations, with inversions, rearrangements, and visible gaps (unaligned regions). Figure 10 shows regions present in some genomes but absent in others. Overall, despite the break in synteny, the blocks still exhibit high similarity.

The figures corresponding to gene synteny analyses for the other eight *Rickettsia* species are found in SUPPLEMENTARY MATERIAL D and refer to *Rickettsia amblyommatis* (D1), *R. bellii* (D2), *R. conorii* (D3), *R. japonica* (D4), *R. parkeri* (D5), *R. prowazekii* (D6), *R. rickettsii* (D7), and *R. sibirica* (D8).

#### Genomic similarity analyses

Comparative genomics analyses for *Rickettsia* species with more than four genomes were performed using the Gegenees software. Based on the genomes uploaded, a distance matrix represented as a heatmap was generated, and all results were exported in “.html” format.

**Fig. 11:**
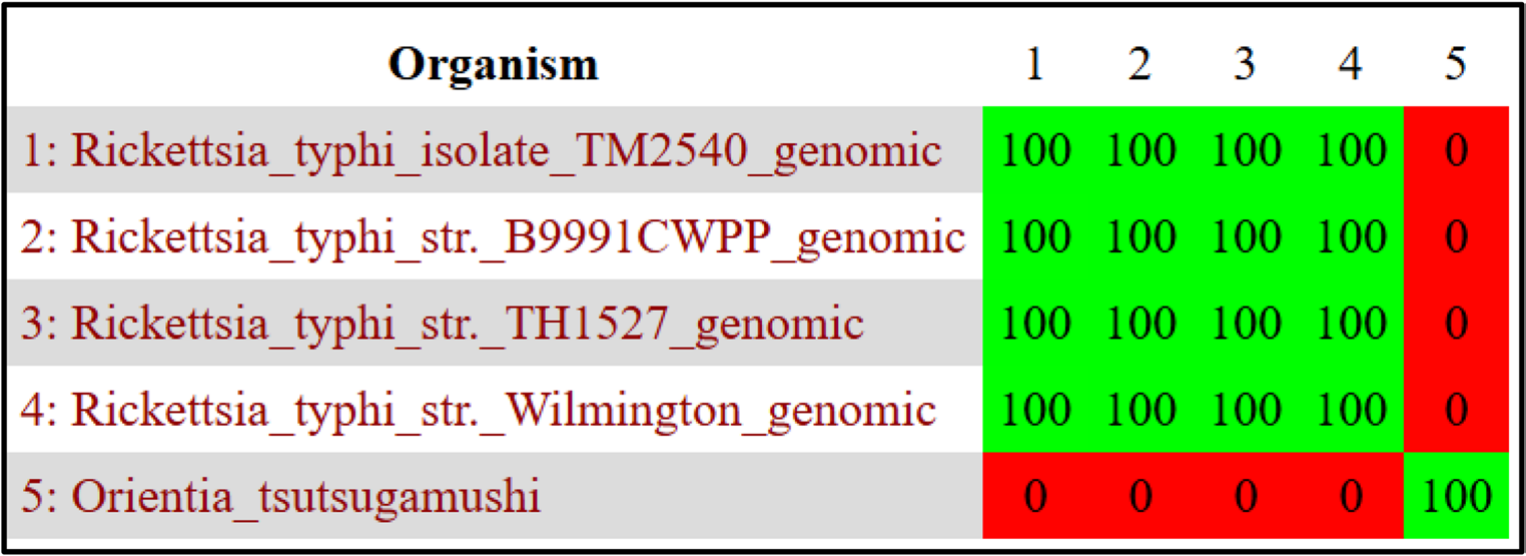
*Heatmap* of *R. typhi. *Source: By author, 2025. Note: Heatmap generated by Gegenees showing the similarity matrix of R. typhi

The analyzed genomes produced the heatmap shown in Fig. 11 for *R. typhi*, in which the four genomes of the species were compared to each other and to the outgroup *O. tsutsugamushi*. Similarity values among *R. typhi* genomes were all 100% or very close. Comparison with *O. tsutsugamushi* resulted in 0% similarity, confirming its appropriate selection as an outgroup.

**Fig. 12:**
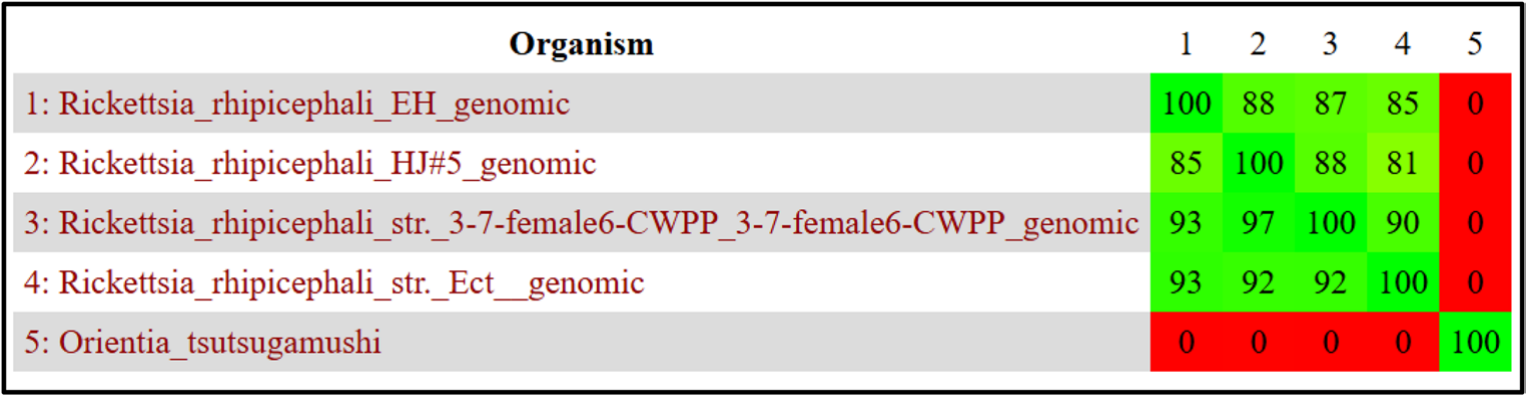
*Heatmap* of *R. rhipicephali. *Source: By author, 2025. Note: Heatmap generated by Gegenees showing the similarity matrix of R. rhipicephali.

In Fig. 12, the heatmap for *R. rhipicephali* shows similarity values ranging from 81% to 100% among the four genomes analyzed, while *O. tsutsugamushi* once again displays 0% similarity with all samples.

The figures corresponding to genomic similarity analyses of the other eight species of the genus *Rickettsia* are found in SUPPLEMENTARY MATERIAL E and refer to *Rickettsia amblyommatis* (E1), *R. bellii* (E2), *R. conorii* (E3), *R. japonica* (E4), *R. parkeri* (E5), *R. prowazekii* (E6), *R. rickettsii* (E7), and *R. sibirica* (E8).

### Philogenetic analysis

**Fig. 13:**
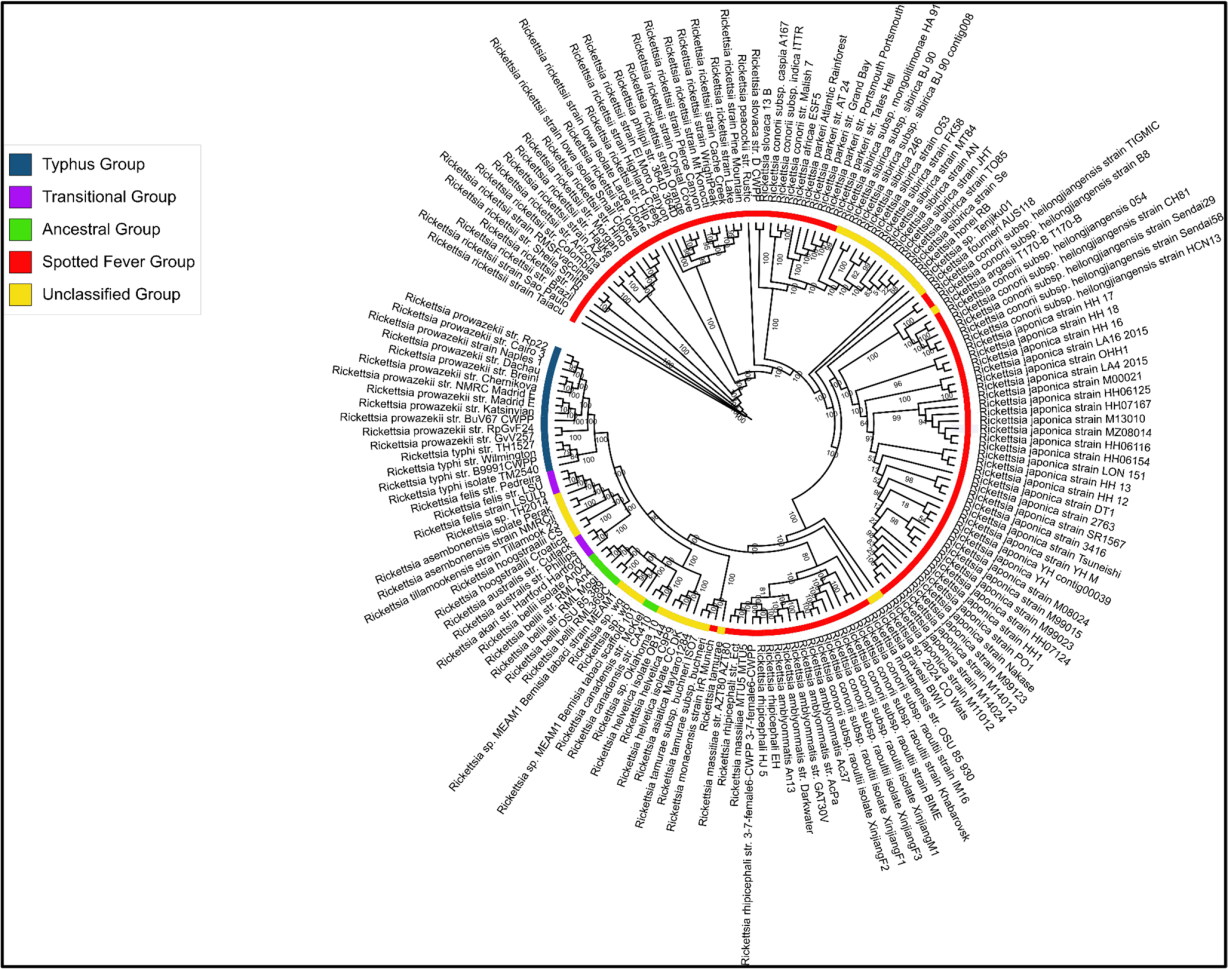
Phylogenomic tree of *Rickettsia* species. Source: By author, 2025. Note: Each tip of the phylogenetic tree represents an individual *Rickettsia* genome, colored according to its taxonomic classification group. Blue corresponds to the Typhus Group, purple to the Transitional Group, green to the Ancestral Group, red to the Spotted Fever Group, and yellow to genomes that remain Unclassified. Bootstrap values are indicated at the nodes and represent branch support

The phylogenomic analysis based on orthologous proteins identified among all analyzed *Rickettsia* genomes is presented in Fig. 13. The maximum-likelihood tree resolved the strains into the major phylogenetic groups currently recognized within the genus, including the Typhus Group (TG), Transitional Group, Ancestral Group, Spotted Fever Group (SFG), and Unclassified Group.

The Typhus Group comprised strains of *R. prowazekii* and *R. typhi*, which formed a clade with bootstrap values of 79 to 100. The Transitional Group included *R. akari*, *R. australis*, and *R. felis* with bootstrap values of 100. The Ancestral Group was composed primarily of *R. bellii* and *R. canadensis* strains, which clustered separately from the remaining lineages with bootstrap values ranging from 84 to 100.

The Spotted Fever Group represented the largest clade and included strains belonging to *R. africae*, *R. amblyommatis*, *R. conorii*, *R. japonica*, *R. massiliae*, *R. monacensis*, *R. parkeri*, *R. peacockii*, *R. philipii*, *R. rhipicephali*, *R. rickettsii*, and *R. slovaca* with bootstrap values ranging from 12 to 100. Within this group, *R. rhipicephali* strains formed a distinct cluster closely associated with *R. massiliae* with bootstrap values of 100. In addition, several species not assigned to the major classical groups formed independent branches, including *R. asembonensis*, *R. hoogstraalii*, *R. helvetica*, *R. asiatica*, *R. tamurae*, *R. tillamookensis*, *R. sibirica*, *R. honei*, *R. fournieri*, *R. gravesii*, and multiple *Rickettsia* spp. isolates with bootstrap values ranging from 22 to 100. Overall, the tree topology was demonstrated a clear separation among the major evolutionary lineages.

### Data correlation

**Fig. 14:**
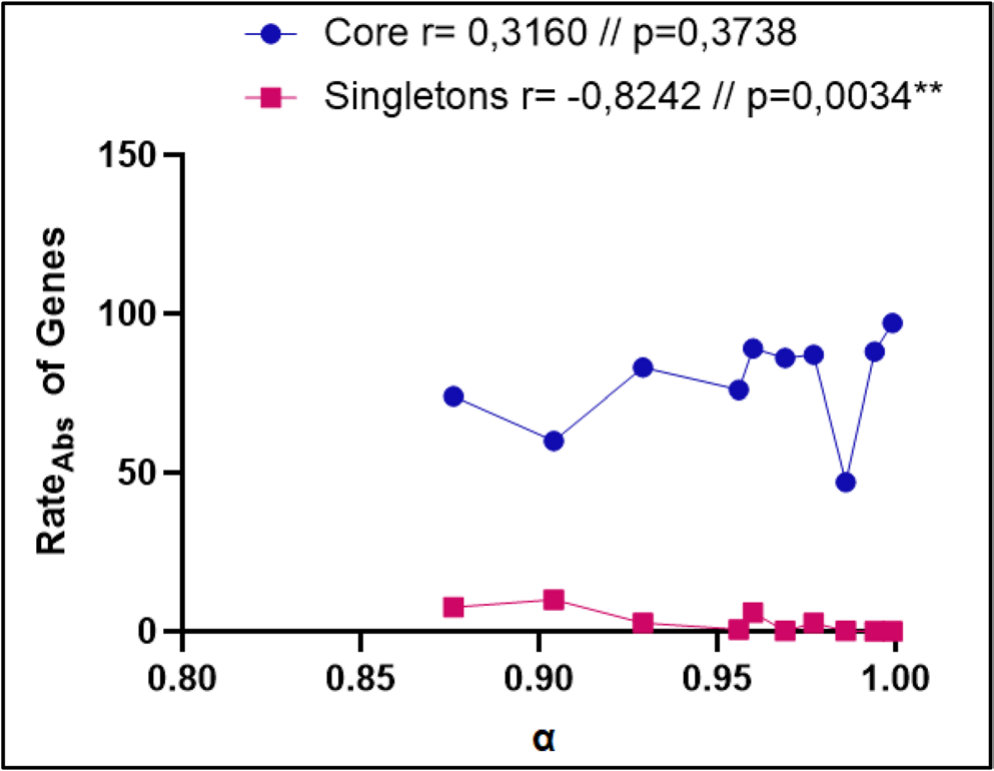
Correlation between α and the absolute rate of core genes and singletons. Source: By author, 2025. Note: The blue line represents core genes and the pink line represents singletons. Blue shows no significant correlation with α (*r* = 0,3160; *p* = 0,3738), whereas pink shows a significant negative correlation (*r* = −0,8242; *p* = 0,0034)

The statistical analysis performed using GraphPad Prism^®^ is shown in Fig. 14. Correlations between variables were evaluated using Pearson’s correlation test, as the data followed a normal distribution, with a significance level of 5% (*p* < 0.05).

Pearson’s correlation analysis assessed the influence of alpha (α) values on the absolute frequency of core genes and singletons, revealing no significant association between core gene counts and α (*r* = 0,3169, *p* = 0,3738), indicating stable values despite variations in α. Conversely, there was a strong and statistically significant negative correlation between singletons and α (*r* = −0,8242, *p* = 0,0034), suggesting that the number of singletons decreases as α increases.

### Identification of virulence and resistance genes

Screening against the NCBI AMRFinderPlus, EcOH, CARD, ARG-ANNOT, ResFinder, and MEGARes databases did not reveal any matches that satisfied the predefined threshold of 95% sequence identity. Conversely, putative virulence-associated genes were identified through searches against the VFDB database.

To ensure high-confidence predictions, only hits exhibiting at least 95% sequence identity and 95% coverage were retained for further analysis. Under these stringent criteria, the virulence-associated genes *ompA* and *rickA* were detected among the analyzed genomes. Both genes exhibited high sequence conservation among the analyzed genomes, with *ompA* predominantly identified in *Rickettsia rickettsii* strains and *rickA* broadly distributed across the species, *R. conorii*,* R. parkeri*,* R. philipii*,* R. rickettsii*,* R. sibirica*, and *R. slovaca*, strains, with identity values ranging from 95.20% to 100% and coverage ranging from 98.46% to 100%. The highest identity value for *rickA* (100%) was observed in *R. conorii* str. Malish 7, while the lowest (95.20%) was found in *R. slovaca* strains 13-B and D-CWPP. Detailed information on the distribution of genes, sequence identity, and coverage of these genes is provided in SUPPLEMENTARY MATERIAL F - Table 4.

### Identification of mobile genetic elements

No plasmid-associated sequences were detected in the analyzed genomes following screening against the PlasmidFinder database. Applying a minimum threshold of 95% sequence identity, no matches met the criteria for plasmid identification.

MEFinder identified mobile genetic elements associated with the insertion sequence ISRpe1 (IS481 family) in two species, *Rickettsia bellii* and *Rickettsia rhipicephali*, with a marked asymmetry in their distribution. In *R. bellii* strain An04, three ISRpe1-associated regions were detected on contig NZ_CP015011.1, comprising one composite transposon and two isolated insertion sequence copies, all with an identity of 0.940 to the reference element. In *R. bellii* strain RML Mogi, 16 ISRpe1-associated regions were identified on contig NZ_LAOJ01000001.1, comprising 14 composite transposons and 2 isolated insertion sequences, with identity values ranging from 0.945 to 0.949. In contrast, only a single isolated ISRpe1 copy was detected in *R. rhipicephali* strain HJ#5, on contig NZ_CP013133.1, with an identity of 0.940.

The composite transposons identified in *R. bellii* RML Mogi varied considerably in size, ranging from small cassettes carrying 3–5 accessory genes to large structures encompassing more than 30 open reading frames. Their gene content included functions related to translation and ribosomal biogenesis (e.g., elongation factors, ribosomal proteins, aminoacyl-tRNA ligases), DNA replication and repair (e.g., DNA polymerase III subunits, RecF, RecR), membrane transport (MFS and ABC transporter families), cell division (FtsA, FtsQ, ZapA, ZapE), and energy metabolism (NADH-quinone oxidoreductase subunits, F0F1 ATP synthase subunits, succinate dehydrogenase complex). Several composite transposons also carried additional transposase-related sequences, including IS110, IS256, and Rpn family recombination-promoting nuclease/putative transposase genes, as well as type II toxin-antitoxin system components (VapC family toxin, Phd/YefM antitoxin), suggesting nested mobile element insertions and potential mechanisms for element maintenance.

Notably, the composite transposon cn_39977_ISRpe1 carried genes encoding a VirB4 family type IV secretion/conjugal transfer ATPase and TrbL/VirB6 family proteins, components typically associated with conjugative elements. Additionally, the IS481 family transposase protein identifier WP_057700078.1 was detected in both *R. bellii* strain An04 and *R. rhipicephali* strain HJ#5, despite the lower identity values observed in these strains (0.940) compared to RML Mogi.

The full set of MGE-associated annotations identified across all analyzed species is provided in SUPPLEMENTARY MATERIAL G - Table 5 and refer to *Rickettsia argasii*,* Rickettsia bellii*,* Rickettsia felis*,* Rickettsia fournieri*,* Rickettsia gravesii*,* Rickettsia massiliae*,* Rickettsia monacensis*,* Rickettsia peacockii*,* Rickettsia rhipicephali*, and *Rickettsia tamurae.*

## Discussion

The average nucleotide identity (ANI) analysis presented in Fig. 3 reveals the degree of genomic similarity among the different strains and species of *Rickettsia* evaluated. A clear separation can be observed between groups of genomes with high identity, above 95%, which corresponds to approximately 70% of in vitro DNA–DNA hybridization (DDH) [42, 43]‌, highlighted in dark red tones, and consistent with current genomic criteria for species definition [44–46]‌.

Among the most cohesive clusters, it is possible to identify groups representing intraspecific lineages, such as *R. typhi*, *R. prowazekii*, and *R. rickettsii*, whose genomes exhibit high similarity (> 98%). This suggests strong genomic conservation and low intraspecific diversity, especially when considering the α values found and described in Table 2.

Conversely, a distinct pattern is seen in species with an open pangenome, such as *R. rhipicephali* and *R. amblyommatis*, which show greater dispersion in ANI values among different lineages and the presence of regions with lighter colors (orange to green). These patterns reflect greater intraspecific genomic diversity, which may be associated with recombination processes, differential selective pressures across hosts and environments, or even lineages undergoing speciation. Such variation reinforces the presence of divergent lineages or distinct species within the analyzed dataset [47].

Furthermore, the dendrogram generated from the ANI matrix allows for the identification of robust phylogenomic relationships among groups, aligning with established taxonomic classifications, while also suggesting potential reclassifications for lineages with borderline ANI values (90–95%) compared to closely related species. This opens avenues for future, more in-depth phylogenetic and functional analyses [48].

The analyses used to generate the pangenome curves indicate that the genus exhibits an open pangenome, in which the addition of new genomes significantly increases the number of detected genes [49]. Studies by Weinert et al., [50] and Felice et al. [3] highlight that the genus *Rickettsia* displays considerable genetic variability across species and that sequencing of new species continues to reveal novel genes. These works also indicate a high adaptive potential to different hosts and environments.

These findings support the results obtained in this study, which point to an open pangenome characterized by continuous acquisition of new genes and the absence of consolidated genomic stability within the genus.

The phylogenomic tree presented in Fig. 13 highlighted the main evolutionary lineages currently recognized within the genus *Rickettsia*, corroborating previous taxonomic and genomic studies [19, 51]. The clear separation of Typhus, Transitional, Ancestral and Spotted Fever groups supports the robustness of the orthology-based approach used for phylogenetic construction and confirms the evolutionary relationships between the species analyzed.

Among the specifically analyzed species, *R. rhipicephali* stands out for having a more open pangenome (α = 0.876) compared to *R. typhi* (α = 0.999). Values closer to 1 indicate a closed pangenome, demonstrating that the latter, which has the highest alpha value found, is a representative group nearing pangenome closure, with few or no new genes added per sequenced genome. This difference in alpha values may reflect each species’ adaptive potential: the more open the pangenome, the greater the likelihood of acquiring new genes through horizontal transfer and, consequently, the greater the adaptive capacity. In contrast, species with higher alpha values show adaptation toward a specific host or environment [52].

The graphs presented in Figs. 4, 5 and 6 support these findings. Visual comparison between *R. typhi* and *R. rhipicephali* highlights differences in pangenome growth dynamics: *R. typhi* displays a more stable curve, whereas *R. rhipicephali* shows a rising curve. Regarding core genome stabilization, *R. typhi* appears stable, while *R. rhipicephali* displays a declining curve. The behavior of the singleton graph for *R. rhipicephali*, for instance, presents a θ (theta) value of approximately 60 genes, reinforcing the hypothesis of an expanding pangenome when compared to the value of 1 gene observed in *R. typhi*, which is closer to 0 and stable.

Regarding singletons, the substantial quantities observed in species such as *R. conorii* and *R. rhipicephali* indicate the presence of exclusive genes that may be associated with specific functions or recent acquisition of mobile genetic elements [53]. Such genes may play important roles in virulence, antibiotic resistance, or modulation of host immune responses, offering opportunities for future functional studies.

From a biological and evolutionary perspective, the data suggest that *R. typhi*, with a nearly closed pangenome, represents a more stable and conserved species, as can be seen in Fig. 13. This is consistent with the findings of Kato et al., [54], whose comparative analyses of complete genomes across different lineages of the species highlighted minimal variations, few single nucleotide polymorphisms (SNPs), and insertions/deletions (INDELs), indicating highly similar genomes.

In contrast, *R. rhipicephali*, with greater gene diversity, reflects a potentially more dynamic and opportunistic evolutionary trajectory, as shown in Fig. 13. These findings align with those of Zeringóta et al. [55], who reported that *R. rhipicephali* stands out as one of the most widely distributed species within the rickettsiae group and demonstrates the ability to infect hosts across various genera and regions worldwide.

The variation in core genome percentage observed in Table 2, ranging from 47% to 97%, indicates that different *Rickettsia* species share distinct levels of genomic similarity. Species such as *R. typhi* (97%) and *R. prowazekii* (88%) exhibited high genomic conservation, suggesting a more closed pangenome likely associated with greater ecological specialization and a more restricted niche. Notably, the high similarity observed in ANI analyses reinforces the genomic stability detected in core and pangenome analyses.

Functional categorization of orthologous genes of the genus using the COG (Clusters of Orthologous Groups) tool enabled comparative analysis of gene distribution across different pangenome compartments: core, shared, and singletons. This approach is essential for understanding not only genomic conservation but also the functional and adaptive potential of these bacteria. It is important to highlight that the COG classifies those unknown genes that have not been annotated and assigned to COGs belonging to category S (unknown function) and category R (general function prediction only) [56, 57].

Core genome analyses (Fig. 2A) of all examined genomes revealed a predominance of genes classified under categories related to translation, ribosomal structure, and biogenesis (Group J), as well as energy production and conversion (Group C). These essential functions indicate that core genes are highly conserved and associated with basic cellular processes vital to survival [58]. Such conservation is expected, as the core genome represents the genetic foundation shared by all species examined, reflecting strong selective pressures that preserved these genes over evolutionary time [59].

In the shared genome (Fig. 2B), the classification exhibited greater functional diversity. Although still present in more than one species, these genes represent mechanisms associated with Group J, but also Groups M and L, which are involved in cellular defense mechanisms, maintenance of cellular integrity, and DNA replication, recombination, and repair, respectively. Classifications within Group R, associated with general predicted functions, were also significant. This distribution suggests that these functional categories participate in the adaptive flexibility of the genus, being useful for adaptation to specific environmental conditions, host variation, or cellular stress [60].

Singletons (Fig. 2C) were concentrated within Groups L, R, and X, the latter being related to genetic mobility. The large number of genes in these categories is particularly relevant, as it suggests the presence of poorly understood genetic elements, possibly acquired through horizontal transfer or recent mutations. This reflects the evolutionary potential of the open pangenome in certain species and reinforces the findings from other analyses, such as those involving *R. rhipicephali* [61].

Functional analyses were further explored for *R. typhi* and *R. rhipicephali*, highlighting distinct trends. *R. typhi* exhibits a core genome highly concentrated in categories J and M, with few genes in other functional groups [58]. The shared genome is practically nonexistent, supporting its stability and low genomic variability, likely linked to ecological specialization and a restricted, stable niche, as shown in genus-level analyses. The absence of singletons also reinforces the stability of this species.

Instead, *R. rhipicephali* displays the opposite pattern: although the core genome still highlights Group J, the number of shared genes and singletons is significantly larger and distributed across various categories, particularly Groups R, L, and X. This suggests considerable genomic flexibility and indicates that *R. rhipicephali* may be undergoing adaptive diversification [61].

Synteny analyses for *R. typhi* and *R. rhipicephali* further illustrate the divergence between the two species. *R. typhi*, evolutionarily more stable and less plastic, displays high conservation of gene order among strains, with no visible inversions or breaks. *R. rhipicephali*, however, exhibits a fragmented genomic architecture marked by multiple inversions, interruptions, and potential insertion or deletion events.

Kato et al. [54] described that *R. typhi* exhibits minimal variation in comparative analyses, reporting only 26 SNPs and 7 INDELs across complete genome comparisons, findings that align with Mauve-based analyses in this study, which detected very few differences among the four lineages.

The diversity observed in *R. rhipicephali* may be linked to mobile genetic elements and greater exposure to horizontal gene transfer, characteristics associated with broader ecological diversity and genetic interactions, as observed in the pangenome curves and functional categorizations, as well as the literature by Zeringóta et al. [55].

The virulence-associated genes identified in this study, *ompA* and *rickA*, displayed distinct distribution patterns among the analyzed genomes, reflecting their different functional roles and evolutionary conservation within the genus. The gene ompA, encoding the Outer Membrane Protein A, in Gram-negative bacteria, is recognized as a key virulence-associated protein involved in multiple steps of the infectious process, acting as both an adhesin and an invasin, it enables bacterial attachment to host cells and promotes cellular uptake via receptor-mediated interactions [62]. This gene was exclusively detected among *R. rickettsii* strains, with sequence identity values ranging from 99.20% to 99.98% and 100% coverage across all positive strains. This high degree of conservation is consistent with the essential role of *OmpA* in host cell adhesion and invasion, as well as its widespread use as a diagnostic and phylogenetic marker for *R. rickettsii* [63].

In contrast, *rickA*, an activator of the host Arp2/3 complex which encodes a protein involved in actin-based intracellular motility [64], was detected across a broader range of Spotted Fever Group species, including *R. conorii*, *R. parkeri*, *R. philipii*, *R. rickettsii*, *R. sibirica*, and *R. slovaca*, with identity values ranging from 95.20% to 100%. This broader distribution suggests that actin-based motility is a conserved virulence strategy shared among the main pathogenic species of the Spotted Fever Group, potentially representing a functional trait maintained by shared selective pressures related to intracellular survival and cell-to-cell spread, because the capacity to utilize two distinct actin polymerization mechanisms may enable the genus to efficiently establish an intracellular environment and disseminate among different host cell types during persistent infection [64]. The variation in identity values observed for *rickA* across different species and strains, while remaining above the 95% threshold adopted in this study, may reflect the greater functional tolerance of this gene to sequence divergence compared to *ompA*, or alternatively, differential evolutionary pressures acting on each gene across distinct host and vector associations.

The identification of mobile genetic elements (MGEs) in *R. bellii* and *R. rhipicephali*, with a marked predominance in *R. bellii*, is consistent with the genomic plasticity observed in the pangenome analyses and supports the distinct evolutionary trajectories proposed for these species [65]. As a member of the Ancestral Group, *R. bellii* is one of the most genomically diverse species in the genus, harboring a larger repertoire of accessory genes and mobile DNA than most species in more derived clades [66]. The abundance of ISRpe1-associated composite transposons identified here reinforces this characteristic, suggesting that mobile elements have contributed to shaping its genomic architecture. These composite transposons carry genes involved in translation, DNA replication and repair, membrane transport, cell division, transcriptional regulation, and energy metabolism, indicating that transposition events may have driven the acquisition, redistribution, and maintenance of accessory genetic content throughout the species’ evolutionary history [65–67], consistent with the larger accessory genome observed for *R. bellii* in this study. The recurrent association of ISRpe1 with other mobility-related genes, including IS110 and IS256 transposases and Rpn family recombination-promoting nucleases, suggests mosaic structures generated through successive recombination and insertion events, a pattern associated with genome remodeling and adaptation in dynamic bacterial genomes [65].

Several composite transposons also carried type II toxin-antitoxin system components (VapC toxins, Phd/YefM antitoxins), which are commonly associated with mobile elements and thought to enhance their persistence within host genomes through post-segregational maintenance [68, 69]. Notably, the composite transposon cn_39977_ISRpe1 carries genes encoding a VirB4 ATPase and TrbL/VirB6 family proteins, key components of type IV secretion and conjugative transfer systems. This suggests the region may represent a remnant of an ancestral conjugative element or reflect past horizontal gene transfer events contributing to the diversification of *R. bellii* [68, 69]‌. While functional activity cannot be inferred from genomic data alone, the conservation of these components highlights the potential role of MGEs in mediating genetic exchange during the evolution of ancestral *Rickettsia* lineages.

In contrast, only a single ISRpe1 copy was detected in *R. rhipicephali* strain HJ#5, indicating a substantially reduced mobilome relative to *R. bellii* and suggesting that transposition-mediated remodeling played a minor role in this species’ evolution. Despite this limited MGE abundance, *R. rhipicephali* exhibits an open pangenome (α = 0.876), indicating that its genomic diversity is likely maintained through alternative mechanisms such as homologous recombination, gene gain and loss, and lineage-specific gene turnover rather than transposable element expansion [65].

Comparative genomics analyses produced a heatmap for *R. typhi*, in which the four genomes were compared among themselves and with *O. tsutsugamushi*. The values demonstrated extremely high conservation among *R. typhi* lineages, as noted by Kato et al. [54], and the comparison with *Orientia* resulted in 0% similarity, highlighting the extensive evolutionary distance between these groups and confirming its use as an appropriate outgroup [27]. The heatmap for *R. rhipicephali* displayed more variable similarity, showing good intraspecific conservation but more variation and less genomic cohesion than in *R. typhi* [61].

The species *R. japonica* and *R. conorii* exhibited intermediate alpha values and partial core genome conservation, indicating an evolutionary profile associated with both the maintenance of conserved genes and the capacity for genomic adaptation. In *R. japonica*, for example, the reduced genetic variability among different isolates suggests high genomic stability, possibly linked to specific ecological environments. In both species, the low genetic variability and preservation of essential genes indicate strong evolutionary conservation, potentially related to their association with specific vectors and hosts, as well as the maintenance of important adaptive mechanisms for ecological persistence [70, 71].

The absence of a significant correlation between core genome size and α (Fig. 13) reinforces the idea that essential, highly conserved genes of *Rickettsia* remain stable regardless of pangenome expansion, reflecting their importance for organism viability and core cellular functions [59].

In contrast, the strong and statistically significant negative correlation between the number of singletons and α indicates that the more open the pangenome, the higher the occurrence of unique genes. This aligns with the evolutionary model of open pangenomes, in which the addition of new genomes leads to the continuous incorporation of rare or unique genes, frequently associated with horizontal gene transfer, mobile genetic elements, and ecological adaptation [60]. Similar patterns have been reported in various bacterial genera with high genomic plasticity and broad environmental distribution [18, 72].

Thus, the data reinforce the role of singletons as indicators of diversity and adaptive capacity, while the core genome remains an evolutionarily conserved functional foundation [72].

## Conclusion

The analyses revealed substantial genomic heterogeneity among species of the genus *Rickettsia*, highlighting striking contrasts between highly conserved genomes and others that are more fragmented. A statistically significant negative correlation was observed between the α parameter and the number of singletons, contributing to the understanding of genomic plasticity driven by active gene acquisition mechanisms, such as horizontal transfer. Despite variations within the pangenome, the core genome exhibited remarkable stability, suggesting the preservation of an essential gene set subjected to intense selective pressure.

Thus, this work confirms the open nature of the pangenome of the genus *Rickettsia* and points to the existence of distinct evolutionary trajectories within the group, with direct implications for the ecology, adaptation, and possibly the pathogenicity of the species. These findings contribute to the understanding of the genomic dynamics of the genus and open new perspectives for phylogenomic, functional, and genomic surveillance studies of emerging pathogens.

In this way, the results provide insights into clonal patterns and the adaptive capacity of species within the genus, support the understanding of genomic evolution and survival strategies of these bacteria, and may be applied in future studies, such as protein–protein interaction (PPI) prediction and the development of databases focused on virulence factors and potential vaccine targets. 

## Supplementary Information

Below is the link to the electronic supplementary material.

Supplementary Material 1 (PNG 23.4 KB)

Supplementary Material 2 (PNG 21.2 KB)

Supplementary Material 3 (PNG 23.9 KB)

Supplementary Material 4 (PNG 20.5 KB)

Supplementary Material 5 (PNG 19.2 KB)

Supplementary Material 6 (PNG 22.7 KB)

Supplementary Material 7 (PNG 53.3 KB)

Supplementary Material 8 (PNG 135 KB)

Supplementary Material 9 (PNG 142 KB)

Supplementary Material 10 (PNG 118 KB)

Supplementary Material 11 (JPG 928 KB)

Supplementary Material 12 (JPG 400 KB)

Supplementary Material 13 (PNG 176 KB)

Supplementary Material 14 (PNG 188 KB)

Supplementary Material 15 (PNG 495 KB)

Supplementary Material 16 (PNG 158 KB)

Supplementary Material 17 (JPG 493 KB)

Supplementary Material 18 (JPG 126 KB)

Supplementary Material 19 (JPG 796 KB)

Supplementary Material 20 (JPG 2.28 MB)

Supplementary Material 21 (JPG 188 KB)

Supplementary Material 22 (JPG 497 KB)

Supplementary Material 23 (PNG 194 KB)

Supplementary Material 24 (PNG 197 KB)

Supplementary Material 25 (PNG 189 KB)

Supplementary Material 26 (PNG 194 KB)

Supplementary Material 27 (PNG 198 KB)

Supplementary Material 28 (PNG 197 KB)

Supplementary Material 29 (PNG 194 KB)

Supplementary Material 30 (PNG 197 KB)

Supplementary Material 31 (PNG 195 KB)

Supplementary Material 32 (PNG 193 KB)

Supplementary Material 33 (PNG 198 KB)

Supplementary Material 34 (PNG 188 KB)

Supplementary Material 35 (PNG 196 KB)

Supplementary Material 36 (PNG 201 KB)

Supplementary Material 37 (PNG 195 KB)

Supplementary Material 38 (PNG 195 KB)

Supplementary Material 39 (PNG 195 KB)

Supplementary Material 40 (PNG 193 KB)

Supplementary Material 41 (PNG 194 KB)

Supplementary Material 42 (PNG 204 KB)

Supplementary Material 43 (PNG 196 KB)

Supplementary Material 44 (PNG 194 KB)

Supplementary Material 45 (PNG 198 KB)

Supplementary Material 46 (PNG 196 KB)

Supplementary Material 47 (PNG 24.2 KB)

Supplementary Material 48 (PNG 24.4 KB)

Supplementary Material 49 (PNG 22.7 KB)

Supplementary Material 50 (PNG 22.6 KB)

Supplementary Material 51 (PNG 20.5 KB)

Supplementary Material 52 (PNG 20.0 KB)

Supplementary Material 53 (PNG 21.0 KB)

Supplementary Material 54 (PNG 19.8 KB)

Supplementary Material 55 (PNG 22.7 KB)

Supplementary Material 56 (PNG 22.6 KB)

Supplementary Material 57 (PNG 21.6 KB)

Supplementary Material 58 (PNG 20.7 KB)

Supplementary Material 59 (PNG 26.8 KB)

Supplementary Material 60 (PNG 24.8 KB)

Supplementary Material 61 (PNG 22.8 KB)

Supplementary Material 62 (PNG 25.7 KB)

Supplementary Material 63 (PNG 23.8 KB)

Supplementary Material 64 (PNG 23.1 KB)

Supplementary Material 65 (DOCX 7.84 MB)
