# Supplementary figures and images for: Pangenomic and genomic plasticity analyses of the genus Rickettsia
Source: Braz J Microbiol. 2026 Jul 24;57(1):217. doi: 10.1007/s42770-026-02030-7 (PMC13400510; doi:10.1007/s42770-026-02030-7)

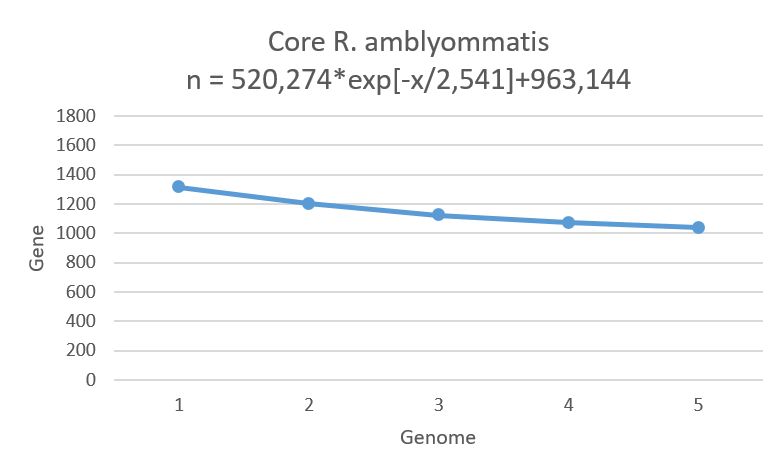

Supplement: Supplementary file 1 — Supplementary Material 1 (PNG 23.4 KB) [file 42770_2026_2030_MOESM1_ESM.png]

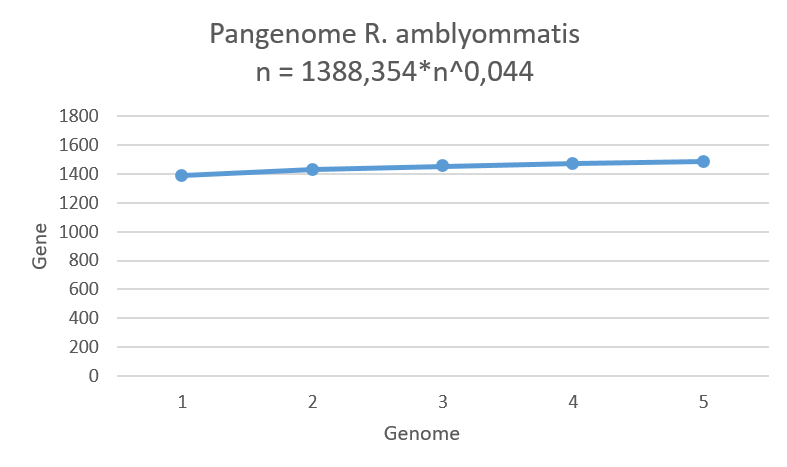

Supplement: Supplementary file 2 — Supplementary Material 2 (PNG 21.2 KB) [file 42770_2026_2030_MOESM2_ESM.png]

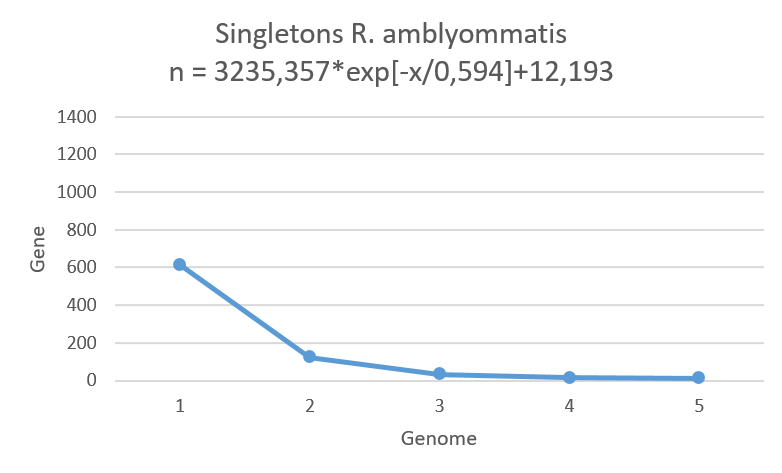

Supplement: Supplementary file 3 — Supplementary Material 3 (PNG 23.9 KB) [file 42770_2026_2030_MOESM3_ESM.png]

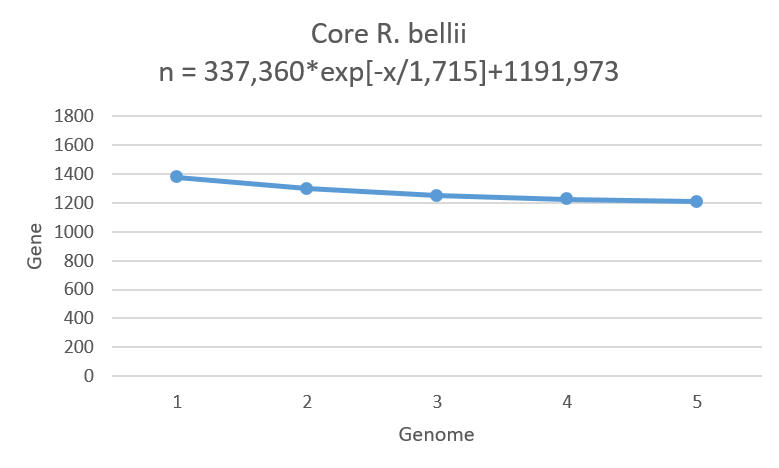

Supplement: Supplementary file 4 — Supplementary Material 4 (PNG 20.5 KB) [file 42770_2026_2030_MOESM4_ESM.png]

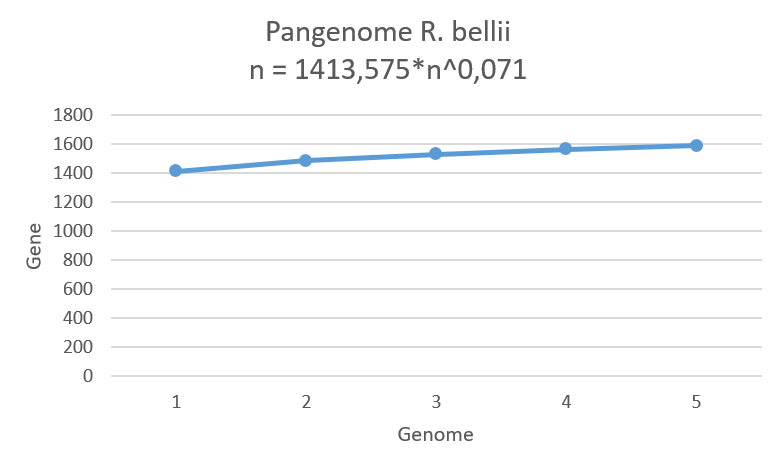

Supplement: Supplementary file 5 — Supplementary Material 5 (PNG 19.2 KB) [file 42770_2026_2030_MOESM5_ESM.png]

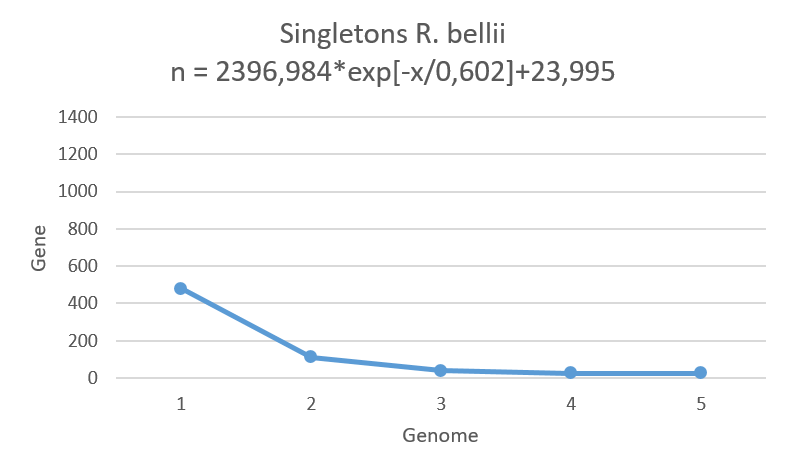

Supplement: Supplementary file 6 — Supplementary Material 6 (PNG 22.7 KB) [file 42770_2026_2030_MOESM6_ESM.png]

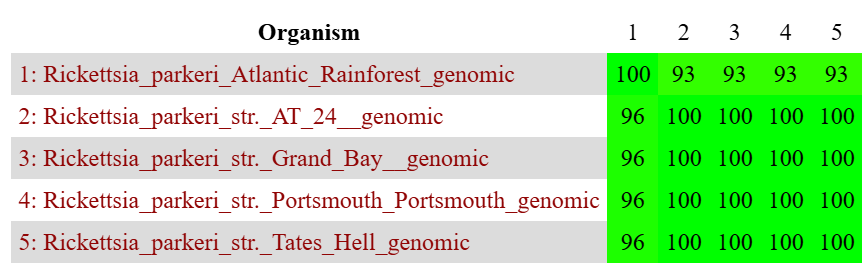

Supplement: Supplementary file 7 — Supplementary Material 7 (PNG 53.3 KB) [file 42770_2026_2030_MOESM7_ESM.png]

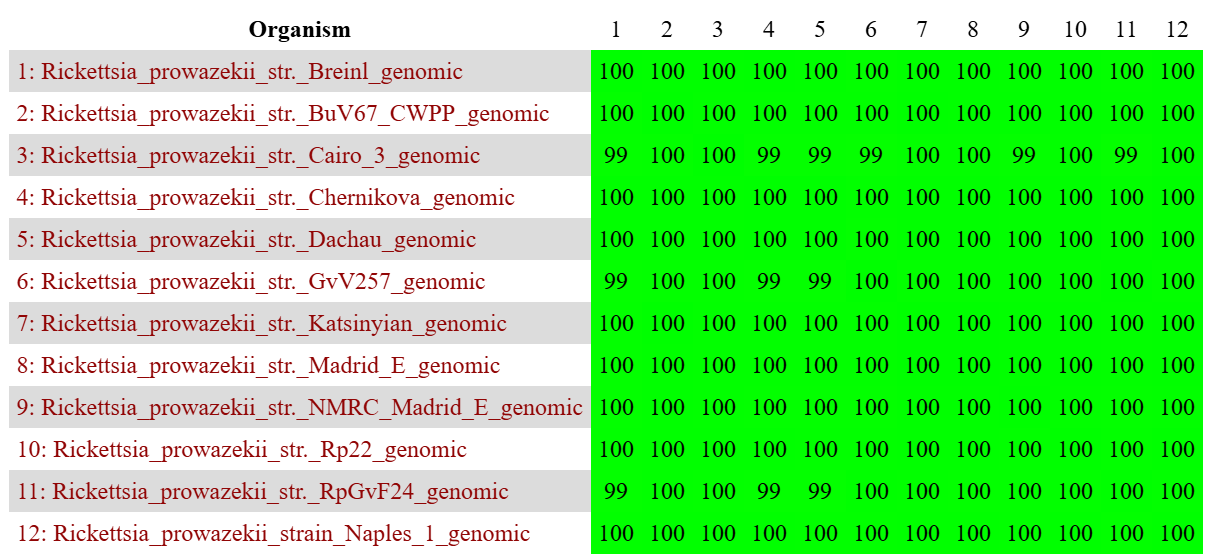

Supplement: Supplementary file 8 — Supplementary Material 8 (PNG 135 KB) [file 42770_2026_2030_MOESM8_ESM.png]

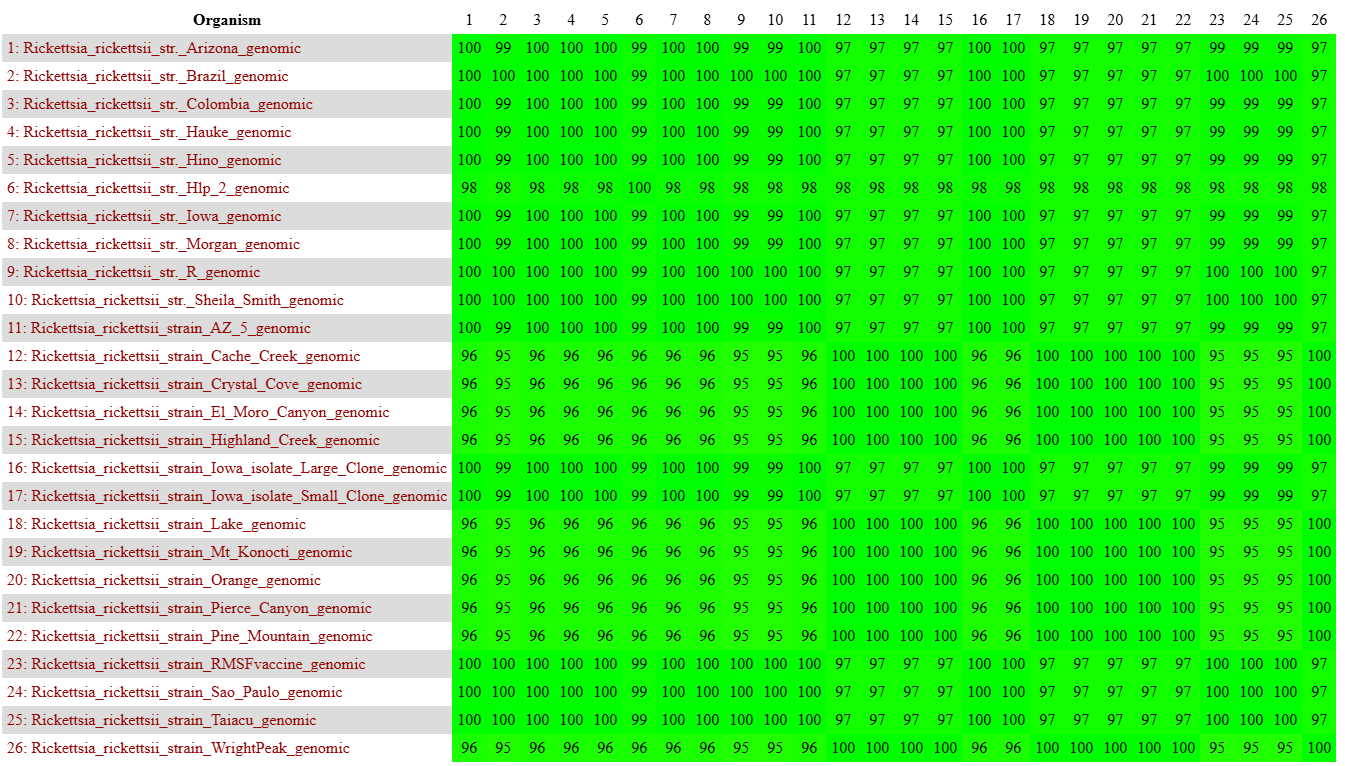

Supplement: Supplementary file 9 — Supplementary Material 9 (PNG 142 KB) [file 42770_2026_2030_MOESM9_ESM.png]

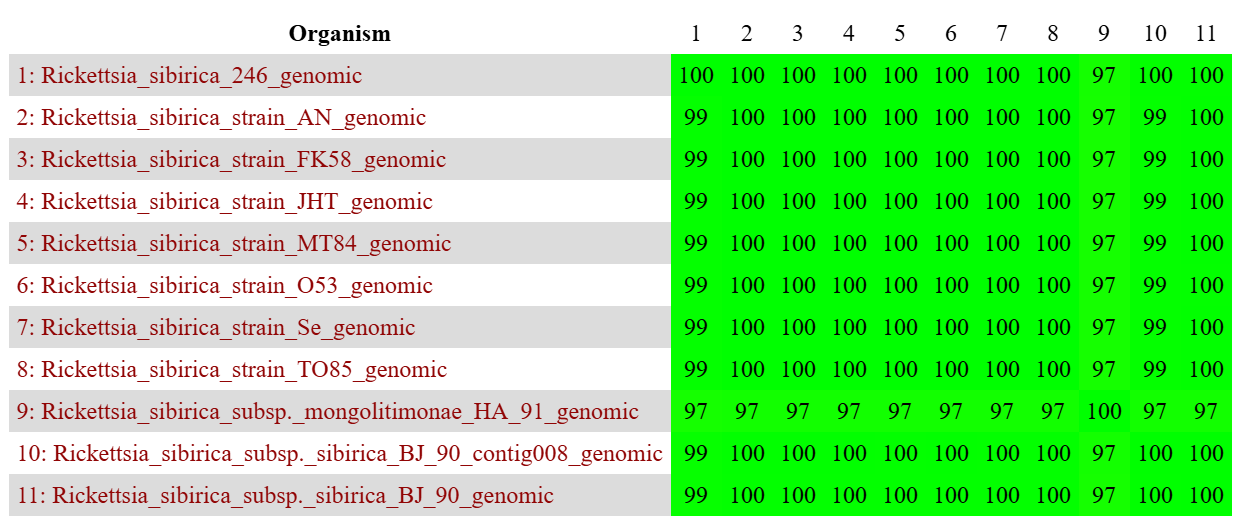

Supplement: Supplementary file 10 — Supplementary Material 10 (PNG 118 KB) [file 42770_2026_2030_MOESM10_ESM.png]

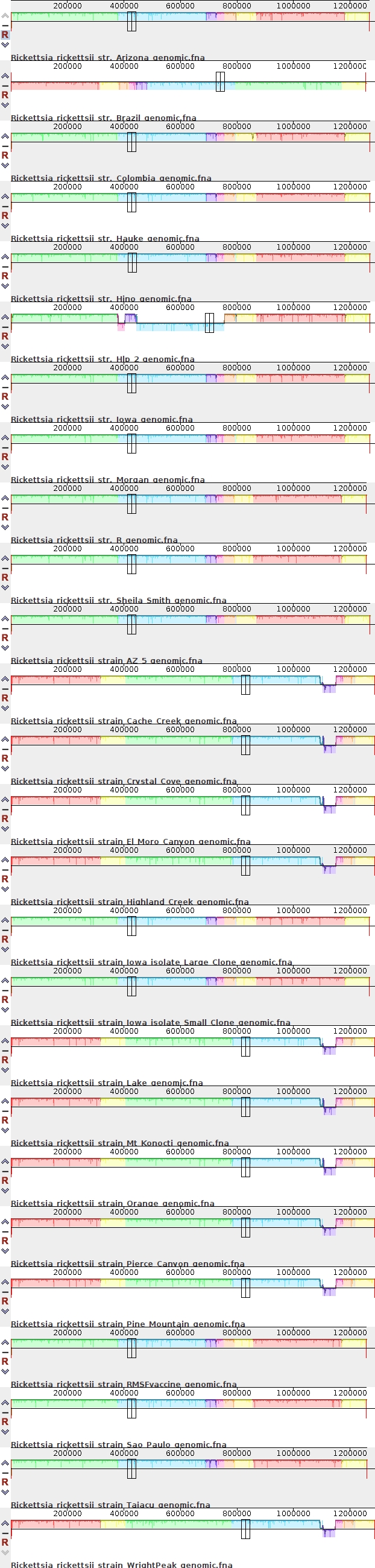

Supplement: Supplementary file 11 — Supplementary Material 11 (JPG 928 KB) [file 42770_2026_2030_MOESM11_ESM.jpg]

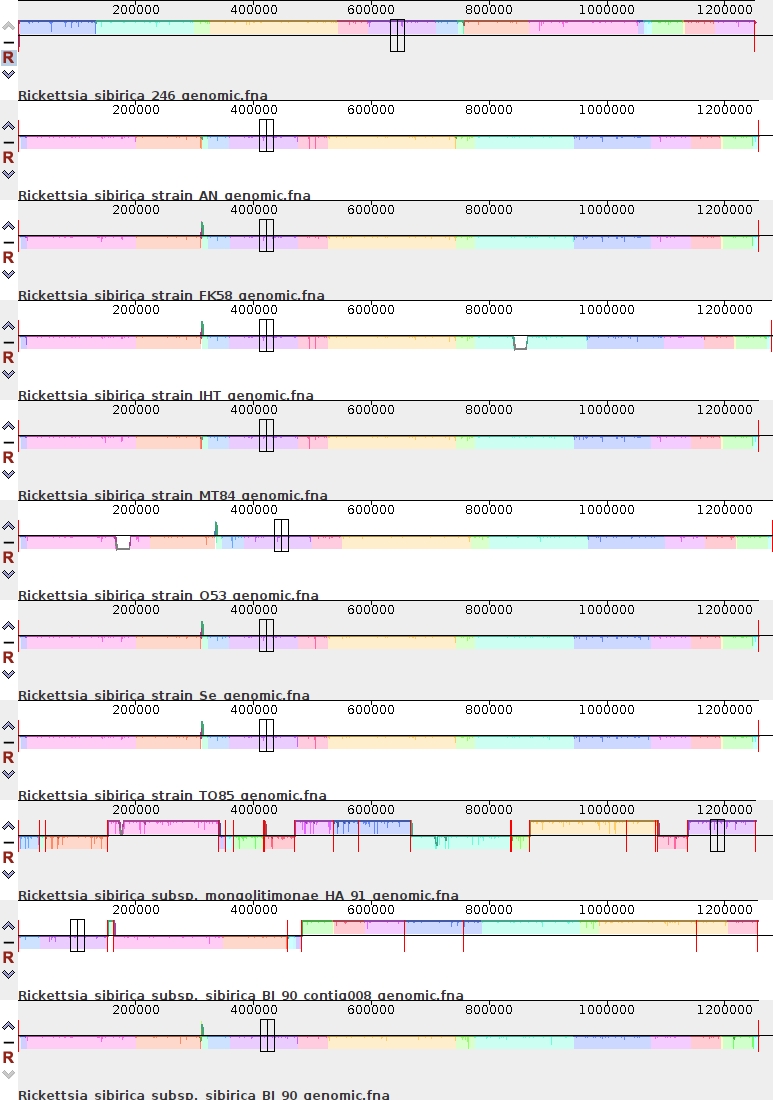

Supplement: Supplementary file 12 — Supplementary Material 12 (JPG 400 KB) [file 42770_2026_2030_MOESM12_ESM.jpg]

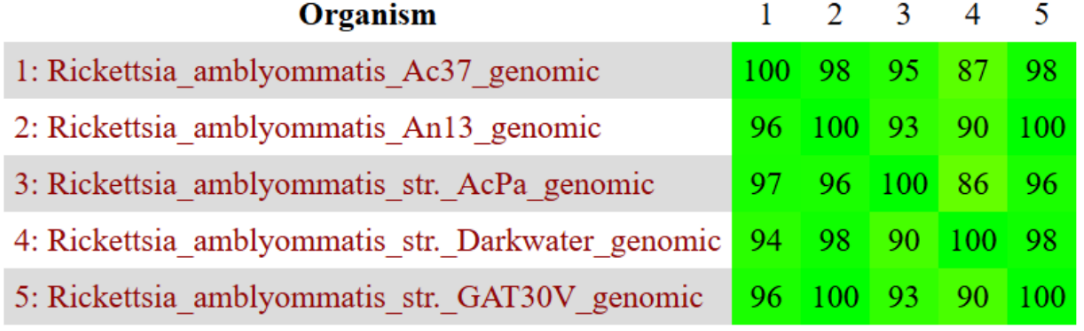

Supplement: Supplementary file 13 — Supplementary Material 13 (PNG 176 KB) [file 42770_2026_2030_MOESM13_ESM.png]

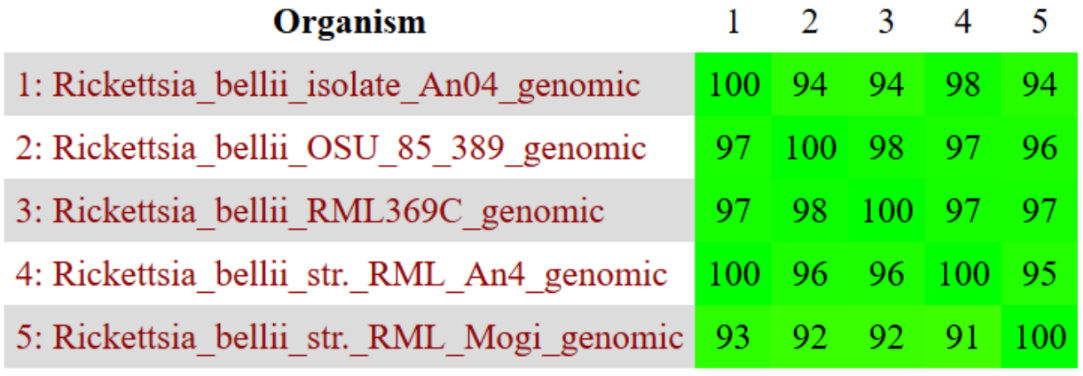

Supplement: Supplementary file 14 — Supplementary Material 14 (PNG 188 KB) [file 42770_2026_2030_MOESM14_ESM.png]

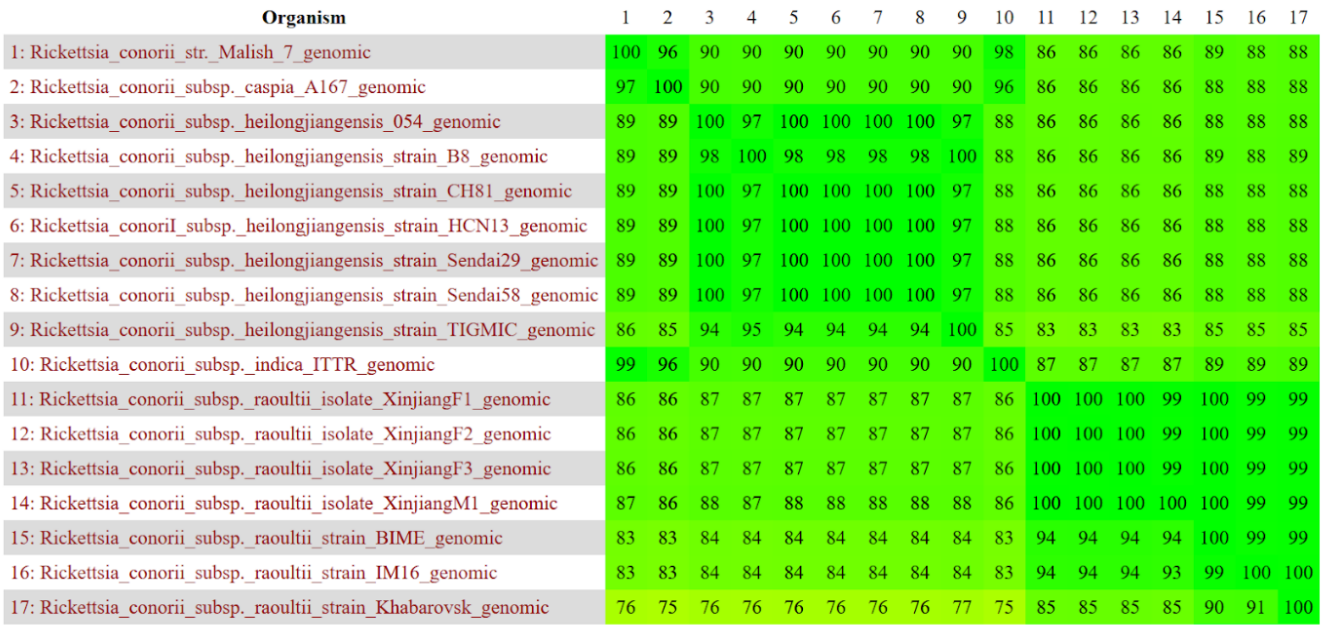

Supplement: Supplementary file 15 — Supplementary Material 15 (PNG 495 KB) [file 42770_2026_2030_MOESM15_ESM.png]

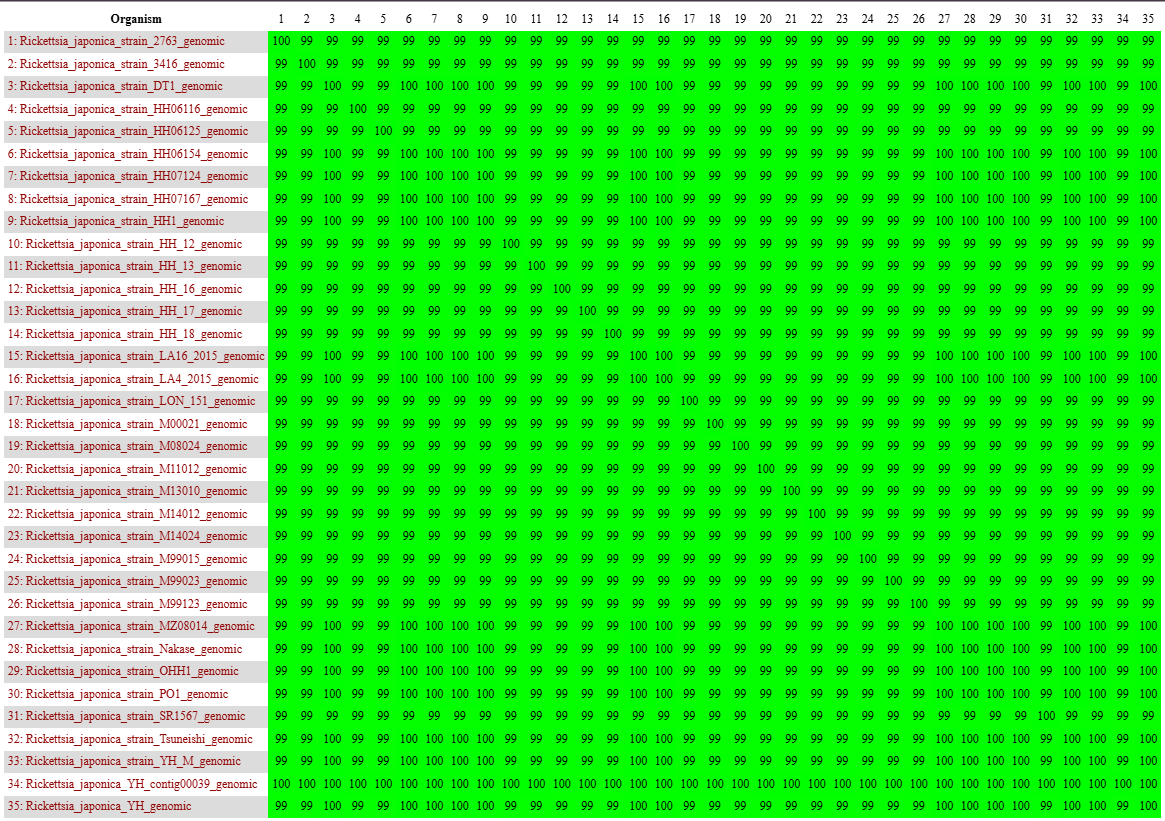

Supplement: Supplementary file 16 — Supplementary Material 16 (PNG 158 KB) [file 42770_2026_2030_MOESM16_ESM.png]

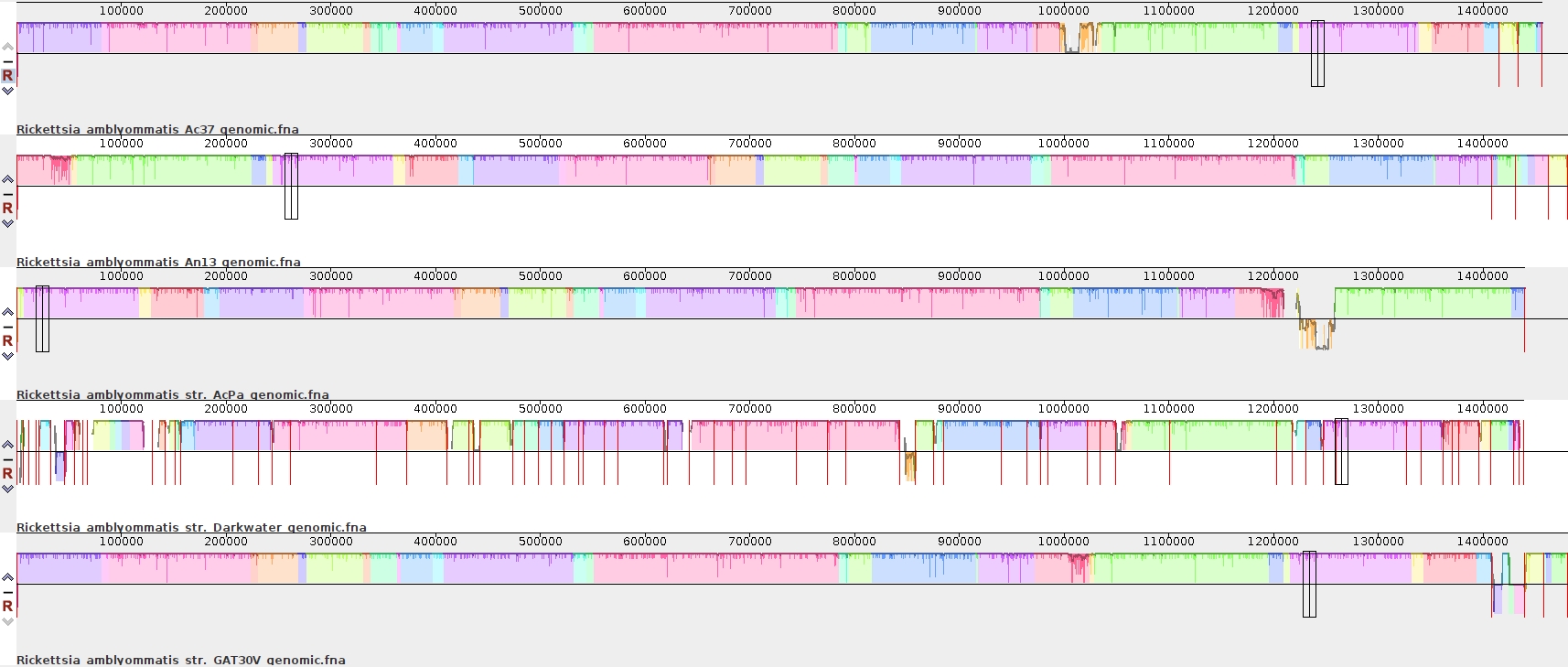

Supplement: Supplementary file 17 — Supplementary Material 17 (JPG 493 KB) [file 42770_2026_2030_MOESM17_ESM.jpg]

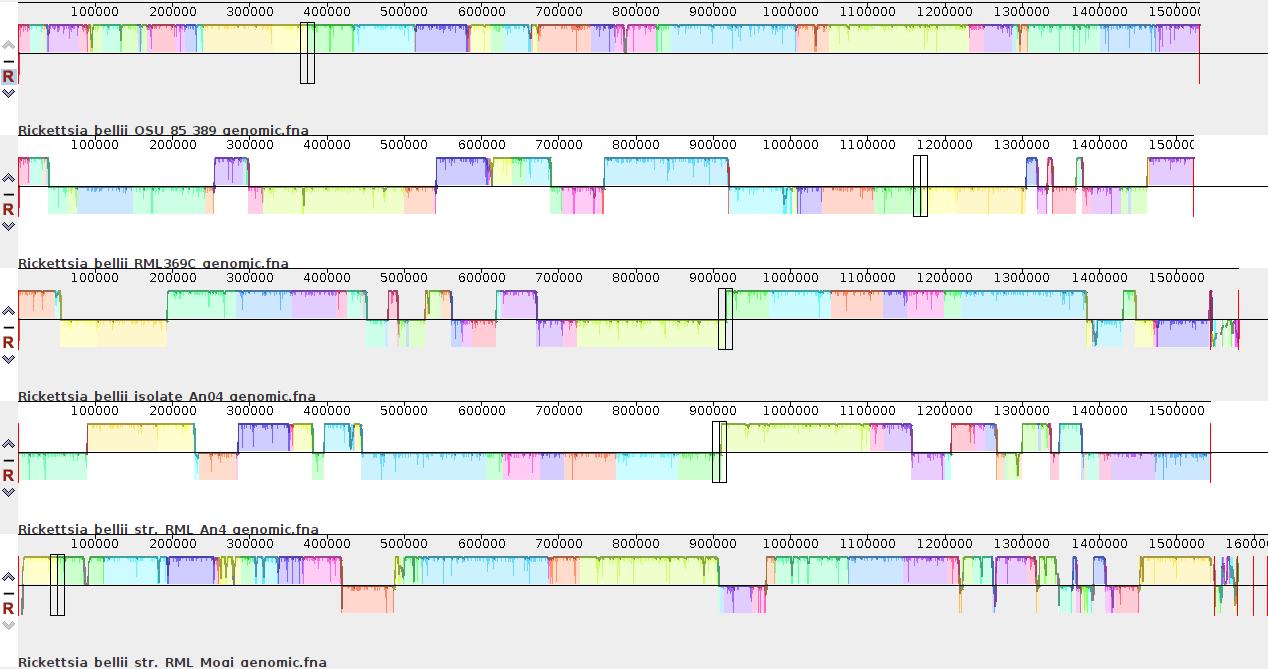

Supplement: Supplementary file 18 — Supplementary Material 18 (JPG 126 KB) [file 42770_2026_2030_MOESM18_ESM.jpg]

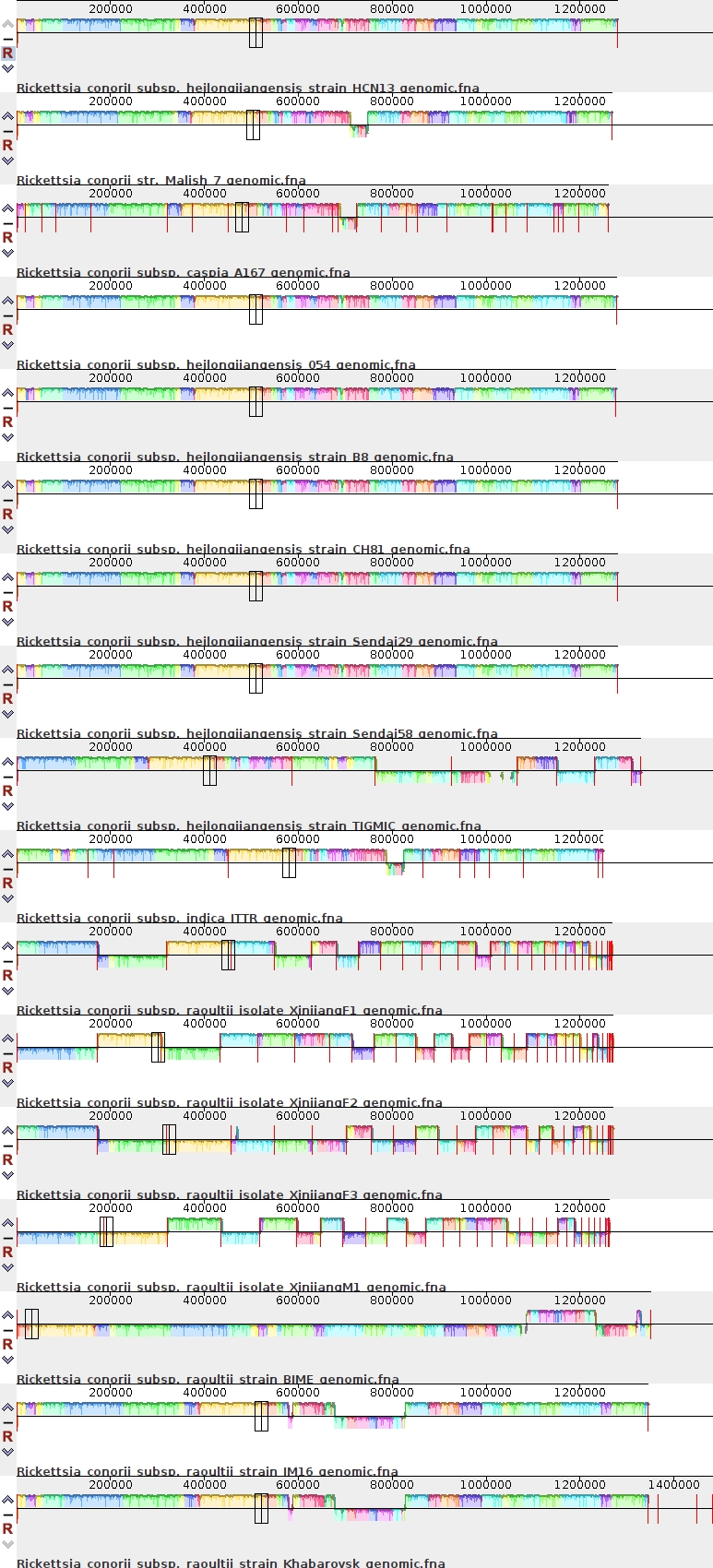

Supplement: Supplementary file 19 — Supplementary Material 19 (JPG 796 KB) [file 42770_2026_2030_MOESM19_ESM.jpg]

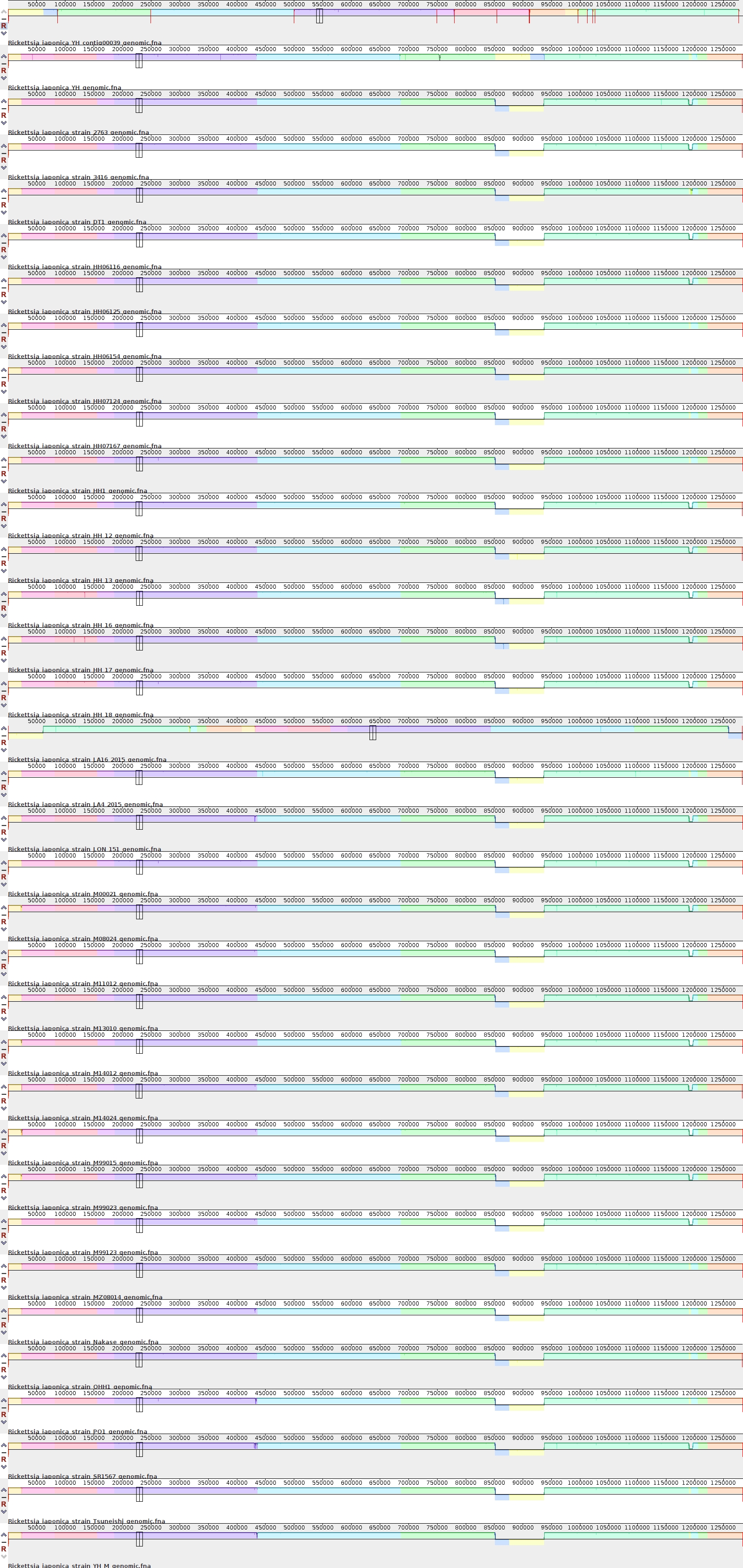

Supplement: Supplementary file 20 — Supplementary Material 20 (JPG 2.28 MB) [file 42770_2026_2030_MOESM20_ESM.jpg]

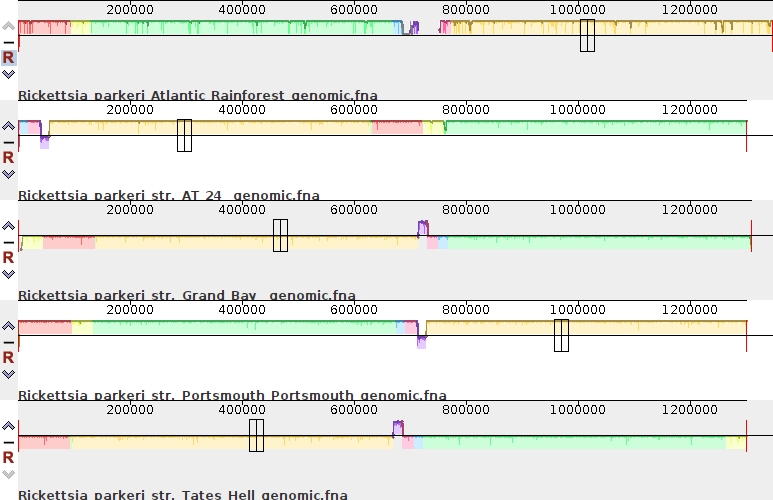

Supplement: Supplementary file 21 — Supplementary Material 21 (JPG 188 KB) [file 42770_2026_2030_MOESM21_ESM.jpg]

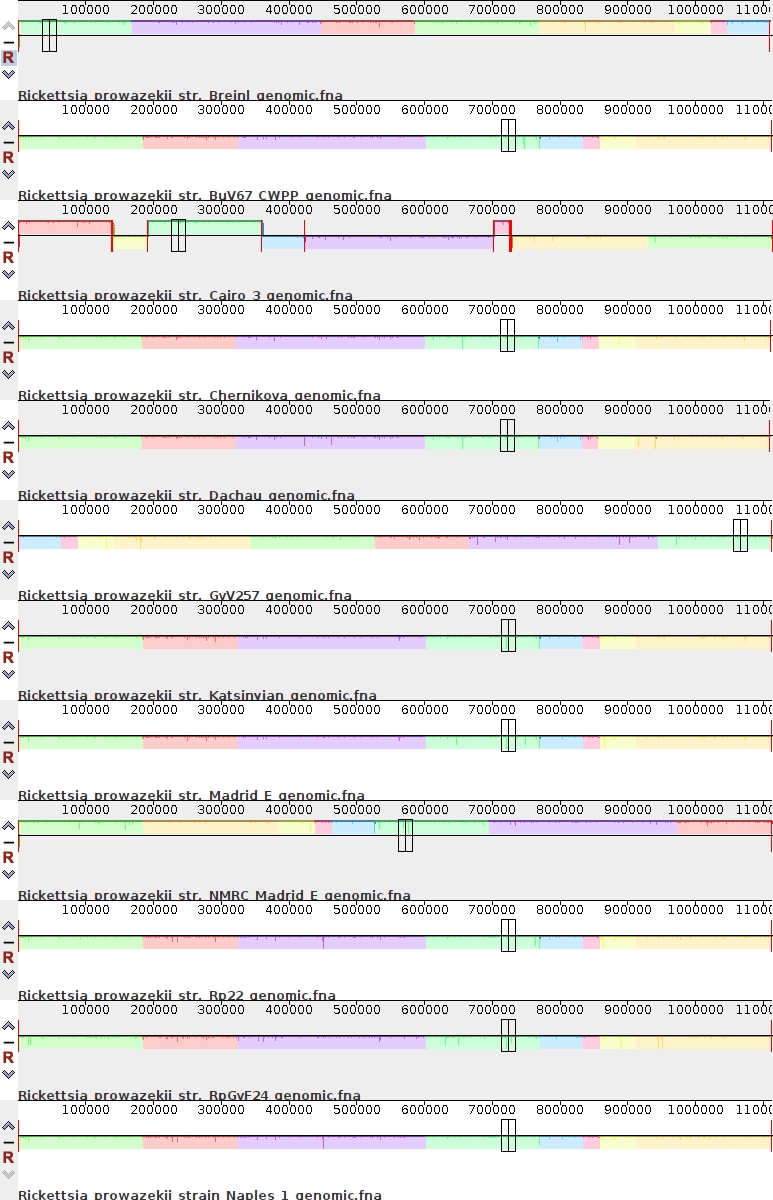

Supplement: Supplementary file 22 — Supplementary Material 22 (JPG 497 KB) [file 42770_2026_2030_MOESM22_ESM.jpg]

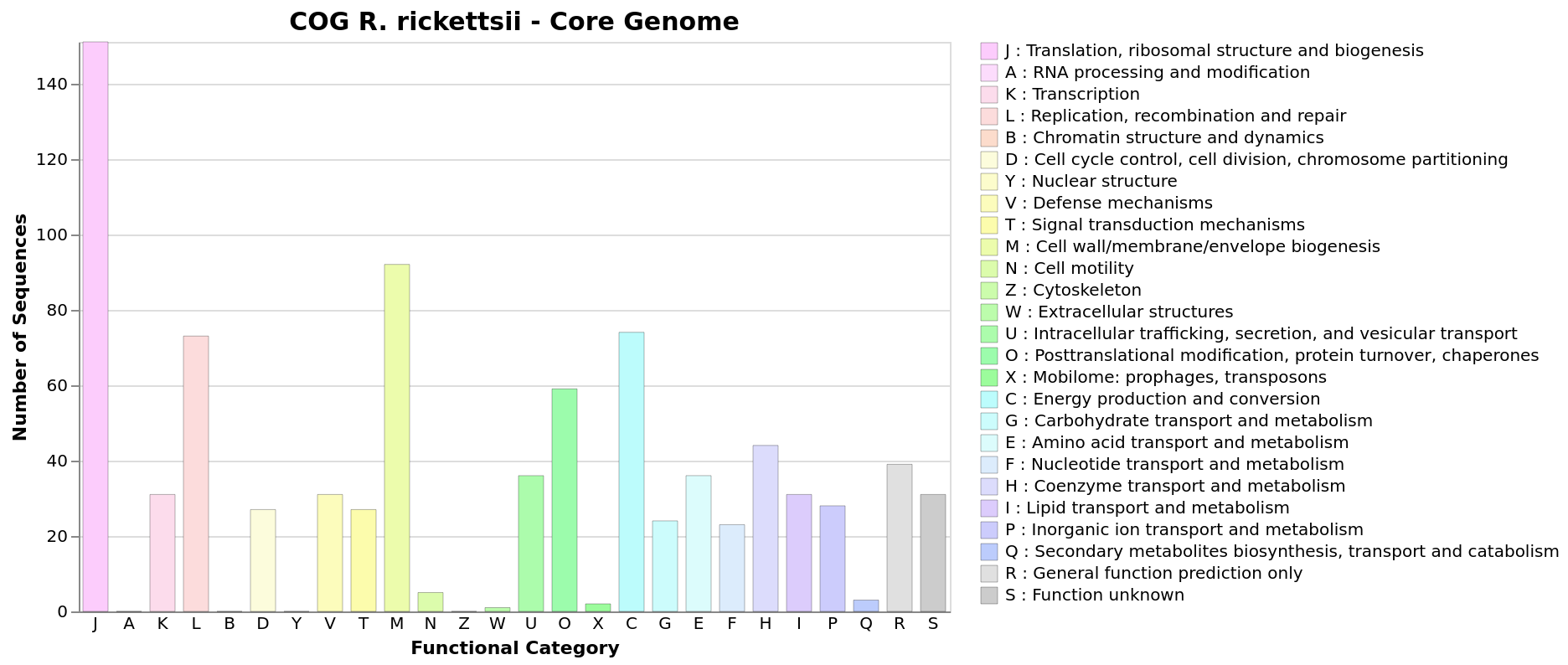

Supplement: Supplementary file 23 — Supplementary Material 23 (PNG 194 KB) [file 42770_2026_2030_MOESM23_ESM.png]

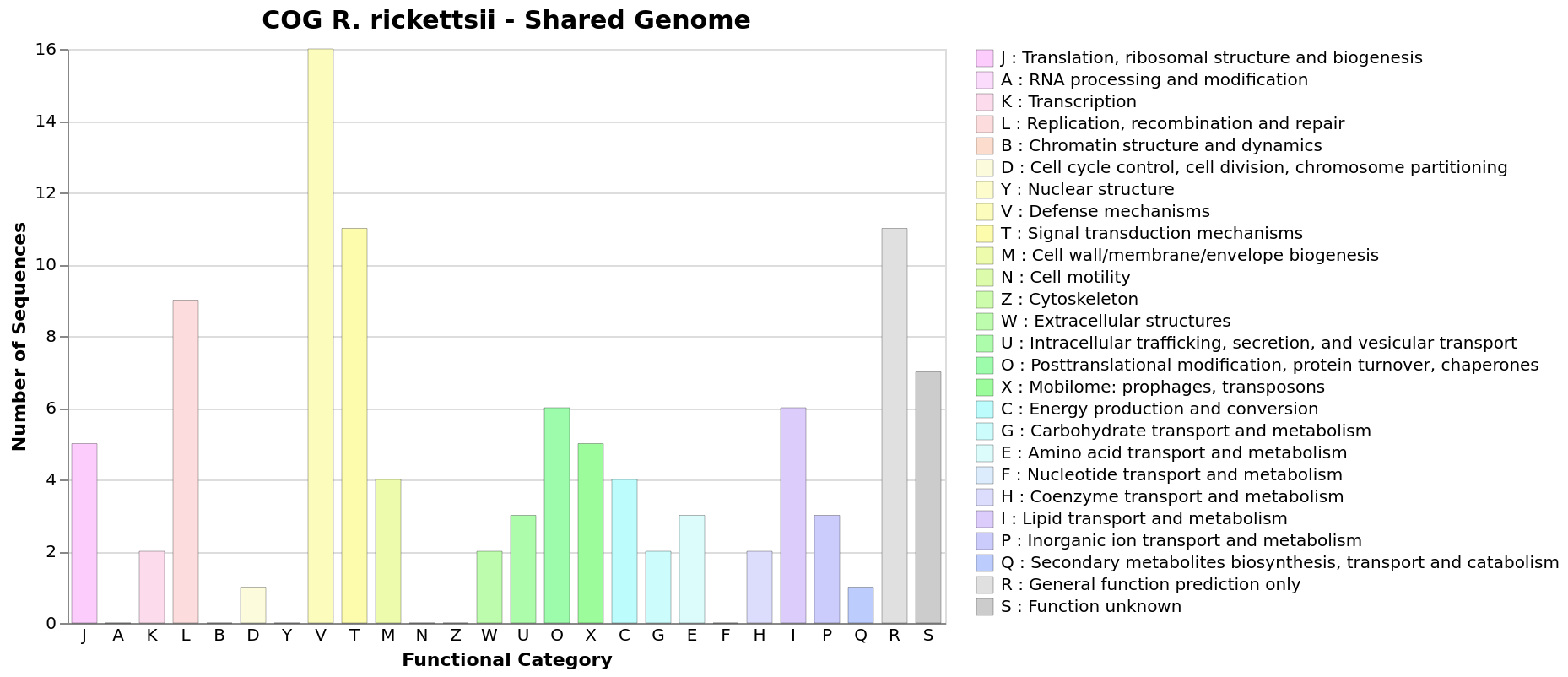

Supplement: Supplementary file 24 — Supplementary Material 24 (PNG 197 KB) [file 42770_2026_2030_MOESM24_ESM.png]

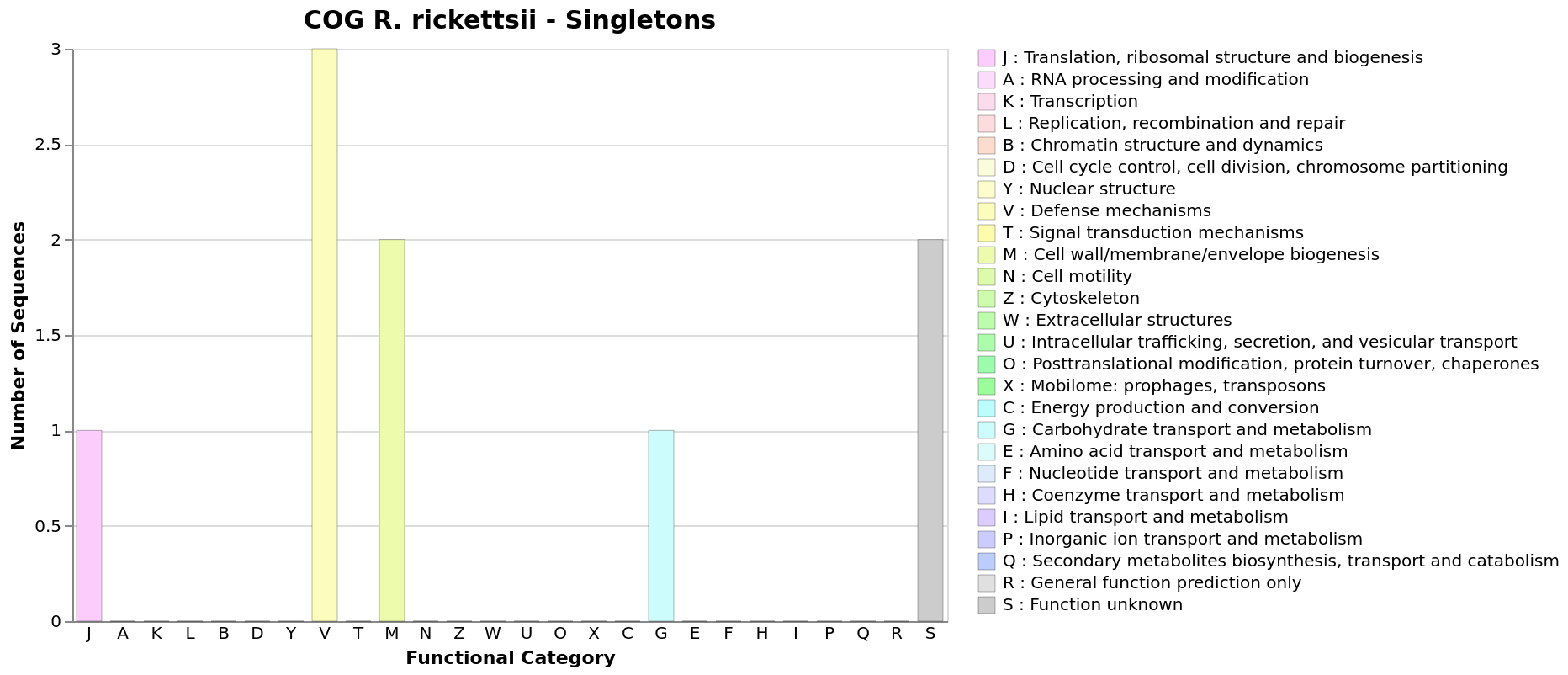

Supplement: Supplementary file 25 — Supplementary Material 25 (PNG 189 KB) [file 42770_2026_2030_MOESM25_ESM.png]

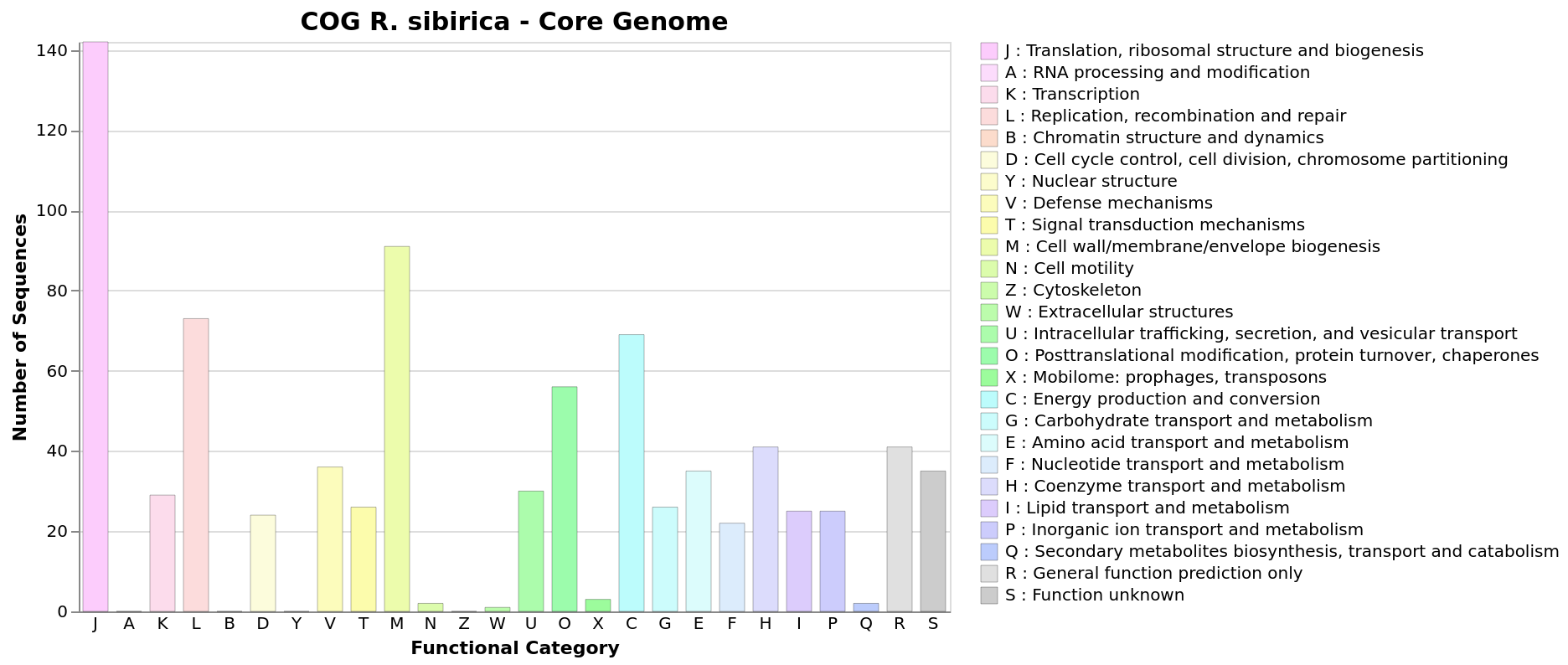

Supplement: Supplementary file 26 — Supplementary Material 26 (PNG 194 KB) [file 42770_2026_2030_MOESM26_ESM.png]

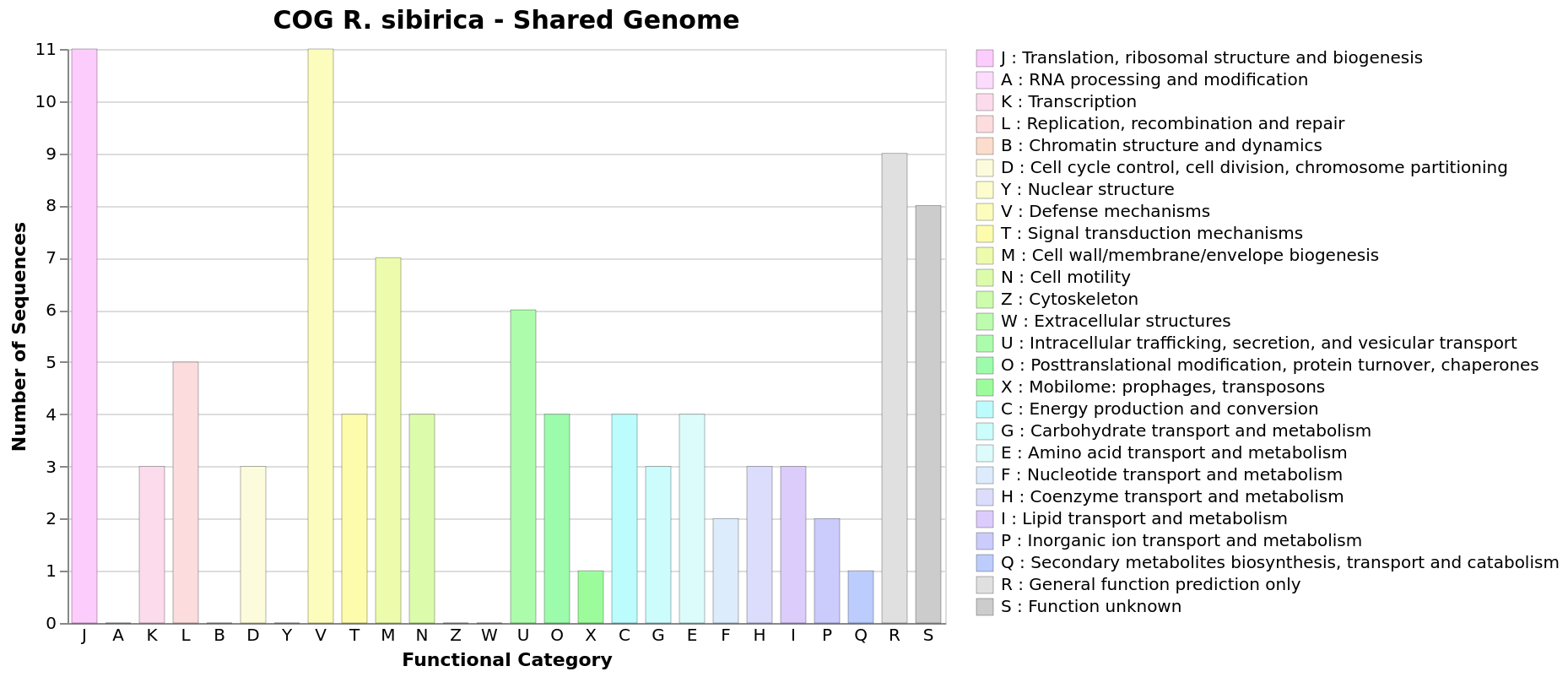

Supplement: Supplementary file 27 — Supplementary Material 27 (PNG 198 KB) [file 42770_2026_2030_MOESM27_ESM.png]

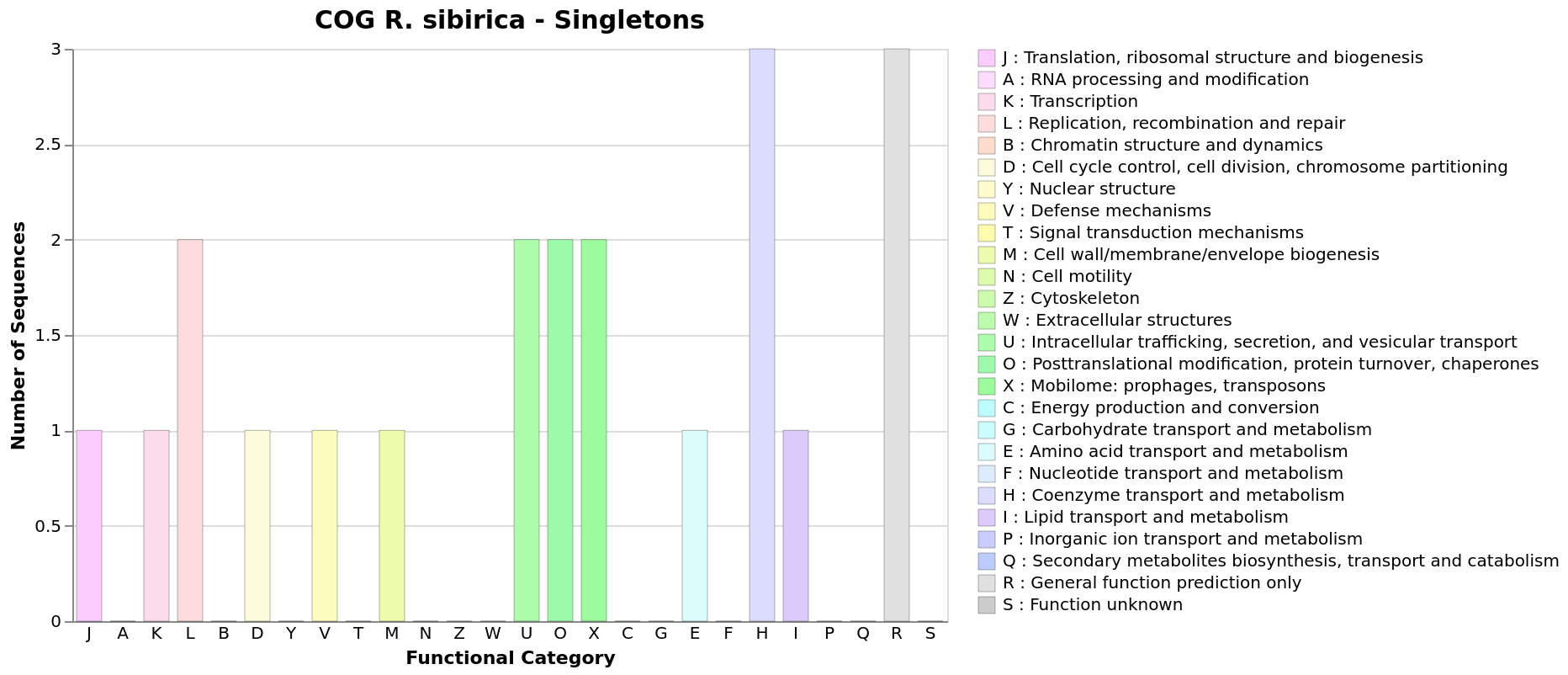

Supplement: Supplementary file 28 — Supplementary Material 28 (PNG 197 KB) [file 42770_2026_2030_MOESM28_ESM.png]

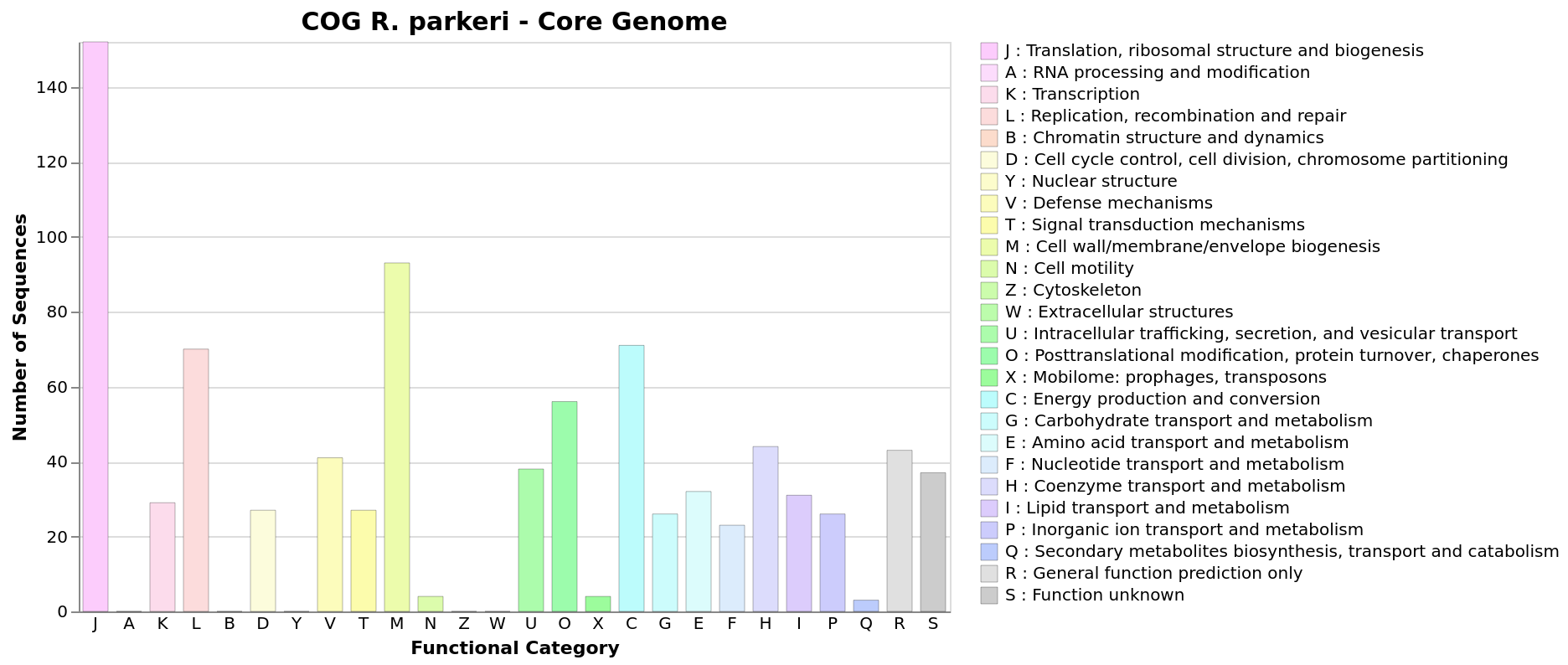

Supplement: Supplementary file 29 — Supplementary Material 29 (PNG 194 KB) [file 42770_2026_2030_MOESM29_ESM.png]

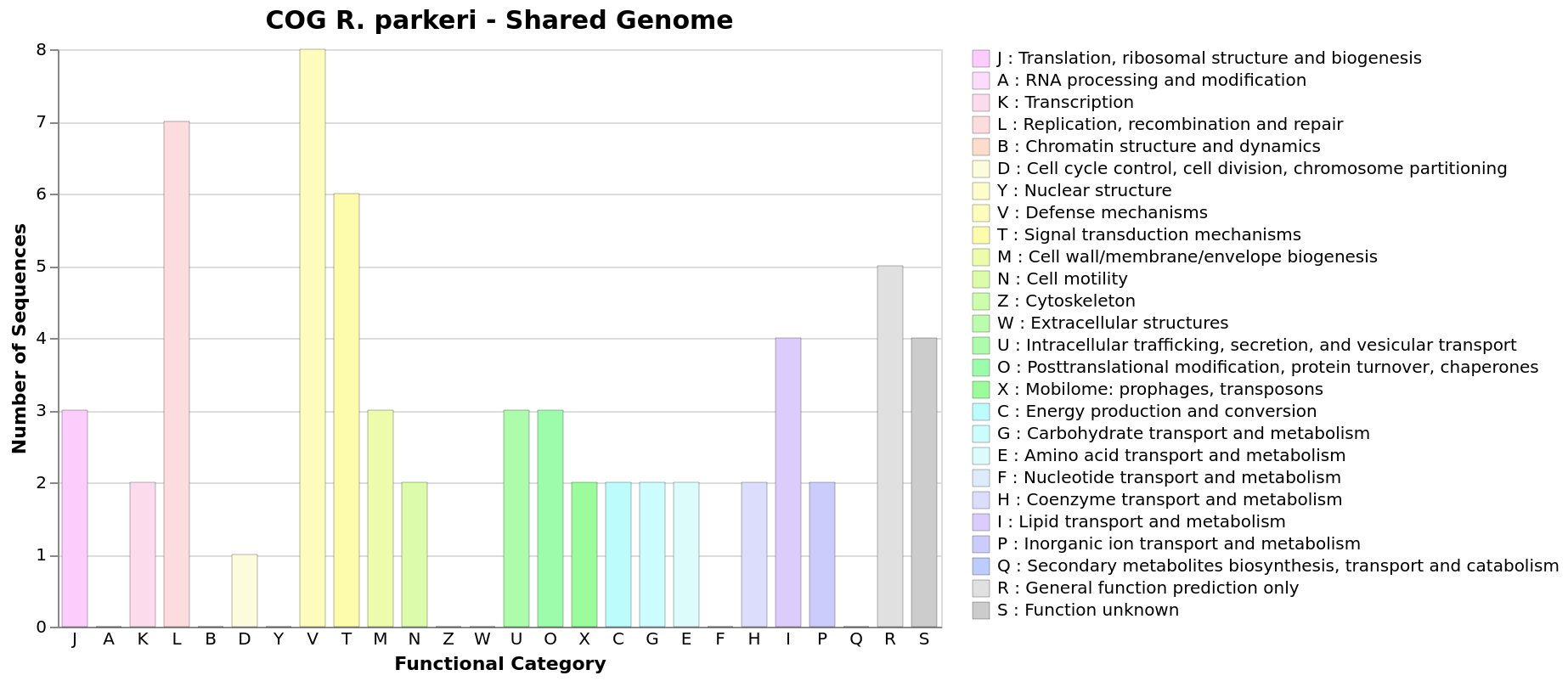

Supplement: Supplementary file 30 — Supplementary Material 30 (PNG 197 KB) [file 42770_2026_2030_MOESM30_ESM.png]

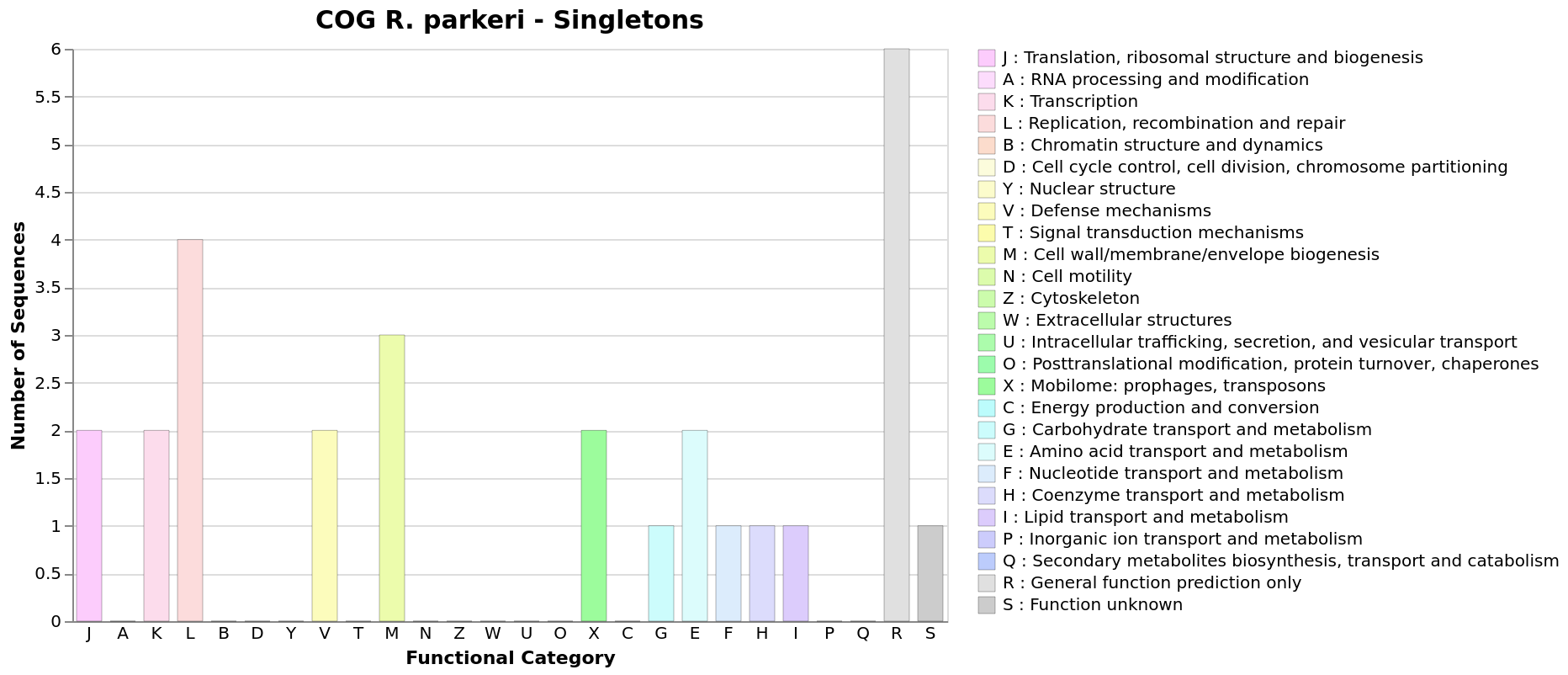

Supplement: Supplementary file 31 — Supplementary Material 31 (PNG 195 KB) [file 42770_2026_2030_MOESM31_ESM.png]

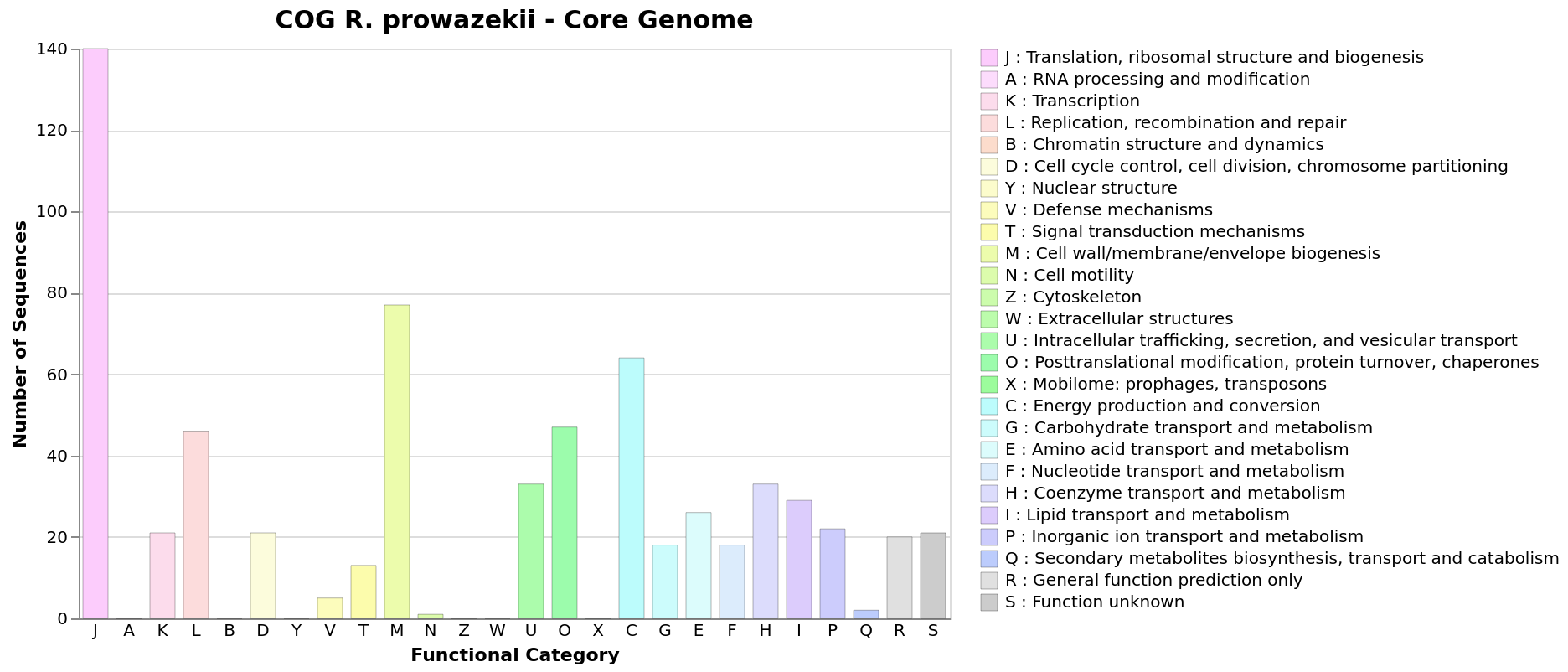

Supplement: Supplementary file 32 — Supplementary Material 32 (PNG 193 KB) [file 42770_2026_2030_MOESM32_ESM.png]

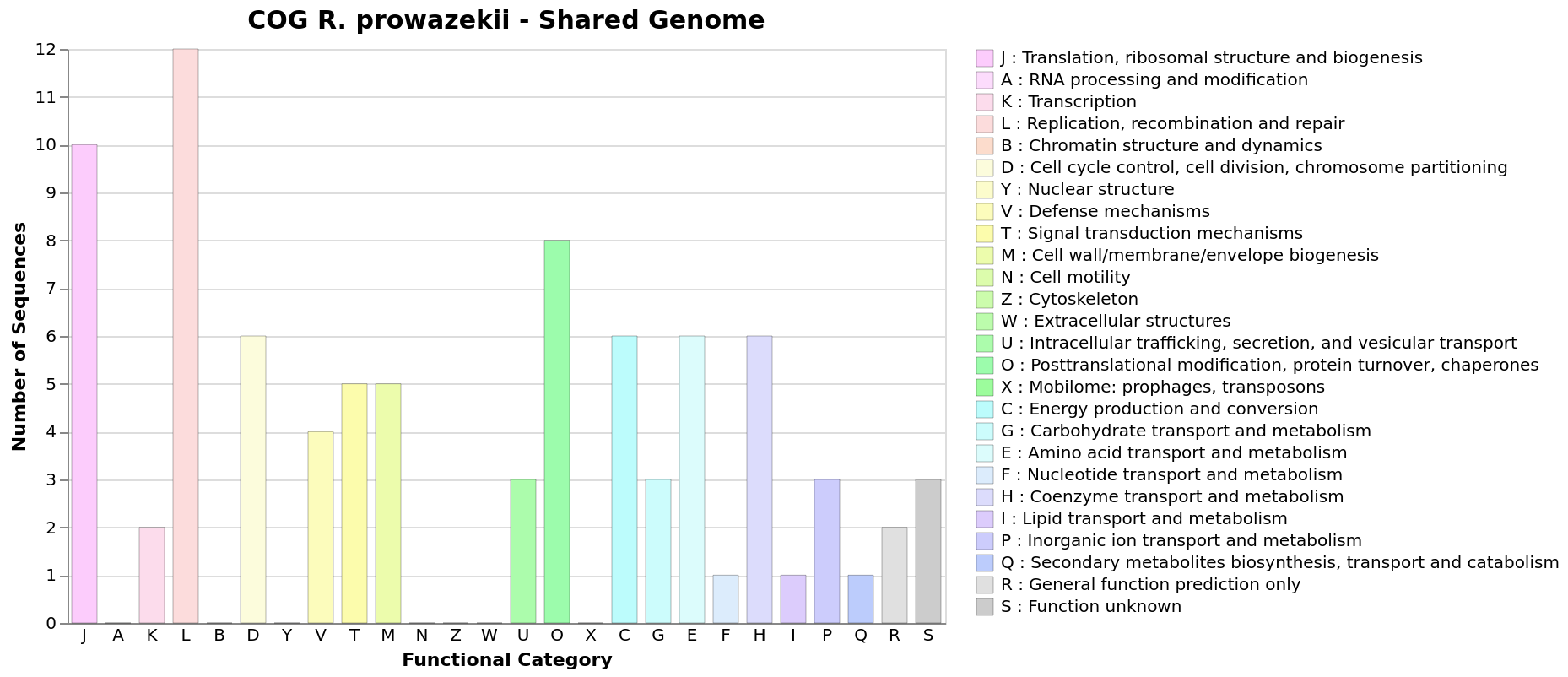

Supplement: Supplementary file 33 — Supplementary Material 33 (PNG 198 KB) [file 42770_2026_2030_MOESM33_ESM.png]

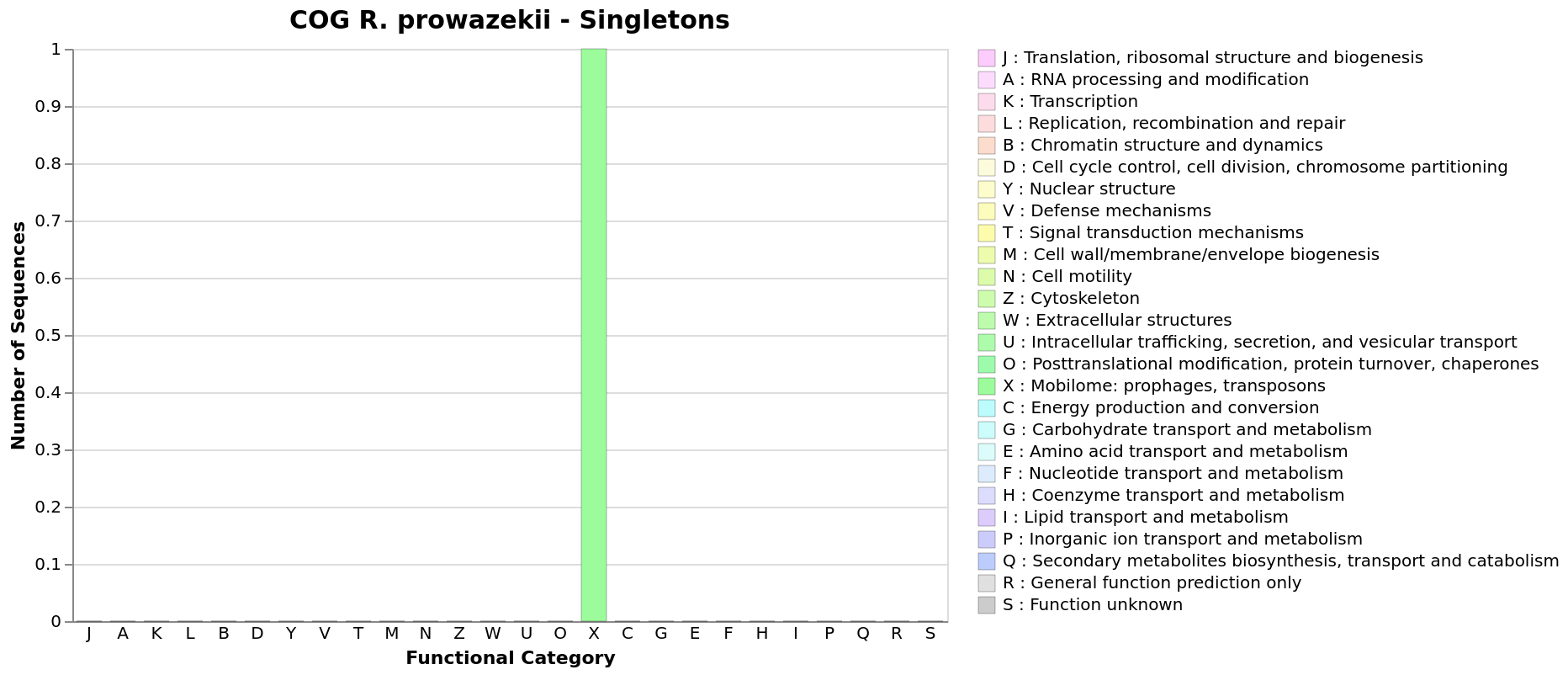

Supplement: Supplementary file 34 — Supplementary Material 34 (PNG 188 KB) [file 42770_2026_2030_MOESM34_ESM.png]

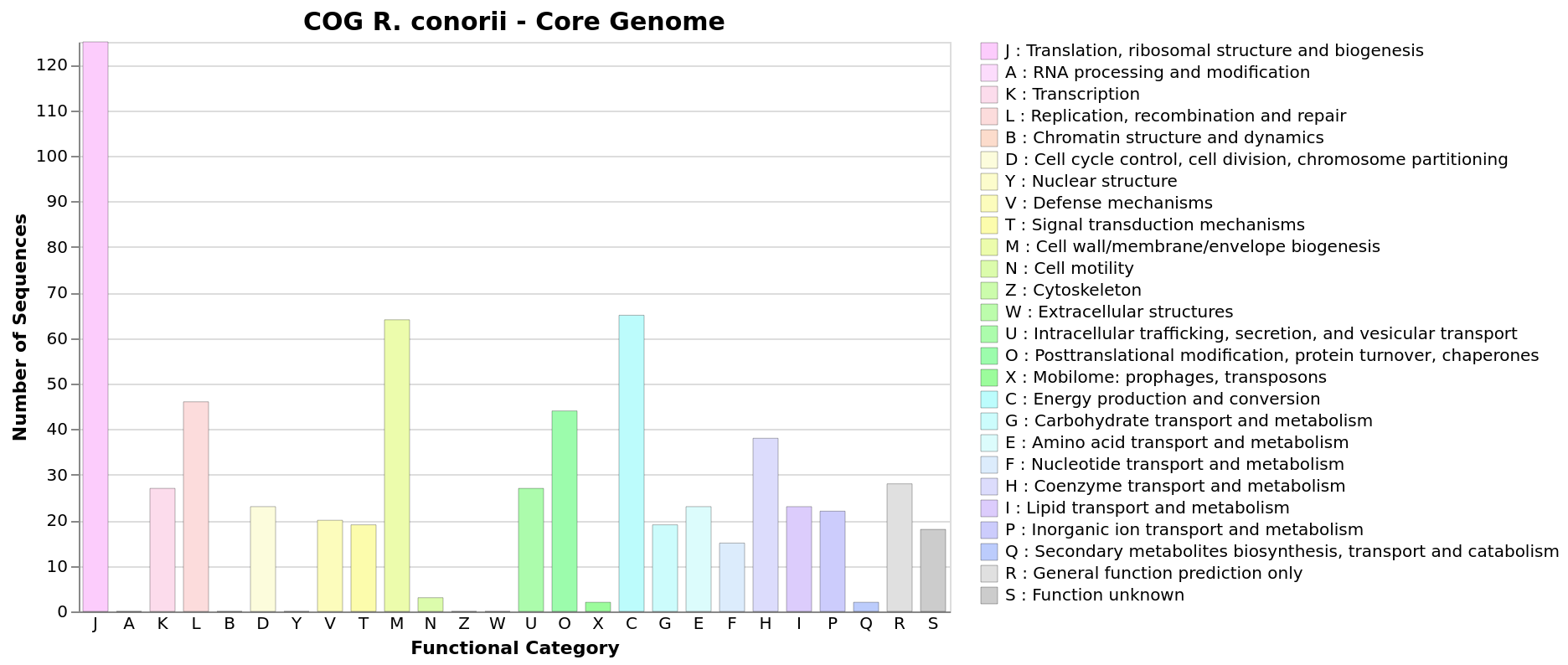

Supplement: Supplementary file 35 — Supplementary Material 35 (PNG 196 KB) [file 42770_2026_2030_MOESM35_ESM.png]

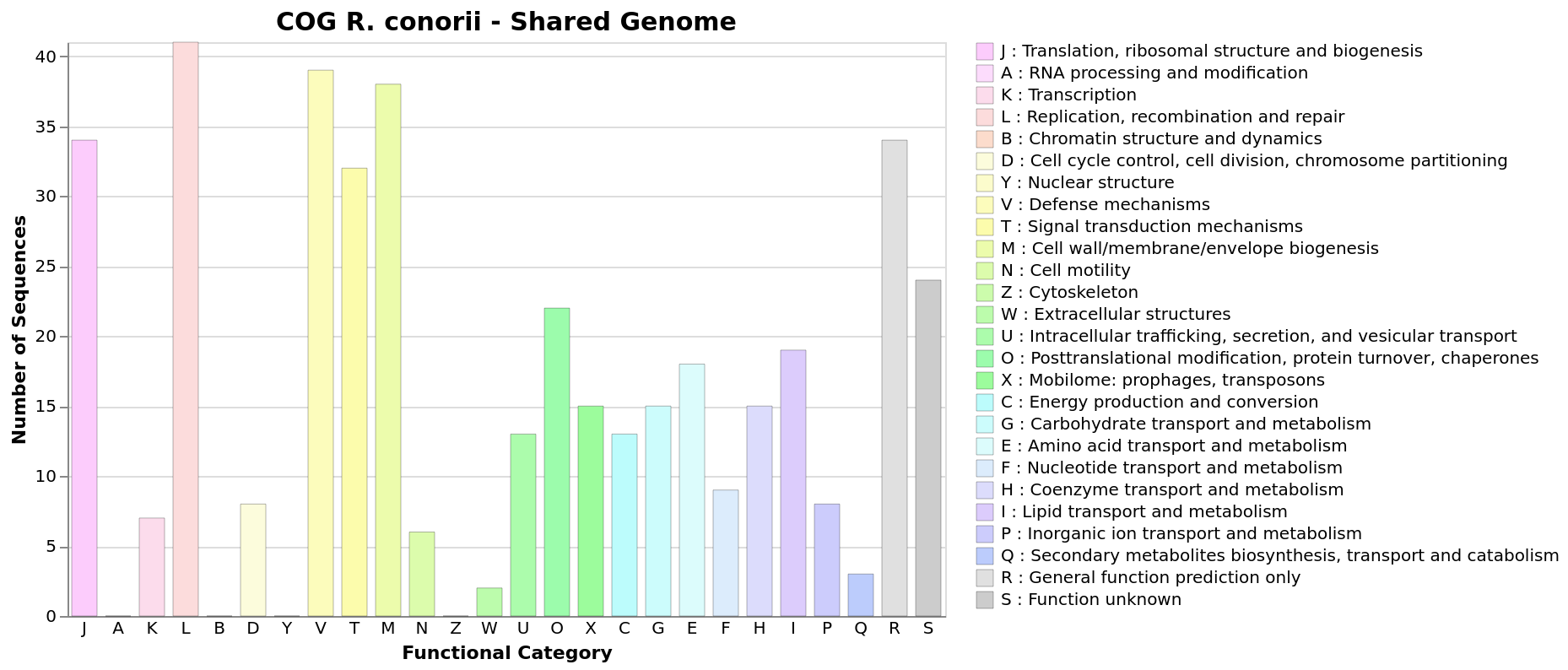

Supplement: Supplementary file 36 — Supplementary Material 36 (PNG 201 KB) [file 42770_2026_2030_MOESM36_ESM.png]

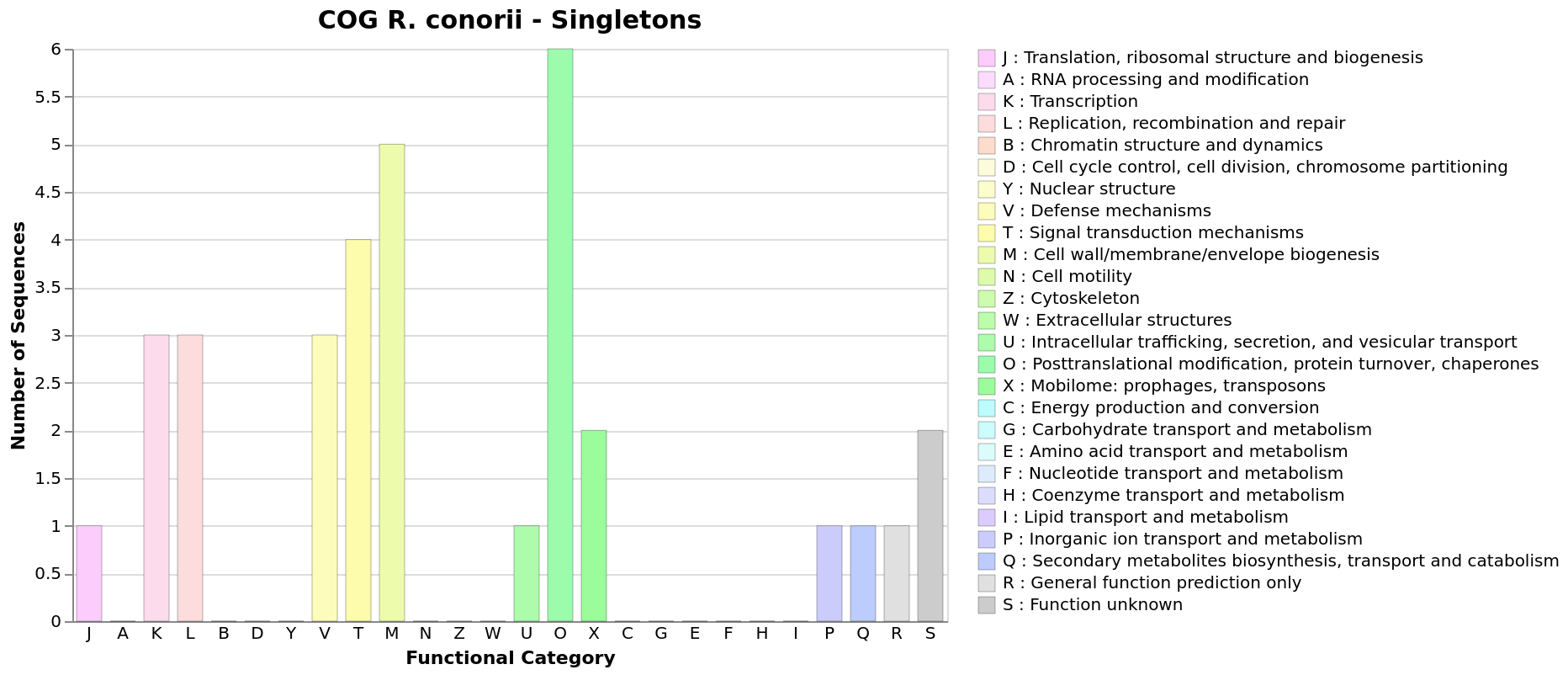

Supplement: Supplementary file 37 — Supplementary Material 37 (PNG 195 KB) [file 42770_2026_2030_MOESM37_ESM.png]

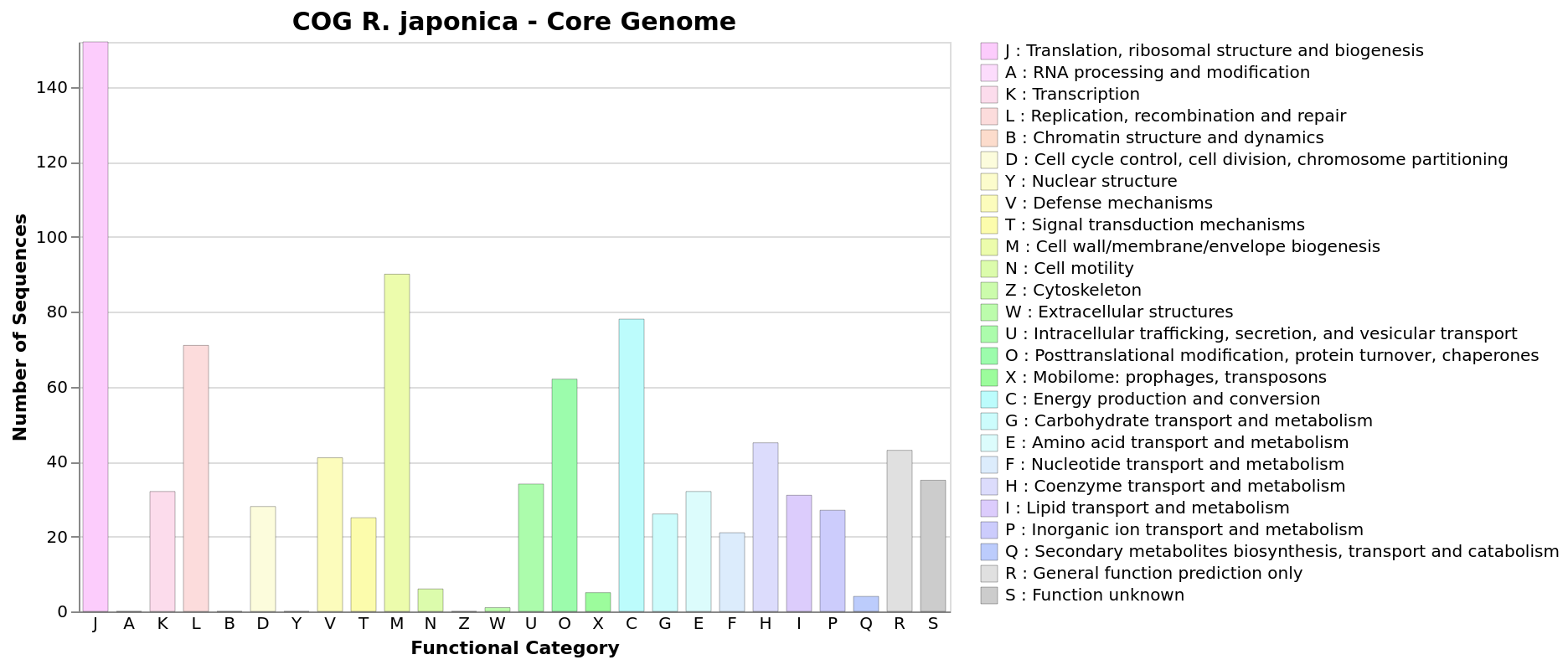

Supplement: Supplementary file 38 — Supplementary Material 38 (PNG 195 KB) [file 42770_2026_2030_MOESM38_ESM.png]

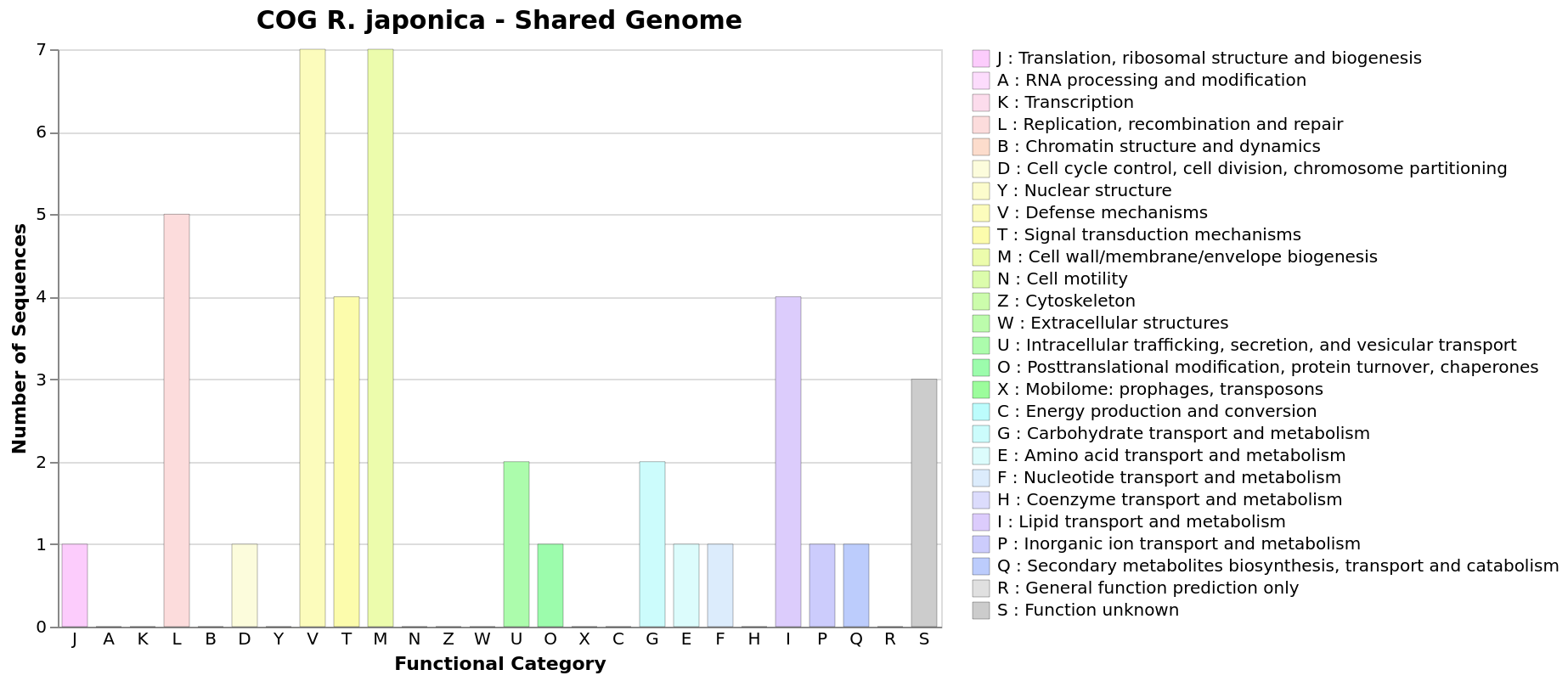

Supplement: Supplementary file 39 — Supplementary Material 39 (PNG 195 KB) [file 42770_2026_2030_MOESM39_ESM.png]

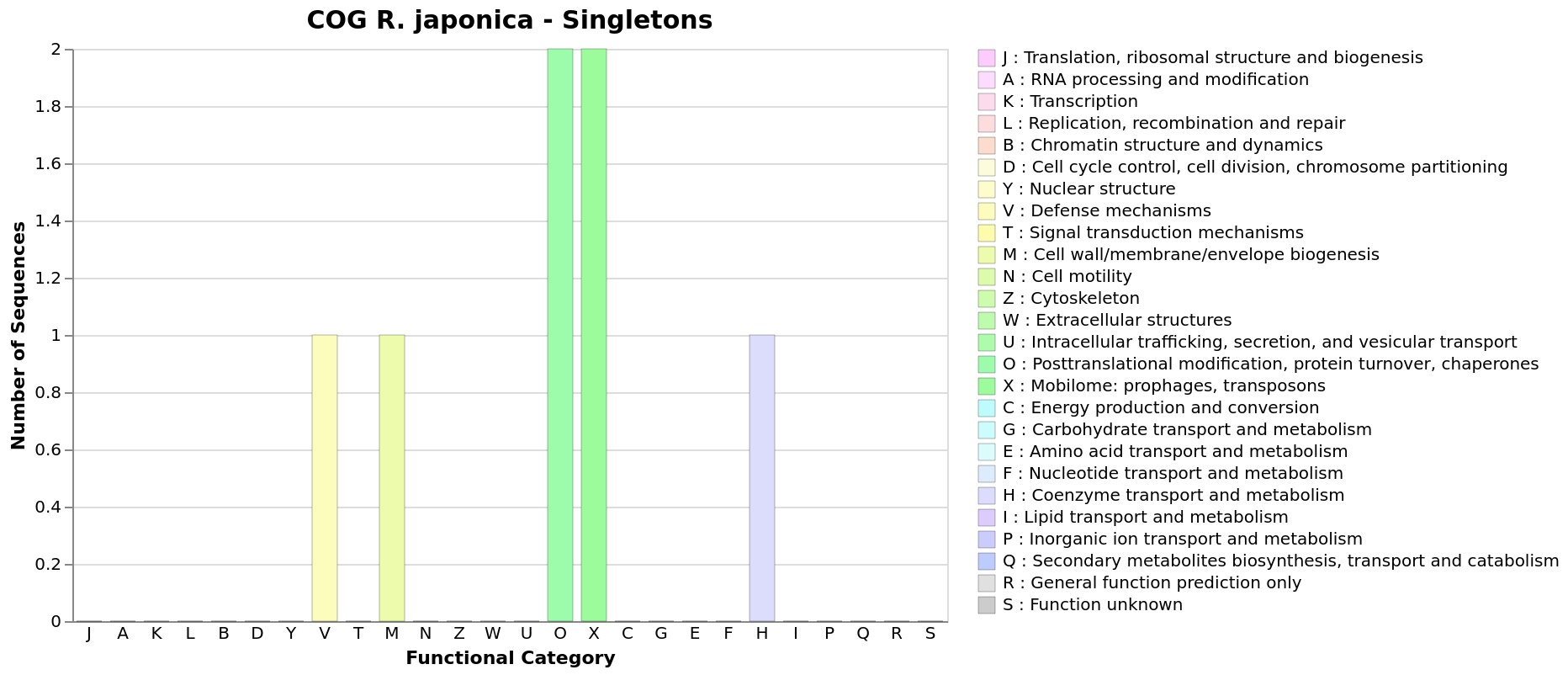

Supplement: Supplementary file 40 — Supplementary Material 40 (PNG 193 KB) [file 42770_2026_2030_MOESM40_ESM.png]

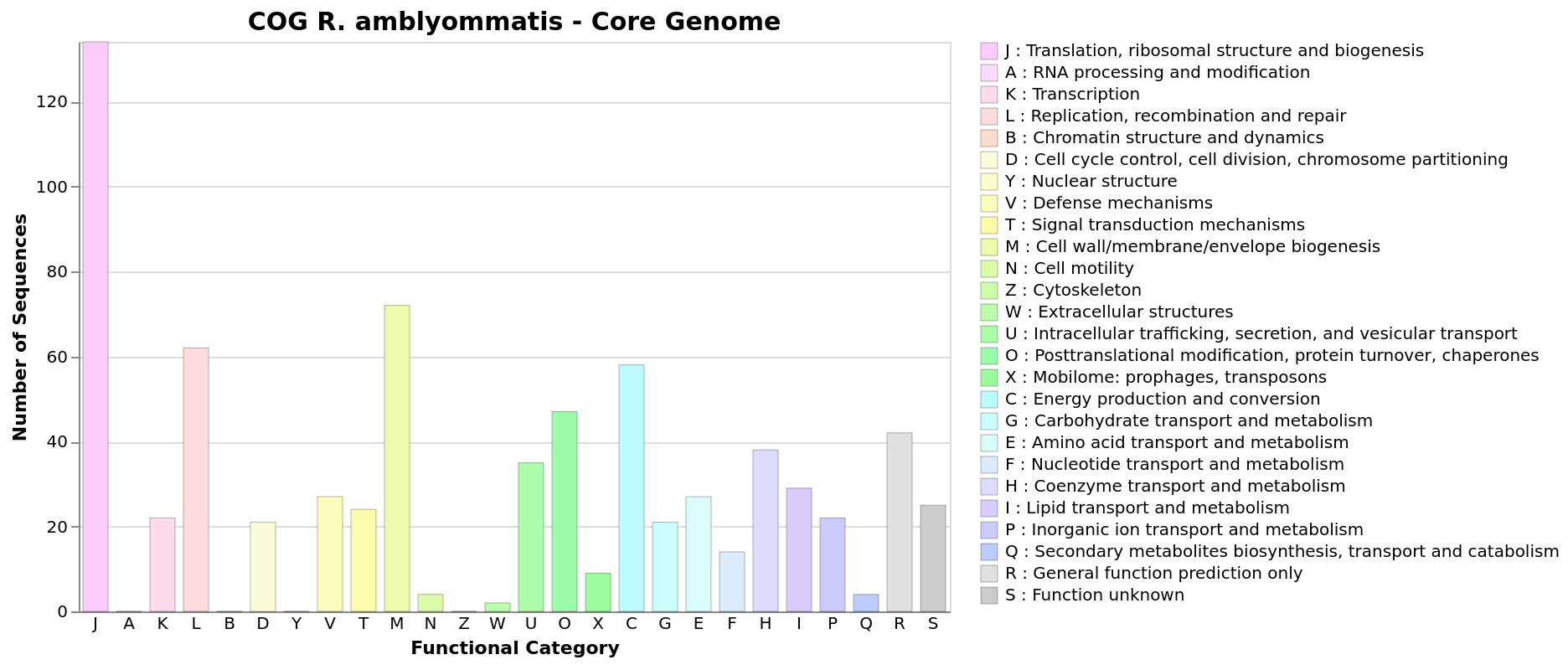

Supplement: Supplementary file 41 — Supplementary Material 41 (PNG 194 KB) [file 42770_2026_2030_MOESM41_ESM.png]

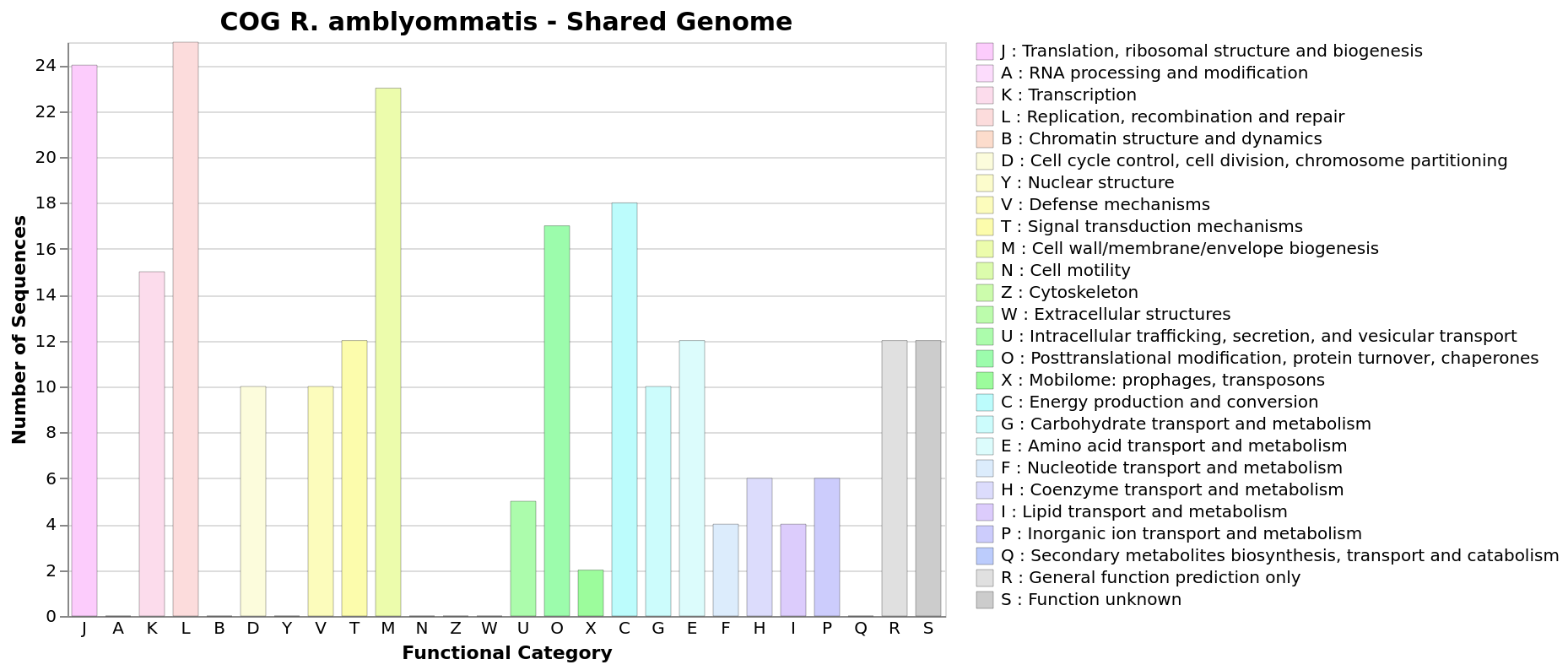

Supplement: Supplementary file 42 — Supplementary Material 42 (PNG 204 KB) [file 42770_2026_2030_MOESM42_ESM.png]

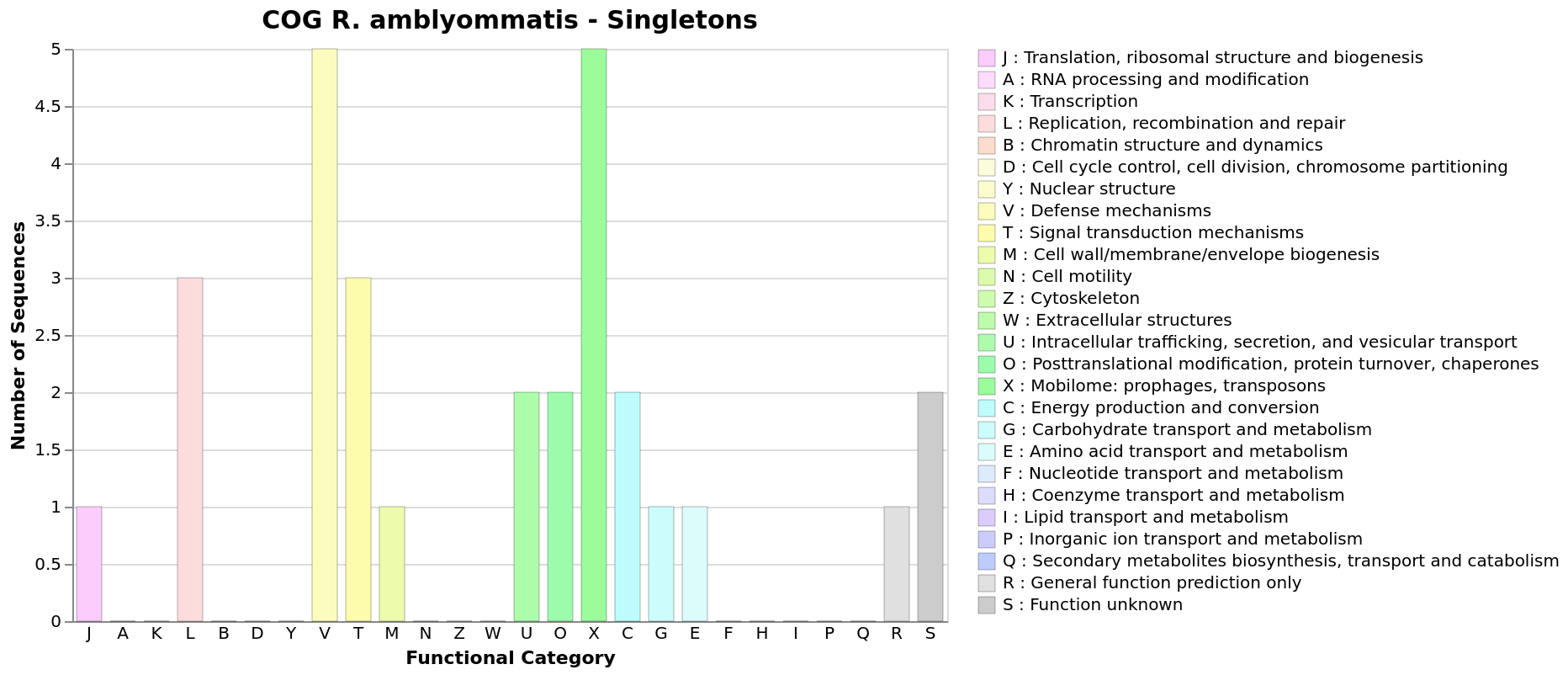

Supplement: Supplementary file 43 — Supplementary Material 43 (PNG 196 KB) [file 42770_2026_2030_MOESM43_ESM.png]

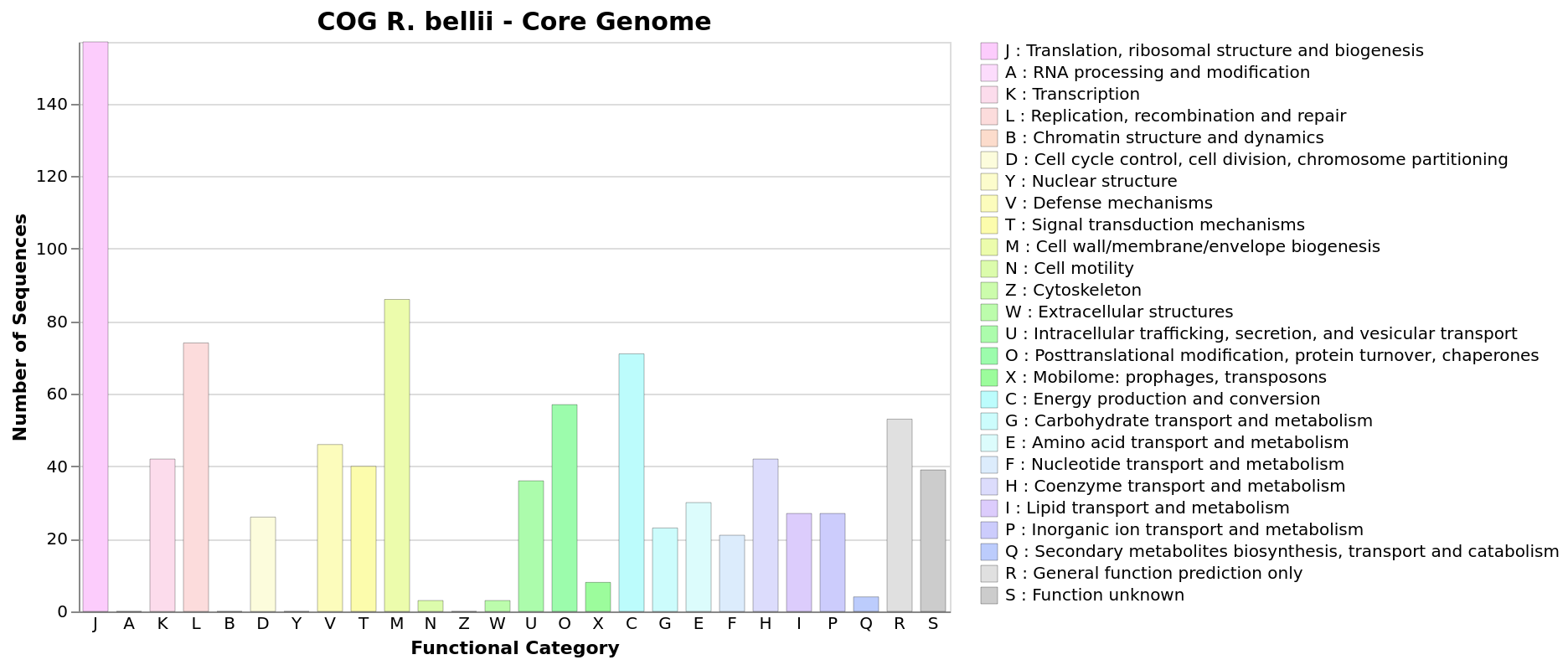

Supplement: Supplementary file 44 — Supplementary Material 44 (PNG 194 KB) [file 42770_2026_2030_MOESM44_ESM.png]

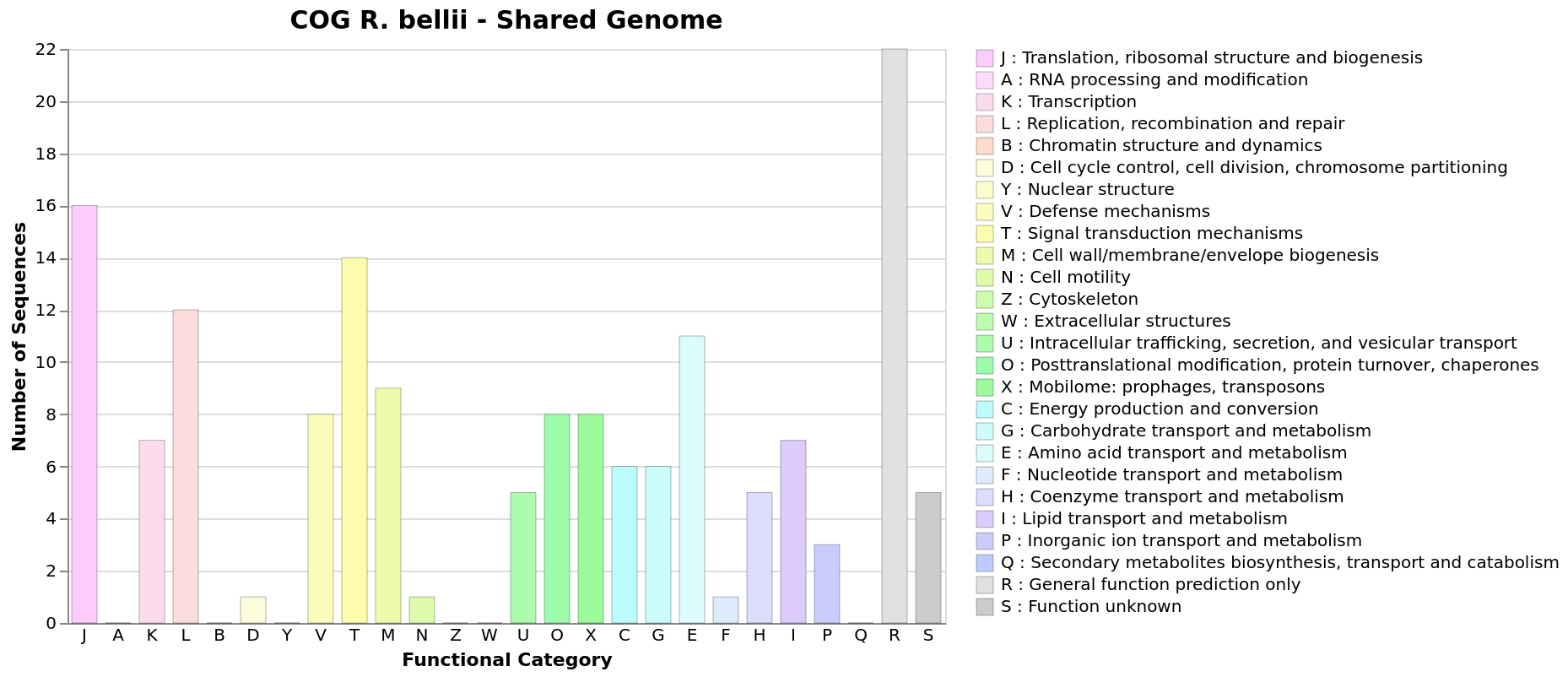

Supplement: Supplementary file 45 — Supplementary Material 45 (PNG 198 KB) [file 42770_2026_2030_MOESM45_ESM.png]

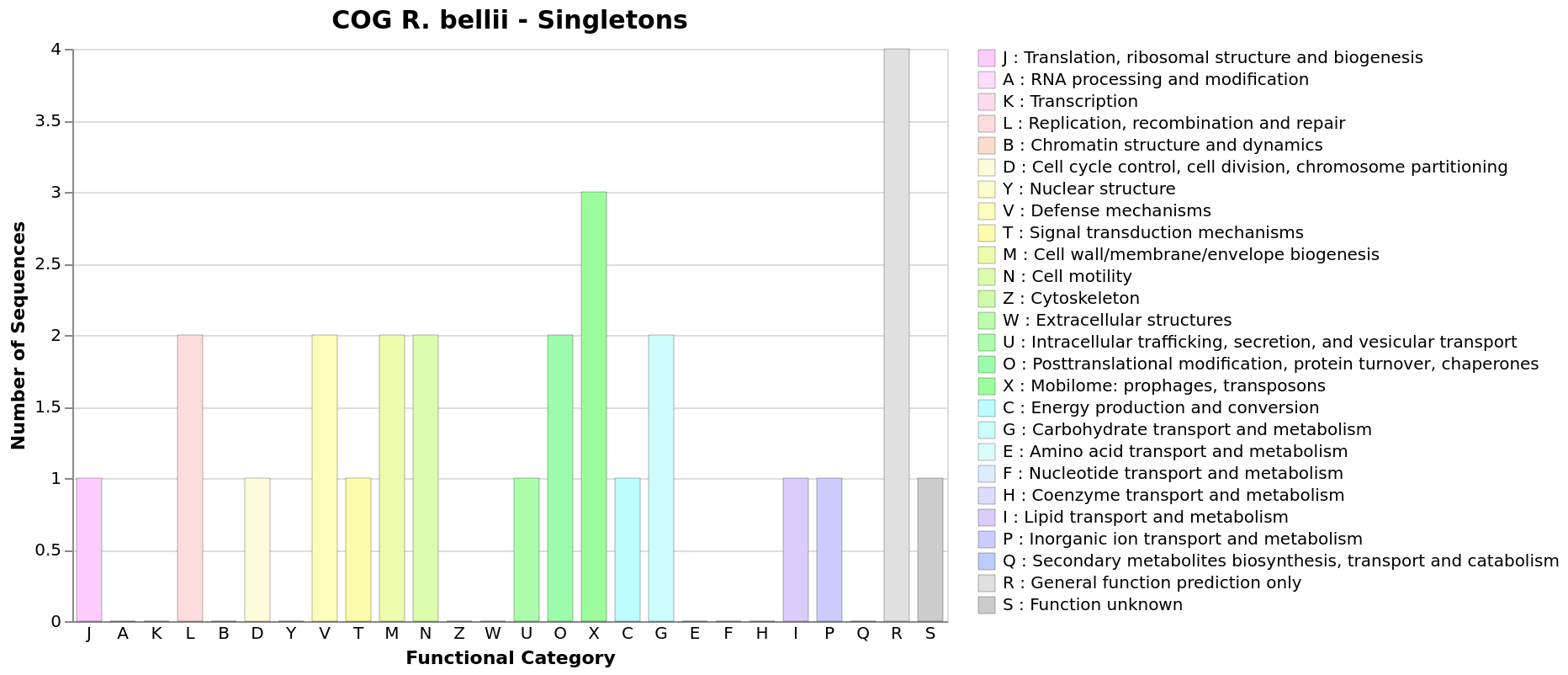

Supplement: Supplementary file 46 — Supplementary Material 46 (PNG 196 KB) [file 42770_2026_2030_MOESM46_ESM.png]

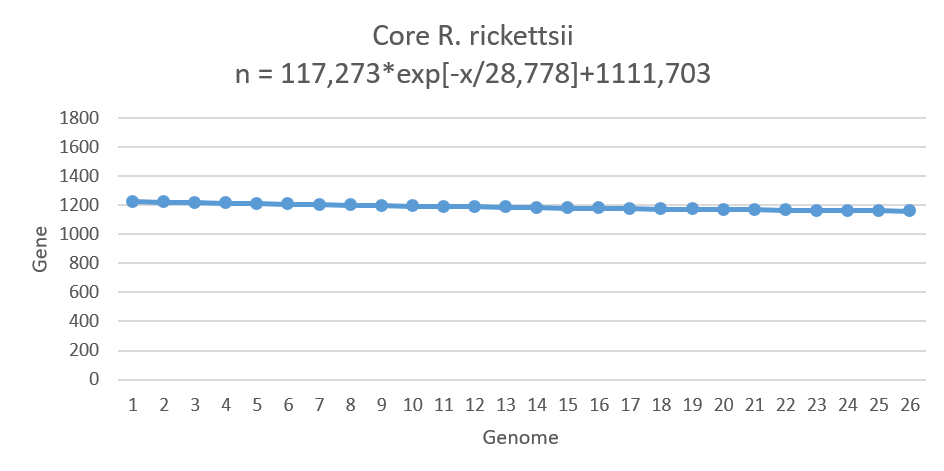

Supplement: Supplementary file 47 — Supplementary Material 47 (PNG 24.2 KB) [file 42770_2026_2030_MOESM47_ESM.png]

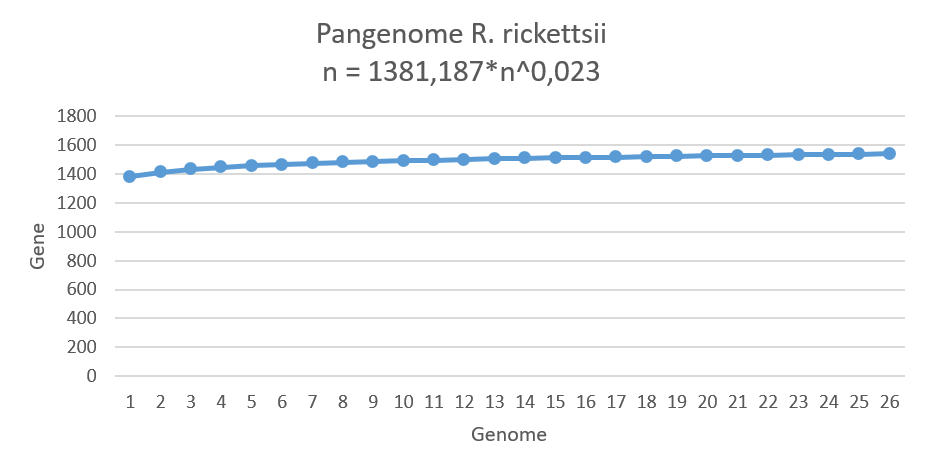

Supplement: Supplementary file 48 — Supplementary Material 48 (PNG 24.4 KB) [file 42770_2026_2030_MOESM48_ESM.png]

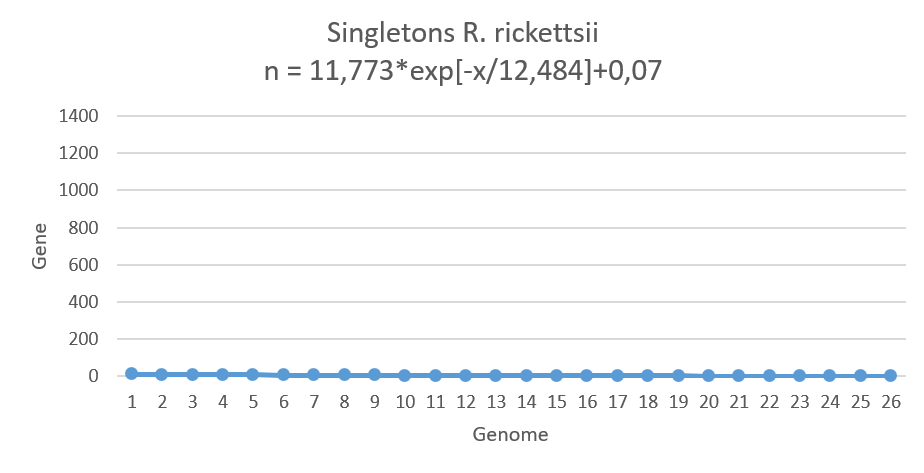

Supplement: Supplementary file 49 — Supplementary Material 49 (PNG 22.7 KB) [file 42770_2026_2030_MOESM49_ESM.png]

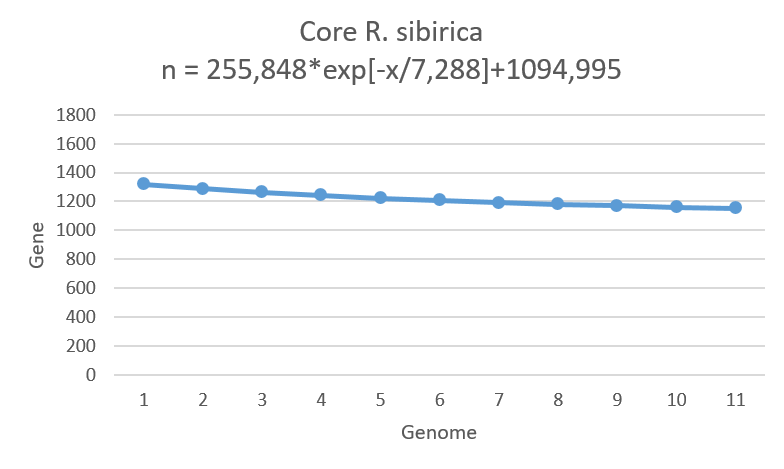

Supplement: Supplementary file 50 — Supplementary Material 50 (PNG 22.6 KB) [file 42770_2026_2030_MOESM50_ESM.png]

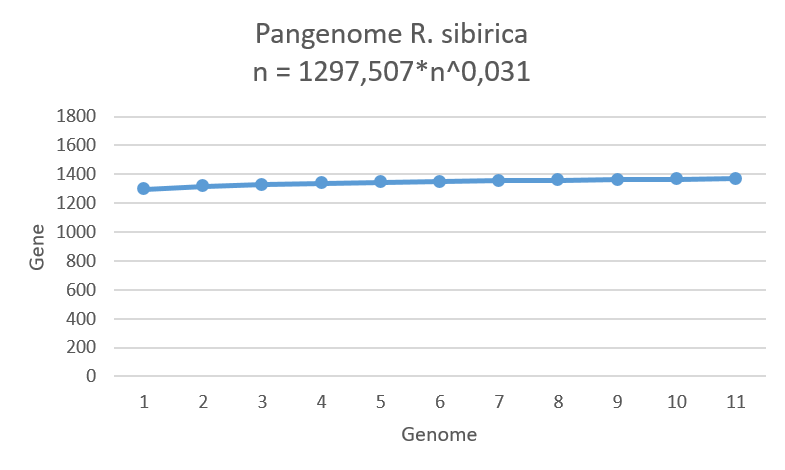

Supplement: Supplementary file 51 — Supplementary Material 51 (PNG 20.5 KB) [file 42770_2026_2030_MOESM51_ESM.png]

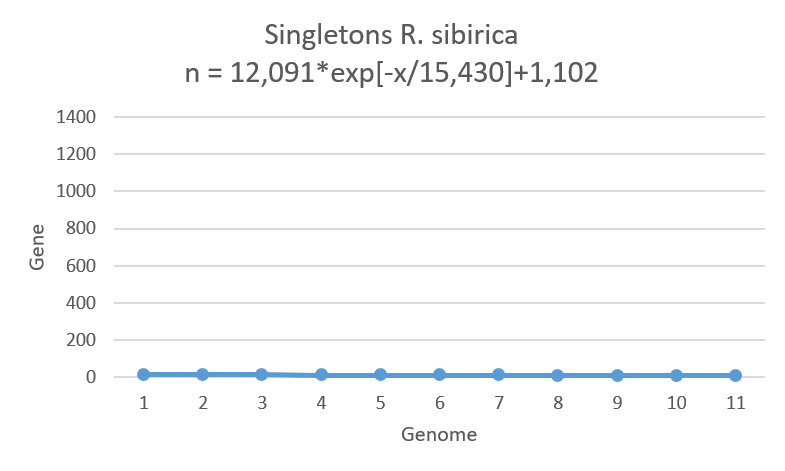

Supplement: Supplementary file 52 — Supplementary Material 52 (PNG 20.0 KB) [file 42770_2026_2030_MOESM52_ESM.png]

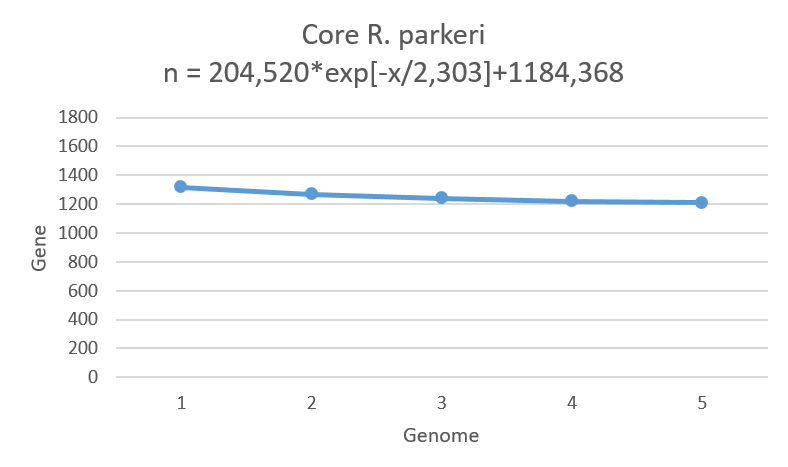

Supplement: Supplementary file 53 — Supplementary Material 53 (PNG 21.0 KB) [file 42770_2026_2030_MOESM53_ESM.png]

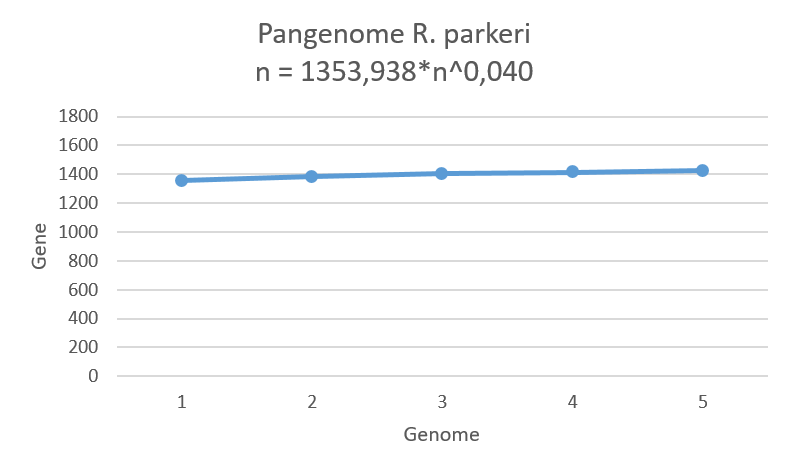

Supplement: Supplementary file 54 — Supplementary Material 54 (PNG 19.8 KB) [file 42770_2026_2030_MOESM54_ESM.png]

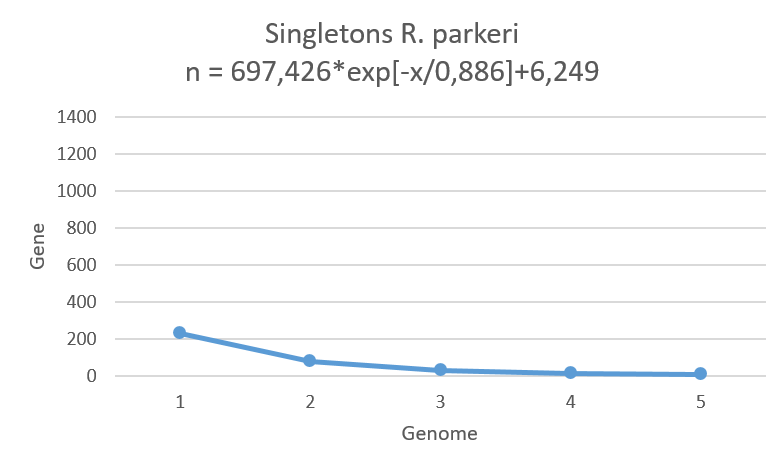

Supplement: Supplementary file 55 — Supplementary Material 55 (PNG 22.7 KB) [file 42770_2026_2030_MOESM55_ESM.png]

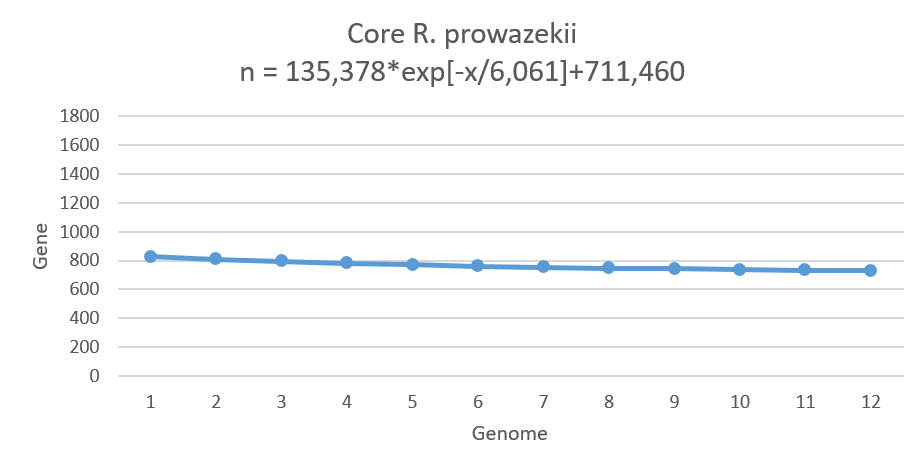

Supplement: Supplementary file 56 — Supplementary Material 56 (PNG 22.6 KB) [file 42770_2026_2030_MOESM56_ESM.png]

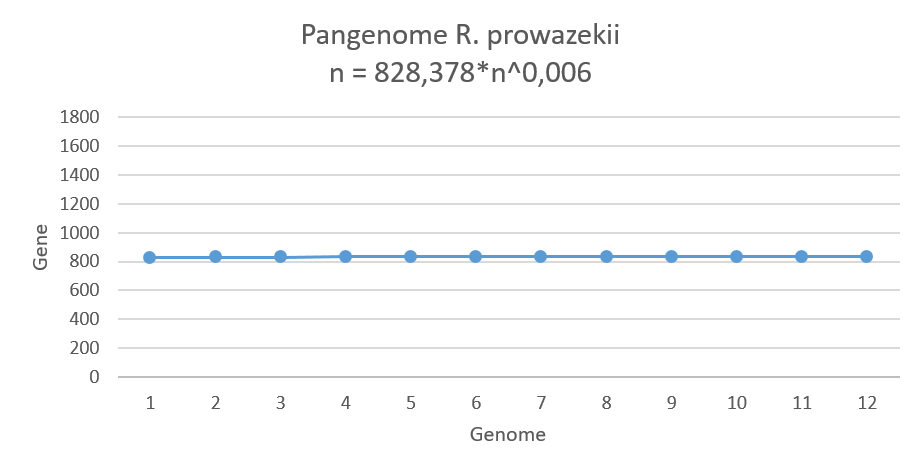

Supplement: Supplementary file 57 — Supplementary Material 57 (PNG 21.6 KB) [file 42770_2026_2030_MOESM57_ESM.png]

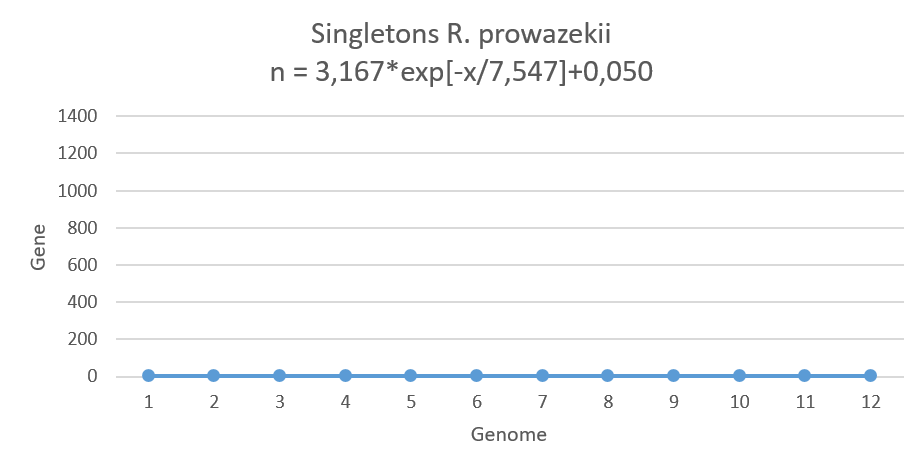

Supplement: Supplementary file 58 — Supplementary Material 58 (PNG 20.7 KB) [file 42770_2026_2030_MOESM58_ESM.png]

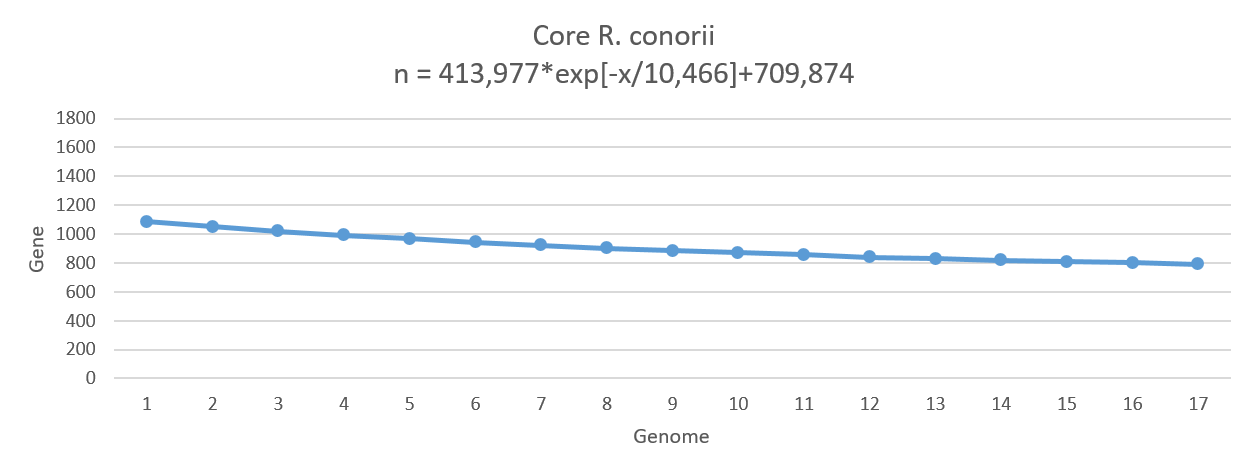

Supplement: Supplementary file 59 — Supplementary Material 59 (PNG 26.8 KB) [file 42770_2026_2030_MOESM59_ESM.png]

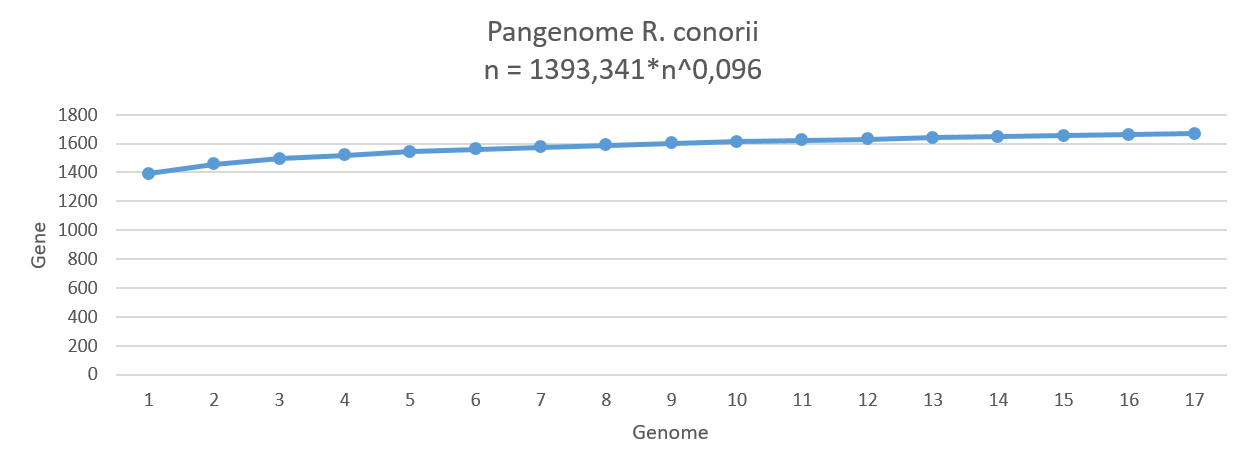

Supplement: Supplementary file 60 — Supplementary Material 60 (PNG 24.8 KB) [file 42770_2026_2030_MOESM60_ESM.png]

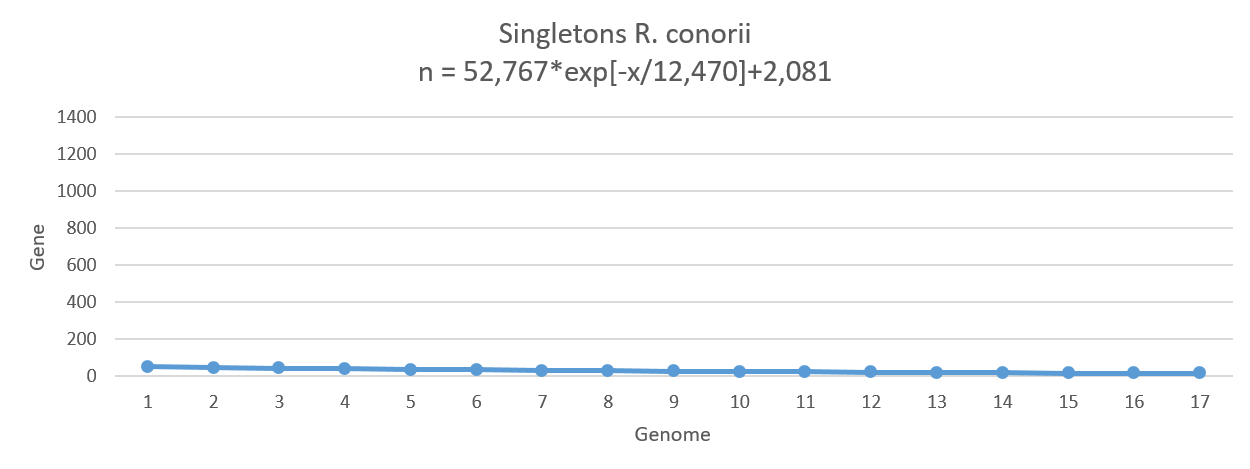

Supplement: Supplementary file 61 — Supplementary Material 61 (PNG 22.8 KB) [file 42770_2026_2030_MOESM61_ESM.png]

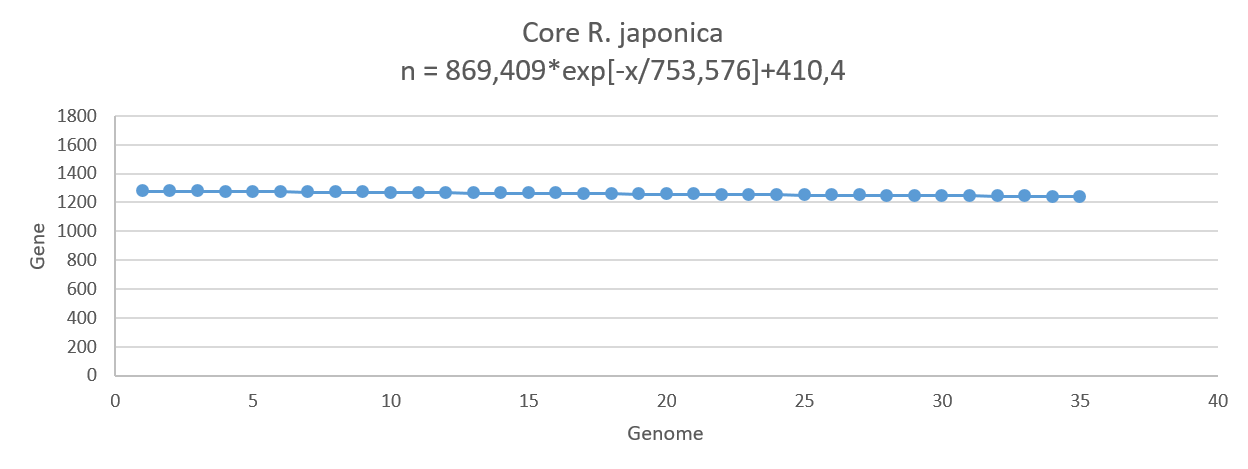

Supplement: Supplementary file 62 — Supplementary Material 62 (PNG 25.7 KB) [file 42770_2026_2030_MOESM62_ESM.png]

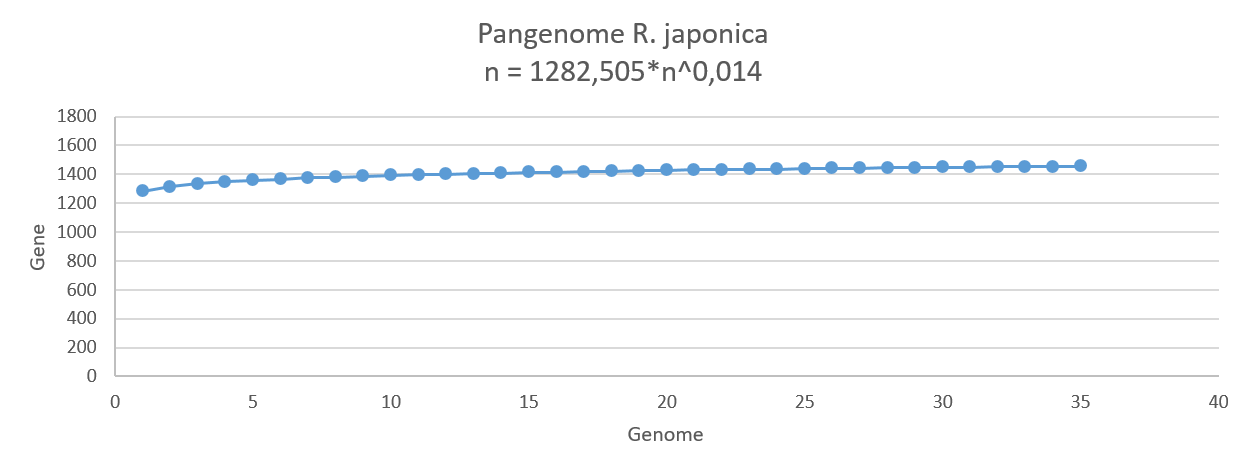

Supplement: Supplementary file 63 — Supplementary Material 63 (PNG 23.8 KB) [file 42770_2026_2030_MOESM63_ESM.png]

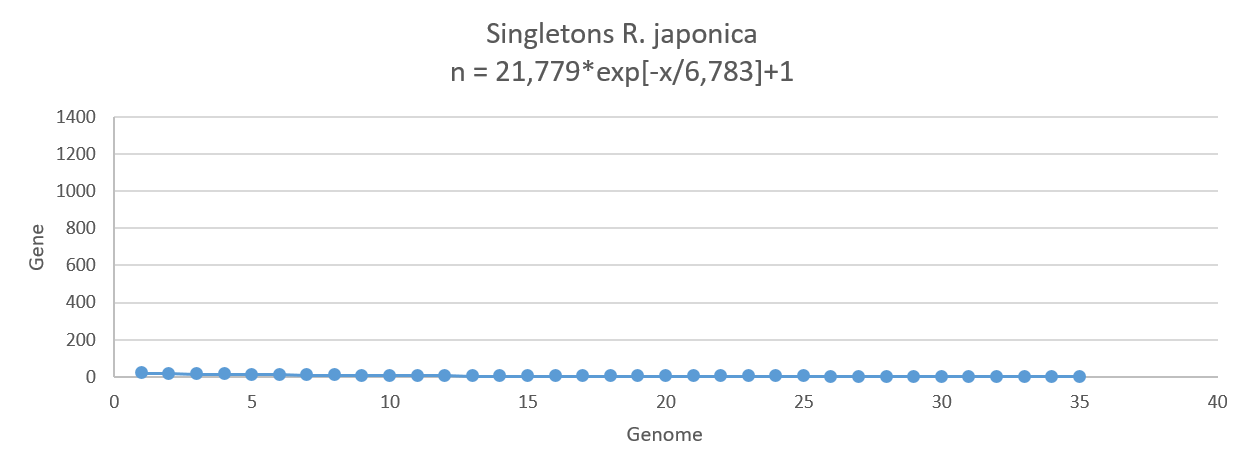

Supplement: Supplementary file 64 — Supplementary Material 64 (PNG 23.1 KB) [file 42770_2026_2030_MOESM64_ESM.png]
