# Supplementary material for: Pangenomic and genomic plasticity analyses of the genus Rickettsia
Source: Braz J Microbiol. 2026 Jul 24;57(1):217. doi: 10.1007/s42770-026-02030-7 (PMC13400510; doi:10.1007/s42770-026-02030-7)
Supplement: Supplementary file 65 — Supplementary Material 65 (DOCX 7.84 MB) [file 42770_2026_2030_MOESM65_ESM.docx]

# **SUPPLEMENTARY MATERIAL A** – COMPLETE COMPARATIVE ANALYSIS OF THE GENUS *RICKETTSIA*

#### Table 3 - Comparative Analysis of *Rickettsia* Species

| **Species** | **Subspecies** | **Strain** | **Genome size (MB)** | **GC (%)** | **Geographical location** | **Protein** | **Assembly** | **Group** |
| --- | --- | --- | --- | --- | --- | --- | --- | --- |
| *Rickettsia africae* | - | ESF-5 | 1,30 | 32,5 | Etiópia | 1343 | ASM2300v1 | Spotted Fever Group |
| *Rickettsia akari* | - | Hartford | 1,20 | 32,5 | - | 1124 | ASM1820v1 | Transitional Group |
| *Rickettsia amblyommatis* | - | Ac37 | 1,45 | 32,5 | Brazil: State Rondonia, Western Amazon | 1399 | ASM127379v1 | Spotted Fever Group |
| *Rickettsia amblyommatis* | - | An13 | 1,48 | 32,5 | Argentina: Dean Funes, Cordoba Province | 1433 | ASM207833v1 | Spotted Fever Group |
| *Rickettsia amblyommatis* | - | Ac/Pa | 1,43 | 32,5 | Panama | 1344 | ASM96467v1 | Spotted Fever Group |
| *Rickettsia amblyommatis* | - | Darkwater | 1,44 | 33,0 | USA | 1321 | ASM96499v1 | Spotted Fever Group |
| *Rickettsia amblyommatis* | - | GAT-30V | 1,50 | 32,5 | - | 1431 | ASM28405v1 | Spotted Fever Group |
| *Rickettsia argasii* | - | T170-B | 1,43 | 32,5 | USA | 1374 | ASM96518v1 | - |
| *Rickettsia asembonensis* | - | NMRCii | 1,37 | 32,0 | - | 1504 | ASM82812v2 | - |
| *Rickettsia asembonensis* | - | Perak | 1,42 | 32,0 | - | 1359 | ASM2857140v1 | - |
| *Rickettsia asiatica* | - | Maytaro1284 | 1,41 | 32,5 | - | 1436 | ASM798942v1 | - |
| *Rickettsia australis* | - | Cutlack | 1,32 | 32,5 | - | 1282 | ASM28415v1 | Spotted Fever Group |
| *Rickettsia australis* | - | Phillips | 1,32 | 32,5 | - | 1278 | Rau1.0 | Spotted Fever Group |
| *Rickettsia bellii* | - | An04 | 1,58 | 31,5 | Argentina: Dean Funes, Cordoba Province | 1491 | ASM207831v1 | Ancestral Group |
| *Rickettsia bellii* | - | OSU 85-389 | 1,53 | 31,5 | USA: Franklin Co., Ohio | 1465 | ASM1824v1 | Ancestral Group |
| *Rickettsia bellii* | - | RML369-C | 1,52 | 31,5 | - | 1439 | ASM1238v1 | Ancestral Group |
| *Rickettsia bellii* | - | RML An4 | 1,54 | 31,5 | Argentina | 1446 | ASM96500v1 | Ancestral Group |
| *Rickettsia bellii* | - | RML Mogi | 1,61 | 31,5 | Brazil | 1480 | ASM96504v1 | Ancestral Group |
| *Rickettsia canadensis* | - | CA410 | 1,15 | 31,0 | - | 916 | ASM28391v1 | Ancestral Group |
| *Rickettsia canadensis* | - | McKiel | 1,16 | 31,0 | - | 944 | ASM1434v1 | Ancestral Group |
| *Rickettsia conorii* | - | Malish 7 | 1,27 | 32,5 | - | 1339 | ASM702v1 | Spotted Fever Group |
| *Rickettsia conorii* | caspia | A-167 | 1,26 | 32,5 | - | 1274 | RcoCa1.0 | Spotted Fever Group |
| *Rickettsia conorii* | heilongjiangensis | Sendai-29 | 1,28 | 32,5 | - | 1323 | ASM973148v1 | Spotted Fever Group |
| *Rickettsia conorii* | heilongjiangensis | Sendai-58 | 1,28 | 32,5 | - | 1323 | ASM973150v1 | Spotted Fever Group |
| *Rickettsia conorii* | heilongjiangensis | HCN-13 | 1,28 | 32,5 | - | 1323 | ASM973152v1 | Spotted Fever Group |
| *Rickettsia conorii* | heilongjiangensis | CH8-1 | 1,28 | 32,5 | - | 1319 | ASM973154v1 | Spotted Fever Group |
| *Rickettsia conorii* | heilongjiangensis | B8 | 1,27 | 32,5 | China: Hanshan county, Anhui province | 1274 | ASM2641090v1 | Spotted Fever Group |
| *Rickettsia conorii* | heilongjiangensis | TIGMIC | 1,33 | 32,5 | China: Beijing | 1321 | ASM3511768v1 | Spotted Fever Group |
| *Rickettsia conorii* | heilongjiangensis | 054 | 1,28 | 32,5 | China: Heilongjiang province | 1319 | ASM22120v1 | Spotted Fever Group |
| *Rickettsia conorii* | indica | ITTR | 1,25 | 32,5 | India | 1237 | RcoIn1.0 | Spotted Fever Group |
| *Rickettsia conorii* | raoultii | Khabarovsk | 1,48 | 32,5 | - | 1505 | ASM94095v1 | Spotted Fever Group |
| *Rickettsia conorii* | raoultii | IM16 | 1,34 | 32,5 | China: Inner Mongolia | 1344 | ASM197518v1 | Spotted Fever Group |
| *Rickettsia conorii* | raoultii | BIME | 1,35 | 32,5 | China: Inner Mongolia | 1351 | ASM2367444v1 | Spotted Fever Group |
| *Rickettsia conorii* | raoultii | XinjiangF1 | 1,27 | 32,5 | China: Xinjiang | 1279 | ASM3385453v1 | Spotted Fever Group |
| *Rickettsia conorii* | raoultii | XinjiangF2 | 1,27 | 32,5 | China: Xinjiang | 1286 | ASM3385455v1 | Spotted Fever Group |
| *Rickettsia conorii* | raoultii | XinjiangF3 | 1,27 | 32,5 | China: Xinjiang | 1281 | ASM3385457v1 | Spotted Fever Group |
| *Rickettsia conorii* | raoultii | XinjiangM1 | 1,26 | 32,5 | China: Xinjiang | 1266 | ASM3385459v1 | Spotted Fever Group |
| *Rickettsia felis* | - | LSU-Lb | 1,58 | 32,5 | - | 1395 | ASM80450v1 | Transitional Group |
| *Rickettsia felis* | - | LSU | 1,55 | 32,5 | - | 1380 | ASM80452v1 | Transitional Group |
| *Rickettsia felis* | - | Pedreira | 1,49 | 32,5 | Brazil | 1352 | ASM96466v1 | Transitional Group |
| *Rickettsia fournieri* | - | AUS118 | 1,44 | 32,5 | - | 1391 | PRJEB23962 | Spotted Fever Group |
| *Rickettsia gravesii* | - | BWI-1 | 1,35 | 32,0 | - | 1372 | RicGra1.0 | - |
| *Rickettsia helvetica* | - | CC_DK | 1,42 | 32,0 | - | 1442 | DK2_Rh | Spotted Fever Group |
| *Rickettsia helvetica* | - | OB144 | 1,42 | 32,0 | - | 1442 | OB144_Rh | Spotted Fever Group |
| *Rickettsia helvetica* | - | C9P9 | 1,41 | 32,0 | Switzerland | 1386 | ASM25535v1 | Spotted Fever Group |
| *Rickettsia honei* | - | RB | 1,27 | 32,5 | - | 1318 | Rho1.0 | Spotted Fever Group |
| *Rickettsia hoogstraalii* | - | Croatica | 1,48 | 32,5 | - | 1544 | Rickettsia hoogstraalii Croatica | Spotted Fever Group |
| *Rickettsia hoogstraalii* | - | CS | 1,57 | 32,5 | China: Yunnan | 1479 | ASM2630929v1 | Spotted Fever Group |
| *Rickettsia japonica* | - | YH_M | 1,28 | 32,5 | - | 1305 | ASM235669v1 | Spotted Fever Group |
| *Rickettsia japonica* | - | SR1567 | 1,28 | 32,5 | - | 1303 | ASM235671v1 | Spotted Fever Group |
| *Rickettsia japonica* | - | M99123 | 1,28 | 32,5 | - | 1303 | ASM235673v1 | Spotted Fever Group |
| *Rickettsia japonica* | - | M99023 | 1,28 | 32,5 | - | 1306 | ASM235675v1 | Spotted Fever Group |
| *Rickettsia japonica* | - | M99015 | 1,28 | 32,5 | - | 1305 | ASM235677v1 | Spotted Fever Group |
| *Rickettsia japonica* | - | M14024 | 1,28 | 32,5 | - | 1316 | ASM235679v1 | Spotted Fever Group |
| *Rickettsia japonica* | - | M14012 | 1,28 | 32,5 | - | 1306 | ASM235681v1 | Spotted Fever Group |
| *Rickettsia japonica* | - | M13010 | 1,28 | 32,5 | - | 1303 | ASM235683v1 | Spotted Fever Group |
| *Rickettsia japonica* | - | M11012 | 1,28 | 32,5 | - | 1303 | ASM235685v1 | Spotted Fever Group |
| *Rickettsia japonica* | - | M08024 | 1,28 | 32,5 | - | 1302 | ASM235687v1 | Spotted Fever Group |
| *Rickettsia japonica* | - | M00021 | 1,28 | 32,5 | - | 1301 | ASM235689v1 | Spotted Fever Group |
| *Rickettsia japonica* | - | LON-151 | 1,28 | 32,5 | - | 1303 | ASM235691v1 | Spotted Fever Group |
| *Rickettsia japonica* | - | HH06125 | 1,28 | 32,5 | - | 1301 | ASM235693v1 | Spotted Fever Group |
| *Rickettsia japonica* | - | HH06116 | 1,28 | 32,5 | - | 1301 | ASM235695v1 | Spotted Fever Group |
| *Rickettsia japonica* | - | HH-18 | 1,28 | 32,5 | - | 1301 | ASM235697v1 | Spotted Fever Group |
| *Rickettsia japonica* | - | HH-17 | 1,28 | 32,5 | - | 1301 | ASM235699v1 | Spotted Fever Group |
| *Rickettsia japonica* | - | HH-16 | 1,28 | 32,5 | - | 1303 | ASM235701v1 | Spotted Fever Group |
| *Rickettsia japonica* | - | HH-13 | 1,28 | 32,5 | - | 1304 | ASM235703v1 | Spotted Fever Group |
| *Rickettsia japonica* | - | HH-12 | 1,28 | 32,5 | - | 1304 | ASM235705v1 | Spotted Fever Group |
| *Rickettsia japonica* | - | 3416 | 1,28 | 32,5 | - | 1303 | ASM235707v1 | Spotted Fever Group |
| *Rickettsia japonica* | - | 2763 | 1,28 | 32,5 | - | 1300 | ASM235709v1 | Spotted Fever Group |
| *Rickettsia japonica* | - | Tsuneishi | 1,28 | 32,5 | - | 1304 | ASM235711v1 | Spotted Fever Group |
| *Rickettsia japonica* | - | PO-1 | 1,28 | 32,5 | - | 1316 | ASM235713v1 | Spotted Fever Group |
| *Rickettsia japonica* | - | OHH-1 | 1,28 | 32,5 | - | 1305 | ASM235715v1 | Spotted Fever Group |
| *Rickettsia japonica* | - | Nakase | 1,28 | 32,5 | - | 1304 | ASM235717v1 | Spotted Fever Group |
| *Rickettsia japonica* | - | MZ08014 | 1,28 | 32,5 | - | 1303 | ASM235719v1 | Spotted Fever Group |
| *Rickettsia japonica* | - | HH07167 | 1,28 | 32,5 | - | 1304 | ASM235721v1 | Spotted Fever Group |
| *Rickettsia japonica* | - | HH07124 | 1,28 | 32,5 | - | 1304 | ASM235723v1 | Spotted Fever Group |
| *Rickettsia japonica* | - | HH06154 | 1,28 | 32,5 | - | 1303 | ASM235725v1 | Spotted Fever Group |
| *Rickettsia japonica* | - | HH-1 | 1,28 | 32,5 | - | 1316 | ASM235727v1 | Spotted Fever Group |
| *Rickettsia japonica* | - | DT-1 | 1,28 | 32,5 | - | 1305 | ASM235729v1 | Spotted Fever Group |
| *Rickettsia japonica* | - | LA4/2015 | 1,28 | 32,5 | China: Zhejiang Provinc | 1329 | ASM345471v1 | Spotted Fever Group |
| *Rickettsia japonica* | - | LA16/2015 | 1,28 | 32,5 | China: Zhejiang | 1312 | ASM985799v1 | Spotted Fever Group |
| *Rickettsia japonica* | - | YH | 1,28 | 32,5 | - | 1313 | ASM28359v1 | Spotted Fever Group |
| *Rickettsia japonica* | - | YH (contig00039) | 1,27 | 32,5 | - | 1294 | ASM30263v2 | Spotted Fever Group |
| *Rickettsia massiliae* | - | MTU5 | 1,38 | 32,5 | - | 1370 | ASM1662v1 | Spotted Fever Group |
| *Rickettsia massiliae* | - | AZT80 | 1,28 | 32,5 | - | 1259 | ASM28385v1 | Spotted Fever Group |
| *Rickettsia monacensis* | - | IrR/Munich | 1,35 | 32,5 | - | 1298 | RMONA_1 | Spotted Fever Group |
| *Rickettsia monacensis* | - | OSU 85-930 | 1,28 | 32,5 | - | 1298 | ASM28417v1 | Spotted Fever Group |
| *Rickettsia parkeri* | - | Atlantic Rainforest | 1,35 | 32,5 | Colombia: Necocli | 1394 | ASM554911v1 | Spotted Fever Group |
| *Rickettsia parkeri* | - | AT#24 | 1,30 | 32,5 | Brazil | 1340 | ASM96507v1 | Spotted Fever Group |
| *Rickettsia parkeri* | - | Grand Bay | 1,31 | 32,5 | USA | 1344 | ASM96508v1 | Spotted Fever Group |
| *Rickettsia parkeri* | - | Portsmouth | 1,30 | 32,5 | - | 1354 | ASM28419v1 | Spotted Fever Group |
| *Rickettsia parkeri* | - | Tate's Hell | 1,30 | 32,5 | USA | 1343 | ASM96514v1 | Spotted Fever Group |
| *Rickettsia peacockii* | - | Rustic | 1,31 | 32,5 | - | 1313 | ASM2152v1 | Spotted Fever Group |
| *Rickettsia philipii* | - | 364D | 1,29 | 32,5 | - | 1328 | ASM28399v1 | Spotted Fever Group |
| *Rickettsia prowazekii* | - | Naples-1 | 1,11 | 29,0 | - | 831 | ASM160221v1 | Typhus Group |
| *Rickettsia prowazekii* | - | Breinl | 1,11 | 29,0 | - | 818 | ASM36740v1 | Typhus Group |
| *Rickettsia prowazekii* | - | BuV67-CWPP | 1,11 | 29,0 | - | 836 | ASM27720v1 | Typhus Group |
| *Rickettsia prowazekii* | - | Cairo 3 | 1,11 | 29,0 | - | 828 | P_2008_03_27_11_29_25_runAssembly | Typhus Group |
| *Rickettsia prowazekii* | - | Chernikova | 1,11 | 29,0 | - | 836 | ASM27716v1 | Typhus Group |
| *Rickettsia prowazekii* | - | Dachau | 1,11 | 29,0 | - | 815 | ASM27722v1 | Typhus Group |
| *Rickettsia prowazekii* | - | GvV257 | 1,11 | 29,0 | - | 820 | ASM27724v1 | Typhus Group |
| *Rickettsia prowazekii* | - | Katsinyian | 1,11 | 29,0 | - | 836 | ASM27718v1 | Typhus Group |
| *Rickettsia prowazekii* | - | Madrid E | 1,11 | 29,0 | - | 822 | ASM19573v1 | Typhus Group |
| *Rickettsia prowazekii* | - | NMRC Madrid E | 1,11 | 29,0 | - | 795 | ASM36390v1 | Typhus Group |
| *Rickettsia prowazekii* | - | Rp22 | 1,11 | 29,0 | - | 832 | ASM2278v1 | Typhus Group |
| *Rickettsia prowazekii* | - | RpGvF24 | 1,11 | 29,0 | - | 829 | ASM27726v1 | Typhus Group |
| *Rickettsia rhipicephali* | - | HJ#5 | 1,45 | 32,5 | - | 1405 | ASM144247v1 | Spotted Fever Group |
| *Rickettsia rhipicephali* | - | EH | 1,39 | 32,5 | China: Yunnan | 1403 | ASM2630931v1 | Spotted Fever Group |
| *Rickettsia rhipicephali* | - | 3-7-female6-CWPP | 1,30 | 32,5 | - | 1278 | ASM28407v1 | Spotted Fever Group |
| *Rickettsia rhipicephali* | - | Ect | 1,27 | 32,5 | USA | 1263 | ASM96490v1 | Spotted Fever Group |
| *Rickettsia rickettsii* | large clone | Iowa | 1,27 | 32,5 | - | 1310 | ASM195099v1 | Spotted Fever Group |
| *Rickettsia rickettsii* | small clone | Iowa | 1,27 | 32,5 | - | 1310 | ASM195101v1 | Spotted Fever Group |
| *Rickettsia rickettsii* | - | AZ-5 | 1,27 | 32,5 | USA: Arizona | 1336 | ASM2371674v1 | Spotted Fever Group |
| *Rickettsia rickettsii* | - | Sao Paulo | 1,26 | 32,5 | Brazil | 1330 | ASM2371676v1 | Spotted Fever Group |
| *Rickettsia rickettsii* | - | Taiacu | 1,26 | 32,5 | Brazil | 1331 | ASM2371678v1 | Spotted Fever Group |
| *Rickettsia rickettsii* | - | RMSFvaccine | 1,26 | 32,5 | USA: Hamilton, Montana | 1300 | ASM2725297v2 | Spotted Fever Group |
| *Rickettsia rickettsii* | - | Cache Creek | 1,29 | 32,5 | - | 1349 | ASM4392913v1 | Spotted Fever Group |
| *Rickettsia rickettsii* | - | Wright Peak | 1,29 | 32,5 | - | 1334 | ASM4403869v1 | Spotted Fever Group |
| *Rickettsia rickettsii* | - | Pierce Canyon | 1,29 | 32,5 | - | 1352 | ASM4403870v1 | Spotted Fever Group |
| *Rickettsia rickettsii* | - | Highland Creek | 1,29 | 32,5 | - | 1357 | ASM4405816v1 | Spotted Fever Group |
| *Rickettsia rickettsii* | - | Pine Mountain | 1,29 | 32,5 | - | 1351 | ASM4405817v1 | Spotted Fever Group |
| *Rickettsia rickettsii* | - | Orange | 1,29 | 32,5 | - | 1344 | ASM4405818v1 | Spotted Fever Group |
| *Rickettsia rickettsii* | - | Mt. Konocti | 1,29 | 32,5 | - | 1335 | ASM4405881v1 | Spotted Fever Group |
| *Rickettsia rickettsii* | - | Lake | 1,29 | 32,5 | - | 1351 | ASM4405882v1 | Spotted Fever Group |
| *Rickettsia rickettsii* | - | El Moro Canyon | 1,29 | 32,5 | - | 1348 | ASM4405883v1 | Spotted Fever Group |
| *Rickettsia rickettsii* | - | Crystal Cove | 1,29 | 32,5 | - | 1348 | ASM4405912v1 | Spotted Fever Group |
| *Rickettsia rickettsii* | - | Sheila Smith | 1,26 | 32,5 | - | 1308 | ASM2990973v1 | Spotted Fever Group |
| *Rickettsia rickettsii* | - | Arizona | 1,27 | 32,5 | - | 1324 | ASM28379v1 | Spotted Fever Group |
| *Rickettsia rickettsii* | - | Brazil | 1,26 | 32,5 | - | 1305 | ASM28395v1 | Spotted Fever Group |
| *Rickettsia rickettsii* | - | Colombia | 1,27 | 32,5 | - | 1318 | ASM28377v1 | Spotted Fever Group |
| *Rickettsia rickettsii* | - | Hauke | 1,27 | 32,5 | - | 1325 | ASM28383v1 | Spotted Fever Group |
| *Rickettsia rickettsii* | - | Hino | 1,27 | 32,5 | - | 1329 | ASM28381v1 | Spotted Fever Group |
| *Rickettsia rickettsii* | - | Hlp#2 | 1,27 | 32,5 | - | 1306 | ASM28393v1 | Spotted Fever Group |
| *Rickettsia rickettsii* | - | Iowa | 1,27 | 32,5 | - | 1310 | ASM1744v3 | Spotted Fever Group |
| *Rickettsia rickettsii* | - | Morgan | 1,27 | 32,5 | - | 1325 | ASM83154v1 | Spotted Fever Group |
| *Rickettsia rickettsii* | - | R | 1,26 | 32,5 | - | 1304 | ASM83152v1 | Spotted Fever Group |
| *Rickettsia sibirica* | - | FK58 | 1,26 | 32,5 | China: Ninghua, FuJian | 1341 | ASM4275737v1 | - |
| *Rickettsia sibirica* | - | O53 | 1,28 | 32,5 | China: Suifenhe, Heilongjiang | 1360 | ASM4275851v1 | - |
| *Rickettsia sibirica* | - | TO85 | 1,26 | 32,5 | China: Hulunbur, Inner Mongolia | 1335 | ASM4275906v1 | - |
| *Rickettsia sibirica* | - | AN | 1,26 | 32,5 | China: Jinghe, Xinjiang | 1332 | ASM4275965v1 | - |
| *Rickettsia sibirica* | - | Se | 1,26 | 32,5 | China: Hulunbur, Inner Mongolia | 1344 | ASM4276047v1 | - |
| *Rickettsia sibirica* | - | JHT | 1,28 | 32,5 | China: Jinghe, Xinjiang | 1358 | ASM4276126v1 | - |
| *Rickettsia sibirica* | - | MT84 | 1,26 | 32,5 | China: Jinghe, Xinjiang | 1333 | ASM4276159v1 | - |
| *Rickettsia sibirica* | - | SFGW | 1,26 | 32,5 | China: Tongliao, Inner Mongolia | 1330 | ASM4534881v1 | - |
| *Rickettsia sibirica* | - | 246 | 1,25 | 32,5 | Russia: Krasnojarsk | 1318 | ASM16693v1 | - |
| *Rickettsia sibirica* | mongolitimonae | HA-91 | 1,25 | 32,5 | China: Alashian region of the Inner Mongolia autonomous region | 1265 | ASM24762v2 | - |
| *Rickettsia sibirica* | sibirica | BJ-90 | 1,25 | 32,5 | China: Beijing | 1291 | ASM24671v2 | - |
| *Rickettsia sibirica* | sibirica | BJ-90 | 1,26 | 32,5 | China: Changping, Beijing | 1336 | ASM4276020v1 | - |
| *Rickettsia slovaca* | - | 13-B | 1,27 | 32,5 |  | 1359 | ASM23784v1 | Spotted Fever Group |
| *Rickettsia slovaca* | - | D-CWPP | 1,27 | 32,5 |  | 1361 | ASM25236v1 | Spotted Fever Group |
| *Rickettsia sp.* | - | 2024-CO-Wats | 1,27 | 32,5 | USA: North Carolina | 1250 | NCSU_Rfin | - |
| *Rickettsia sp.* | - | MEAM1 (Bemisia tabaci) | 1,22 | 32,0 | China: Hangzhou | 1159 | ASM26522v2 | - |
| *Rickettsia sp.* | - | MEAM1 | 1,38 | 32,5 | USA: Charleston | 1405 | ASM228590v1 | - |
| *Rickettsia sp.* | - | Oklahoma-10 | 1,17 | 30,5 | - | 915 | NCSU_Roklah_1 | - |
| *Rickettsia sp.* | - | Tenjiku01 | 1,26 | 32,5 | - | 1277 | ASM165301v1 | - |
| *Rickettsia sp.* | - | TH2014 | 1,37 | 33,0 | Malaysia: Perak | 1287 | ASM1924919v1 | - |
| *Rickettsia sp.* | - | wb | 1,20 | 32,0 | Israel | 1160 | ASM170792v1 | - |
| *Rickettsia sp.* | - | wq | 1,20 | 32,0 | Israel | 1163 | ASM170780v1 | - |
| *Rickettsia tamurae* | - | AT-1 | 1,45 | 32,5 | - | 1513 | Rickettsia tamurae AT-1 | Spotted Fever Group |
| *Rickettsia tamurae* | buchneri | ISO7 | 1,65 | 32,5 | - | 1499 | REISMNv1 | Spotted Fever Group |
| *Rickettsia tamurae* | buchneri |  | 1,78 | 32,5 | USA: Oklahoma State University Tick Rearing Facility | 1683 | ASM2672368v1 | Spotted Fever Group |
| *Rickettsia tillamookensis* | - | Tillamook 23 | 1,44 | 32,5 | - | 1286 | ASM1674379v2 | Transitional Group |
| *Rickettsia typhi* | - | TM2540 | 1,11 | 29,0 | - | 808 | TM2540 | Typhus Group |
| *Rickettsia typhi* | - | B9991CWPP | 1,11 | 29,0 | - | 811 | ASM27730v1 | Typhus Group |
| *Rickettsia typhi* | - | TH1527 | 1,11 | 29,0 | - | 811 | ASM27728v1 | Typhus Group |
| *Rickettsia typhi* | - | Wilmington | 1,11 | 29,0 | - | 806 | ASM804v1 | Typhus Group |

Source: By author, 2025. The “-” sign represents species that have not yet been classified in the literature or those for which information was not available on the search engines used.

# **SUPPLEMENTARY MATERIAL B - PANGENOME DEVELOPMENT**

B1. Pangenome, core genome and singletons of *Rickettsia amblyommatis*

*
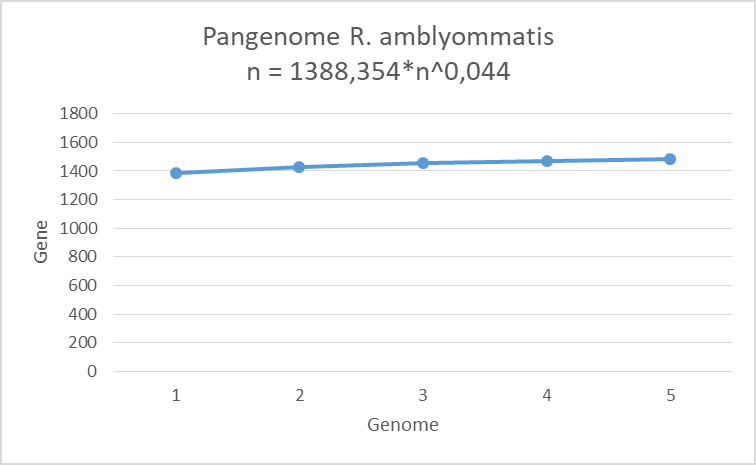

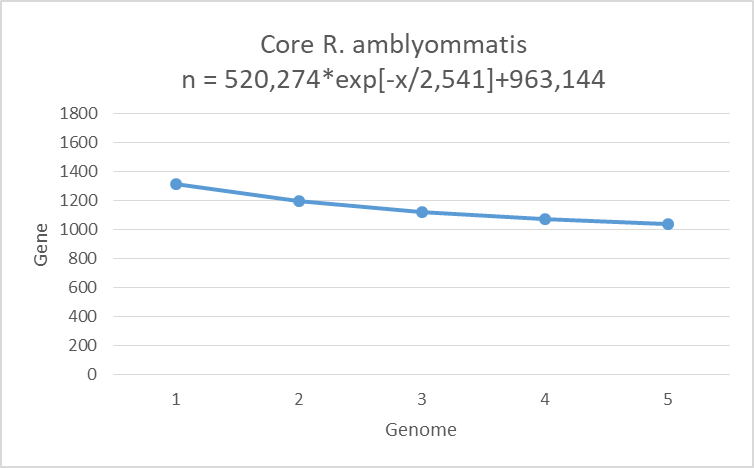
*

*
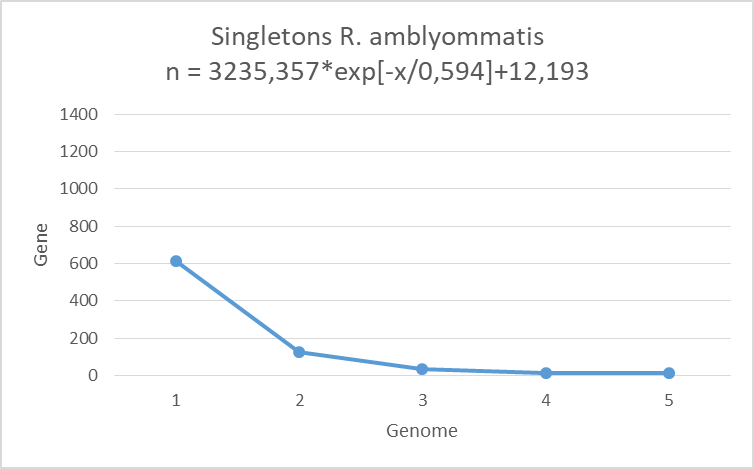
*

Source: By author, 2025. Note: Graphs representing the dynamics of the pangenome, core, and singletons among the analyzed genomes of *R. amblyommatis*. The fitted equations are shown in each graph, representing the regression model used to describe the data trend.

B2. Pangenome, core genome and singletons of *Rickettsia bellii*


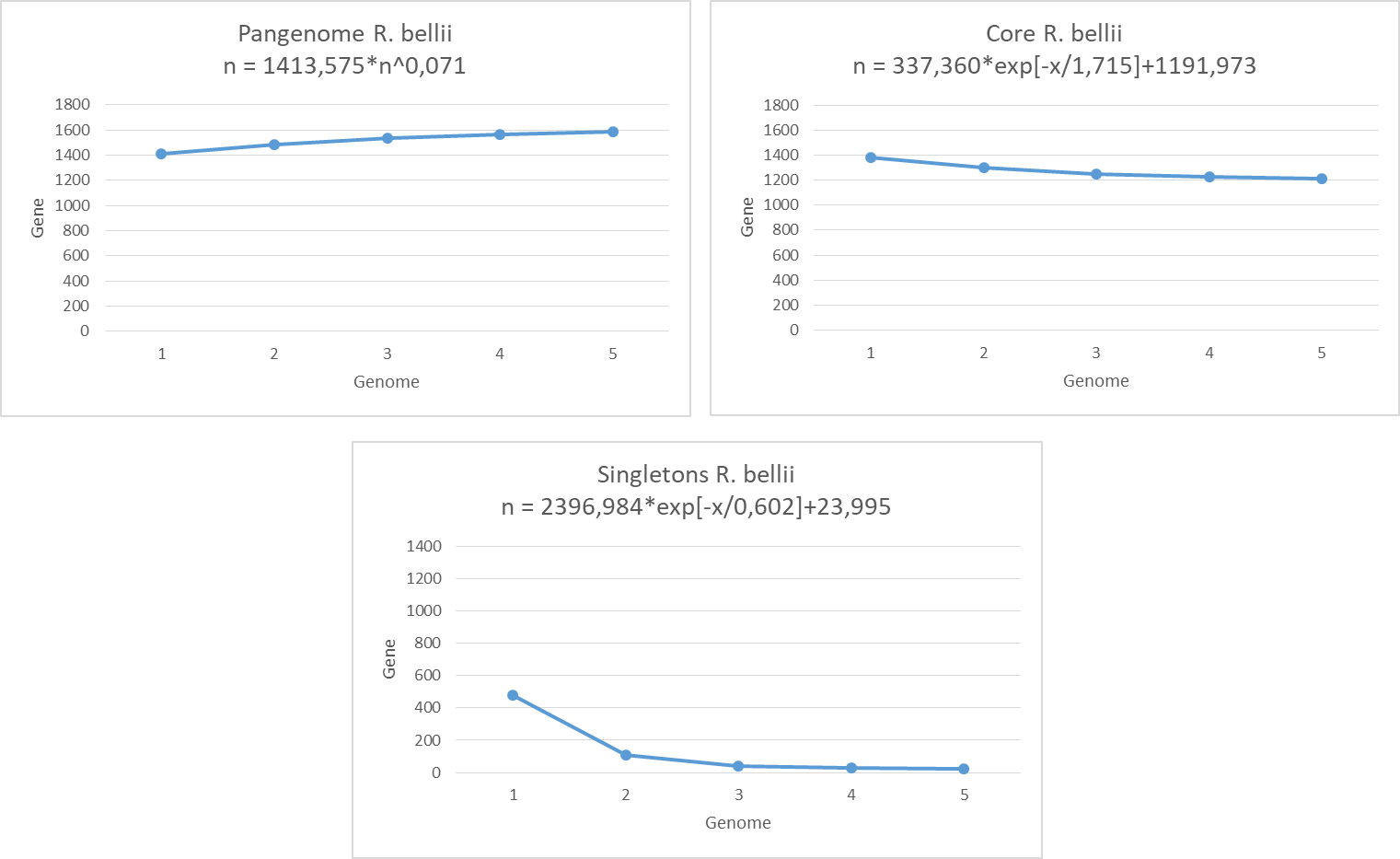


Source: By author, 2025. Note: Graphs representing the dynamics of the pangenome, core, and singletons among the analyzed genomes of *R. bellii*. The fitted equations are shown in each graph, representing the regression model used to describe the data trend.

B3. Pangenome, core genome and singletons of *Rickettsia conorii*


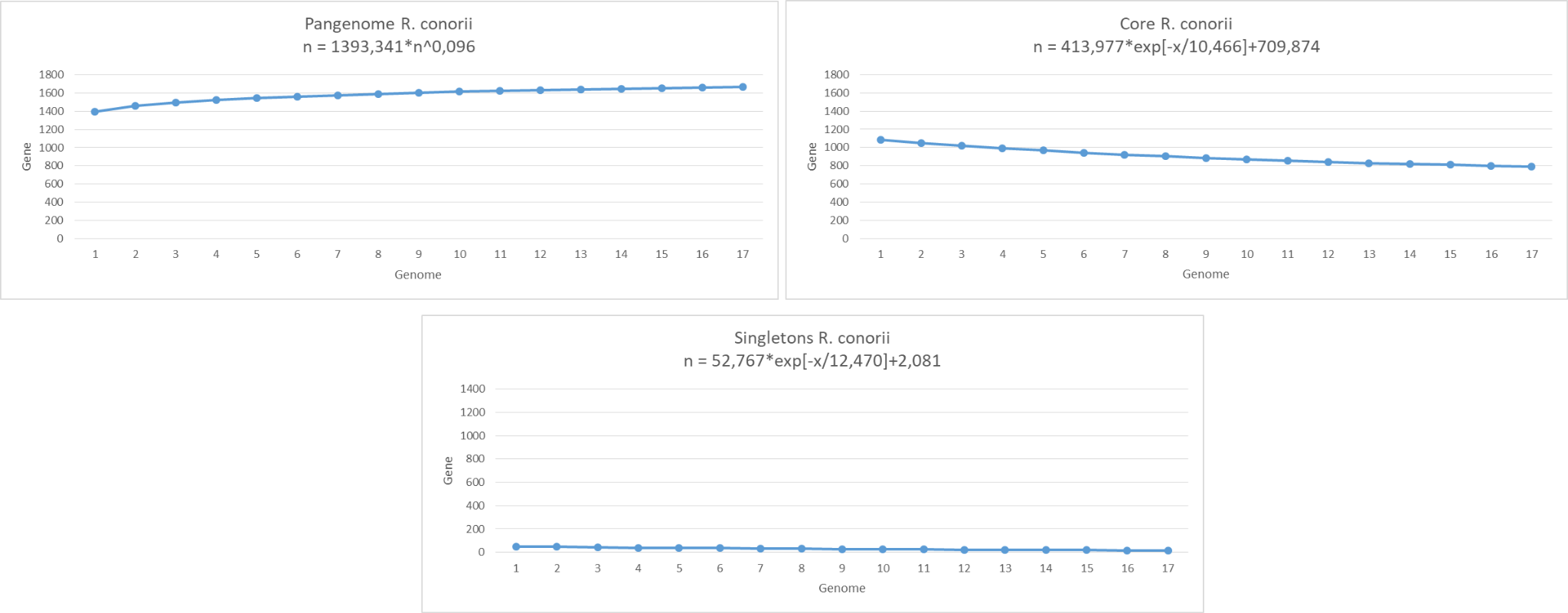


Source: By author, 2025. Note: Graphs representing the dynamics of the pangenome, core, and singletons among the analyzed genomes of *R. conorii*. The fitted equations are shown in each graph, representing the regression model used to describe the data trend.

B4. Pangenome, core genome and singletons of *Rickettsia japonica*


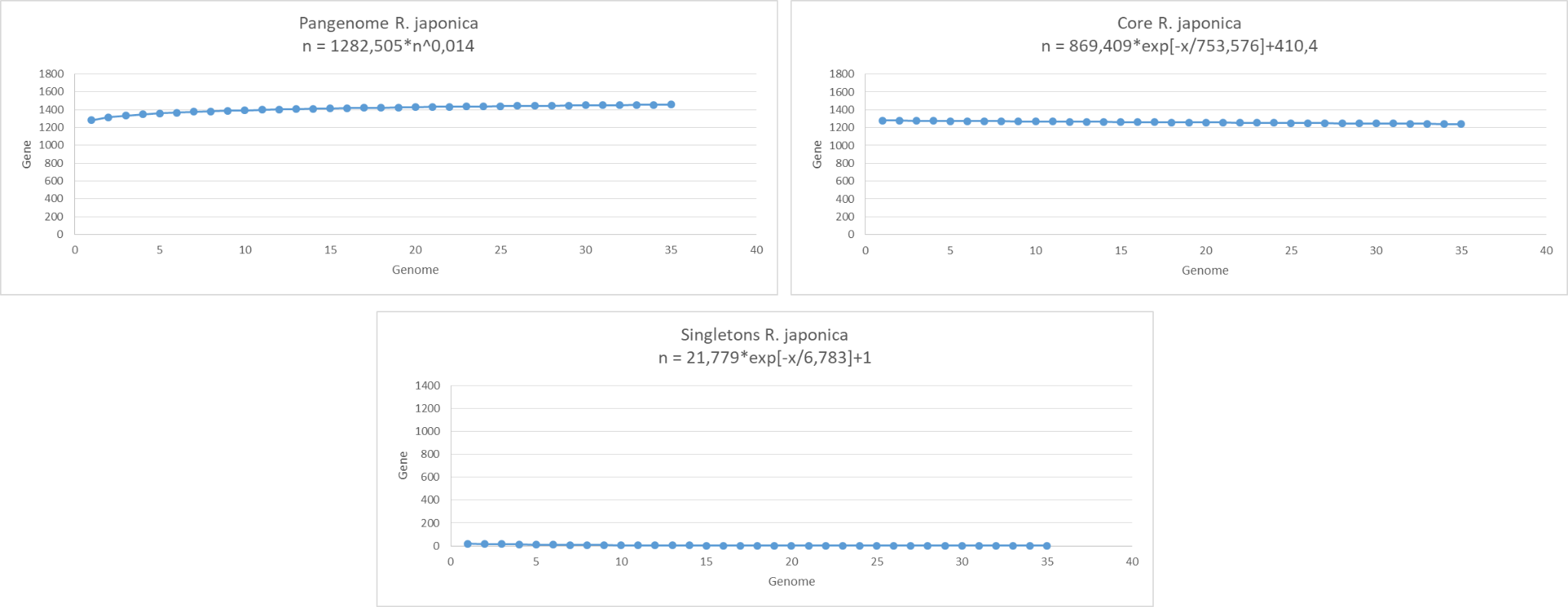


Source: By author, 2025. Note: Graphs representing the dynamics of the pangenome, core, and singletons among the analyzed genomes of *R. japonica*. The fitted equations are shown in each graph, representing the regression model used to describe the data trend.

B5. Pangenome, core genome and singletons of *Rickettsia parkeri*

*
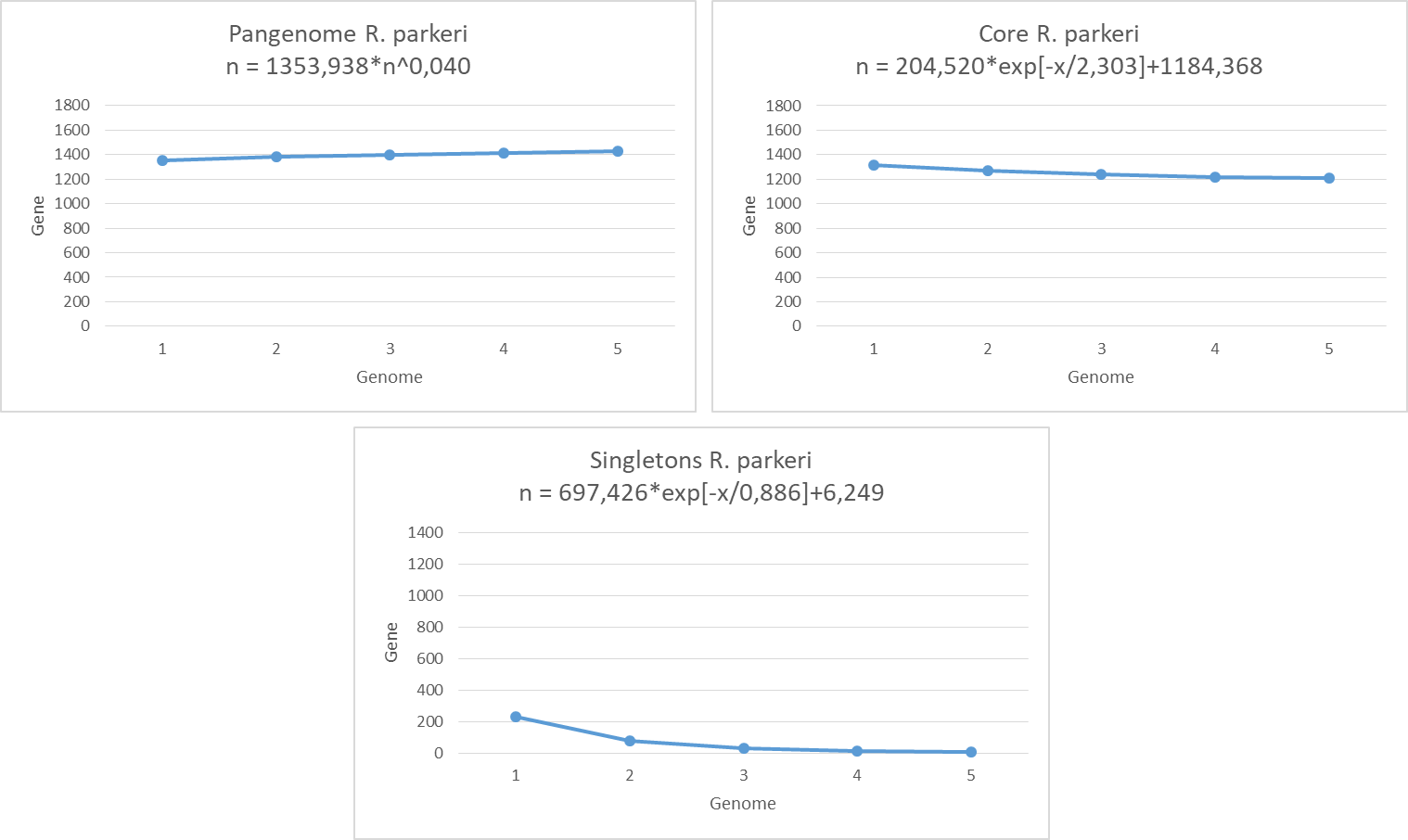
*

Source: By author, 2025. Note: Graphs representing the dynamics of the pangenome, core, and singletons among the analyzed genomes of *R. parkeri*. The fitted equations are shown in each graph, representing the regression model used to describe the data trend.

B6. Pangenome, core genome and singletons of *Rickettsia prowazekii*


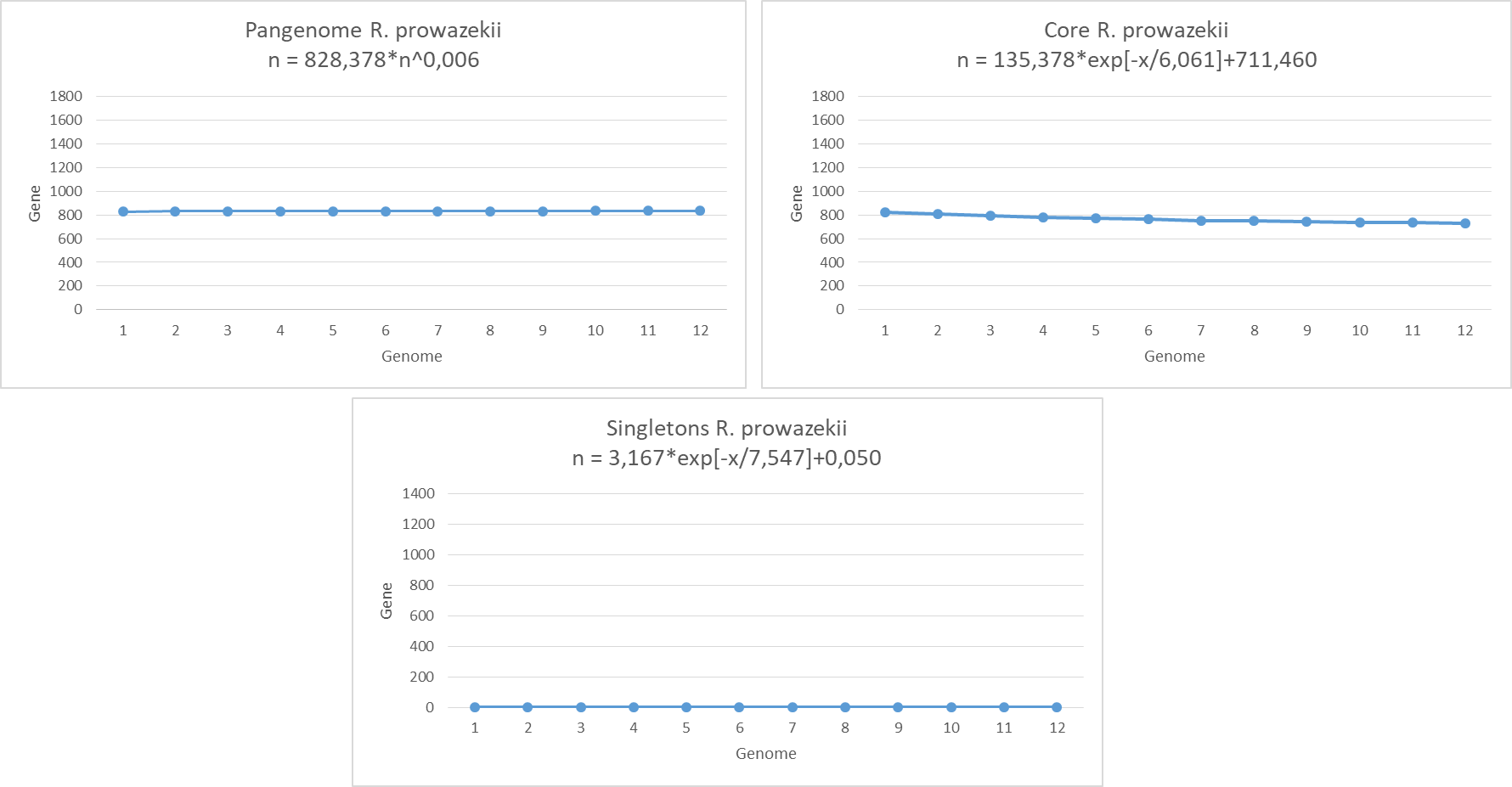


Source: By author, 2025. Note: Graphs representing the dynamics of the pangenome, core, and singletons among the analyzed genomes of *R. prowazekii*. The fitted equations are shown in each graph, representing the regression model used to describe the data trend.

B7. Pangenome, core genome and singletons of *Rickettsia rickettsii*


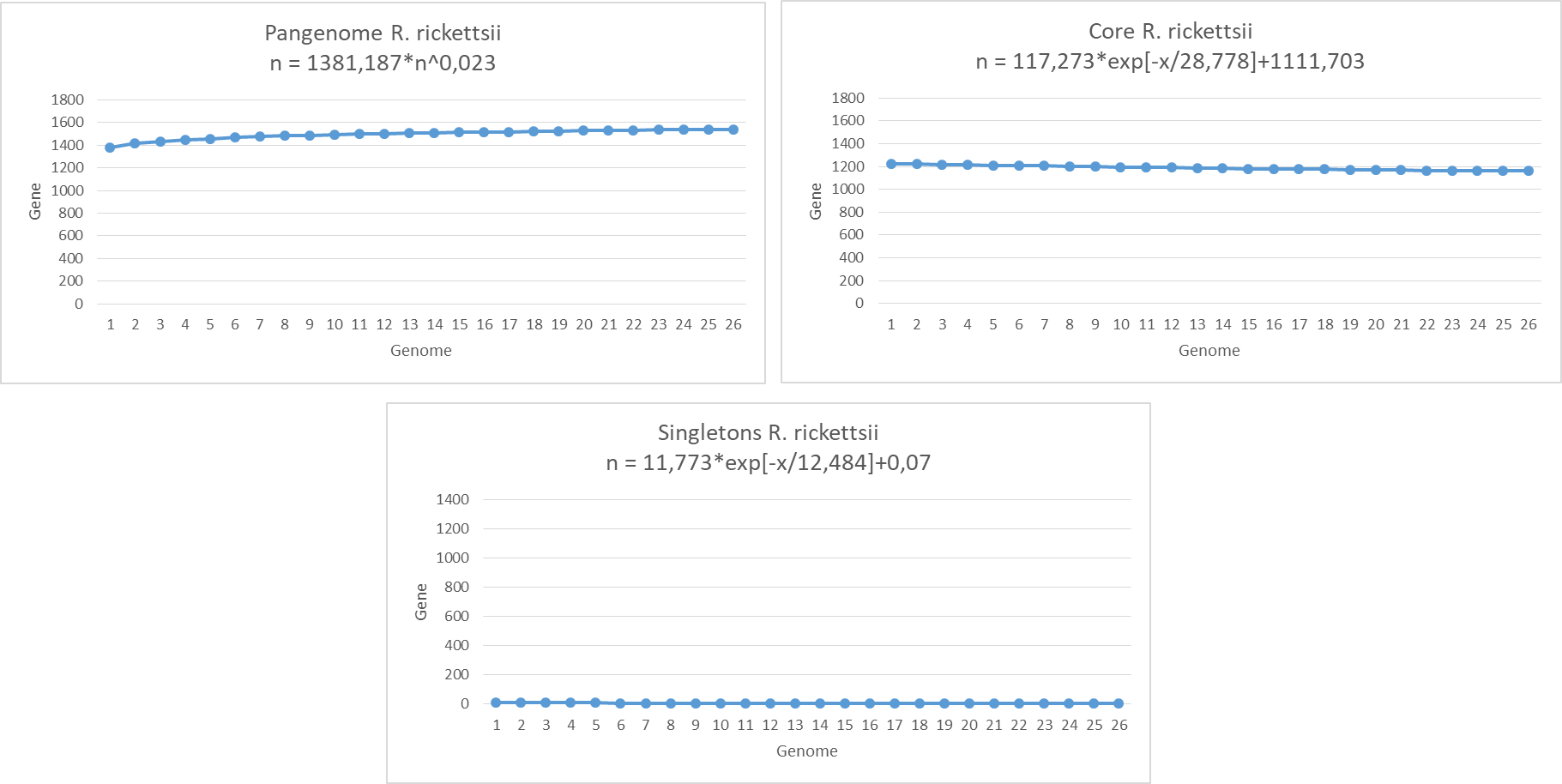


Source: By author, 2025. Note: Graphs representing the dynamics of the pangenome, core, and singletons among the analyzed genomes of *R. rickettsii*. The fitted equations are shown in each graph, representing the regression model used to describe the data trend.

B8. Pangenome, core genome and singletons of *Rickettsia sibirica*

*
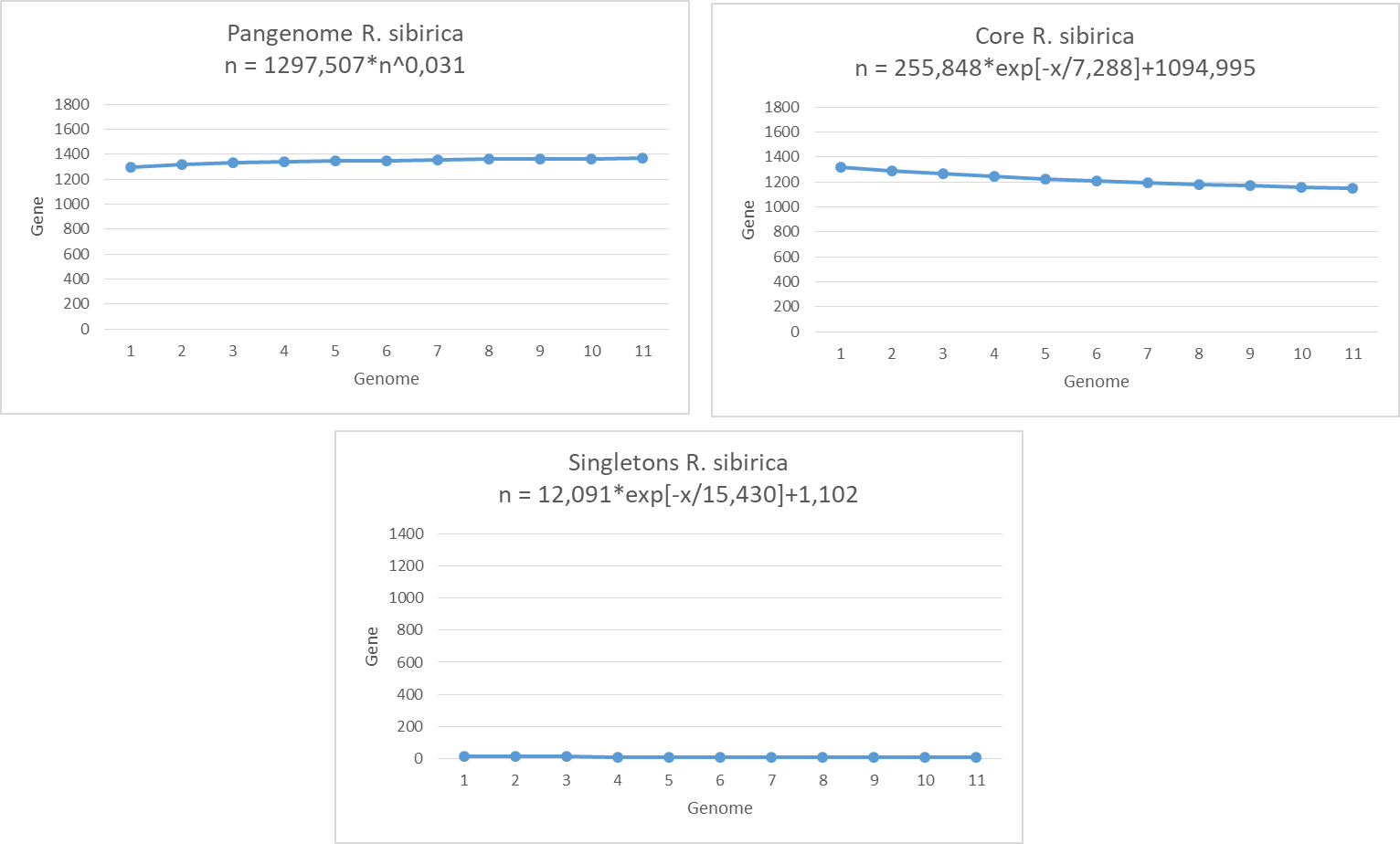
*

Source: By author, 2025. Note: Graphs representing the dynamics of the pangenome, core, and singletons among the analyzed genomes of *R. sibirica*. The fitted equations are shown in each graph, representing the regression model used to describe the data trend.

# **SUPPLEMENTARY MATERIAL C - CHARACTERIZATION OF ORTHOLOGOUS GENES**

C1. Characterization of orthologous genes of *R. amblyommatis*


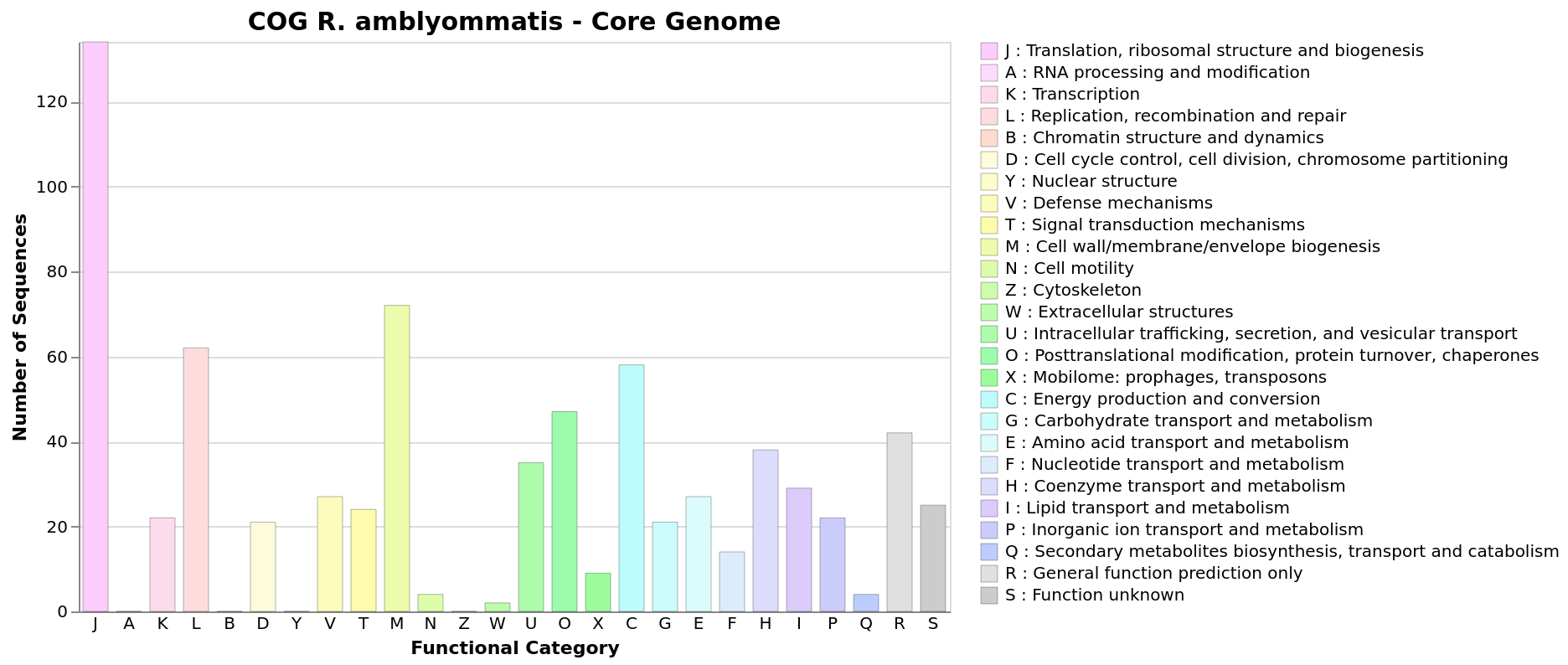

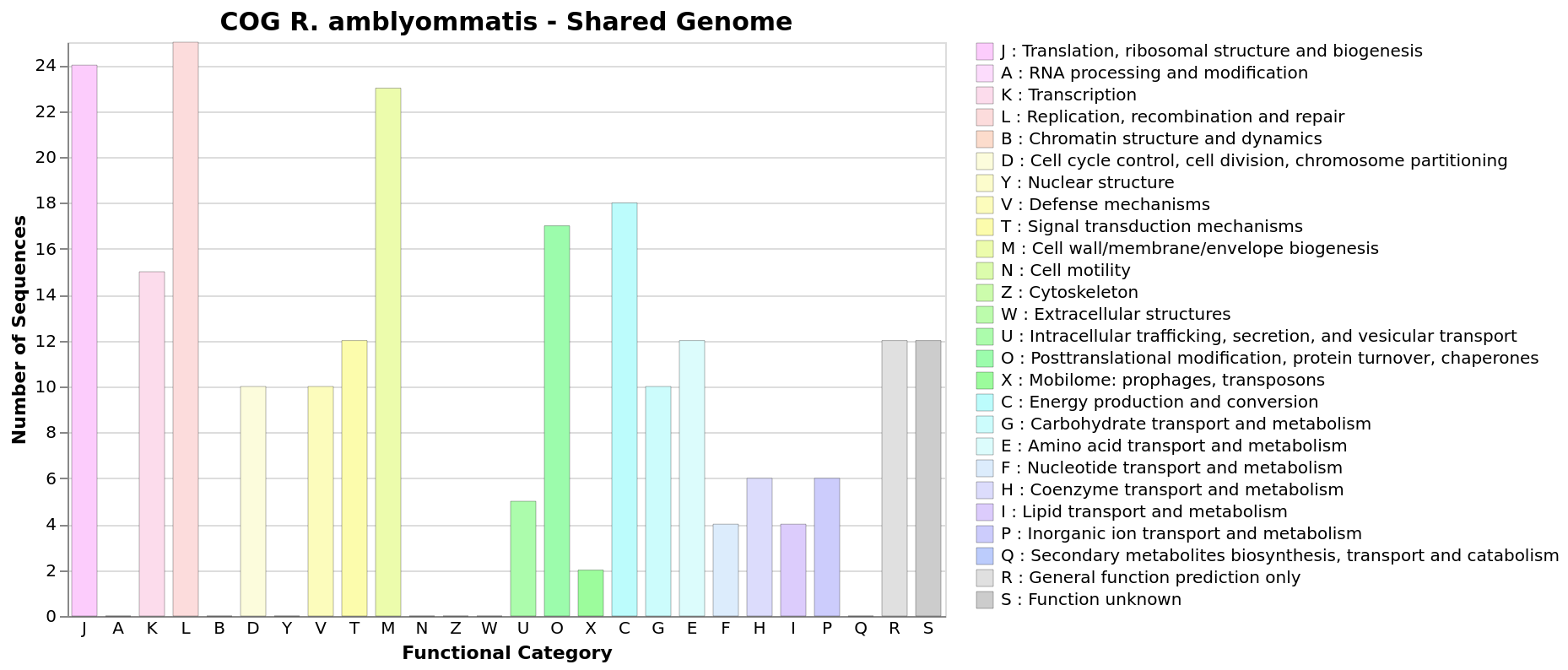

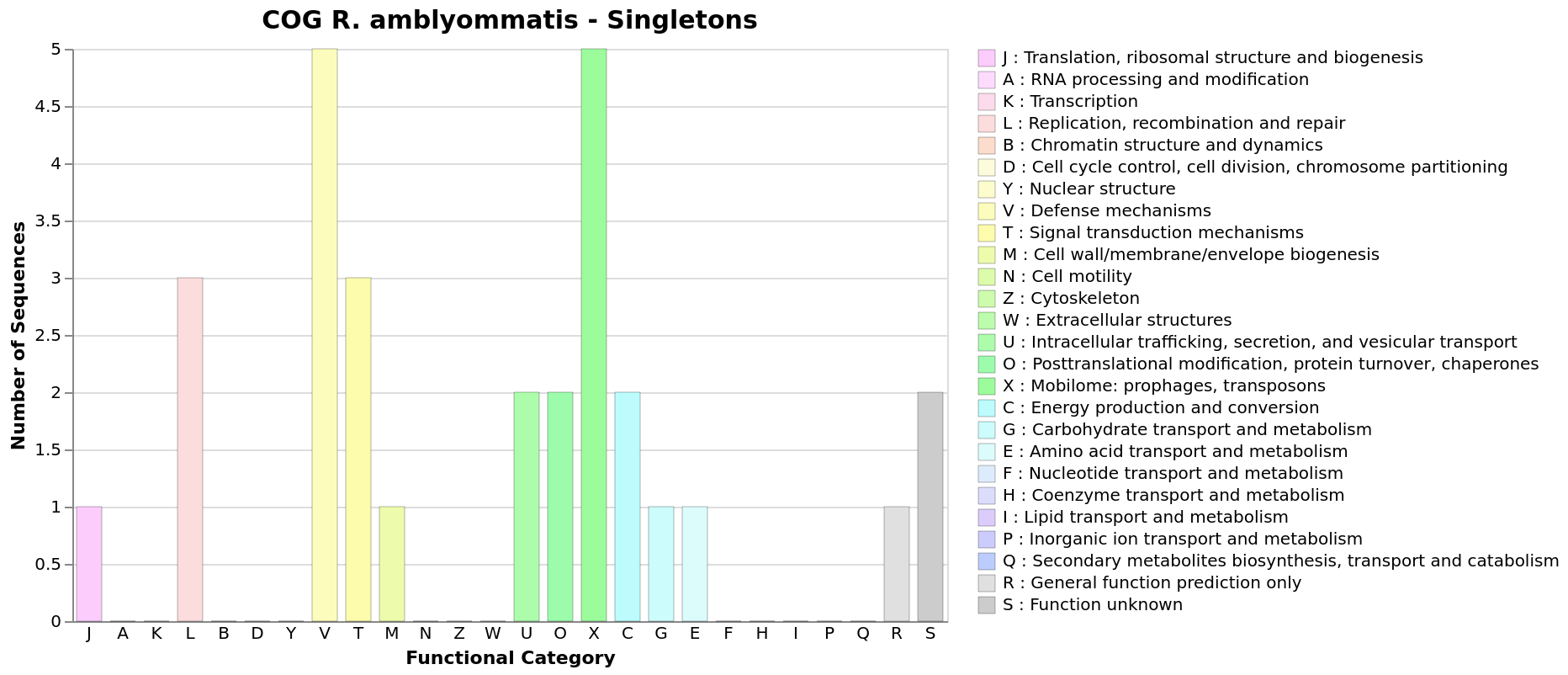


Source: By author, 2025. Note: Functional distribution of orthologous genes according to COG categories in the core genome, shared genome, and singleton gene sets. The bars represent the number of sequences assigned to each COG functional category for *R. amblyommatis*, as indicated in the legend beside each chart.

C2. Characterization of orthologous genes of *R. bellii*


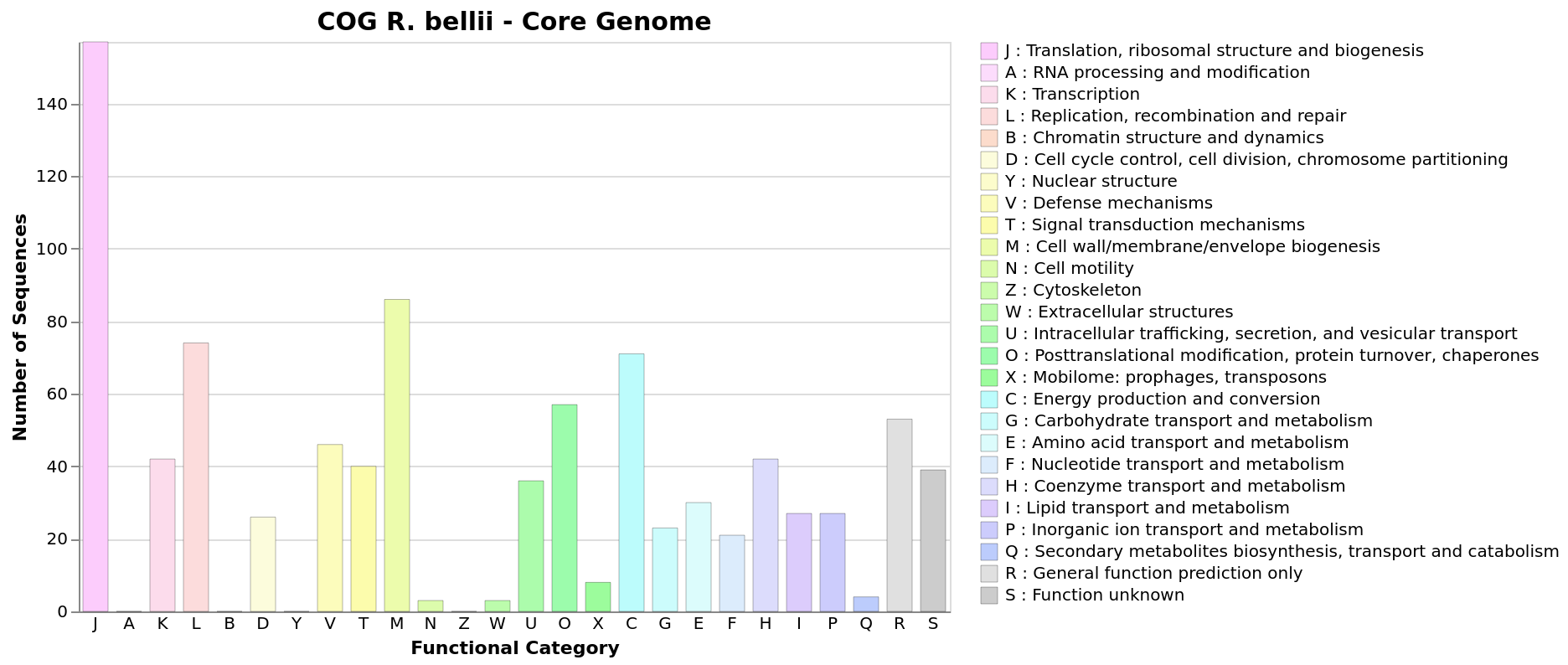

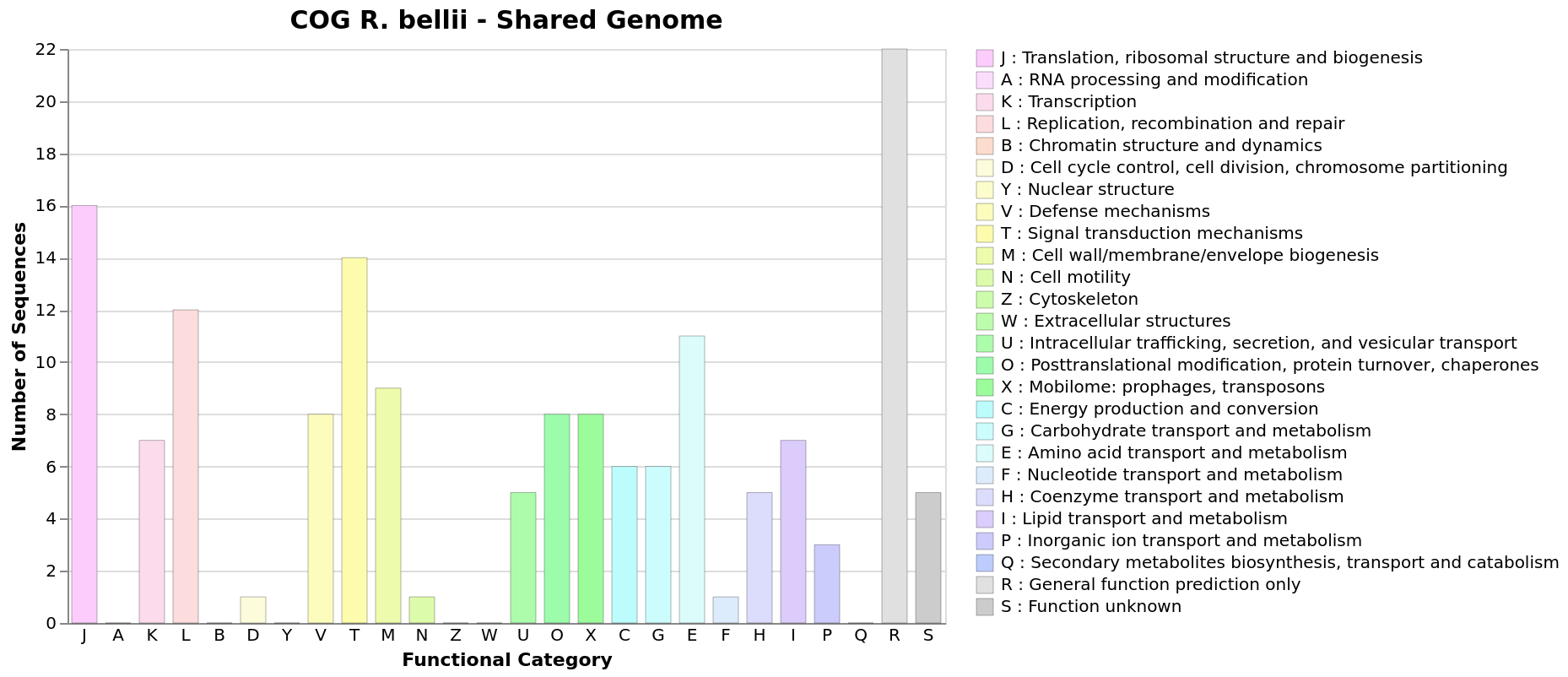

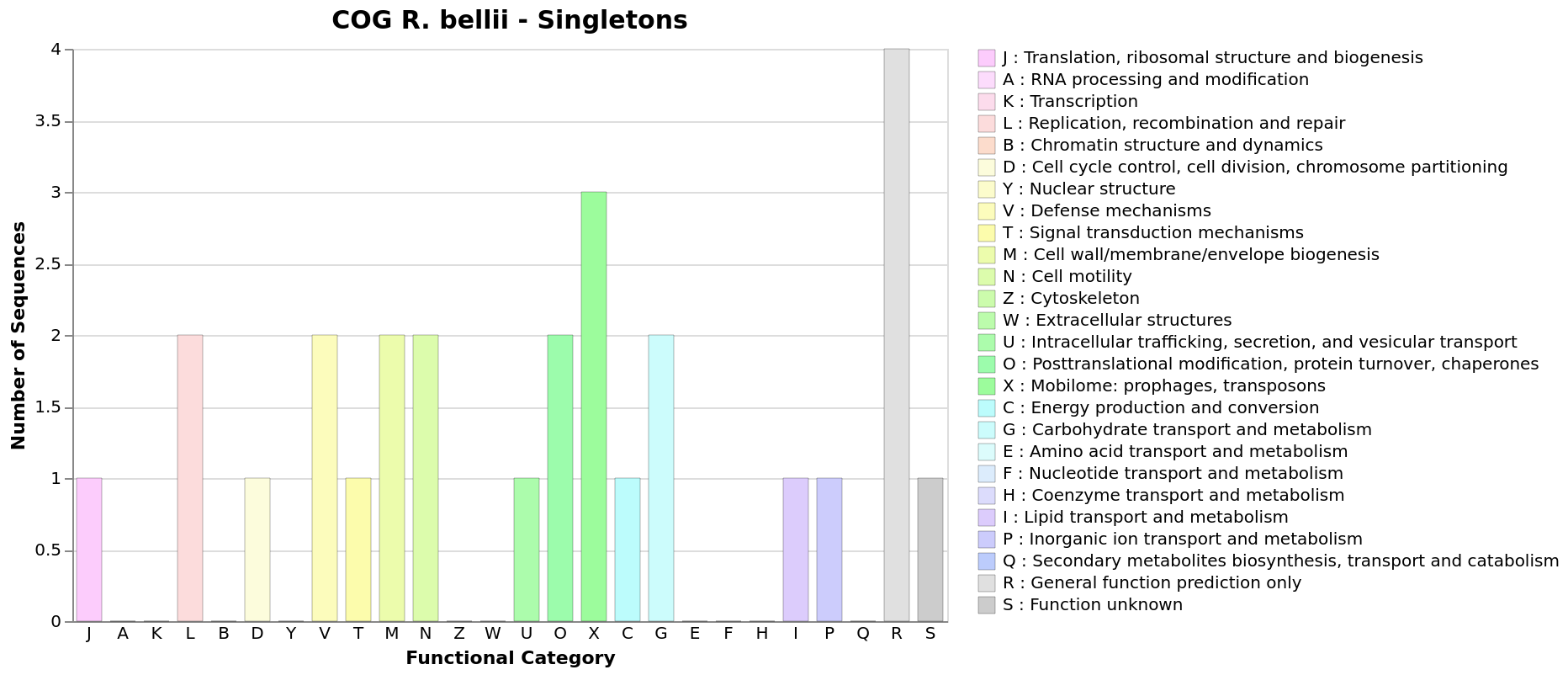


Source: By author, 2025. Note: Functional distribution of orthologous genes according to COG categories in the core genome, shared genome, and singleton gene sets. The bars represent the number of sequences assigned to each COG functional category for *R. bellii*, as indicated in the legend beside each chart.

C3. Characterization of orthologous genes of *R. conorii*


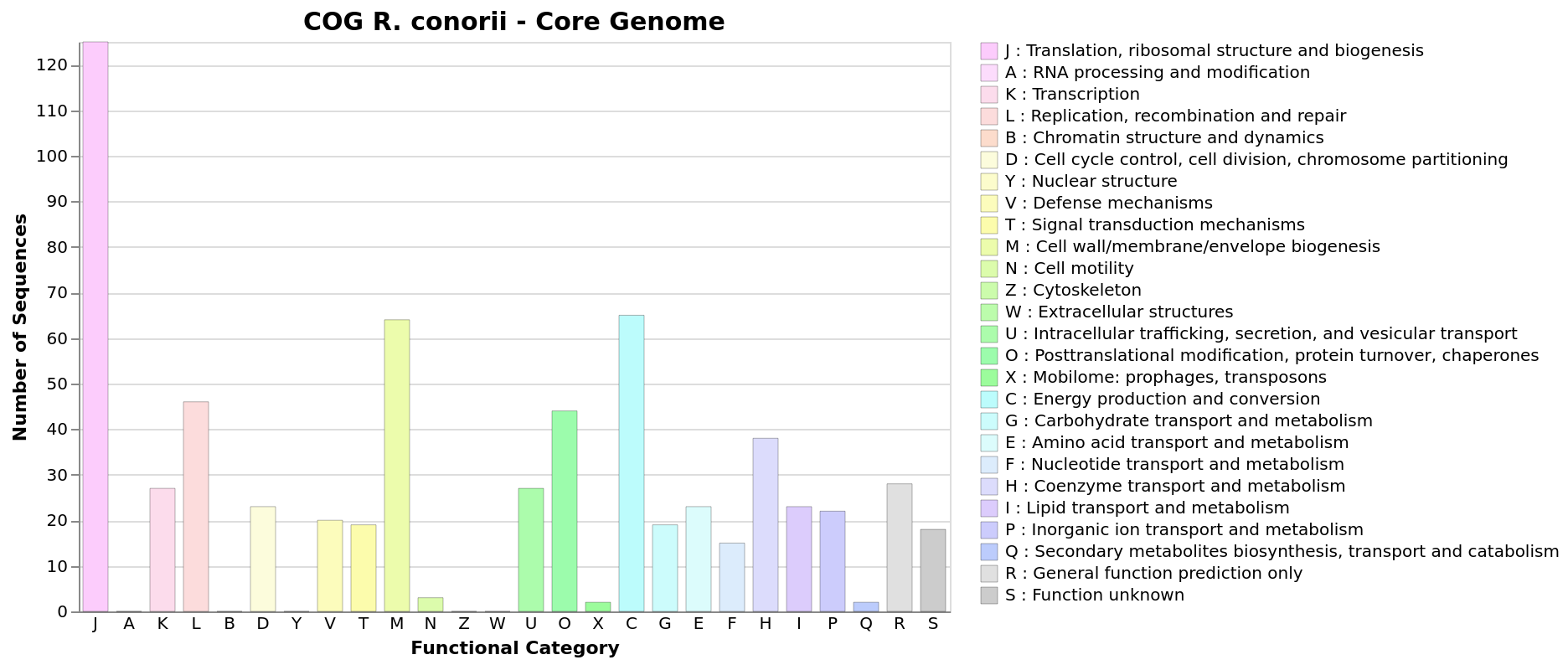

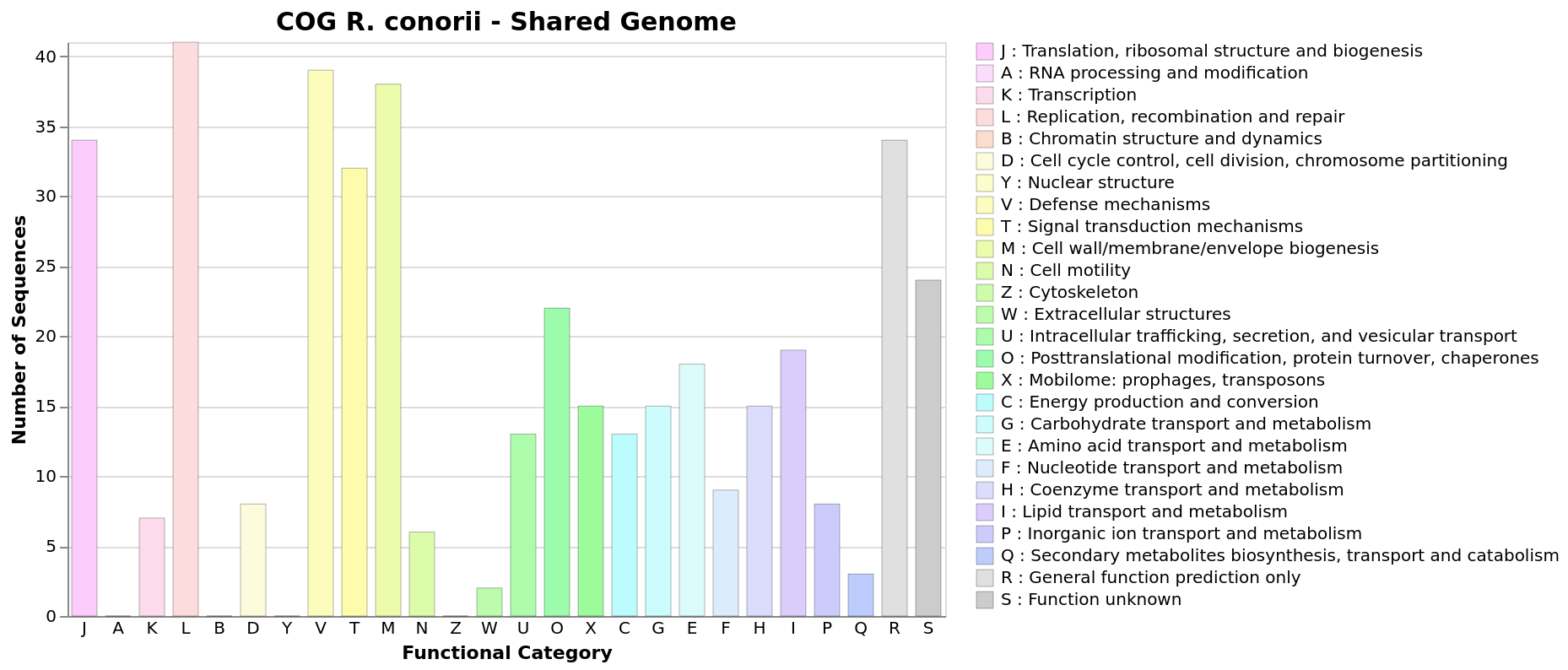


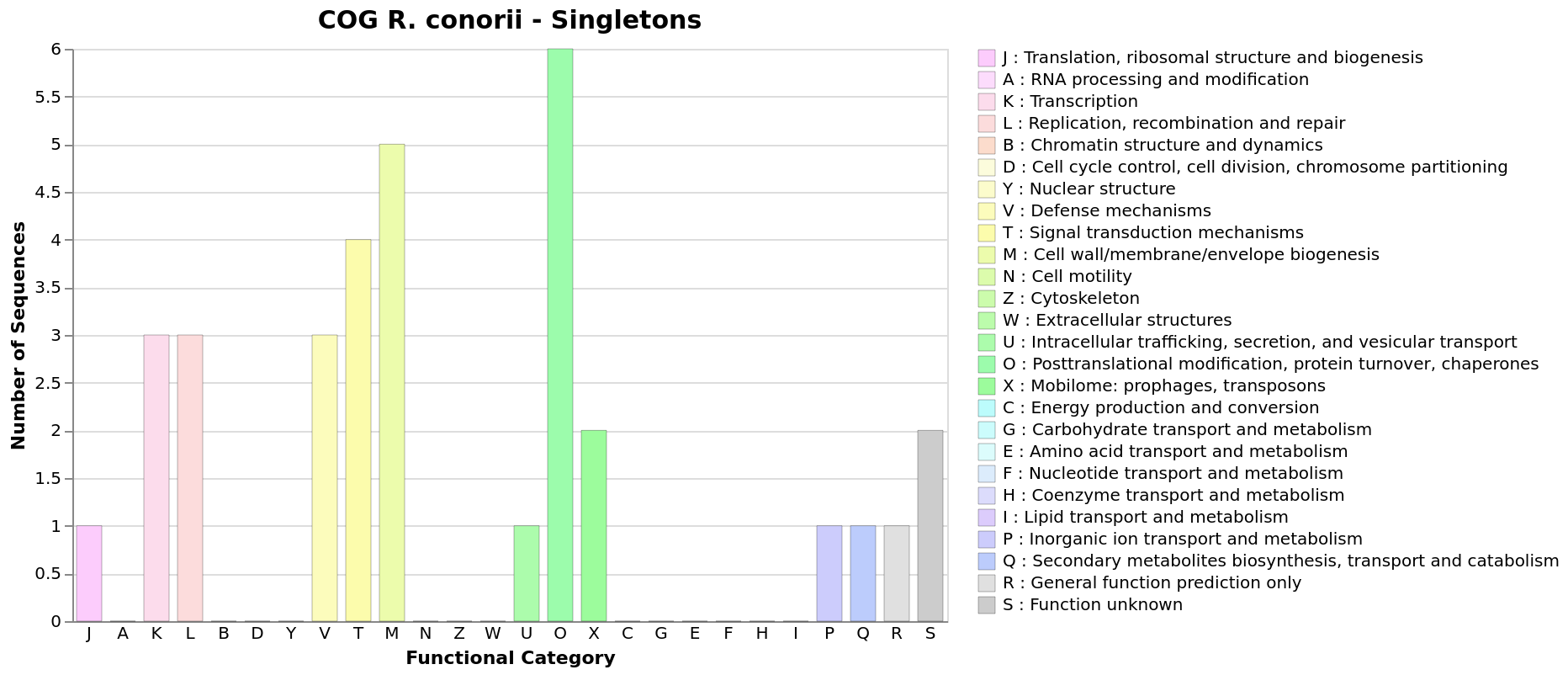


Source: By author, 2025. Note: Functional distribution of orthologous genes according to COG categories in the core genome, shared genome, and singleton gene sets. The bars represent the number of sequences assigned to each COG functional category for *R. conorii*, as indicated in the legend beside each chart.

C4. Characterization of orthologous genes of *R. japonica*


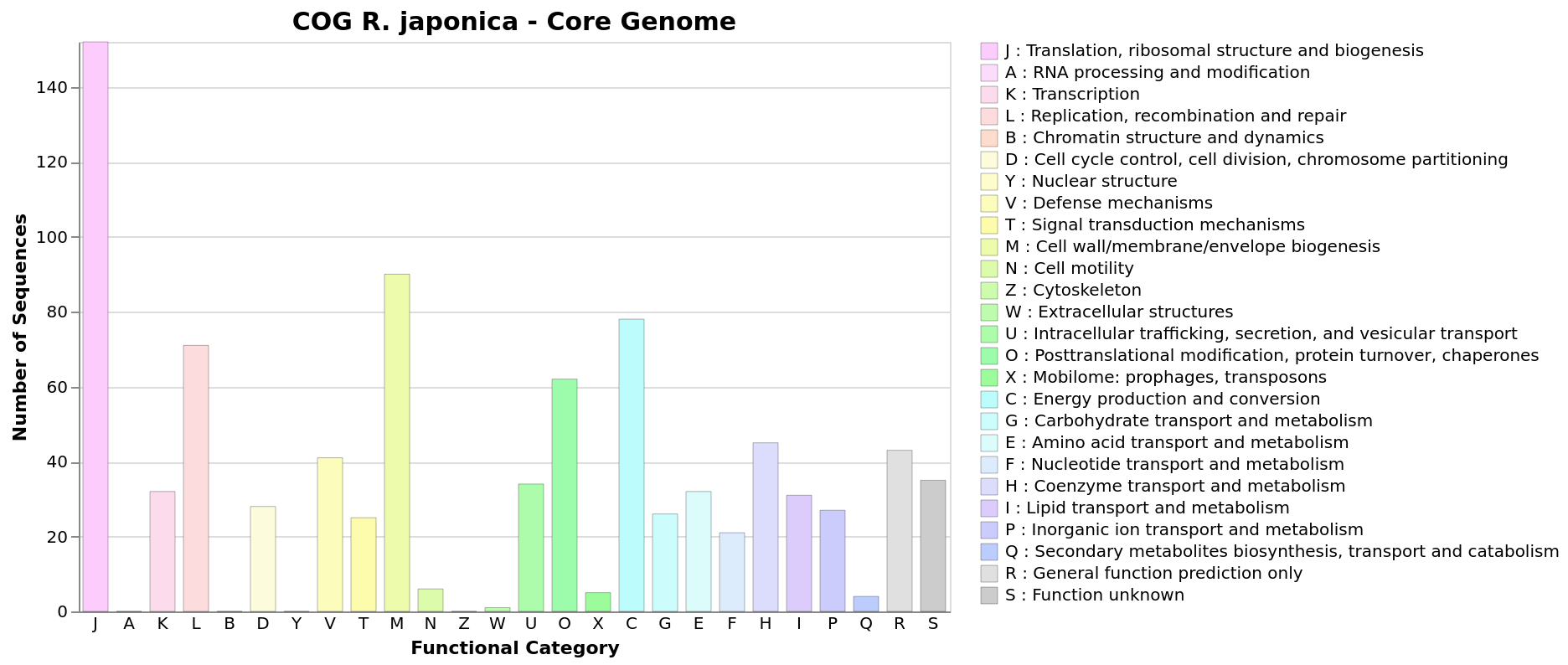

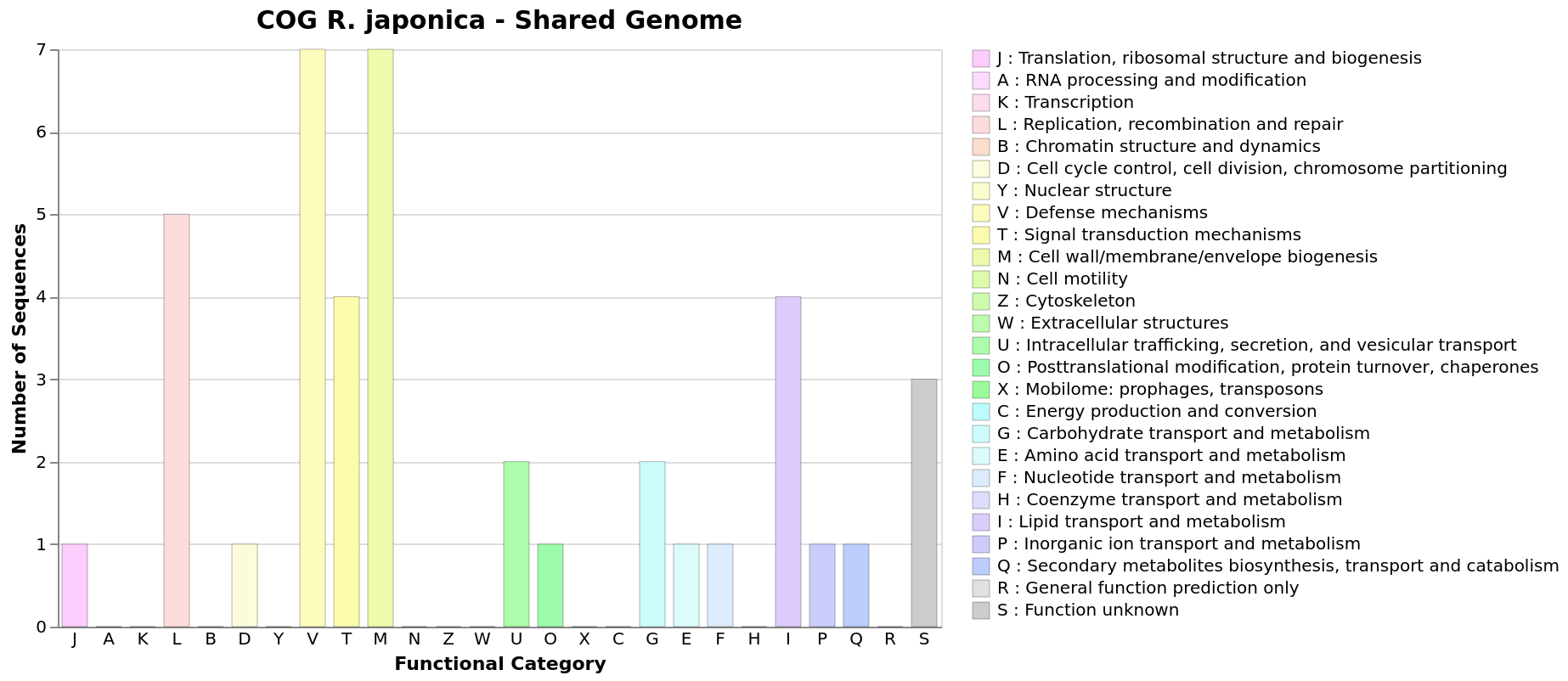

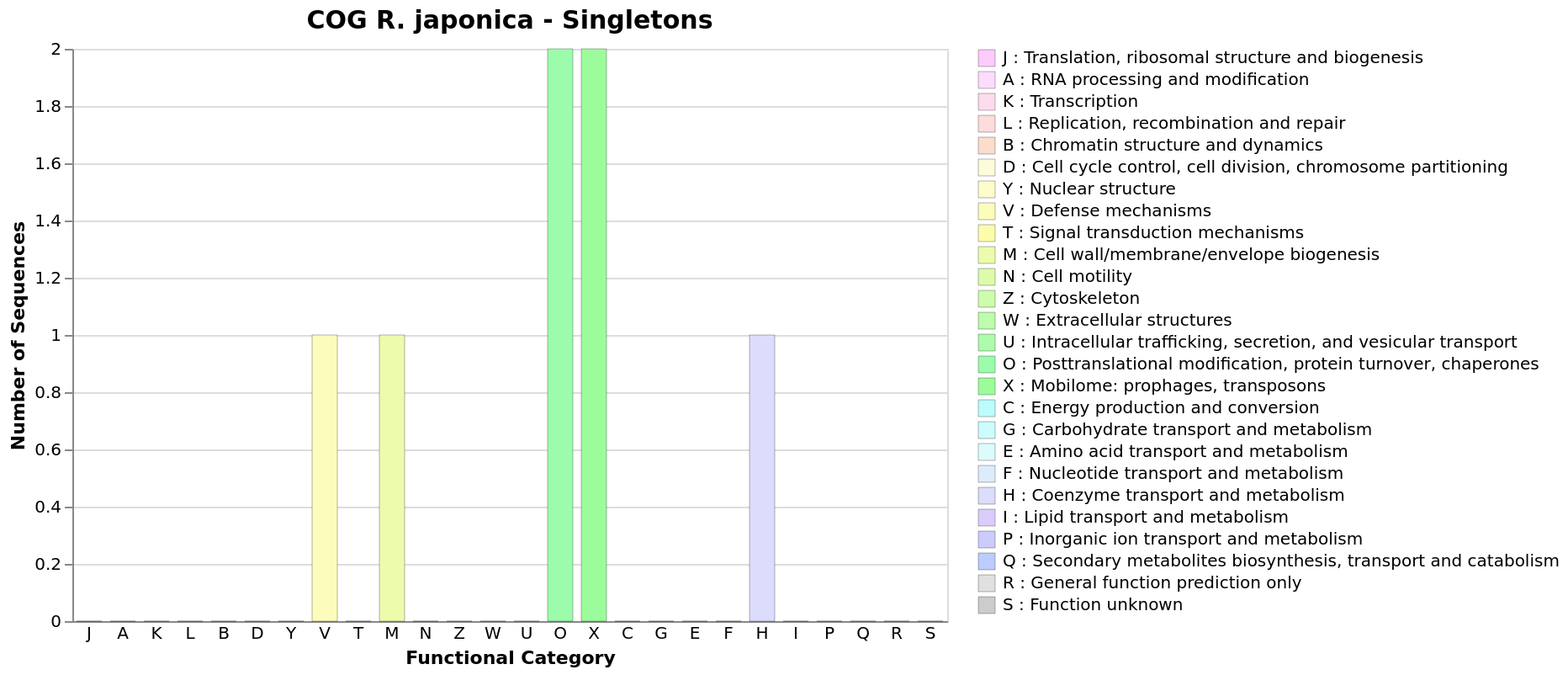


Source: By author, 2025. Note: Functional distribution of orthologous genes according to COG categories in the core genome, shared genome, and singleton gene sets. The bars represent the number of sequences assigned to each COG functional category for *R. japonica*, as indicated in the legend beside each chart.

C5. Characterization of orthologous genes of *R. parkeri*


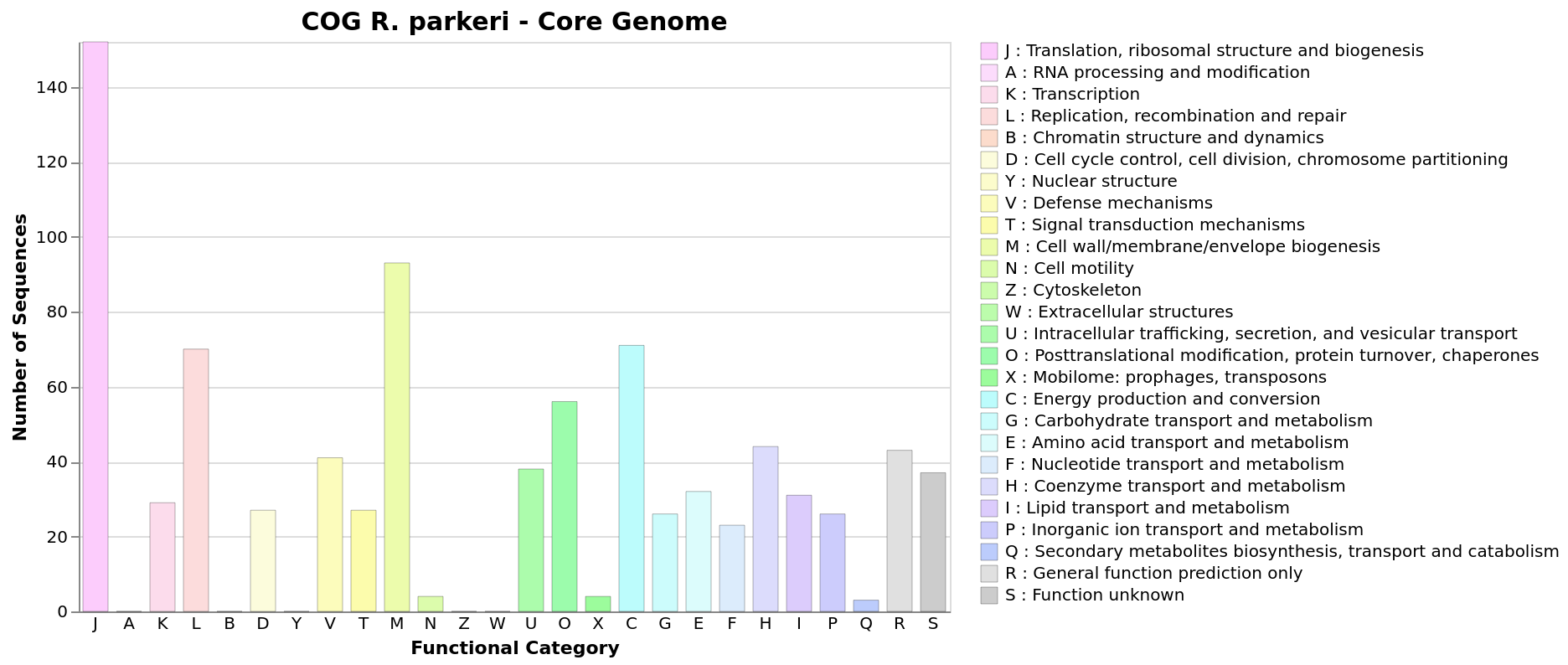

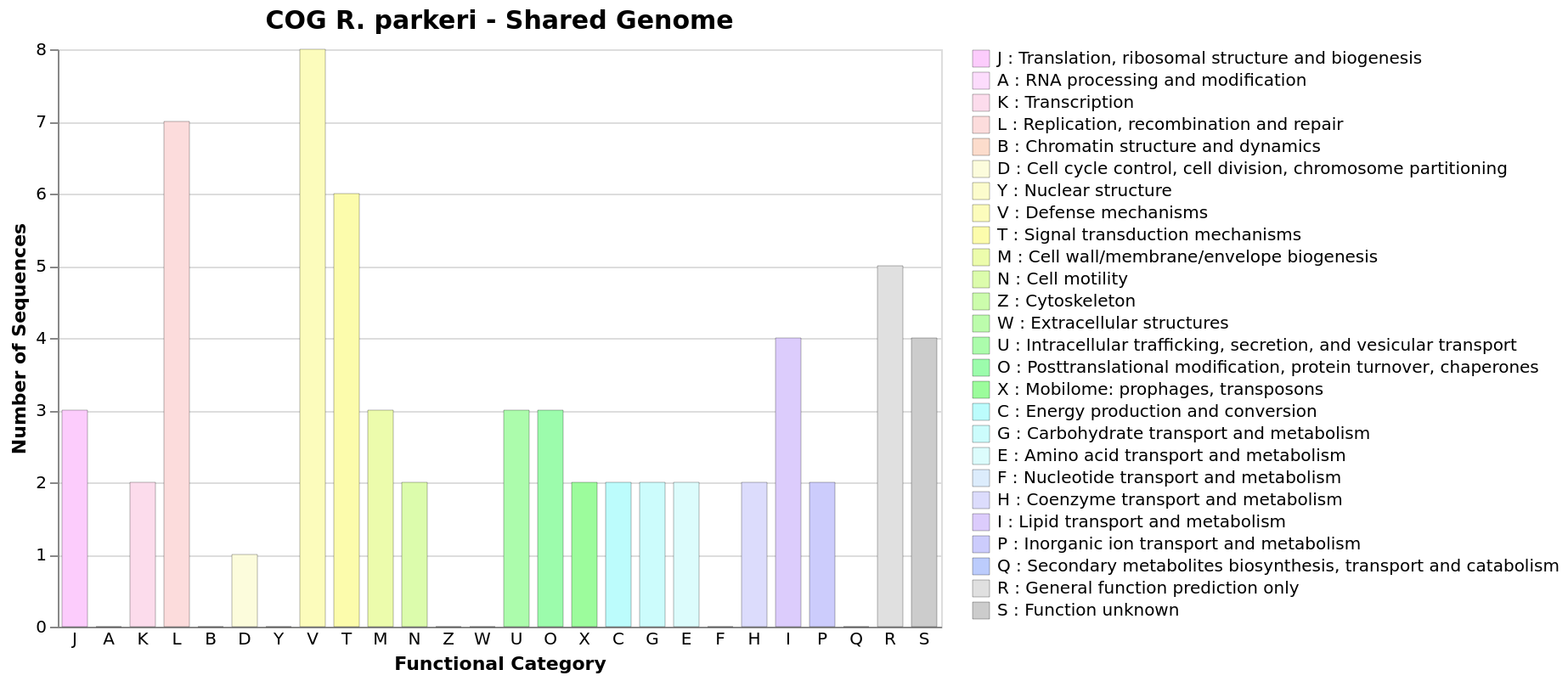

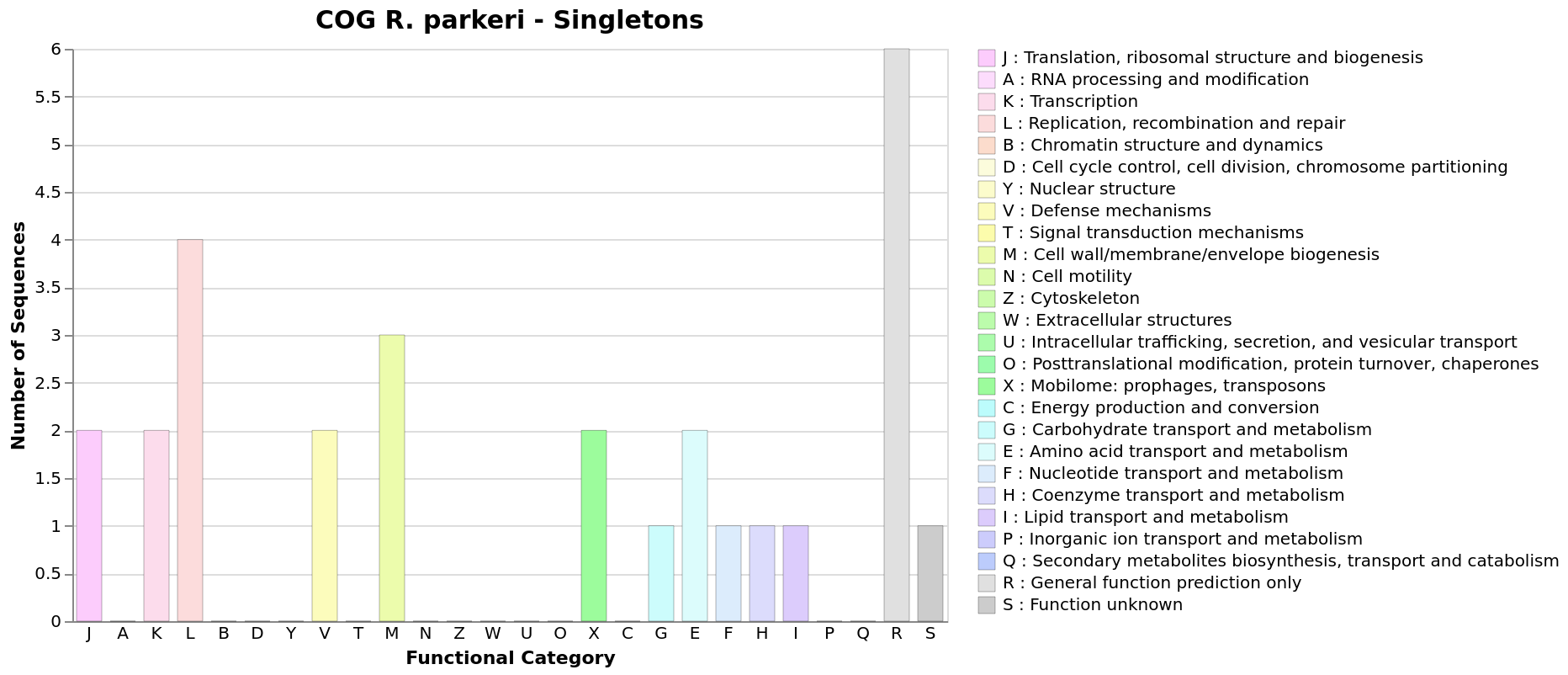


Source: By author, 2025. Note: Functional distribution of orthologous genes according to COG categories in the core genome, shared genome, and singleton gene sets. The bars represent the number of sequences assigned to each COG functional category for *R. parkeri*, as indicated in the legend beside each chart.

C6. Characterization of orthologous genes of *R. prowazekii*


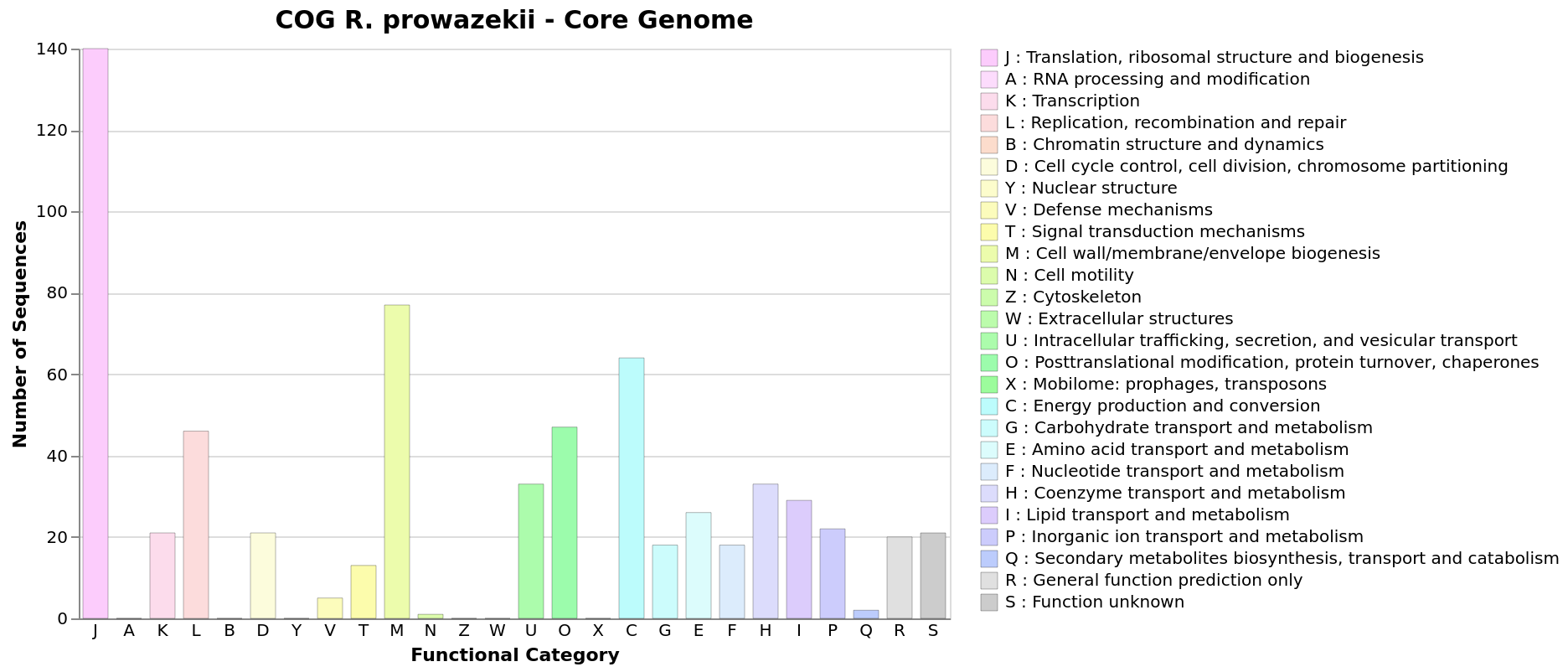

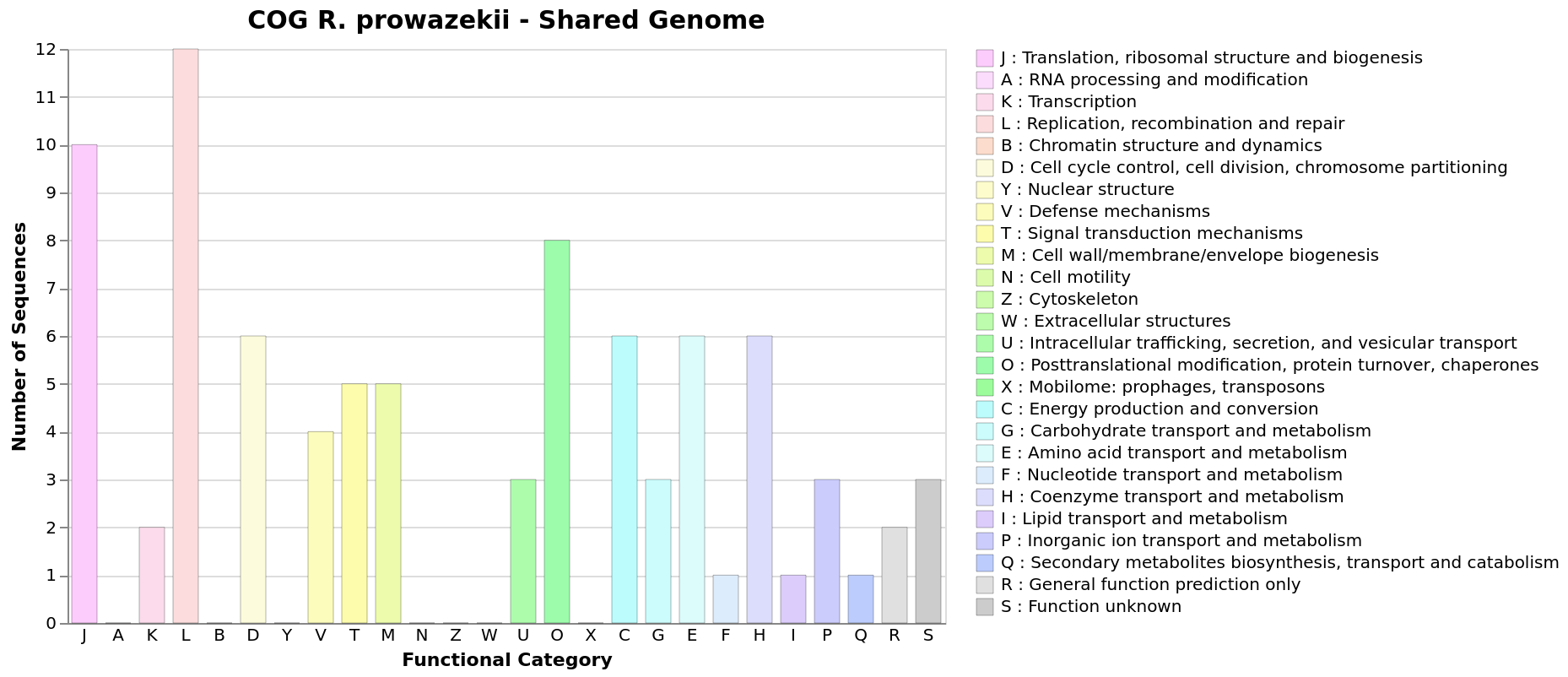

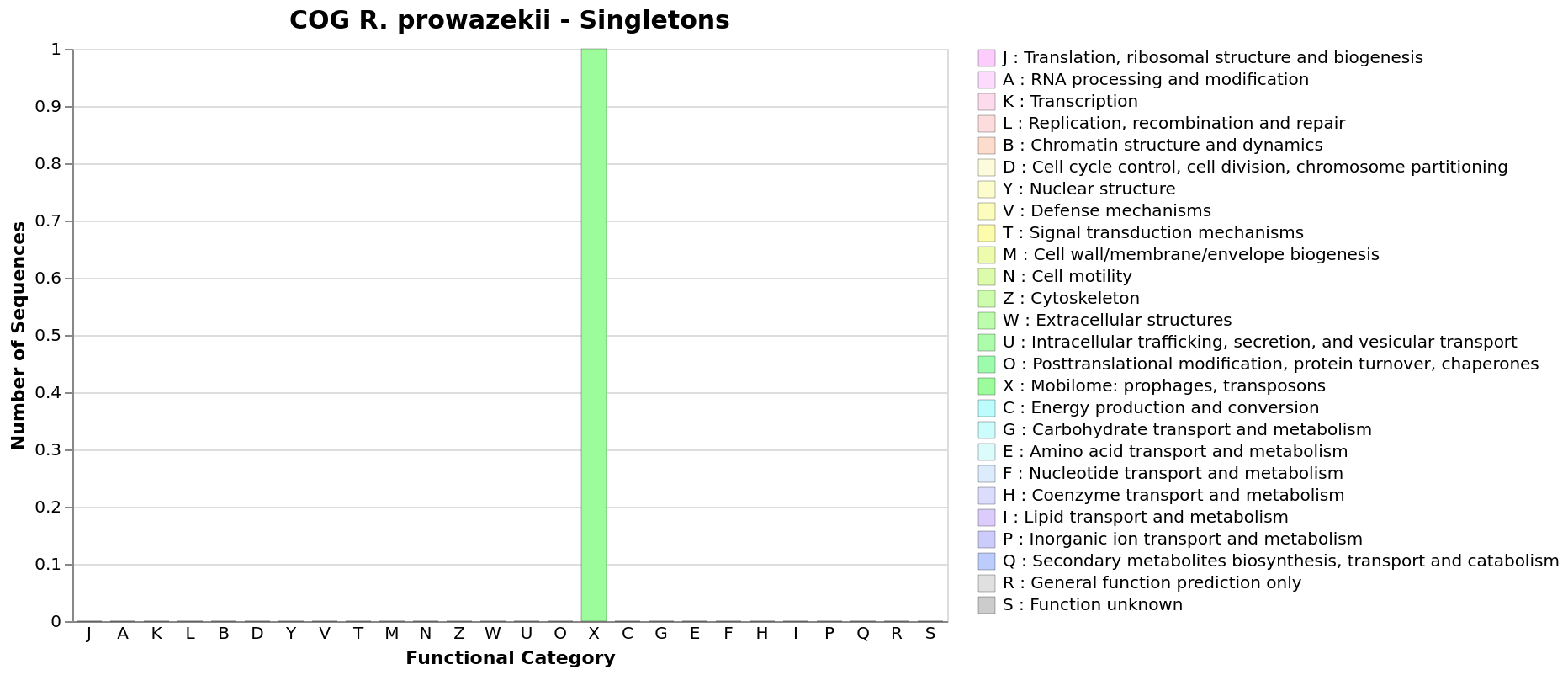


Source: By author, 2025. Note: Functional distribution of orthologous genes according to COG categories in the core genome, shared genome, and singleton gene sets. The bars represent the number of sequences assigned to each COG functional category for *R. prowazekii*, as indicated in the legend beside each chart.

C7. Characterization of orthologous genes of *R. rickettsii*


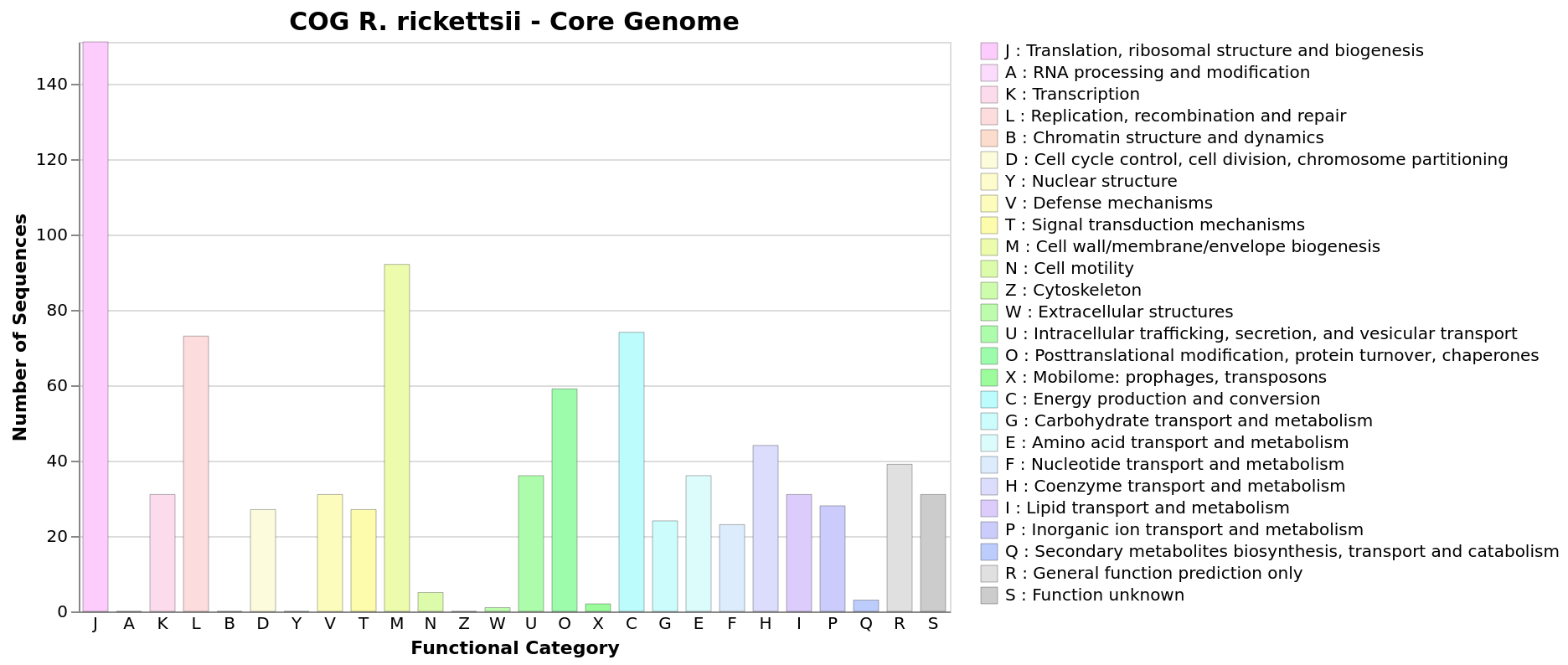

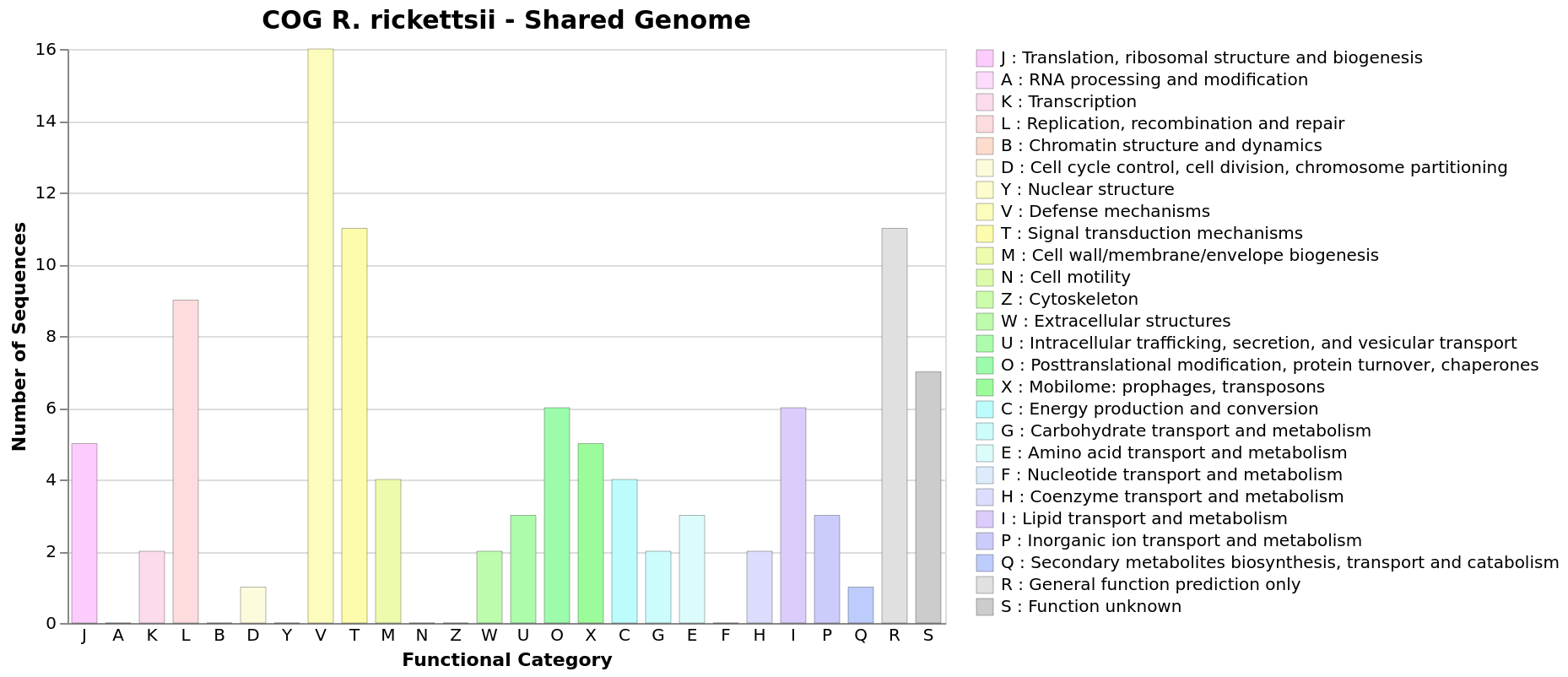

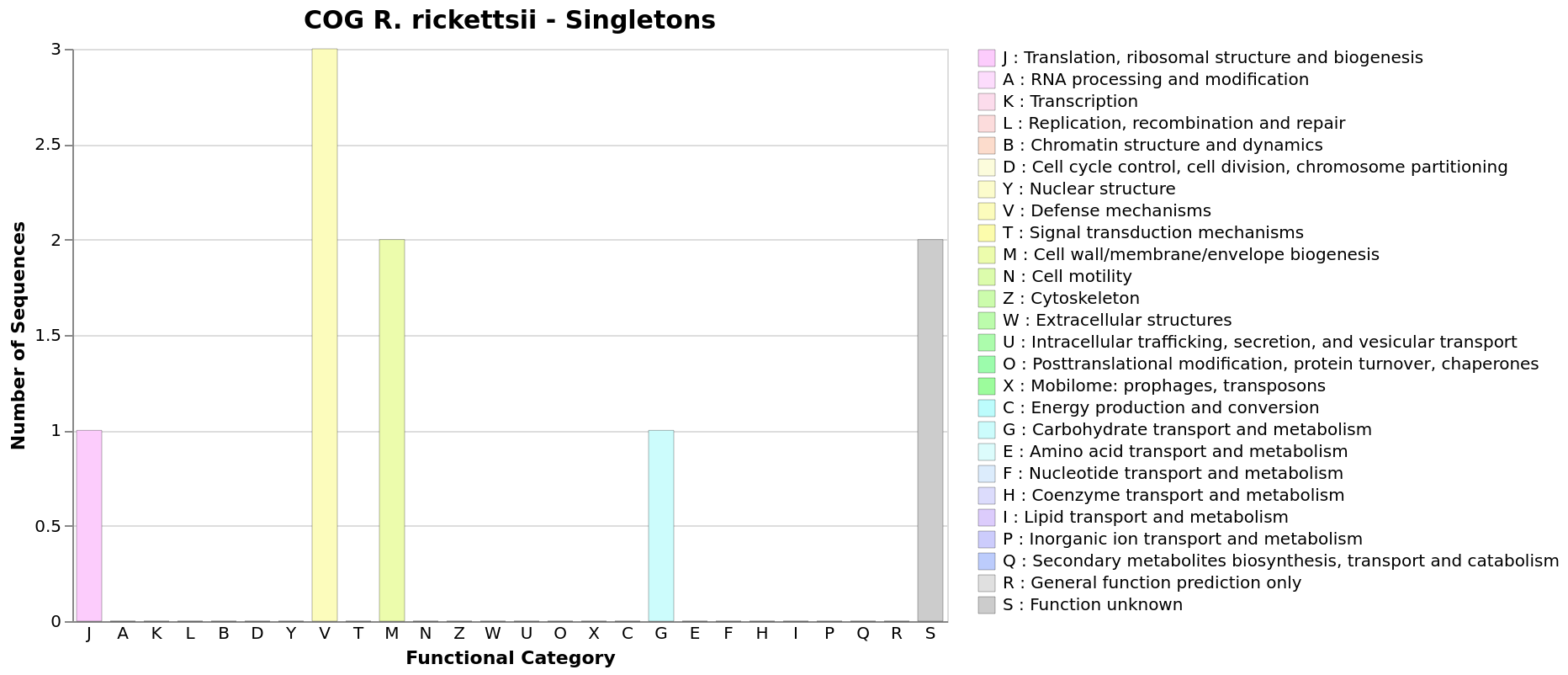


Source: By author, 2025. Note: Functional distribution of orthologous genes according to COG categories in the core genome, shared genome, and singleton gene sets. The bars represent the number of sequences assigned to each COG functional category for *R. rickettsii*, as indicated in the legend beside each chart.

C8. Characterization of orthologous genes of *R. sibirica*


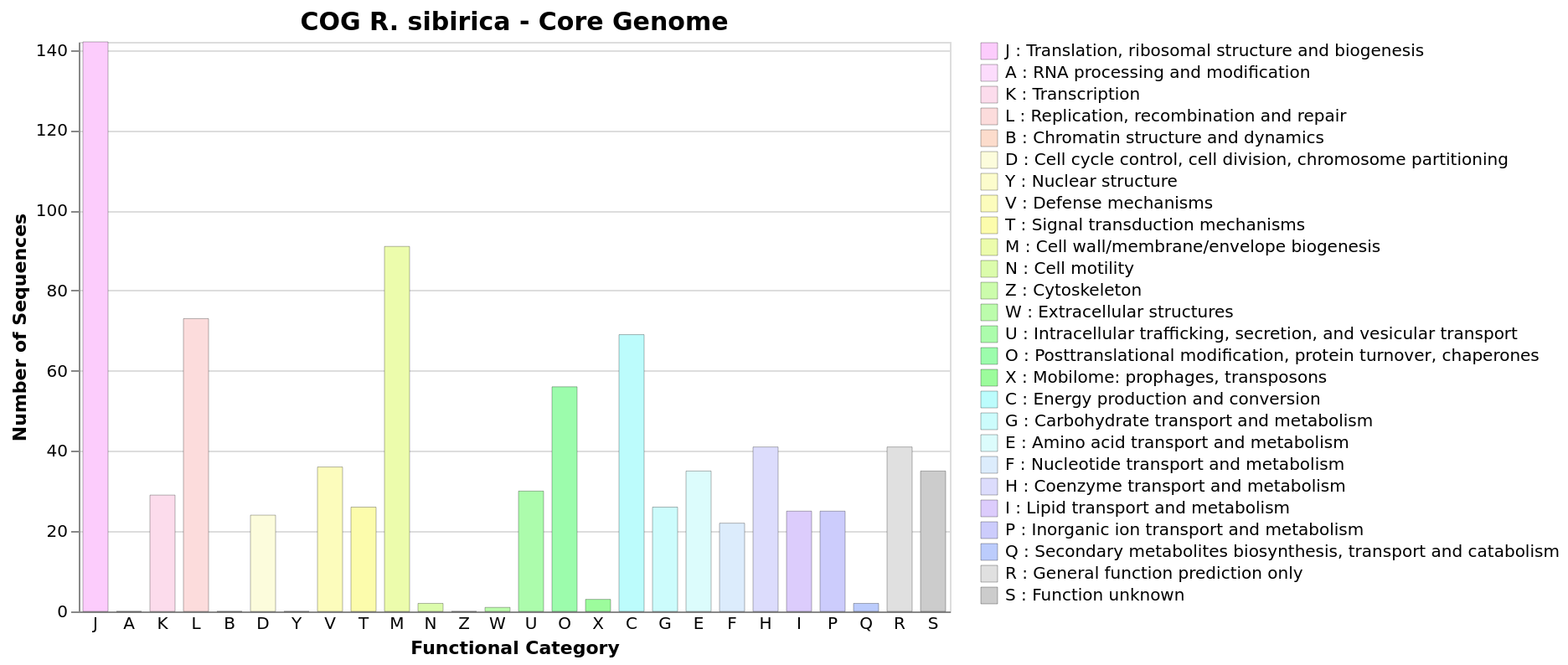

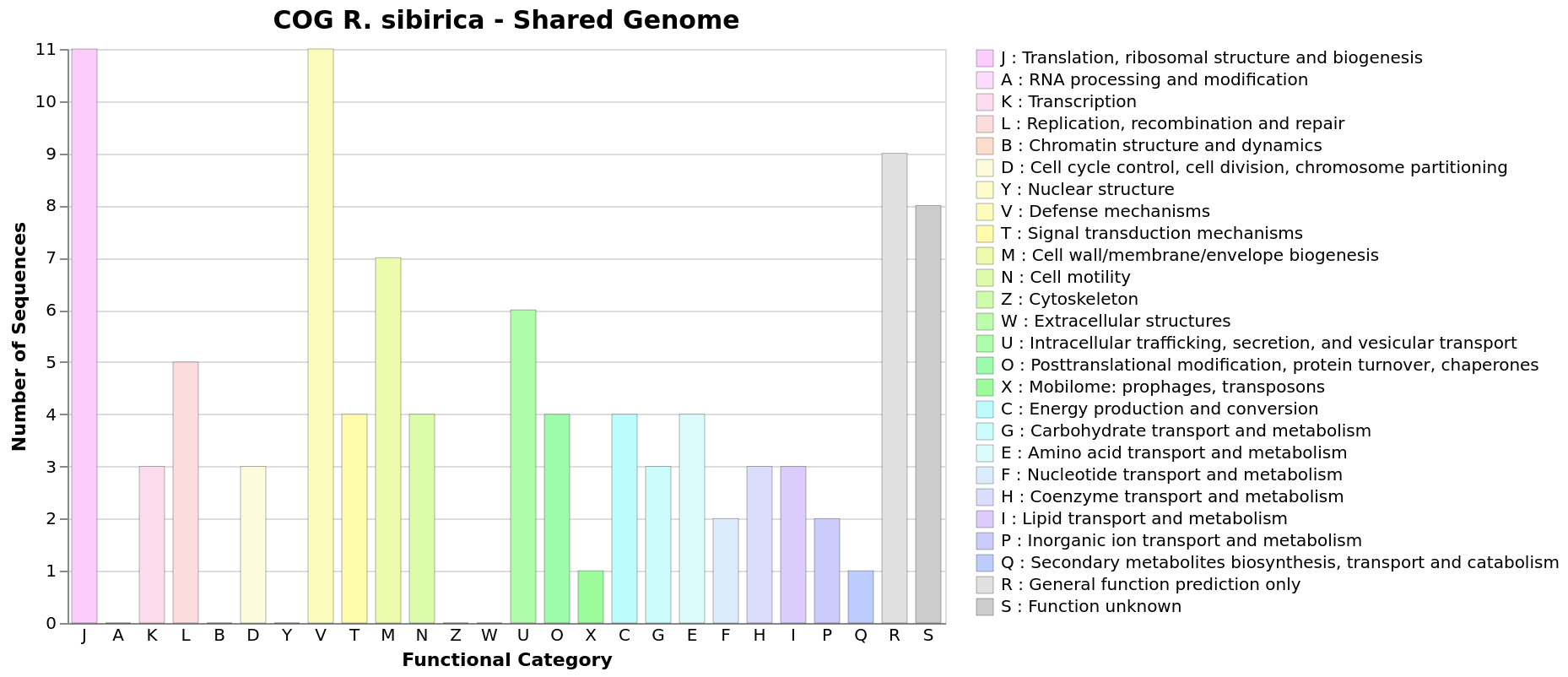

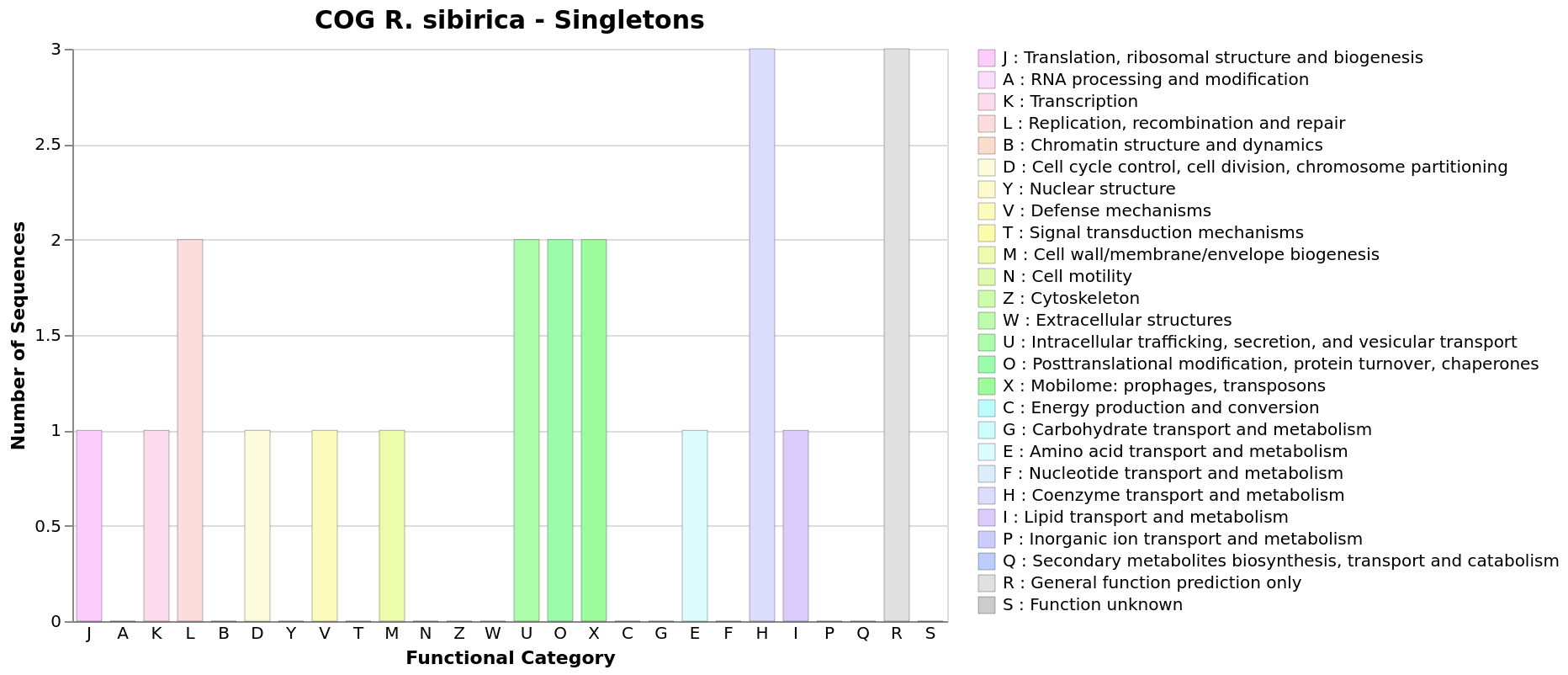


Source: By author, 2025. Note: Functional distribution of orthologous genes according to COG categories in the core genome, shared genome, and singleton gene sets. The bars represent the number of sequences assigned to each COG functional category for *R. sibirica*, as indicated in the legend beside each chart.

# **SUPPLEMENTARY MATERIAL D - GENE SYNTENY**

D1. Mauve Analysis of *R. amblyommatis*
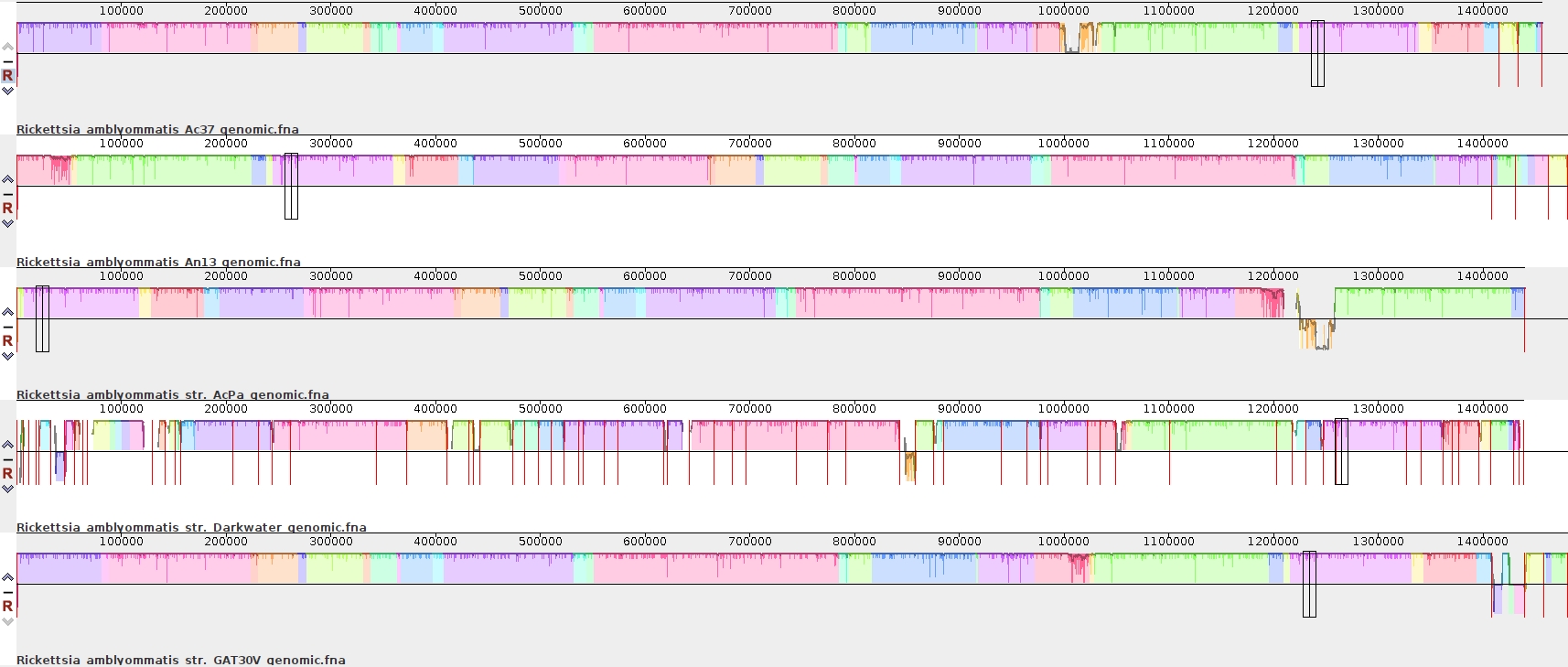


Source: By author, 2025. Note: Genomic alignment among genomes of *R. amblyommatis*. The open bars observed in this figure represent software indicators associated with nucleotide residue positioning across samples, facilitating the visualization of potential translocated regions.

D2. Mauve Analysis of *R. bellii*


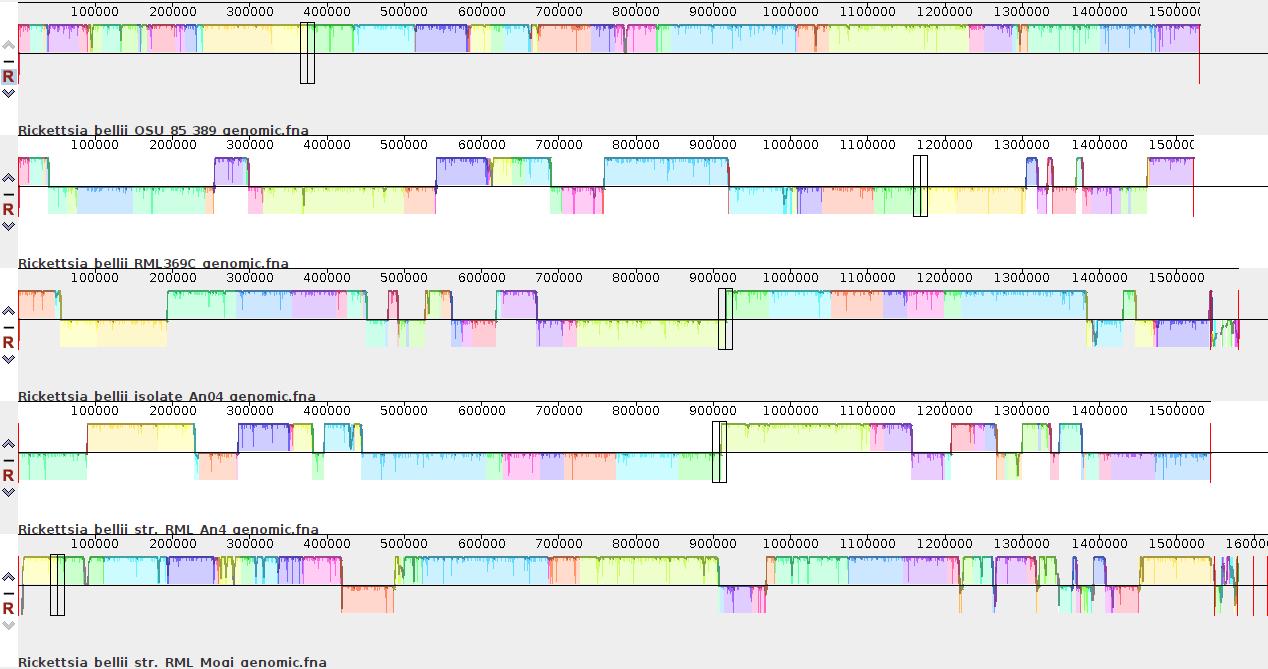


Source: By author, 2025. Note: Genomic alignment among genomes of *R. bellii*. The open bars observed in this figure represent software indicators associated with nucleotide residue positioning across samples, facilitating the visualization of potential translocated regions.

D3. Mauve Analysis of *R. conorii*


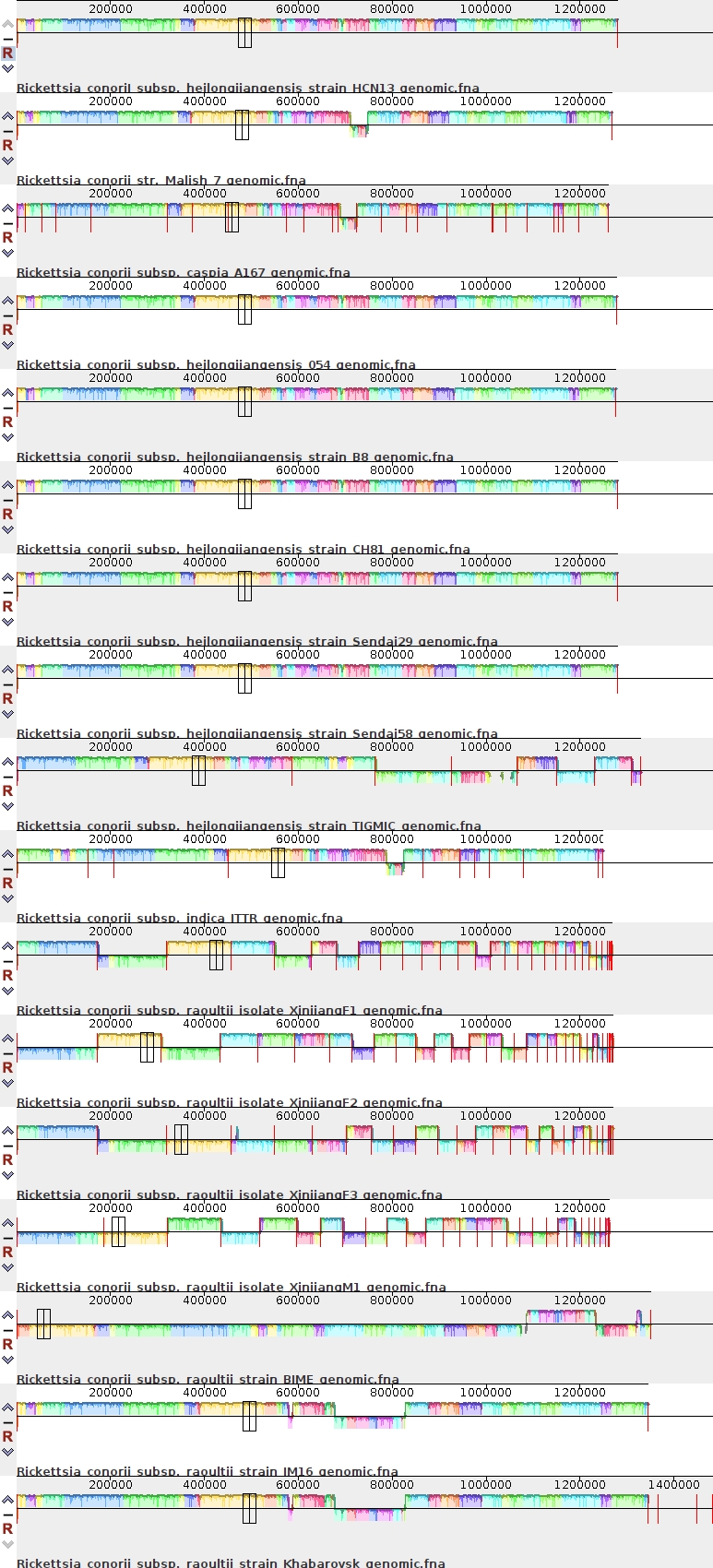


Source: By author, 2025. Note: Genomic alignment among genomes of *R. conorii*. The open bars observed in this figure represent software indicators associated with nucleotide residue positioning across samples, facilitating the visualization of potential translocated regions.

D4. Mauve Analysis of *R. japonica*

*
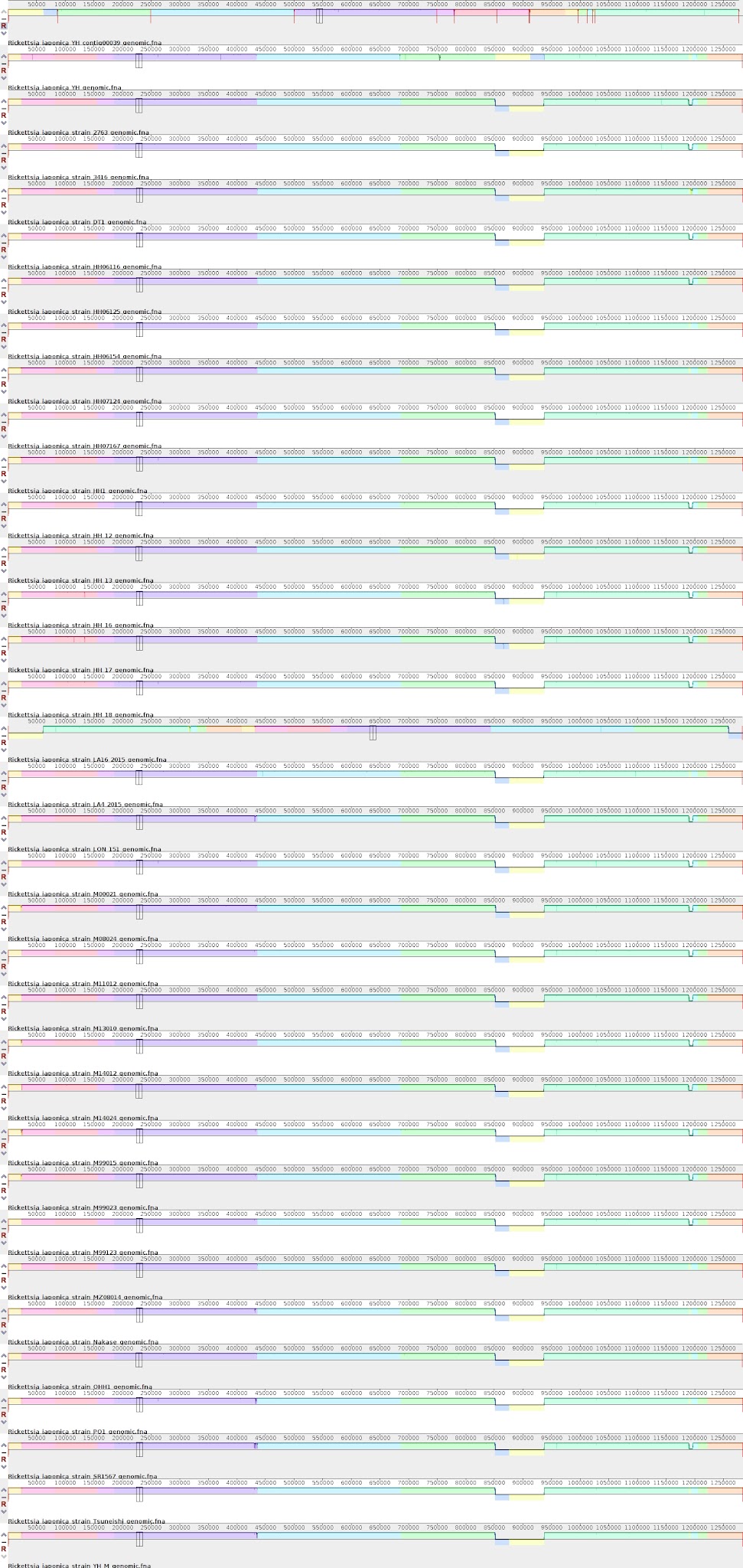
*

Source: By author, 2025. Note: Genomic alignment among genomes of *R. japonica*. The open bars observed in this figure represent software indicators associated with nucleotide residue positioning across samples, facilitating the visualization of potential translocated regions.

D5. Mauve Analysis of *R. parkeri*

*
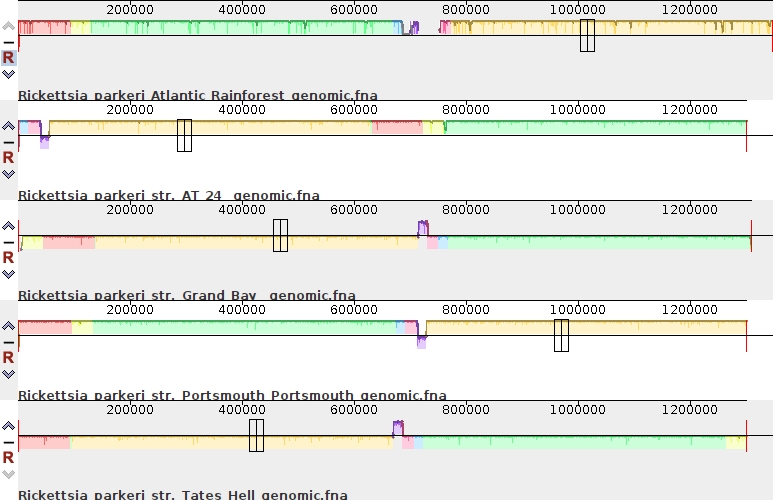
*

Source: By author, 2025. Note: Genomic alignment among genomes of *R. parkeri*. The open bars observed in this figure represent software indicators associated with nucleotide residue positioning across samples, facilitating the visualization of potential translocated regions.

D6. Mauve Analysis of *R. prowazekii*


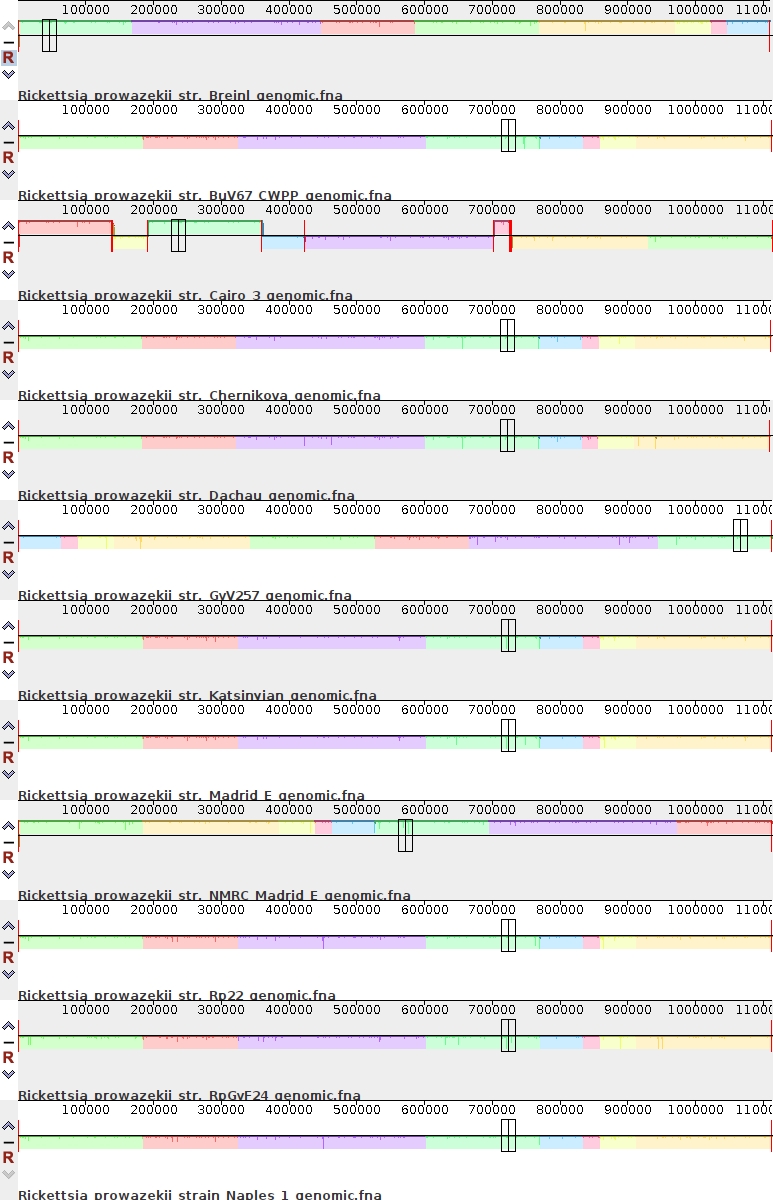


Source: By author, 2025. Note: Genomic alignment among genomes of *R. prowazekii*. The open bars observed in this figure represent software indicators associated with nucleotide residue positioning across samples, facilitating the visualization of potential translocated regions.

D7. Mauve Analysis of *R. rickettsii*
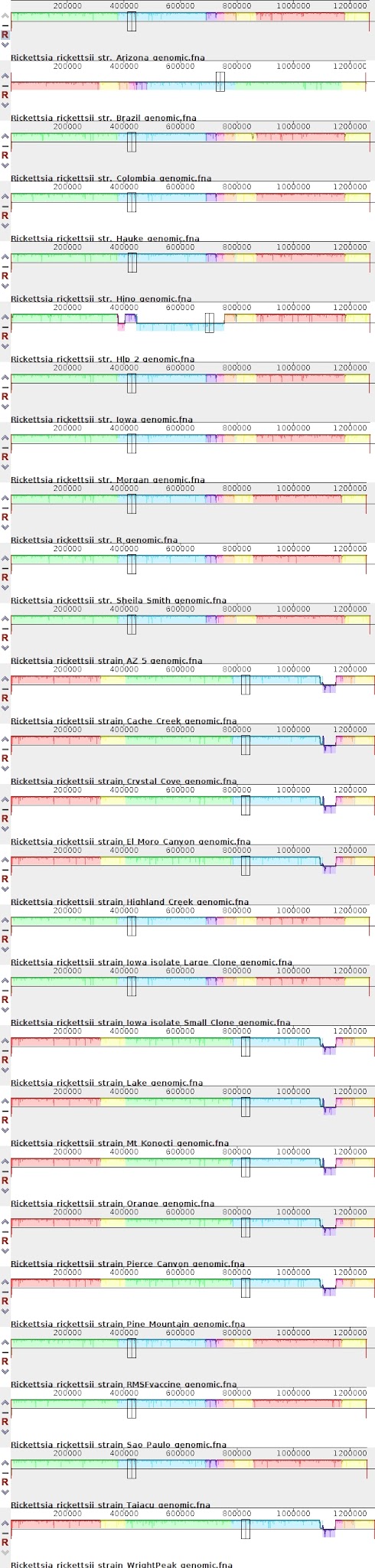


Source: By author, 2025. Note: Genomic alignment among genomes of *R. rickettsii*. The open bars observed in this figure represent software indicators associated with nucleotide residue positioning across samples, facilitating the visualization of potential translocated regions.

D8. Mauve Analysis of *R. sibirica*

*
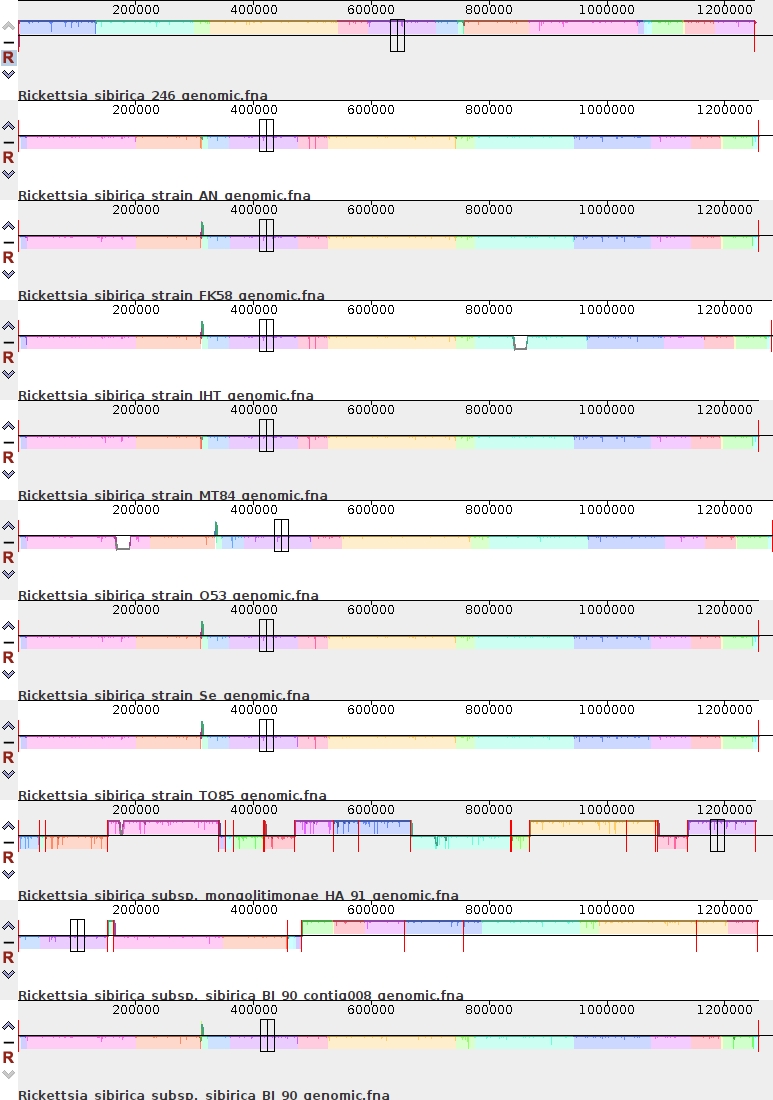
*

Source: By author, 2025. Note: Genomic alignment among genomes of *R. sibirica*. The open bars observed in this figure represent software indicators associated with nucleotide residue positioning across samples, facilitating the visualization of potential translocated regions.

# **SUPPLEMENTARY MATERIAL E - GENOMIC SIMILARITY**

E1. Heatmap of *R. amblyommatis*

*
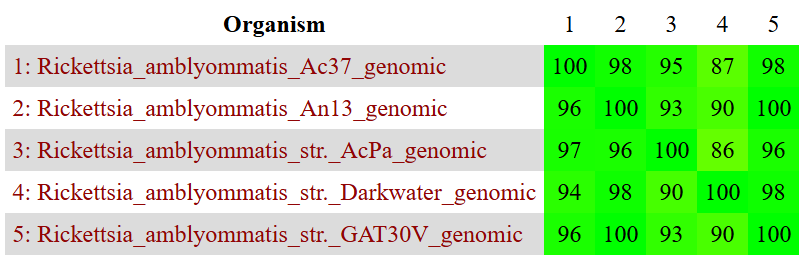
*

Source: By author, 2025. Note: Heatmap generated using the Gegenees software, showing the similarity matrix of *R. amblyommatis.*

E2. Heatmap of *R. bellii*


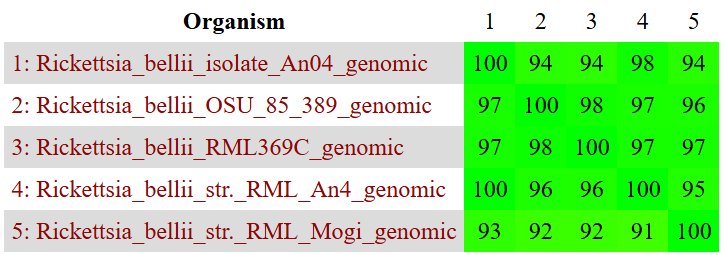


Source: By author, 2025. Note: Heatmap generated using the Gegenees software, showing the similarity matrix of *R. bellii*.

E3. Heatmap of *R. conorii*

*
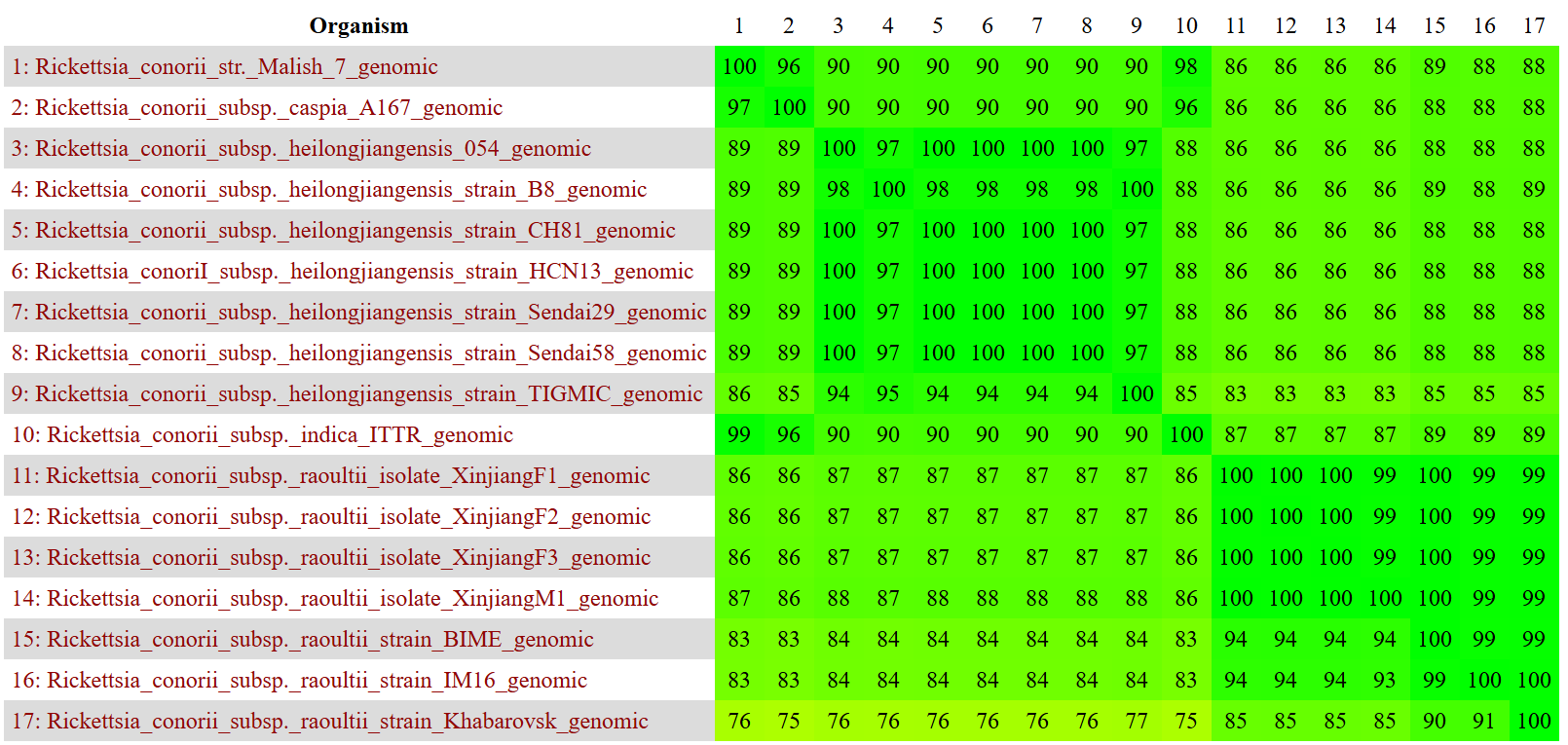
*

Source: By author, 2025. Note: Heatmap generated using the Gegenees software, showing the similarity matrix of *R. conorii*.

E4. Heatmap of *R. japonica*

*
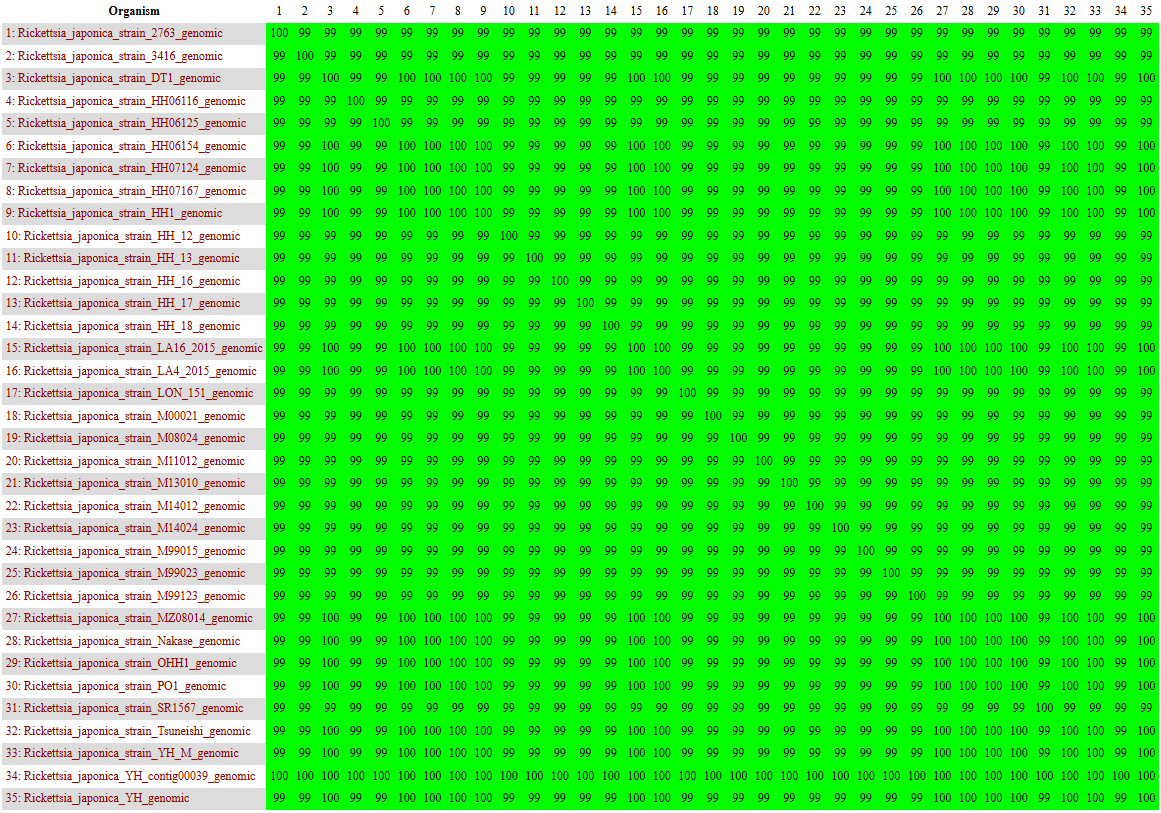
*

Source: By author, 2025. Note: Heatmap generated using the Gegenees software, showing the similarity matrix of *R. japonica*.

E5. Heatmap of *R. parkeri*

*
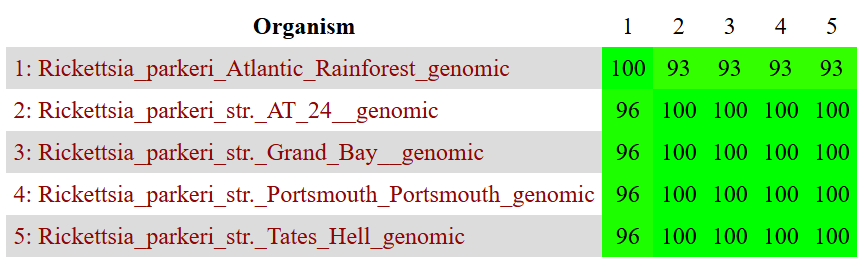
*

Source: By author, 2025. Note: Heatmap generated using the Gegenees software, showing the similarity matrix of *R. parkeri*.

E6. Heatmap of *R. prowazekii*


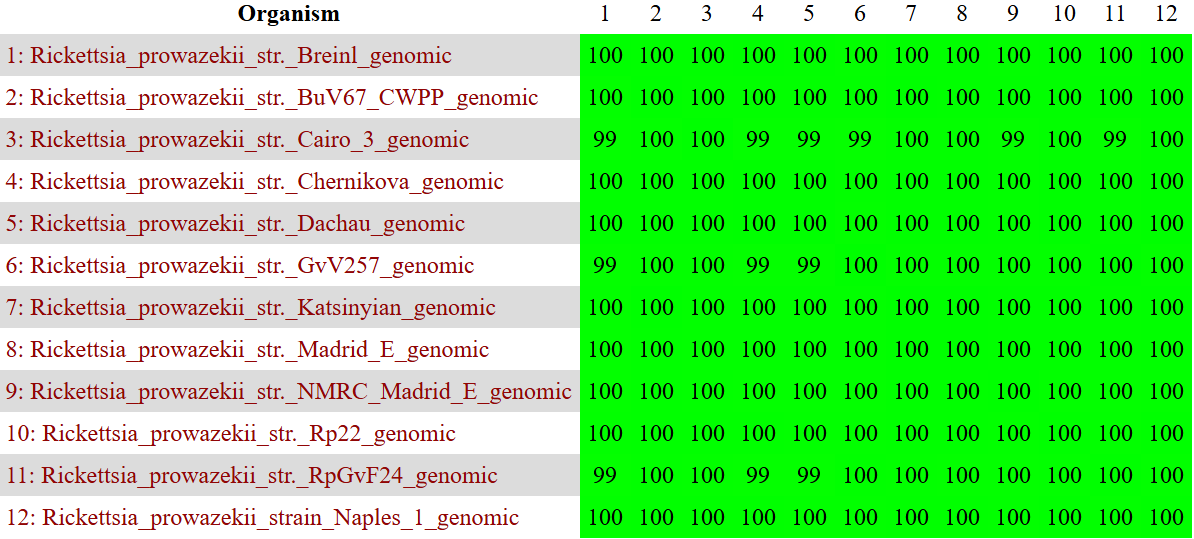


Source: By author, 2025. Note: Heatmap generated using the Gegenees software, showing the similarity matrix of *R. prowazekii*.

E7. Heatmap of *R. rickettsii*


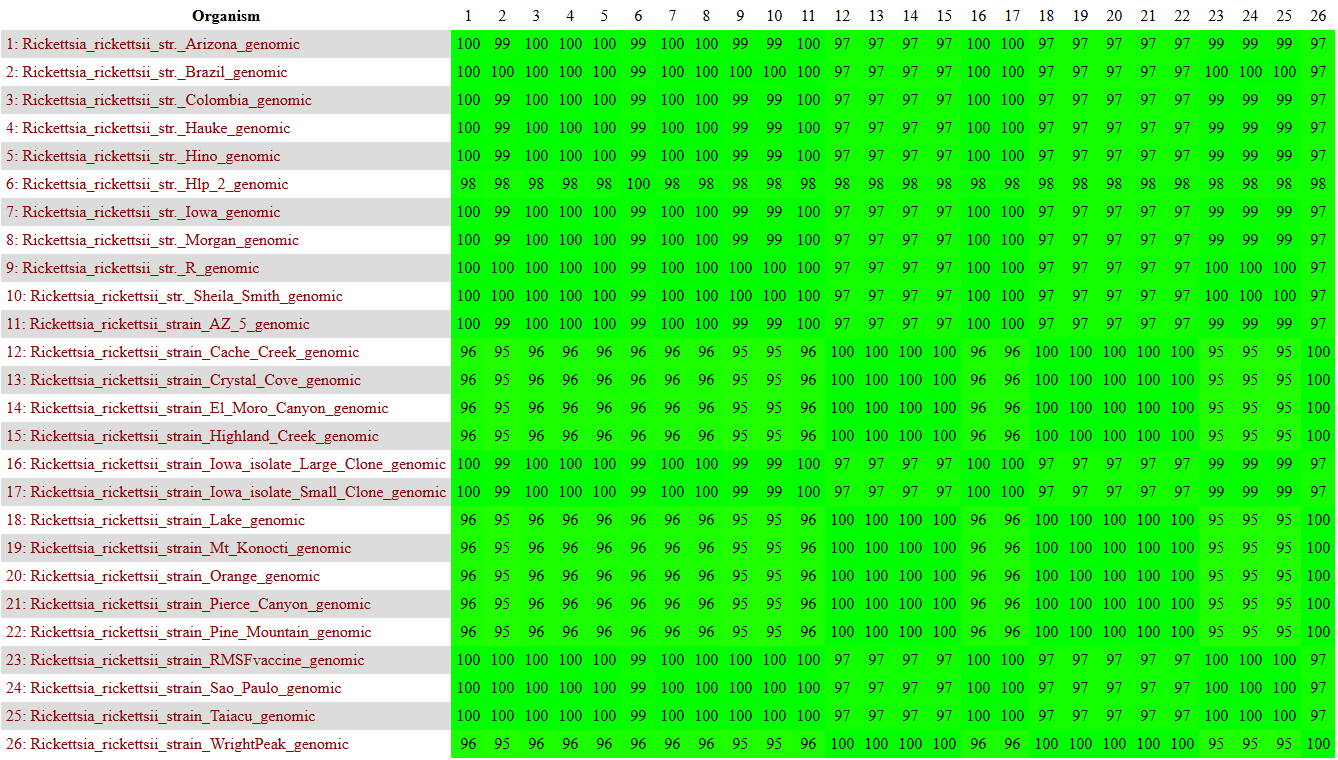


Source: By author, 2025. Note: Heatmap generated using the Gegenees software, showing the similarity matrix of *R. rickettsii*.

E8. Heatmap of *R. sibirica*

*
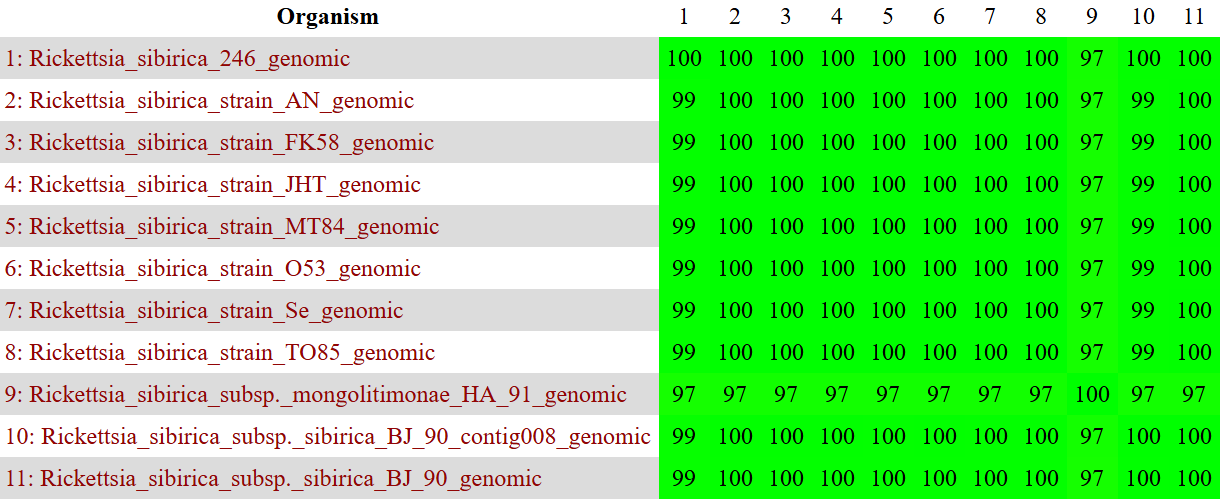
*

Source: By author, 2025. Note: Heatmap generated using the Gegenees software, showing the similarity matrix of *R. sibirica*.

# **SUPPLEMENTARY MATERIAL F - VIRULENCE AND RESISTANCE GENES**

#### Table 4 - Virulence-associated genes identified in *Rickettsia* genomes through VFDB screening

| **Name of the Specie str.** | **Gene** | **%Coverage** | **%Identity** |
| --- | --- | --- | --- |
| *Rickettsia conorii* str. Malish 7 | rickA | 100 | 100 |
| *Rickettsia rickettsii* str. Sheila Smith | ompA | 100 | 99,98 |
| *Rickettsia rickettsii* strain RMSFvaccine | ompA | 100 | 99,96 |
| *Rickettsia rickettsii* str. R | ompA | 100 | 99,9 |
| *Rickettsia rickettsii* strain AZ 5 | ompA | 100 | 99,69 |
| *Rickettsia rickettsii* str. Hauke | ompA | 100 | 99,66 |
| *Rickettsia rickettsii* str. Hino | ompA | 100 | 99,66 |
| *Rickettsia rickettsii* str. Morgan | ompA | 100 | 99,64 |
| *Rickettsia rickettsii* strain Taiacu | ompA | 100 | 99,57 |
| *Rickettsia rickettsii* strain Sao Paulo | ompA | 100 | 99,48 |
| *Rickettsia rickettsii* str. Hlp 2 | ompA | 100 | 99,2 |
| *Rickettsia conorii* subsp. indica ITTR | rickA | 98,78 | 97,71 |
| *Rickettsia parkeri* str. Tates Hell | rickA | 99,23 | 97,06 |
| *Rickettsia rickettsii* strain Cache Creek | rickA | 98,85 | 96,93 |
| *Rickettsia rickettsii* strain Lake | rickA | 98,85 | 96,93 |
| *Rickettsia rickettsii* strain Pine Mountain | rickA | 98,85 | 96,93 |
| *Rickettsia philipii* str. 364D 364D | rickA | 98,46 | 96,67 |
| *Rickettsia rickettsii* strain Crystal Cove | rickA | 98,46 | 96,67 |
| *Rickettsia rickettsii* strain El Moro Canyon | rickA | 98,46 | 96,67 |
| *Rickettsia rickettsii* strain Highland Creek | rickA | 98,46 | 96,67 |
| *Rickettsia rickettsii* strain Mt Konocti | rickA | 98,46 | 96,67 |
| *Rickettsia rickettsii* strain Orange | rickA | 98,46 | 96,67 |
| *Rickettsia rickettsii* strain Pierce Canyon | rickA | 98,46 | 96,67 |
| *Rickettsia rickettsii* strain WrightPeak | rickA | 98,46 | 96,67 |
| *Rickettsia parkeri* str. Grand Bay | rickA | 98,46 | 96,42 |
| *Rickettsia parkeri* str. Portsmouth Portsmouth | rickA | 100 | 95,97 |
| *Rickettsia sibirica* 246 | rickA | 99,23 | 95,63 |
| *Rickettsia sibirica* strain FK58 | rickA | 99,23 | 95,63 |
| *Rickettsia sibirica* strain JHT | rickA | 99,23 | 95,63 |
| *Rickettsia sibirica* strain MT84 | rickA | 99,23 | 95,63 |
| *Rickettsia sibirica* strain O53 | rickA | 99,23 | 95,63 |
| *Rickettsia sibirica* strain Se | rickA | 99,23 | 95,63 |
| *Rickettsia sibirica* strain TO85 | rickA | 99,23 | 95,63 |
| *Rickettsia sibirica* strain AN | rickA | 99,3 | 95,57 |
| *Rickettsia slovaca* 13 B | rickA | 98,46 | 95,2 |
| *Rickettsia slovaca* str. D CWPP | rickA | 98,46 | 95,2 |

Source: By author, 2025. Note: Virulence-associated genes identified through screening against the VFDB database, showing the species/strain, gene name, percentage of coverage, and percentage of sequence identity. Only hits with at least 95% sequence identity and 95% coverage were retained. The "-" sign represents fields for which information was not available or not applicable.

####

#

# **SUPPLEMENTARY MATERIAL G - IDENTIFICATION OF MOBILE ELEMENTS**

#### Table 5 - Mobile Genetic Elements Identified in the Rickettsia Genus

| **Species** | **Subsp.** | **Strain** | **Type of MGE** | **MGE name** | **Identity** | **Contig Number** | **Protein Product's Annotation** |
| --- | --- | --- | --- | --- | --- | --- | --- |
| *Rickettsia argasii* | - | T170-B | insertion sequence | ISRpe1 | 0,968779 | NZ_LAOQ01000008.1 | IS481 family transposase |
| *Rickettsia bellii* | - | An04 | composite transposon | cn_13857_ISRpe1 | 0,940497 | NZ_CP015011.1 | hypothetical protein/protein_id="WP_155980556.1, WP_052692917.1, WP_155980557.1, WP_269208699.1"; IS481 family transposase/protein_id="WP_057700078.1, WP_155980557.1, WP_269208699.1"; Rpn family recombination-promoting nuclease/putative transposase/protein_id="WP_081178597.1"; helix-turn-helix domain-containing protein/protein_id="WP_081178599.1"; |
| *Rickettsia bellii* | - | An04 | insertion sequence | ISRpe1 | 0,940497 | NZ_CP015011.1 | IS481 family transposase/protein_id="WP_057700078.1" |
| *Rickettsia bellii* | - | An04 | insertion sequence | ISRpe1 | 0,940497 | NZ_CP015011.1 | hypothetical protein/protein_id="WP_155980556.1"; IS481 family transposase/protein_id="WP_057700078.1" |
| *Rickettsia bellii* | - | RML Mogi | composite transposon | cn_46648_ISRpe1 | 0,947649 | NZ_LAOJ01000001.1 | IS481 family transposase; BRO-N domain-containing protein/proteinid="WP011477226.1"; hypothetical protein/proteinid="WP012151779.1, WP231569862.1, WP045799583.1, WP045799584.1, WP012151780.1, WP231569861.1, WP052691000.1, WP011477245.1, WP231289229.1, WP011477251.1, WP045798812.1, WP045799588.1, WP231569864.1, WP231289228.1, WP011477265.1, WP012151804.1, WP269429607.1, WP269207861.1"; helix-turn-helix domain-containing protein/proteinid="WP011477227.1"; twin transmembrane helix small protein/proteinid="WP011477228.1"; Fe-S cluster assembly protein IscX/proteinid="WP011477229.1"; peptide deformylase/proteinid="WP045798798.1"; methionyl-tRNA formyltransferase/proteinid="WP045799581.1"; IS256 family transposase; glycosyltransferase family 61 protein/proteinid="WP045799936.1"; cell division protein ZapE/proteinid="WP045798800.1"; HD domain-containing protein/proteinid="WP231569860.1"; MFS transporter/proteinid="WP331385707.1"; sugar phosphate nucleotidyltransferase/proteinid="WP045799582.1"; YebC/PmpR family DNA-binding transcriptional regulator/proteinid="WP045798802.1"; type B 50S ribosomal protein L36/proteinid="WP004998227.1"; lytic transglycosylase domain-containing protein/proteinid="WP011477242.1, WP045799940.1"; DUF2532 domain-containing protein/proteinid="WP012151790.1"; methyltransferase domain-containing protein/proteinid="WP081423319.1"; dihydrolipoyl dehydrogenase/proteinid="WP045799585.1"; LD-carboxypeptidase/proteinid="WP045799586.1"; ankyrin repeat domain-containing protein/proteinid="WP045799580.1, WP231569863.1, WP011477258.1"; tetratricopeptide repeat protein/proteinid="WP231569865.1"; chaperonin GroEL/proteinid="WP011477259.1"; co-chaperone GroES/proteinid="WP011477260.1"; bifunctional (p)ppGpp synthetase/guanosine-3',5'-bis(diphosphate) 3'-pyrophosphohydrolase/proteinid="WP012151800.1"; HD domain-containing protein; fibronectin type III domain-containing protein/proteinid="WP082068162.1"; glycoside hydrolase family 18 protein/proteinid="WP231569866.1" |
| *Rickettsia bellii* | - | RML Mogi | composite transposon | cn_29905_ISRpe1 | 0,948582 | NZ_LAOJ01000001.1 | IS481 family transposase; hypothetical protein/protein_id="WP_231569867.1, WP_269208688.1, WP_231569870.1, WP_012151820.1, WP_231569871.1, WP_231569872.1"; peptide chain release factor 2/protein_id="WP_011477267.1"; translation elongation factor 4/protein_id="WP_012151806.1"; ankyrin repeat domain-containing protein/protein_id="WP_331385711.1"; isoleucine--tRNA ligase/protein_id="WP_045799589.1"; IS110 family RNA-guided transposase/protein_id="WP_011476926.1"; queuosine precursor transporter/protein_id="WP_012151809.1"; AbrB/MazE/SpoVT family DNA-binding domain-containing protein/protein_id="WP_012151810.1"; type II toxin-antitoxin system VapC family toxin/protein_id="WP_045799590.1"; AI-2E family transporter/protein_id="WP_012151811.1"; nucleotide exchange factor GrpE/protein_id="WP_011477275.1"; ribonuclease PH/protein_id="WP_011477276.1"; TlyA family RNA methyltransferase/protein_id="WP_045799591.1"; Rrf2 family transcriptional regulator/protein_id="WP_011477278.1"; globin domain-containing protein/protein_id="WP_231569869.1"; ribosome maturation factor RimP/protein_id="WP_041804650.1"; transcription termination factor NusA/protein_id="WP_045799592.1"; translation initiation factor IF-2/protein_id="WP_045799593.1"; excinuclease ABC subunit UvrC/protein_id="WP_045798823.1"; leucine-rich repeat domain-containing protein/protein_id="WP_045799594.1" |
| *Rickettsia bellii* | - | RML Mogi | composite transposon | cn_6865_ISRpe1 | 0,945084 | NZ_LAOJ01000001.1 | IS481 family transposase; hypothetical protein/protein_id="WP_197067230.1, WP_045799648.1"; transcriptional regulator/protein_id="WP_231289304.1"; flavodoxin family protein; IS110 family transposase/protein_id="WP_045799647.1"; IS256 family transposase/protein_id="WP_052692913.1"; tetratricopeptide repeat protein/protein_id="WP_082068138.1" |
| *Rickettsia bellii* | - | RML Mogi | composite transposon | cn_14284_ISRpe1 | 0,946761 | NZ_LAOJ01000001.1 | IS481 family transposase; tetratricopeptide repeat protein/protein_id="WP_082068138.1, WP_082068139.1"; lipase family protein/protein_id="WP_156145456.1"; A1G_07140 family DUF167 domain protein/protein_id="WP_011477302.1"; molecular chaperone HtpG/protein_id="WP_045798902.1"; 5-aminolevulinate synthase/protein_id="WP_011477300.1"; hypothetical protein/protein_id="WP_231569882.1, WP_012151912.1"; class I SAM-dependent methyltransferase/protein_id="WP_052692887.1"; GNAT family N-acetyltransferase/protein_id="WP_231569883.1"; ankyrin repeat domain-containing protein/protein_id="WP_045799650.1"; IS481 family transposase |
| *Rickettsia bellii* | - | RML Mogi | composite transposon | cn_5871_ISRpe1 | 0,949378 | NZ_LAOJ01000001.1 | IS481 family transposase/protein_id="WP_045799839.1"; DNA replication/repair protein RecF/protein_id="WP_012151492.1"; dihydrofolate reductase/protein_id="WP_011476729.1"; dihydropteroate synthase/protein_id="WP_011476728.1"; dihydroneopterin aldolase/protein_id="WP_082068169.1" |
| *Rickettsia bellii* | - | RML Mogi | composite transposon | cn_25482_ISRpe1 | 0,947695 | NZ_LAOJ01000001.1 | IS481 family transposase; alanine--tRNA ligase/protein_id="WP_045799840.1"; hypothetical protein/protein_id="WP_231569952.1, WP_156145463.1, WP_082068151.1, WP_045799842.1"; RP853 family protein/protein_id="WP_011476764.1"; NlpC/P60 family protein/protein_id="WP_197067235.1"; SH3 domain-containing protein/protein_id="WP_052692905.1"; MFS transporter/protein_id="WP_011476762.1"; palindromic element RPE5 domain-containing protein; Rpn family recombination-promoting nuclease/putative transposase/protein_id="WP_045799843.1"; epoxyqueuosine reductase QueH/protein_id="WP_045799844.1"; nucleotidyltransferase and HEPN domain-containing protein/protein_id="WP_012151531.1"; tRNA pseudouridine(38-40) synthase TruA/protein_id="WP_045799845.1"; N-6 DNA methylase/protein_id="WP_269208690.1, WP_045799846.1"; RNA polymerase sigma factor RpoD/protein_id="WP_045799847.1"; DNA primase/protein_id="WP_011476751.1"; diacylglycerol/polyprenol kinase family protein/protein_id="WP_011476750.1"; transcription elongation factor GreA/protein_id="WP_011476749.1"; lipoyl(octanoyl) transferase LipB/protein_id="WP_045799848.1"; YdcF family protein/protein_id="WP_011476747.1"; Rpn family recombination-promoting nuclease/putative transposase hypothetical protein/protein_id="WP_045799849.1" |
| *Rickettsia bellii* | - | RML Mogi | composite transposon | cn_18516_ISRpe1 | 0,948490 | NZ_LAOJ01000001.1 | IS481 family transposase; YbaB/EbfC family nucleoid-associated protein/protein_id="WP_045799850.1"; DNA polymerase III subunit gamma/tau/protein_id="WP_011476743.1"; signal recognition particle sRNA small type outer-membrane lipoprotein carrier protein LolA/protein_id="WP_011476742.1"; NAD(P) transhydrogenase subunit alpha/protein_id="WP_011476741.1"; NAD(P) transhydrogenase subunit alpha part 2/protein_id="WP_011476740.1"; hypothetical protein/protein_id="WP_231569954.1, WP_012151542.1"; MFS transporter/protein_id="WP_045799136.1"; cell division protein ZapA/protein_id="WP_011476737.1"; helix-turn-helix domain-containing protein/protein_id="WP_045799138.1"; NAD-glutamate dehydrogenase/protein_id="WP_045799851.1"; tRNA uridine-5-carboxymethylaminomethyl(34) synthesis GTPase MnmE/protein_id="WP_011476733.1"; tetratricopeptide repeat protein/protein_id="WP_045799852.1"; IS110 family transposase |
| *Rickettsia bellii* | - | RML Mogi | composite transposon | cn_13465_ISRpe1 | 0,947695 | NZ_LAOJ01000001.1 | IS481 family transposase; hypothetical protein/protein_id="WP_231569956.1, WP_231569957.1"; NACHT domain-containing protein/protein_id="WP_045799867.1"; trigger factor/protein_id="WP_011478013.1"; type II toxin-antitoxin system Phd/YefM family antitoxin; PIN domain-containing protein; glycine--tRNA ligase subunit alpha/protein_id="WP_045799868.1"; glycine--tRNA ligase subunit beta/protein_id="WP_045799869.1"; L-threonylcarbamoyladenylate synthase/protein_id="WP_045799870.1"; |
| *Rickettsia bellii* | - | RML Mogi | composite transposon | cn_14704_ISRpe1 | 0,948582 | NZ_LAOJ01000001.1 | IS481 family transposase; hypothetical protein/protein_id="WP_011478007.1"; 50S ribosomal protein L11 methyltransferase/protein_id="WP_011478006.1"; ATP/ADP exchange transporter Tlc1/protein_id="WP_045799871.1"; MFS transporter/protein_id="WP_045799872.1"; nucleoside-diphosphate kinase/protein_id="WP_011478003.1"; tRNA uridine-5-carboxymethylaminomethyl(34) synthesis enzyme MnmG/protein_id="WP_011478002.1"; 16S rRNA (guanine(527)-N(7))-methyltransferase RsmG/protein_id="WP_045799116.1"; ParA family protein/protein_id="WP_011478000.1"; ParB/RepB/Spo0J family partition protein/protein_id="WP_041804950.1"; energy-dependent translational throttle protein EttA/protein_id="WP_045799873.1"; DUF2672 domain-containing protein/protein_id="WP_011477997.1"; preprotein translocase subunit SecG/protein_id="WP_011477996.1"; 3-deoxy-8-phosphooctulonate synthase/protein_id="WP_011477995.1"; tRNA-binding protein/protein_id="WP_011477994.1" |
| *Rickettsia bellii* | - | RML Mogi | composite transposon | cn_4502_ISRpe1 | 0,948490 | NZ_LAOJ01000001.1 | IS481 family transposase; Rpn family recombination-promoting nuclease/putative transposase/protein_id="WP_045799113.1"; DHHA1 domain-containing protein; hypothetical protein/protein_id="WP_045799875.1" |
| *Rickettsia bellii* | - | RML Mogi | composite transposon | cn_19194_ISRpe1 | 0,946809 | NZ_LAOJ01000001.1 | IS481 family transposase; DUF5394 family protein/protein_id="WP_011476727.1"; tetratricopeptide repeat protein/protein_id="WP_231569958.1"; hypothetical protein/protein_id="WP_012151485.1, WP_231569959.1"; SCO family protein signal recognition particle-docking protein; FtsY/protein_id="WP_011476724.1"; YqgE/AlgH family protein/protein_id="WP_011476723.1"; CvpA family protein/protein_id="WP_011476722.1"; cell cycle transcriptional regulator TrcR/protein_id="WP_011476721.1"; NAD(P)(+) transhydrogenase (Re/Si-specific) subunit beta/protein_id="WP_045799876.1"; IS110 family RNA-guided transposase/protein_id="WP_045799512.1"; OmpW family outer membrane protein/protein_id="WP_011476718.1"; DMT family transporter/protein_id="WP_045799877.1"; MFS transporter/protein_id="WP_011476716.1"; BolA/IbaG family iron-sulfur metabolism protein/protein_id="WP_011476715.1"; ribosome silencing factor/protein_id="WP_011476714.1"; monovalent cation:proton antiporter-2 (CPA2) family protein/protein_id="WP_045799878.1"; SH3 domain-containing protein/protein_id="WP_011476712.1" |
| *Rickettsia bellii* | - | RML Mogi | composite transposon | cn_26447_ISRpe1 | 0,948582 | NZ_LAOJ01000001.1 | IS481 family transposase; hypothetical protein/protein_id="WP_045799882.1, WP_012151558.1, WP_045799879.1"; DUF5510 family protein/protein_id="WP_011477992.1"; ABC transporter ATP-binding protein/protein_id="WP_012151553.1"; MlaE family ABC transporter permease/protein_id="WP_011477990.1"; alanine racemase/protein_id="WP_045799880.1"; IS110 family RNA-guided transposase/protein_id="WP_045799512.1, WP_011476926.1"; uracil-DNA glycosylase family protein/protein_id="WP_045799881.1"; RluA family pseudouridine synthase/protein_id="WP_011477901.1"; type II toxin-antitoxin system Phd/YefM family antitoxin/protein_id="WP_011477900.1"; type II toxin-antitoxin system VapC family toxin/protein_id="WP_012151561.1"; bifunctional peptide chain release factor N(5)-glutamine methyltransferase PrmC/tRNA (guanosine(46)-N7)-methyltransferase TrmB; MlaC/ttg2D family ABC transporter substrate-binding protein/protein_id="WP_012151559.1"; MlaA family lipoprotein/protein_id="WP_011477981.1"; methyltransferase domain-containing protein/protein_id="WP_011477982.1"; pyridoxal phosphate-dependent aminotransferase/protein_id="WP_011477983.1"; lysophospholipid acyltransferase family protein/protein_id="WP_011477984.1"; lipid IV(A) 3-deoxy-D-manno-octulosonic acid transferase/protein_id="WP_012151556.1"; Sca4 family protein/protein_id="WP_052692906.1"; ribose-phosphate diphosphokinase/protein_id="WP_045799883.1"; heme ABC transporter permease/protein_id="WP_045799081.1"; ferredoxin FdxA/protein_id="WP_011477924.1"; adhesin Adr/protein_id="WP_045799884.1"; outer membrane protein/protein_id="WP_045799885.1"; GTPase/protein_id="WP_052692908.1" |
| *Rickettsia bellii* | - | RML Mogi | composite transposon | cn_27660_ISRpe1 | 0,946018 | NZ_LAOJ01000001.1 | IS481 family transposase; IS110 family transposase; phosphate acetyltransferase/protein_id="WP_045799093.1"; acetate/propionate family kinase; AbrB/MazE/SpoVT family DNA-binding domain-containing protein/protein_id="WP_011477870.1, WP_011477886.1"; type II toxin-antitoxin system VapC family toxin/protein_id="WP_045799887.1, WP_012151577.1"; ankyrin repeat domain-containing protein/protein_id="WP_012151584.1, WP_231569960.1"; DMP19 family protein/protein_id="WP_012151585.1"; tRNA (guanine(37)-N(1))-methyltransferase/protein_id="WP_331370631.1"; 50S ribosomal protein L19/protein_id="WP_011477875.1"; hypothetical protein/protein_id="WP_011477878.1, WP_228368737.1, WP_011477881.1, WP_231569961.1"; penicillin-binding protein 2/protein_id="WP_045799888.1"; NAD(P)H-dependent glycerol-3-phosphate dehydrogenase/protein_id="WP_011477882.1"; succinate--CoA ligase subunit alpha/protein_id="WP_011477883.1"; ADP-forming succinate--CoA ligase subunit beta/protein_id="WP_011477884.1"; cytochrome b/protein_id="WP_082068157.1"; S9 family peptidase/protein_id="WP_045799889.1"; Tim44 domain-containing protein/protein_id="WP_011477890.1"; 30S ribosome-binding factor RbfA/protein_id="WP_011477891.1"; YqaA family protein/protein_id="WP_011477892.1"; RDD family protein/protein_id="WP_011477893.1"; recombination mediator RecR/protein_id="WP_011477894.1" |
| *Rickettsia bellii* | - | RML Mogi | composite transposon | cn_39977_ISRpe1 | 0,945084 | NZ_LAOJ01000001.1 | IS481 family transposase/protein_id="WP_045799898.1"; hypothetical protein/protein_id="WP_231569964.1, WP_231569965.1, WP_045799891.1, WP_231569961.1, WP_231569962.1"; RP439 family protein; NAD kinase/protein_id="WP_012151570.1"; palindromic element RPE5 domain-containing protein; ribonuclease J/protein_id="WP_045799890.1"; MFS transporter; enoyl-ACP reductase FabI/protein_id="WP_011477908.1"; apolipoprotein N-acyltransferase/protein_id="WP_011477909.1"; helix-turn-helix domain-containing protein/protein_id="WP_228368738.1"; tRNA guanosine(34) transglycosylase Tgt/protein_id="WP_045799893.1"; type II toxin-antitoxin system Phd/YefM family antitoxin/protein_id="WP_011477912.1"; tRNA (adenosine(37)-N6)-threonylcarbamoyltransferase complex transferase subunit TsaD/protein_id="WP_011477913.1"; type IV secretion system protein/protein_id="WP_045799894.1"; TrbL/VirB6 family protein/protein_id="WP_045799895.1, WP_045799896.1, WP_045799085.1, WP_045799897.1"; VirB4 family type IV secretion/conjugal transfer ATPase/protein_id="WP_011477919.1"; IS110 family RNA-guided transposase/protein_id="WP_045799512.1"; DNA polymerase I/protein_id="WP_011477866.1" |
| *Rickettsia bellii* | - | RML Mogi | insertion sequence | ISRpe1 | 0,945084 | NZ_LAOJ01000001.1 | IS481 family transposase; hypothetical protein/protein_id="WP_231569961.1" |
| *Rickettsia bellii* | - | RML Mogi | insertion sequence | ISRpe1 | 0,945922; 0,946018; 0,946761; 0,946809; 0,947649 | NZ_LAOJ01000001.1 | IS481 family transposase; GTPase/protein_id="WP_052692908.1"; tetratricopeptide repeat protein/protein_id="WP_082068138.1"; hypothetical protein/protein_id="WP_269429609.1" |
| *Rickettsia bellii* | - | RML Mogi | insertion sequence | ISRpe1 | 0,947695; 0,94849; 0,948582; 0,949378; 0,947272; 0,949378 | NZ_LAOJ01000001.1 | hypothetical protein/protein_id="WP_231569956.1"; IS481 family transposase/protein_id="WP_045799839.1, WP_045799898.1"; IS110 family transposase; S1C family serine protease/protein_id="WP_045799499.1" |
| *Rickettsia felis* | - | LSU | composite transposon | cn_5316_ISRpe1 | 0,929076 | NZ_JSEM01000022.1 | IS256 family transposase; hypothetical protein/protein_id="WP_236682742.1"; Hsp20/alpha crystallin family protein/protein_id="WP_039595634.1"; Hsp20/alpha crystallin family protein/protein_id="WP_039595613.1" |
| *Rickettsia felis* | - | LSU | insertion sequence | ISRpe1 | 0,929076 | NZ_JSEM01000022.1 | IS481 family transposase |
| *Rickettsia fournieri* | - | AUS118 | composite transposon | cn_39541_ISRpe1 | 0,977698 | NZ_LT978482.1 | helix-turn-helix domain-containing protein/protein_id="WP_198913009.1"; IS256 family transposase; transposase/protein_id="WP_209445172.1"; ABC transporter permease; ABC transporter substrate binding protein; N-acetylmuramoyl-L-alanine amidase; hypothetical protein/protein_id="WP_198913010.1, WP_245207373.1, WP_103896942.1, WP_245207375.1, WP_245207376.1"; apolipoprotein N-acyltransferase/protein_id="WP_103896835.1"; enoyl-ACP reductase FabI/protein_id="WP_045805466.1"; DUF5410 family protein/protein_id="WP_103896836.1"; site-specific tyrosine recombinase XerD/protein_id="WP_103896837.1"; phage terminase large subunit/protein_id="WP_245207372.1"; DUF2155 domain-containing protein/protein_id="WP_004995979.1"; O-antigen ligase family protein/protein_id="WP_103896838.1"; NADH-quinone oxidoreductase subunit A/protein_id="WP_014014184.1"; NuoB/complex I 20 kDa subunit family protein/protein_id="WP_004995988.1"; NADH-quinone oxidoreductase subunit C/protein_id="WP_103896839.1"; NADH dehydrogenase (quinone) subunit D/protein_id="WP_103896840.1"; GIY-YIG nuclease family protein/protein_id="WP_103896841.1"; NADH-quinone oxidoreductase subunit NuoE/protein_id="WP_016926163.1"; membrane protein insertion efficiency factor YidD/protein_id="WP_103896842.1"; DNA-3-methyladenine glycosylase/protein_id="WP_103896940.1"; bestrophin-like domain; CopG family antitoxin/protein_id="WP_103896843.1"; toxin/protein_id="WP_103896844.1"; exodeoxyribonuclease VII small subunit/protein_id="WP_014014175.1"; AsmA-like C-terminal region-containing protein/protein_id="WP_103896941.1"; ribosome maturation factor RimM/protein_id="WP_103896845.1"; AsmA family protein; DUF4385 family protein/protein_id="WP_012737014.1"; spore photoproduct lyase family protein/protein_id="WP_103896847.1"; heme o synthase/protein_id="WP_014120700.1"; 30S ribosomal protein S4/protein_id="WP_103896943.1"; ATP-binding cassette domain-containing protein/protein_id="WP_245207377.1"; glycosyltransferase family 4 protein/protein_id="WP_103896848.1"; alpha/beta hydrolase/protein_id="WP_014014163.1"; DUF2608 domain-containing protein/protein_id="WP_103896849.1"; efflux RND transporter periplasmic adaptor subunit; IS481 family transposase; type IV secretion system DNA-binding domain-containing protein |
| *Rickettsia fournieri* | - | AUS118 | composite transposon | cn_18743_ISRpe1 | 0,978591 | NZ_LT978482.1 | N-acetylmuramoyl-L-alanine amidase; hypothetical protein/protein_id="WP_198913010.1, WP_245207373.1"; apolipoprotein N-acyltransferase/protein_id="WP_103896835.1"; enoyl-ACP reductase FabI/protein_id="WP_045805466.1"; DUF5410 family protein/protein_id="WP_103896836.1"; site-specific tyrosine recombinase XerD/protein_id="WP_103896837.1"; phage terminase large subunit/protein_id="WP_245207372.1"; DUF2155 domain-containing protein/protein_id="WP_004995979.1"; O-antigen ligase family protein/protein_id="WP_103896838.1"; NADH-quinone oxidoreductase subunit A/protein_id="WP_014014184.1"; NuoB/complex I 20 kDa subunit family protein/protein_id="WP_004995988.1"; NADH-quinone oxidoreductase subunit C/protein_id="WP_103896839.1"; NADH dehydrogenase (quinone) subunit D/protein_id="WP_103896840.1"; GIY-YIG nuclease family protein/protein_id="WP_103896841.1"; NADH-quinone oxidoreductase subunit NuoE/protein_id="WP_016926163.1"; membrane protein insertion efficiency factor YidD/protein_id="WP_103896842.1"; DNA-3-methyladenine glycosylase/protein_id="WP_103896940.1"; bestrophin-like domain; CopG family antitoxin/protein_id="WP_103896843.1"; toxin/protein_id="WP_103896844.1"; exodeoxyribonuclease VII small subunit/protein_id="WP_014014175.1"; AsmA-like C-terminal region-containing protein/protein_id="WP_103896941.1" |
| *Rickettsia fournieri* | - | AUS118 | composite transposon | cn_2871_ISRpe1; cn_12025_ISRpe1; cn_20767_ISRpe1 | 0,978591 | NZ_LT978482.1 |  |
| *Rickettsia fournieri* | - | AUS118 | composite transposon | cn_22976_ISRpe1; cn_13993_ISRpe1 | 0,978591 | NZ_LT978482.1 |  |
| *Rickettsia fournieri* | - | AUS118 | insertion sequence | ISRpe1 | 0,977698 | NZ_LT978482.1 | IS481 family transposase; type IV secretion system DNA-binding  domain-containing protein |
| *Rickettsia fournieri* | - | AUS118 | insertion sequence | ISRpe1 | 0,978591 | NZ_LT978482.1 | AsmA-like C-terminal region-containing protein/protein_id="WP_103896941.1"; |
| *Rickettsia gravesii* | - | BWI-1 | insertion sequence | ISRpe1 | 0,938163 | NZ_AWXL01000014.1 | IS481 family transposase |
| *Rickettsia massiliae* | - | MTU5 | insertion sequence | ISRpe1 | 0,952043 | NC_009900.1 | IS481 family transposase /protein_id="WP_041404634.1"; ATP-binding protein/protein_id="WP_049749363.1" |
| *Rickettsia monacensis* | - | IrR/Munich | composite transposon | cn_25069_ISOt3 | 0,903602 | NZ_LN794217.1 | IS630 family transposase; glycoside hydrolase family 3 N-terminal domain-containing protein/protein_id="WP_023507101.1"; MerR family transcriptional regulator; transposase/protein_id="WP_023507102.1, WP_023507103.1, WP_052701310.1, WP_046058374.1"; hypothetical protein/protein_id="WP_008580348.1, WP_023507105.1, WP_023507118.1"; threonine--tRNA ligase/protein_id="WP_023507104.1"; Dabb family protein/protein_id="WP_096001052.1"; phosphoribosylaminoimidazolesuccinocarboxamide synthase/protein_id="WP_023507106.1"; M16 family metallopeptidase/protein_id="WP_023507107.1"; glycosyltransferase family 2 protein/protein_id="WP_023507108.1"; cytochrome d ubiquinol oxidase subunit II/protein_id="WP_023507109.1"; cytochrome ubiquinol oxidase subunit I/protein_id="WP_023507110.1"; DUF2671 domain-containing protein/protein_id="WP_023507111.1"; ABC transporter ATP-binding protein/protein_id="WP_023507112.1"; IS481 family transposase; cell division protein ZapE/protein_id="WP_023507114.1"; nucleotidyltransferase substrate binding protein/protein_id="WP_023507115.1"; nucleotidyltransferase domain-containing protein/protein_id="WP_023507116.1"; tRNA preQ1(34) S-adenosylmethionine ribosyltransferase-isomerase QueA/protein_id="WP_023507117.1"; glycosyltransferase family 61 protein |
| *Rickettsia monacensis* | - | IrR/Munich | composite transposon |  | 0,904661 | NZ_LN794217.1 | integrase core domain-containing protein; IS630 family transposase; IS481 family transposase; ankyrin repeat domain-containing protein/protein_id="WP_023507884.1, WP_096001029.1, WP_023507901.1"; hypothetical protein/protein_id="WP_242402802.1, WP_269429013.1, WP_023507900.1, WP_242402803.1, WP_008580410.1, WP_037231493.1, WP_023507902.1, WP_023507904.1, WP_157699831.1"; HD domain-containing protein; transposase/protein_id="WP_023507103.1, WP_023507102.1"; magnesium transporter/protein_id="WP_023507890.1"; TIGR01459 family HAD-type hydrolase/protein_id="WP_023507891.1"; DNA topoisomerase (ATP-hydrolyzing) subunit B/protein_id="WP_023507892.1"; UDP-N-acetylglucosamine 1-carboxyvinyltransferase/protein_id="WP_023507893.1"; DNA-directed RNA polymerase subunit omega/protein_id="WP_023507894.1"; holo-ACP synthase/protein_id="WP_023507895.1"; foldase protein PrsA/protein_id="WP_023507896.1"; preprotein translocase subunit SecA/protein_id="WP_023507897.1"; nucleotidyltransferase domain-containing protein/protein_id="WP_023507898.1"; HEPN domain-containing protein/protein_id="WP_023507899.1"; IS982 family transposase/protein_id="WP_339325424.1" |
| *Rickettsia monacensis* | - | IrR/Munich | insertion sequence | ISOt3 | 0,904762 | NZ_LN794217.1 | transposase/protein_id="WP_096001018.1"; IS630 family transposase |
| *Rickettsia monacensis* | - | IrR/Munich | insertion sequence | ISOt3 | 0,903602 | NZ_LN794217.1 | IS630 family transposase |
| *Rickettsia monacensis* | - | IrR/Munich | insertion sequence | ISOt3 | 0,904661 | NZ_LN794217.1 | integrase core domain-containing protein; IS630 family transposase |
| *Rickettsia monacensis* | - | IrR/Munich | insertion sequence | ISOt3 | 0,908898 | NZ_LN794217.1 | IS630 family transposase |
| *Rickettsia peacockii* | - | Rustic | composite transposon | cn_12053_ISRpe1 | 0,998216 | NC_012730.1 | IS481-like element ISRpe1 family transposase/protein_id="WP_012736375.1"; bifunctional methylenetetrahydrofolate dehydrogenase/methenyltetrahydrofolate cyclohydrolase FolD/protein_id="WP_012736376.1"; gamma carbonic anhydrase family protein/protein_id="WP_012736377.1"; ribosome hibernation-promoting factor, HPF/YfiA family/protein_id="WP_012736378.1"; hypothetical protein/protein_id="WP_012736379.1, WP_187145820.1, WP_228368863.1, WP_187145794.1, WP_041472339.1"; endopeptidase La/protein_id="WP_012736380.1"; serine/threonine dehydratase/protein_id="WP_012150822.1" |
| *Rickettsia peacockii* | - | Rustic | composite transposon | cn_28805_ISRpe1 | 0,996432 | NC_012730.1 | IS481-like element ISRpe1 family transposase/protein_id="WP_012736375.1"; hypothetical protein/protein_id="WP_146603356.1, WP_085065586.1, WP_228368864.1, WP_012736381.1, WP_012736382.1, WP_012150794.1, WP_012736385.1, WP_012736387.1, WP_012736388.1, WP_012736389.1"; DNA helicase PcrA/protein_id="WP_012736383.1"; glycosyltransferase/protein_id="WP_012736384.1"; NAD-dependent succinate-semialdehyde dehydrogenase/protein_id="WP_012736386.1"; thioredoxin-disulfide reductase/protein_id="WP_012736390.1"; alpha/beta fold hydrolase/protein_id="WP_012736391.1"; AEC family transporter/protein_id="WP_012736392.1"; NAD(P)H-dependent glycerol-3-phosphate dehydrogenase; ribonuclease J/protein_id="WP_010977243.1"; NAD kinase/protein_id="WP_012736393.1"; recombination mediator RecR/protein_id="WP_012736394.1"; RDD family protein/protein_id="WP_004995736.1"; YqaA family protein/protein_id="WP_012736395.1"; 30S ribosome-binding factor RbfA/protein_id="WP_012736396.1"; Tim44 domain-containing protein/protein_id="WP_012736397.1"; S9 family peptidase/protein_id="WP_012736398.1"; cytochrome b/protein_id="WP_014362974.1"; AbrB/MazE/SpoVT family DNA-binding domain-containing protein/protein_id="WP_012736399.1"; type II toxin-antitoxin system VapC family toxin/protein_id="WP_012736400.1"; ADP-forming succinate--CoA ligase subunit beta/protein_id="WP_012736401.1"; succinate--CoA ligase subunit alpha/protein_id="WP_012736402.1" |
| *Rickettsia peacockii* | - | Rustic | composite transposon | cn_30087_ISRpe1; cn_15437_ISRpe1; cn_6947_ISRpe1 | 0,995540 | NC_012730.1 | succinate--CoA ligase subunit alpha/protein_id="WP_012736402.1"; IS481-like element ISRpe1 family transposase/protein_id="WP_012736403.1, WP_012736421.1, WP_012736375.1"; NADH-quinone oxidoreductase subunit NuoN/protein_id="WP_012736404.1"; hypothetical protein/protein_id="WP_012736405.1, WP_228368865.1, WP_014120887.1, WP_012736426.1, WP_228368866.1, WP_085065590.1, WP_016830817.1, WP_228368867.1, WP_085065593.1, WP_014362268.1, WP_085065591.1, WP_014273092.1, WP_187145795.1, WP_014364812.1, WP_012736411.1, WP_012736429.1, WP_004998178.1, WP_012736423.1, WP_012736424.1"; porphobilinogen synthase/protein_id="WP_012736406.1"; primosomal protein N'/protein_id="WP_012736408.1"; UbiX family flavin prenyltransferase/protein_id="WP_012736409.1"; replicative DNA helicase/protein_id="WP_012736410.1"; aromatic amino acid transport family protein/protein_id="WP_085065588.1, WP_012150982.1"; to amino acid permease/protein_id="WP_014362372.1"; pseudouridine synthase/protein_id="WP_012736412.1"; effector protein SrfD/protein_id="WP_012736413.1"; RsmD family RNA methyltransferase/protein_id="WP_012736414.1"; DNA repair protein RadA/protein_id="WP_012736415.1"; DNA repair protein RecO/protein_id="WP_012736416.1"; tRNA (adenosine(37)-N6)-threonylcarbamoyltransferase complex dimerization subunit type 1 TsaB/protein_id="WP_012736417.1"; SrfC family effector protein/protein_id="WP_012736418.1"; translation initiation factor IF-2/protein_id="WP_012736419.1"; transcription termination factor NusA/protein_id="WP_012736420.1"; conjugal transfer protein TraV/protein_id="WP_085065589.1"; TraB/VirB10 family protein/protein_id="WP_012736422.1"; TraE/TraK family type IV conjugative transfer system protein/protein_id="WP_012736425.1"; leucine-rich repeat domain-containing protein/protein_id="WP_012736427.1"; tyrosine-type recombinase/integrase; ankyrin repeat domain-containing protein/protein_id="WP_012736428.1"; tRNA dihydrouridine synthase DusB/protein_id="WP_085065592.1"; tRNA (adenosine(37)-N6)-threonylcarbamoyltransferase complex ATPase subunit type 1 TsaE/protein_id="WP_012736432.1"; metal ABC transporter solute-binding protein, Zn/Mn family/protein_id="WP_012736433.1"; palindromic element RPE1 domain-containing protein; ComEC/Rec2 family competence protein/protein_id="WP_266105317.1"; TlyA family RNA methyltransferase/protein_id="WP_012736436.1" |
| *Rickettsia peacockii* | - | Rustic | composite transposon | cn_6849_ISRpe1 | 0,996432 | NC_012730.1 | IS481-like element ISRpe1 family transposase/protein_id="WP_012736375.1"; TlyA family RNA methyltransferase/protein_id="WP_012736436.1"; tyrosine--tRNA ligase/protein_id="WP_012736437.1"; CopG family antitoxin/protein_id="WP_085065594.1"; hypothetical protein/protein_id="WP_016830838.1" |
| *Rickettsia peacockii* | - | Rustic | composite transposon | cn_13759_ISRpe1 | 0,998216 | NC_012730.1 | IS481-like element ISRpe1 family transposase/protein_id="WP_012736375.1"; lipase family protein/protein_id="WP_410526303.1"; AAA family ATPase/protein_id="WP_085065595.1"; tetratricopeptide repeat protein/protein_id="WP_010976710.1"; hypothetical protein/protein_id="WP_085065596.1"; F0F1 ATP synthase subunit B/protein_id="WP_012736438.1"; F0F1 ATP synthase subunit B family protein/protein_id="WP_010976713.1"; F0F1 ATP synthase subunit C/protein_id="WP_010976714.1"; palindromic element RPE1 domain-containing protein; F0F1 ATP synthase subunit A/protein_id="WP_012736439.1"; AtpZ/AtpI family protein/protein_id="WP_012736440.1"; DsbA family protein/protein_id="WP_012736441.1"; CarD family transcriptional regulator/protein_id="WP_010976718.1"; methyltransferase regulatory domain-containing protein/protein_id="WP_012736442.1"; DNA replication/repair protein RecF/protein_id="WP_012736443.1"; CADD family putative folate metabolism protein; dihydrofolate reductase |
| *Rickettsia peacockii* | - | Rustic | composite transposon | cn_32608_ISRpe1 | 0,998165 | NC_012730.1 | dihydrofolate reductase; IS481-like element ISRpe1 family transposase/protein_id="WP_012736375.1"; dihydropteroate synthase; dihydroneopterin aldolase; DUF5394 family protein/protein_id="WP_012736444.1"; hypothetical protein/protein_id="WP_014273113.1, WP_012736451.1, WP_014273210.1, WP_085065600.1, WP_014419678.1, WP_187145796.1"; SCO family protein; YqgE/AlgH family protein/protein_id="WP_012736445.1"; CvpA family protein/protein_id="WP_012736446.1"; secreted effector lipase Rlip/protein_id="WP_012736447.1"; RlmE family RNA methyltransferase/protein_id="WP_012736448.1"; transcription antitermination factor NusB/protein_id="WP_012736449.1"; RIP metalloprotease RseP/protein_id="WP_004996601.1"; outer membrane protein assembly factor BamA/protein_id="WP_012736450.1"; DUF2674 domain-containing protein/protein_id="WP_085065602.1"; DHA2 family efflux MFS transporter permease subunit/protein_id="WP_012736452.1"; monovalent cation/H+ antiporter subunit E/protein_id="WP_004996606.1"; UMP kinase/protein_id="WP_004996607.1"; ribosome recycling factor/protein_id="WP_012736453.1"; nucleoside triphosphate pyrophosphohydrolase family protein/protein_id="WP_010976865.1"; Asp-tRNA(Asn)/Glu-tRNA(Gln) amidotransferase subunit GatC/protein_id="WP_012736454.1"; Asp-tRNA(Asn)/Glu-tRNA(Gln) amidotransferase subunit GatA/protein_id="WP_012736455.1"; Asp-tRNA(Asn)/Glu-tRNA(Gln) amidotransferase subunit GatB/protein_id="WP_012736456.1"; ABC transporter substrate-binding protein/protein_id="WP_012736457.1"; flavin reductase family protein/protein_id="WP_010976860.1"; 4-hydroxy-tetrahydrodipicolinate reductase/protein_id="WP_012736458.1"; Bax inhibitor-1/YccA family protein/protein_id="WP_012736459.1"; T4SS effector phosphatidylinositol 3-Kinase RisK1/protein_id="WP_012736460.1" |
| *Rickettsia peacockii* | - | Rustic | composite transposon | cn_36551_ISRpe1 | 0,998216 | NC_012730.1 | IS481-like element ISRpe1 family transposase/protein_id="WP_012736375.1"; hypothetical protein/protein_id="WP_146603319.1, WP_187145821.1, WP_155813506.1, WP_228368869.1, WP_085065792.1, WP_012736472.1, WP_012736480.1"; NB-ARC domain-containing protein/protein_id="WP_085065603.1"; outer membrane protein OmpB/protein_id="WP_012736461.1"; HD domain-containing protein/protein_id="WP_012736462.1"; beta-N-acetylhexosaminidase/protein_id="WP_012736463.1"; HU family DNA-binding protein/protein_id="WP_012736464.1"; thioredoxin-dependent thiol peroxidase/protein_id="WP_004997602.1"; lipid A biosynthesis lauroyl acyltransferase/protein_id="WP_012736465.1"; tetraacyldisaccharide 4'-kinase/protein_id="WP_012736466.1"; M23 family metallopeptidase/protein_id="WP_012736467.1"; 23S rRNA (adenine(2030)-N(6))-methyltransferase RlmJ/protein_id="WP_012736468.1"; RhuM family protein/protein_id="WP_228368906.1"; NAD-dependent DNA ligase LigA/protein_id="WP_012736469.1"; type II toxin-antitoxin system Phd/YefM family antitoxin/protein_id="WP_014364972.1"; tRNA guanosine(34) transglycosylase Tgt/protein_id="WP_012736470.1"; RC1041 family protein/protein_id="WP_228368907.1"; bifunctional (p)ppGpp synthetase/guanosine-3',5'-bis(diphosphate) 3'-pyrophosphohydrolase/protein_id="WP_085065604.1"; outer membrane lipid asymmetry maintenance protein MlaD/protein_id="WP_004997581.1"; NADH-ubiquinone oxidoreductase subunit NDUFA12 family protein/protein_id="WP_012736473.1"; ribonuclease HI/protein_id="WP_012736474.1"; SspB family protein/protein_id="WP_012736475.1"; LpxI family protein/protein_id="WP_012736476.1"; dephospho-CoA kinase/protein_id="WP_012736477.1"; DNA polymerase III subunit epsilon/protein_id="WP_004997573.1"; SURF1 family protein/protein_id="WP_012736478.1"; UvrD-helicase domain-containing protein/protein_id="WP_012736479.1"; ACP S-malonyltransferase/protein_id="WP_012736481.1"; IS481-like element ISRpe1 family transposase/protein_id="WP_012736482.1" |
| *Rickettsia peacockii* | - | Rustic | composite transposon | cn_8805_ISRpe1; cn_3391_ISRpe1; cn_35482_ISRpe1; cn_33163_ISRpe1; cn_12260_ISRpe1 | 0,999108 | NC_012730.1 | IS481-like element ISRpe1 family transposase/protein_id="WP_012736482.1, WP_012736483.1"; hypothetical protein/protein_id="WP_085065660.1, WP_228368871.1, WP_085065606.1, WP_085065607.1, WP_085065609.1, WP_085065610.1, WP_085065611.1, WP_228368872.1, WP_266105320.1, WP_085065612.1, WP_014273624.1, WP_085065664.1, WP_014410871.1, WP_085065665.1, WP_085065666.1, WP_085065667.1, WP_085065668.1, WP_004998070.1, WP_014364863.1, WP_146603335.1, WP_228368898.1, WP_012736710.1, WP_012736711.1, WP_085065678.1, WP_085065808.1, WP_228368899.1, WP_228368901.1, WP_187145777.1, WP_014419861.1, WP_228368795.1"; palindromic element RPE4 domain-containing protein/protein_id="WP_085065608.1"; AAA domain-containing protein/protein_id="WP_228368874.1"; ankyrin repeat domain-containing protein/protein_id="WP_012736679.1, WP_085065669.1"; excinuclease ABC subunit UvrC/protein_id="WP_012736680.1"; copper chaperone PCu(A)C/protein_id="WP_012736681.1"; DNA alkylation repair protein/protein_id="WP_085065661.1"; DNA alkylation repair protein; methylated-DNA--[protein]-cysteine S-methyltransferase; methylated-DNA-protein-cysteine methyltransferase; Ada metal-binding domain-containing protein/protein_id="WP_085065662.1"; methylated-DNA--[protein]-cysteine S-methyltransferase/protein_id="WP_085065663.1"; type I restriction enzyme subunit R domain-containing protein/protein_id="WP_085065805.1"; nucleotidyltransferase preprotein translocase subunit SecA/protein_id="WP_012736682.1"; foldase protein PrsA/protein_id="WP_012736683.1"; holo-ACP synthase/protein_id="WP_012262491.1"; DNA-directed RNA polymerase subunit omega/protein_id="WP_012736684.1"; UDP-N-acetylglucosamine 1-carboxyvinyltransferase/protein_id="WP_012736685.1"; DNA topoisomerase (ATP-hydrolyzing) subunit B/protein_id="WP_012736686.1"; TIGR01459 family HAD-type hydrolase/protein_id="WP_012736687.1"; magnesium transporter; HD domain-containing protein; proline/betaine transporter; MFS transporter/protein_id="WP_266105326.1"; antitoxin of toxin-antitoxin (TA) system/protein_id="WP_012736689.1"; palindromic element RPE1 domain-containing protein; preprotein translocase subunit YajC/protein_id="WP_012736690.1"; protein translocase subunit SecD/protein_id="WP_012736691.1"; SCO family protein/protein_id="WP_012736692.1"; cytochrome c maturation protein CcmE/protein_id="WP_012736693.1"; inorganic diphosphatase/protein_id="WP_012736694.1"; murein biosynthesis integral membrane protein MurJ/protein_id="WP_012736695.1"; TraX family protein/protein_id="WP_012736696.1"; ribonuclease P protein component/protein_id="WP_012736698.1"; 50S ribosomal protein L34/protein_id="WP_011270688.1, WP_012736699.1, WP_004997924.1"; 7-carboxy-7-deazaguanine synthase QueE/protein_id="WP_012736700.1"; 50S ribosomal protein L25/general stress protein Ctc/protein_id="WP_012736701.1"; aminoacyl-tRNA hydrolase/protein_id="WP_012736702.1"; N-acetyltransferase/protein_id="WP_085065673.1"; redox-regulated ATPase YchF/protein_id="WP_012736703.1"; sodium:solute symporter family protein/protein_id="WP_012736704.1"; division/cell wall cluster transcriptional repressor MraZ/protein_id="WP_012736706.1"; 16S rRNA (cytosine(1402)-N(4))-methyltransferase RsmH/protein_id="WP_012736707.1"; cell division protein FtsL/protein_id="WP_012736708.1"; peptidoglycan D,D-transpeptidase FtsI family protein/protein_id="WP_012736709.1"; penicillin-binding protein 2/protein_id="WP_010977459.1"; pentapeptide repeat-containing protein/protein_id="WP_012736712.1"; sigma-54-dependent transcriptional regulator/protein_id="WP_012719845.1"; monovalent cation/H+ antiporter complex subunit F/protein_id="WP_085065807.1"; 2-octaprenyl-6-methoxyphenyl hydroxylase/protein_id="WP_012736714.1"; ComF family protein/protein_id="WP_085065679.1, WP_228368900.1"; 3-hydroxyacyl-CoA dehydrogenase family protein/protein_id="WP_085065680.1, WP_014410844.1"; enoyl-CoA hydratase/isomerase family protein/protein_id="WP_085065681.1, WP_014419862.1"; alpha-2-macroglobulin family protein phosphatase PAP2 family protein/protein_id="WP_012736715.1"; leucine-rich repeat domain-containing protein/protein_id="WP_012736716.1"; nucleotidyltransferase domain-containing protein/protein_id="WP_004997933.1"; HEPN domain-containing protein/protein_id="WP_228368896.1, WP_228368897.1"; patatin-like phospholipase family protein |
| *Rickettsia peacockii* | - | Rustic | composite transposon | cn_46665_ISRpe1 | 0,998216 | NC_012730.1 | IS481-like element ISRpe1 family transposase/protein_id="WP_012736483.1, WP_012736514.1"; Rpn family recombination-promoting nuclease/putative transposase/protein_id="WP_228368875.1"; HlyD family secretion protein/protein_id="WP_012736484.1"; CDP-diacylglycerol--serine O-phosphatidyltransferase/protein_id="WP_010976983.1"; phosphatidylserine decarboxylase/protein_id="WP_012736485.1"; DUF2610 domain-containing protein/protein_id="WP_010976982.1"; inositol monophosphatase family protein/protein_id="WP_012736486.1"; elongation factor P/protein_id="WP_012736487.1"; PleD family two-component system response regulator/protein_id="WP_012736488.1"; RNA pyrophosphohydrolase/protein_id="WP_004996360.1"; 4a-hydroxytetrahydrobiopterin dehydratase/protein_id="WP_012736489.1"; peptidase M15; M15 family metallopeptidase domain-containing protein/protein_id="WP_010976975.1"; hypothetical protein/protein_id="WP_187145797.1, WP_012736501.1, WP_228368877.1, WP_228368878.1, WP_012736504.1, WP_085065615.1, WP_085065616.1, WP_085065617.1"; 30S ribosomal protein S9/protein_id="WP_012736490.1"; 50S ribosomal protein L13/protein_id="WP_004996373.1"; outer membrane protein assembly factor BamB/protein_id="WP_012736491.1"; DUF2659 family protein/protein_id="WP_012736492.1"; tol-pal system protein YbgF/protein_id="WP_012736493.1"; sensor histidine kinase/protein_id="WP_012736494.1"; S41 family peptidase/protein_id="WP_012736495.1"; DNA topoisomerase IV subunit B/protein_id="WP_012736496.1"; ankyrin repeat domain-containing protein/protein_id="WP_012736497.1, WP_228368880.1"; DUF2497 domain-containing protein/protein_id="WP_012736499.1"; TolC family protein/protein_id="WP_012736500.1"; DUF2660 domain-containing protein/protein_id="WP_012736503.1"; DUF8594 domain-containing protein/protein_id="WP_045805356.1"; ATP-binding cassette domain-containing protein/protein_id="WP_343230982.1, WP_146603322.1"; ABC transporter ATP-binding protein/protein_id="WP_228368879.1, WP_012736512.1"; threonine--tRNA ligase/protein_id="WP_012736505.1"; phosphoribosylaminoimidazolesuccinocarboxamide synthase/protein_id="WP_004996420.1"; M16 family metallopeptidase/protein_id="WP_012736507.1"; glycosyltransferase family 2 protein/protein_id="WP_012736508.1"; Txe/YoeB family addiction module toxin/protein_id="WP_012736509.1"; type II toxin-antitoxin system Phd/YefM family antitoxin/protein_id="WP_012150507.1"; cytochrome d ubiquinol oxidase subunit II/protein_id="WP_012736510.1"; cytochrome ubiquinol oxidase subunit I/protein_id="WP_012736511.1"; DUF2671 domain-containing protein/protein_id="WP_010976947.1"; tRNA preQ1(34) S-adenosylmethionine ribosyltransferase-isomerase QueA/protein_id="WP_012736513.1"; cell division protein ZapE; glycosyltransferase family 61 protein/protein_id="WP_012736515.1" |
| *Rickettsia peacockii* | - | Rustic | composite transposon | cn_26168_ISRpe1 | 0,996432 | NC_012730.1 | IS481-like element ISRpe1 family transposase/protein_id="WP_012736514.1"; glycosyltransferase family 61 protein/protein_id="WP_012736515.1"; hypothetical protein/protein_id="WP_012736516.1, WP_012736520.1, WP_187145798.1, WP_228368881.1"; methionyl-tRNA formyltransferase/protein_id="WP_012736517.1"; peptide deformylase/protein_id="WP_012150494.1, WP_085065619.1"; Fe-S cluster assembly protein IscX; HIG1 domain-containing protein/protein_id="WP_012736518.1"; cation:dicarboxylate symporter family transporter/protein_id="WP_010976935.1, WP_266105321.1"; DNA topoisomerase (ATP-hydrolyzing) subunit A/protein_id="WP_012736519.1"; ABCB family ABC transporter ATP-binding protein/permease glutaredoxin 3/protein_id="WP_012736521.1"; excinuclease ABC subunit UvrB/protein_id="WP_012736522.1"; ribonuclease HII/protein_id="WP_085065621.1"; Fe-S protein assembly co-chaperone HscB/protein_id="WP_012736524.1"; Fe-S protein assembly chaperone HscA/protein_id="WP_012736525.1"; ferredoxin family 2Fe-2S iron-sulfur cluster binding protein/protein_id="WP_012736526.1"; RP198 family tick cell line-upregulated protein/protein_id="WP_012736527.1"; TAXI family TRAP transporter solute-binding subunit/protein_id="WP_012262256.1"; universal stress protein/protein_id="WP_012736528.1" |
| *Rickettsia peacockii* | - | Rustic | composite transposon | cn_27106_ISRpe1 | 0,998216 | NC_012730.1 | IS481-like element ISRpe1 family transposase/protein_id="WP_012736545.1"; hypothetical protein/protein_id="WP_228368881.1, WP_012719454.1, WP_012150454.1, WP_014013930.1, WP_014419715.1"; 2,3,4,5-tetrahydropyridine-2,6-dicarboxylate N-succinyltransferase/protein_id="WP_012736529.1"; MJ0042-type zinc finger domain-containing protein/protein_id="WP_012736530.1"; TrbC/VirB2 family type IV secretion system protein/protein_id="WP_012736531.1"; cytochrome c oxidase subunit 3/protein_id="WP_004996526.1"; demethoxyubiquinone hydroxylase family protein/protein_id="WP_012736532.1"; DNA polymerase III subunit delta/protein_id="WP_012736533.1"; S1C family serine protease/protein_id="WP_085065623.1"; molecular chaperone DnaK/protein_id="WP_012736535.1"; molecular chaperone DnaJ/protein_id="WP_012736536.1"; ChaB family protein/protein_id="WP_016830649.1"; palindromic element RPE1 domain-containing protein; outer membrane protein assembly factor BamD/protein_id="WP_012736537.1"; DNA repair protein RecN/protein_id="WP_012736538.1"; carboxypeptidase M32/protein_id="WP_012736539.1"; 2-oxoglutarate dehydrogenase E1 component/protein_id="WP_012736540.1"; 2-oxoglutarate dehydrogenase complex dihydrolipoyllysine-residue succinyltransferase/protein_id="WP_012736541.1"; 6-pyruvoyl tetrahydropterin synthase family protein/protein_id="WP_012736542.1"; divalent-cation tolerance protein CutA/protein_id="WP_012736543.1"; dicarboxylate/amino acid:cation symporter/protein_id="WP_012736544.1" |
| *Rickettsia peacockii* | - | Rustic | composite transposon | cn_41600_ISRpe1; cn_10734_ISRpe1 | 0,996432 | NC_012730.1 | IS481-like element ISRpe1 family transposase/protein_id="WP_012736545.1, WP_012736634.1, WP_012736483.1"; hypothetical protein/protein_id="WP_004996338.1, WP_266105322.1, WP_012150563.1, WP_085065627.1, WP_187145799.1, WP_266105323.1, WP_187145822.1, WP_085065796.1, WP_014410506.1, WP_228368882.1, WP_410526306.1, WP_012736638.1, WP_187145805.1"; BolA family protein/protein_id="WP_012736546.1"; EAL domain-containing protein/protein_id="WP_085065624.1"; UDP-N-acetylmuramate--L-alanine ligase/protein_id="WP_012736548.1"; UDP-N-acetylmuramate dehydrogenase/protein_id="WP_012736549.1"; D-alanine--D-alanine ligase/protein_id="WP_012736550.1"; cell division protein FtsQ/DivIB/protein_id="WP_012736551.1"; cell division protein FtsA/protein_id="WP_012736552.1"; NfeD family protein/protein_id="WP_085065625.1"; c-type cytochrome/protein_id="WP_012736554.1"; UDP-3-O-acyl-N-acetylglucosamine deacetylase/protein_id="WP_012719516.1"; palindromic element RPE1 domain-containing protein; MFS transporter/protein_id="WP_012736556.1"; Rne/Rng family ribonuclease/protein_id="WP_012736557.1"; COX15/CtaA family protein/protein_id="WP_014410489.1"; RluA family pseudouridine synthase/protein_id="WP_012736559.1"; serine hydrolase domain-containing protein/protein_id="WP_012736560.1"; exodeoxyribonuclease III/protein_id="WP_012736561.1"; pyruvate dehydrogenase (acetyl-transferring) E1 component subunit alpha/protein_id="WP_004996289.1"; pyruvate dehydrogenase complex E1 component subunit beta/protein_id="WP_012736562.1"; translational GTPase TypA/protein_id="WP_012736563.1"; OmpH family outer membrane protein/protein_id="WP_010977007.1"; NADP-dependent isocitrate dehydrogenase/protein_id="WP_012736564.1"; Na+/H+ antiporter subunit G/protein_id="WP_004996272.1"; DUF4040 domain-containing protein/protein_id="WP_012736565.1"; heme exporter protein CcmB/protein_id="WP_012150568.1"; DUF2670 domain-containing protein/protein_id="WP_012736566.1"; ubiquinol-cytochrome c reductase iron-sulfur subunit/protein_id="WP_004996261.1"; cytochrome b/protein_id="WP_012736567.1"; cytochrome c1/protein_id="WP_012736568.1"; Hsp20/alpha crystallin family protein/protein_id="WP_012736569.1"; PDDEXK nuclease domain-containing protein; peptide chain release factor 2/protein_id="WP_146603327.1"; translation elongation factor 4/protein_id="WP_012736571.1"; DNA adenine methylase; helix-turn-helix transcriptional regulator/protein_id="WP_012736635.1"; (d)CMP kinase/protein_id="WP_012719757.1"; 30S ribosomal protein S1/protein_id="WP_012736636.1"; ATP-dependent Clp endopeptidase proteolytic subunit ClpP/protein_id="WP_012736637.1"; type 2 isopentenyl-diphosphate Delta-isomerase/protein_id="WP_012736639.1" |
| *Rickettsia peacockii* | - | Rustic | composite transposon | cn_43088_ISRpe1 | 0,997248 | NC_012730.1 | IS481-like element ISRpe1 family transposase/protein_id="WP_012736717.1, WP_012736747.1"; aspartate--tRNA ligase; hypothetical protein/protein_id="WP_012736719.1"; division plane positioning ATPase MipZ/protein_id="WP_012736720.1"; leucyl aminopeptidase/protein_id="WP_012736721.1"; palindromic element RPE1 domain-containing protein/protein_id="WP_012736722.1"; DNA-directed RNA polymerase subunit beta'/protein_id="WP_012736723.1"; DNA-directed RNA polymerase subunit beta/protein_id="WP_012736724.1"; 50S ribosomal protein L7/L12/protein_id="WP_085065809.1"; 50S ribosomal protein L10/protein_id="WP_012736726.1"; 50S ribosomal protein L1/protein_id="WP_010976849.1"; 50S ribosomal protein L11/protein_id="WP_012736727.1"; transcription termination/antitermination protein NusG/protein_id="WP_012736728.1"; preprotein translocase subunit SecE/protein_id="WP_004996644.1"; elongation factor G/protein_id="WP_012736730.1"; 30S ribosomal protein S7/protein_id="WP_004996646.1"; 30S ribosomal protein S12/protein_id="WP_012736731.1"; amino acid ABC transporter permease/protein_id="WP_004996648.1"; succinate dehydrogenase flavoprotein subunit/protein_id="WP_012736732.1"; succinate dehydrogenase, hydrophobic membrane anchor protein/protein_id="WP_012736733.1"; succinate dehydrogenase, cytochrome b556 subunit/protein_id="WP_012736734.1"; oxygen-dependent tRNA uridine(34) hydroxylase TrhO/protein_id="WP_012736735.1"; Do family serine endopeptidase/protein_id="WP_012736736.1"; protease modulator HflC/protein_id="WP_004996654.1"; FtsH protease activity modulator HflK/protein_id="WP_012736737.1"; Mrp/NBP35 family ATP-binding protein/protein_id="WP_012736738.1"; mitochondrial fission ELM1 family protein/protein_id="WP_012736739.1"; nucleotidyltransferase substrate binding protein/protein_id="WP_228368796.1"; RNA methyltransferase/protein_id="WP_012736741.1"; crossover junction endodeoxyribonuclease RuvC/protein_id="WP_004996663.1"; GTPase Era/protein_id="WP_012736742.1"; ribonuclease III/protein_id="WP_012736743.1"; signal peptidase I/protein_id="WP_012736744.1"; NADH-quinone oxidoreductase subunit NuoF/protein_id="WP_012736745.1"; protein translocase subunit SecF/protein_id="WP_012736746.1"; DUF2312 domain-containing protein/protein_id="WP_004996682.1" |
| *Rickettsia peacockii* | - | Rustic | composite transposon | cn_13226_ISRpe1 | 0,998216 | NC_012730.1 | IS481-like element ISRpe1 family transposase/protein_id="WP_012736747.1, WP_012736482.1"; chromosomal replication initiator protein DnaA/protein_id="WP_012736748.1"; rhodanese-like domain-containing protein/protein_id="WP_012736749.1"; succinate dehydrogenase assembly factor 2/protein_id="WP_012151075.1"; transcription-repair coupling factor/protein_id="WP_012736750.1"; UDP-N-acetylmuramoyl-L-alanyl-D-glutamate--2,6-diaminopimelate ligase/protein_id="WP_012736751.1"; UDP-N-acetylmuramoyl-tripeptide--D-alanyl-D-alanine ligase/protein_id="WP_012736752.1"; phospho-N-acetylmuramoyl-pentapeptide-transferase/protein_id="WP_012736753.1" |
| *Rickettsia peacockii* | - | Rustic | composite transposon | cn_7973_ISRpe1 | 1,000000 | NC_012730.1 | IS481-like element ISRpe1 family transposase/protein_id="WP_012736482.1, WP_012736375.1"; hypothetical protein/protein_id="WP_228368797.1, WP_228368798.1, WP_014419753.1, WP_228368799.1"; HD domain-containing protein/protein_id="WP_012736755.1"; MFS transporter/protein_id="WP_343230983.1"; rod shape-determining protein RodA/protein_id="WP_012736756.1" |
| *Rickettsia peacockii* | - | Rustic | composite transposon | cn_29087_ISRpe1 | 0,996432 | NC_012730.1 | IS481-like element ISRpe1 family transposase/protein_id="WP_012736375.1"; tryptophan--tRNA ligase/protein_id="WP_012736799.1"; lysophospholipid acyltransferase family protein/protein_id="WP_012262415.1"; host attachment protein/protein_id="WP_012736800.1"; alpha/beta hydrolase/protein_id="WP_012736801.1"; tetratricopeptide repeat protein/protein_id="WP_012736802.1"; palindromic element RPE2 domain-containing protein/protein_id="WP_012736803.1"; uroporphyrinogen-III synthase/protein_id="WP_012736804.1"; LysE/ArgO family amino acid transporter/protein_id="WP_012736805.1"; hypothetical protein/protein_id="WP_012150903.1, WP_004998351.1, WP_004998335.1, WP_012736808.1, WP_085065702.1, WP_228368806.1, WP_085065703.1"; AmpG family muropeptide MFS transporter/protein_id="WP_085065814.1"; transposase/protein_id="WP_085065700.1"; Rpn family recombination-promoting nuclease/putative transposase/protein_id="WP_228368917.1"; glycosyltransferase family 8 protein nucleotide exchange transporter Tlc3/protein_id="WP_012736807.1"; polyprenyl synthetase family protein/protein_id="WP_085065815.1"; aminopeptidase P family protein/protein_id="WP_012736810.1"; lytic transglycosylase domain-containing protein; APC family permease/protein_id="WP_012736811.1"; LysM peptidoglycan-binding domain-containing protein; iron-sulfur cluster assembly accessory protein/protein_id="WP_004998325.1"; Fe-S cluster assembly scaffold IscU/protein_id="WP_010977348.1"; IscS subfamily cysteine desulfurase/protein_id="WP_012736812.1"; cysteine desulfurase family protein/protein_id="WP_012736813.1"; Fe-S cluster assembly transcription factor/protein_id="WP_012150915.1"; DUF1189 family protein/protein_id="WP_012736814.1"; PDDEXK nuclease domain-containing protein; GNAT family N-acetyltransferase/protein_id="WP_012736815.1"; acetyltransferase/protein_id="WP_012719761.1" |
| *Rickettsia peacockii* | - | Rustic | composite transposon | cn_8906_ISRpe1 | 0,998216 | NC_012730.1 | IS481-like element ISRpe1 family transposase/protein_id="WP_012736375.1"; type IV secretion system DNA-binding domain-containing protein; Rpn family recombination-promoting nuclease/putative transposase; toprim domain-containing protein; conjugal transfer protein TraD; MFS transporter/protein_id="WP_266105305.1, WP_266105306.1" |
| *Rickettsia peacockii* | - | Rustic | composite transposon | cn_46647_ISRpe1 | 0,995540 | NC_012730.1 | IS481-like element ISRpe1 family transposase; DNA polymerase III subunit alpha/protein_id="WP_012736816.1"; UDP-glucose dehydrogenase family protein/protein_id="WP_012736817.1"; YhdP family protein/protein_id="WP_012736818.1"; MFS transporter/protein_id="WP_228368808.1"; twin-arginine translocase subunit TatC/protein_id="WP_012736820.1"; serine--tRNA ligase/protein_id="WP_012736821.1"; palindromic element RPE5 domain-containing protein/protein_id="WP_014411077.1"; VirB4 family type IV secretion/conjugal transfer ATPase/protein_id="WP_012736822.1"; hypothetical protein/protein_id="WP_228368810.1, WP_012736823.1, WP_228368809.1"; TerC/Alx family metal homeostasis membrane protein/protein_id="WP_012736824.1"; outer membrane protein/protein_id="WP_012736825.1"; methyltransferase regulatory domain-containing protein/protein_id="WP_012736826.1"; helix-turn-helix domain-containing protein/protein_id="WP_012736828.1"; NADH-quinone oxidoreductase subunit J/protein_id="WP_010977789.1"; NADH-quinone oxidoreductase subunit NuoK/protein_id="WP_012151334.1"; NADH-quinone oxidoreductase subunit L/protein_id="WP_085065706.1"; NADH-quinone oxidoreductase subunit M/protein_id="WP_012736830.1"; heme ABC exporter ATP-binding protein CcmA/protein_id="WP_012151337.1"; NADH-quinone oxidoreductase subunit NuoI/protein_id="WP_012736832.1"; NADH-quinone oxidoreductase subunit NuoH/protein_id="WP_012736833.1"; NADH-quinone oxidoreductase subunit NuoG/protein_id="WP_085065818.1"; DUF4870 family protein/protein_id="WP_004997331.1"; aconitate hydratase AcnA/protein_id="WP_012736835.1"; F0F1 ATP synthase subunit epsilon/protein_id="WP_012736836.1"; F0F1 ATP synthase subunit beta/protein_id="WP_012736837.1"; ATP synthase F1 subunit gamma/protein_id="WP_012736838.1"; F0F1 ATP synthase subunit alpha/protein_id="WP_012736839.1"; ATP synthase F1 subunit delta/protein_id="WP_012736840.1"; dihydrolipoyl dehydrogenase/protein_id="WP_012736841.1"; Dps family protein/protein_id="WP_012151348.1" |
| *Rickettsia peacockii* | - | Rustic | composite transposon | cn_17095_ISRpe1 | 0,994648 | NC_012730.1 | IS481-like element ISRpe1 family transposase/proteinid="WP012736866.IS481-like element ISRpe1 family transposase/protein_id="WP_012736866.1"; hydroxymethylbilane synthase/protein_id="WP_012736867.1"; LD-carboxypeptidase/protein_id="WP_012736868.1"; sodium:solute symporter family transporter; ankyrin repeat domain-containing protein/protein_id="WP_228368822.1"; hypothetical protein/protein_id="WP_012736869.1, WP_014273554.1, WP_014364794.1, WP_012736872.1, WP_012736876.1"; CAF17-like 4Fe-4S cluster assembly/insertion protein YgfZ/protein_id="WP_012736870.1"; RMD1 family protein/protein_id="WP_012150942.1"; ribonuclease D/protein_id="WP_012736871.1"; 5-formyltetrahydrofolate cyclo-ligase/protein_id="WP_012736873.1"; dihydrolipoyl dehydrogenase/protein_id="WP_012736874.1"; methyltransferase domain-containing protein/protein_id="WP_228368922.1"; PD-(D/E)XK nuclease superfamily protein; transposase/protein_id="WP_266105308.1" |
| *Rickettsia peacockii* | - | Rustic | composite transposon | cn_4080_ISRpe1 | 0,995540 | NC_012730.1 | IS481-like element ISRpe1 family transposase; HigA family addiction module antitoxin/protein_id="WP_085065718.1"; autotransporter outer membrane beta-barrel domain-containing protein/protein_id="WP_317133660.1"; hypothetical protein/protein_id="WP_187145781.1, WP_085065719.1"; NACHT domain-containing protein/protein_id="WP_228368824.1"; IS481 family transposase |
| *Rickettsia peacockii* | - | Rustic | composite transposon | cn_6707_ISRpe1; cn_7463_ISRpe1 | 0,996432 | NC_012730.1 | IS481 family transposase; nucleotidyltransferase family protein/protein_id="WP_004997297.1"; HEPN domain-containing protein/protein_id="WP_266105339.1"; tRNA (N6-isopentenyl adenosine(37)-C2)-methylthiotransferase MiaB/protein_id="WP_012736877.1"; penicillin-binding protein 1A/protein_id="WP_012736878.1"; hypothetical protein/protein_id="WP_343230978.1, WP_012736882.1, WP_012736881.1"; IS481-like element ISRpe1 family transposase/protein_id="WP_012736483.1, WP_012736747.1"; sugar phosphate nucleotidyltransferase/protein_id="WP_012736879.1"; YebC/PmpR family DNA-binding transcriptional regulator/protein_id="WP_012736880.1"; type B 50S ribosomal protein L36/protein_id="WP_004998227.1"; lytic transglycosylase domain-containing protein/protein_id="WP_012736883.1"; DUF2532 domain-containing protein/protein_id="WP_012719801.1" |
| *Rickettsia peacockii* | - | Rustic | composite transposon | cn_13810_ISRpe1 | 0,994648 | NC_012730.1 | IS481-like element ISRpe1 family transposase/protein_id="WP_012736375.1"; ankyrin repeat domain-containing protein/protein_id="WP_085065738.1"; hypothetical protein/protein_id="WP_004995953.1, WP_228368831.1, WP_228368830.1"; disulfide bond formation protein B/protein_id="WP_012736929.1"; lysine--tRNA ligase/protein_id="WP_012736930.1"; AEC family transporter/protein_id="WP_012736931.1"; NADP-dependent malic enzyme/protein_id="WP_012736932.1"; DUF2748 family protein/protein_id="WP_012736933.1"; autotransporter domain-containing protein/protein_id="WP_187145784.1" |
| *Rickettsia peacockii* | - | Rustic | composite transposon | cn_5004_ISRpe1 | 0,998216 | NC_012730.1 | IS481-like element ISRpe1 family transposase/protein_id="WP_012736375.1"; hypothetical protein/protein_id="WP_228368831.1"; MFS transporter/protein_id="WP_012736934.1"; AbrB/MazE/SpoVT family DNA-binding domain-containing protein/protein_id="WP_010977150.1"; type II toxin-antitoxin system VapC family toxin/protein_id="WP_012150708.1"; |
| *Rickettsia peacockii* | - | Rustic | composite transposon | cn_43934_ISRpe1 | 0,995540 | NC_012730.1 | IS481-like element ISRpe1 family transposase/protein_id="WP_012737045.1"; hypothetical protein/protein_id="WP_012737084.1, WP_085065768.1, WP_187145789.1, WP_014273665.1, WP_014410916.1, WP_085065774.1, WP_187145790.1, WP_343230988.1, WP_146603349.1, WP_085065772.1, WP_085065770.1, WP_228368845.1, WP_228368850.1, WP_085065778.1, WP_004997861.1, WP_146603352.1"; magnesium transporter CorA family protein/protein_id="WP_012737030.1"; 16S ribosomal RNA phosphotransferase/protein_id="WP_228368934.1"; sensor histidine kinase NtrY-like/protein_id="WP_012737031.1"; 30S ribosomal protein S21/protein_id="WP_012148483.1"; COQ9 family protein/protein_id="WP_012737032.1"; isoleucine--tRNA ligase/protein_id="WP_012737033.1"; ankyrin repeat domain-containing protein; tetratricopeptide repeat protein/protein_id="WP_085065773.1"; acetyl-CoA carboxylase biotin carboxylase subunit/protein_id="WP_012151112.1"; acyl-CoA carboxylase subunit beta/protein_id="WP_012737034.1"; acyl-[ACP]--phospholipid O-acyltransferase/protein_id="WP_012737035.1"; TfoX/Sxy family protein/protein_id="WP_012737036.1"; metal ABC transporter permease/protein_id="WP_012737037.1"; bifunctional 2-polyprenyl-6-hydroxyphenol methylase/3-demethylubiquinol 3-O-methyltransferase UbiG/protein_id="WP_012737038.1"; glutamate--tRNA ligase/protein_id="WP_012737039.1"; invasion associated locus B family protein/protein_id="WP_012737040.1"; lytic polysaccharide monooxygenase/protein_id="WP_228368936.1"; chaperonin GroEL/protein_id="WP_012151120.1"; co-chaperone GroES/protein_id="WP_012737041.1"; glycosyltransferase family 2 protein/protein_id="WP_228368846.1"; glycosyltransferase family protein/protein_id="WP_228368847.1"; ribonuclease PH/protein_id="WP_010977568.1"; nucleotide exchange factor GrpE/protein_id="WP_010977570.1"; AI-2E family transporter/protein_id="WP_012737043.1"; HdaA/DnaA family protein/protein_id="WP_012737044.1"; MFS transporter/protein_id="WP_085065777.1"; MFS sugar transporter/protein_id="WP_228368849.1" |
| *Rickettsia peacockii* | - | Rustic | composite transposon | cn_1804_ISRpe1 | 0,996432 | NC_012730.1 | IS481-like element ISRpe1 family transposase/protein_id="WP_012737045.1" |
| *Rickettsia peacockii* | - | Rustic | composite transposon | cn_12839_ISRpe1 | 0,998216 | NC_012730.1 | CopG family ribbon-helix-helix protein; acyl-ACP--UDP-N-acetylglucosamine O-acyltransferase/protein_id="WP_012736336.1"; 3-hydroxyacyl-ACP dehydratase FabZ/protein_id="WP_004997000.1"; UDP-3-O-(3-hydroxymyristoyl)glucosamine N-acyltransferase/protein_id="WP_012736337.1"; hypothetical protein/protein_id="WP_350223204.1, WP_085065571.1, WP_016916841.1, WP_085065572.1"; patatin-like phospholipase family protein/protein_id="WP_228368857.1"; YaaA family protein/protein_id="WP_012736339.1"; HU family DNA-binding protein/protein_id="WP_012150884.1"; signal peptide peptidase SppA; transcription termination factor Rho/protein_id="WP_012736340.1"; class I SAM-dependent methyltransferase; single-stranded-DNA-specific exonuclease RecJ/protein_id="WP_012736341.1"; peptide chain release factor 1/protein_id="WP_012736342.1" |
| *Rickettsia peacockii* | - | Rustic | insertion sequence | ISRpe1 | 0,99554; 0,994648; | NC_012730.1 | IS481-like element ISRpe1 family transposase/protein_id="WP_012736866.1, WP_012736403.1, WP_012736421.1, WP_012736375.1"; tRNA (adenosine(37)-N6)-threonylcarbamoyltransferase complex ATPase subunit type 1 TsaE/protein_id="WP_012736432.1"; succinate--CoA ligase subunit alpha/protein_id="WP_012736402.1"; conjugal transfer protein TraV/protein_id="WP_085065589.1"; single-stranded-DNA-specific exonuclease RecJ/protein_id="WP_012736341.1"; peptide chain release factor 1/protein_id="WP_012736342.1" |
| *Rickettsia peacockii* | - | Rustic | insertion sequence | ISRpe1 | 0,996432 | NC_012730.1 | IS481-like element ISRpe1 family transposase/protein_id="WP_012736545.1, WP_012736634.1, WP_012736483.1, WP_012736375.1, WP_012737045.1, WP_012736514.1"; TlyA family RNA methyltransferase/protein_id="WP_012736436.1"; glycosyltransferase family 61 protein/protein_id="WP_012736515.1"; hypothetical protein/protein_id="WP_343230978.1" |
| *Rickettsia peacockii* | - | Rustic | insertion sequence | ISRpe1 | 0,997324; 0,997319; 0,997248 | NC_012730.1 | IS481-like element ISRpe1 family transposase/protein_id="WP_012736483.1, WP_012736717.1, WP_012736545.1, WP_012736747.1"; hypothetical protein/protein_id="WP_187145805.1, WP_228368882.1" |
| *Rickettsia peacockii* | - | Rustic | insertion sequence | ISRpe1 | 0,998216; 0,998165 | NC_012730.1 | IS481-like element ISRpe1 family transposase/protein_id="WP_012736375.1, WP_012736747.1, WP_012736483.1"; hypothetical protein/protein_id="WP_228368831.1, WP_228368881.1"; CopG family ribbon-helix-helix protein; acyl-ACP--UDP-N-acetylglucosamine O-acyltransferase/protein_id="WP_012736336.1"; 3-hydroxyacyl-ACP dehydratase FabZ/protein_id="WP_004997000.1" |
| *Rickettsia peacockii* | - | Rustic | insertion sequence | ISRpe1 | 0,999108 | NC_012730.1 | IS481-like element ISRpe1 family transposase/protein_id="WP_012736482.1, WP_012736375.1"; hypothetical protein/protein_id="WP_085065660.1"; ribonuclease P protein component/protein_id="WP_012736698.1" |
| *Rickettsia peacockii* | - | Rustic | insertion sequence | ISRpe1 | 1,000000 | NC_012730.1 | IS481-like element ISRpe1 family transposase/protein_id="WP_012736482.1" |
| *Rickettsia rhipicephali* | - | HJ#5 | insertion sequence | ISRpe1 | 0,939964 | NZ_CP013133.1 | IS481 family transposase/protein_id="WP_057700078.1" |
| *Rickettsia tamurae* | buchneri | - | insertion sequence | ISRpe1 | 0,943223 | NZ_CP113531.1 | IS481 family transposase /protein_id="WP_008579339.1" |
| *Rickettsia tamurae* | buchneri | ISO7 | insertion sequence | ISRpe1 | 0,941229 | NZ_JFKF01000136.1 | IS481 family transposase /protein_id="WP_008579339.1" |

Source: By author, 2025. Note: Results obtained using MEFinder, showing the type of mobile genetic element (MGE), MGE name, sequence identity to the reference element, contig number, and the functional annotation of associated protein products retrieved from the NCBI Nucleotide database. The "-" sign represents fields for which information was not available or not applicable.
